# Boron-Mediated Hydroalkylation of Unactivated Olefins: An *Anti*-Markovnikov Approach to Congested Carbon Centers

**DOI:** 10.1021/jacs.6c01091

**Published:** 2026-03-13

**Authors:** Hanwen Zhang, Ruocheng Sang, Gianluca Simionato, Jasper L. Tyler, Adam Noble, Varinder K. Aggarwal

**Affiliations:** School of Chemistry, 1980 University of Bristol, Cantock’s Close, Bristol BS8 1TS, U.K.

## Abstract

Transition metal-catalyzed hydroalkylations offer efficient routes to install alkyl fragments onto readily available olefins. However, the formation of quaternary carbon centers through olefin hydroalkylations with tertiary alkyl electrophiles remains a formidable synthetic challenge. Difficulty arises as a result of steric congestion, which slows the rate of oxidative addition and promotes deleterious β-hydride elimination. Here, we report a boron-mediated coupling strategy using nickel/photoredox dual catalysis that circumvents these limitations and enables *anti*-Markovnikov hydroalkylations of unactivated olefins with carboxylic acid derivatives as alkylating partners. Our protocol proceeds via initial hydroboration, followed by a sequence of deboronative and decarboxylative radical generation, radical sorting, and bimolecular homolytic substitution (S_H_2). This mild and practical approach allows the construction of densely substituted carbon frameworks, including sterically congested quaternary carbon centers, providing a solution to a long-standing challenge in fragment-coupling chemistry.

## Graphical abstract

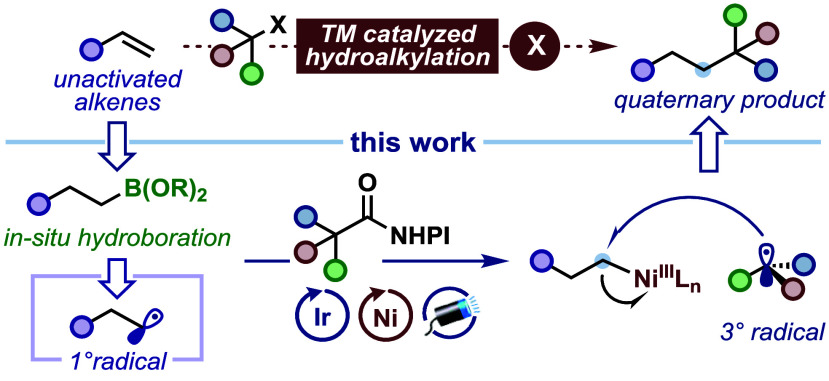

## Introduction

Transition metal-catalyzed cross-couplings between alkyl nucleophiles and electrophiles represent a powerful approach for C­(sp^3^)–C­(sp^3^) bond formation,^1^ enabling direct access to medicinally valuable sp^3^-rich architectures from simple precursors.^2^ While these methods previously relied on the use of air- and moisture-sensitive organometallic species,^3^ recent advances have expanded the diversity of C­(sp^3^)-nucleophilic coupling partners to include readily available and bench-stable functional groups, such as carboxylic acids, alcohols, and amines.^4,5^ An attractive alternative strategy for C­(sp^3^)–C­(sp^3^) cross-couplings is the use of unactivated olefins as latent nucleophiles, where metal hydride-catalyzed hydrometalation or hydrogen atom transfer forms alkyl metal intermediates that subsequently react with alkyl electrophiles, giving hydroalkylation products with *anti*-Markovnikov or Markovnikov selectivity, respectively (Figure 1a).^6,7^ This approach can provide improvements in atom economy compared to conventional cross-couplings of alkyl nucleophiles, since the generation of stoichiometric C­(sp^3^) nucleophiles is avoided. However, limitations currently exist with respect to the use of tertiary alkyl electrophiles,^8,9^ a consequence of their steric congestion, which impedes oxidative addition and promotes deleterious β-hydride elimination.^10^ As a result, introducing quaternary carbon centers via metal hydride-catalyzed olefin hydroalkylations is limited to the use of activated tertiary alkyl halides (e.g., α-carbonyl),^8a^ whereas the use of unactivated tertiary alkyl electrophiles remains a significant challenge.^9,11^

**Figure 1:**
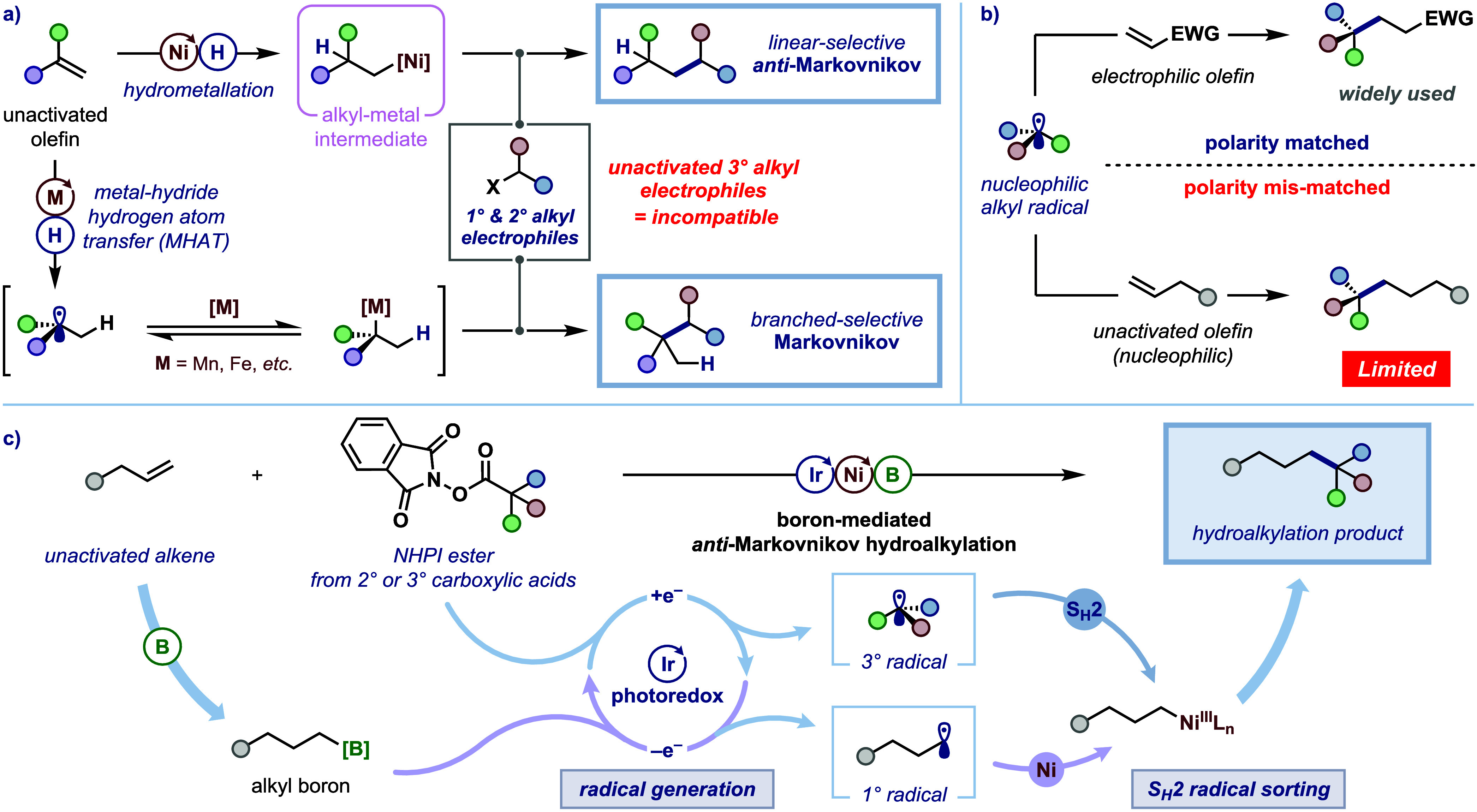
Hydroalkylation of terminal alkenes. (**a**) Transition-metal-catalyzed Markovnikov and *anti*-Markovnikov hydroalkylations of olefins. (**b**) Radical additions to olefins (EWG = electron-withdrawing group). (**c**) This work: boron-mediated *anti*-Markovnikov hydroalkylation of unactivated olefins with tertiary alkyl electrophiles using nickel/photoredox dual catalysis.

A mechanistically distinct alternative to metal hydride-catalyzed *anti*-Markovnikov hydroalkylations for introducing quaternary carbon centers onto olefins is via the addition of tertiary alkyl radicals (Figure 1b).^12^ However, for reactions of nonstabilized alkyl radicals, which are nucleophilic, electrophilic olefins are required. This is due to the necessity for matching the polarity between the radical and the olefin for kinetically favored addition.^13^ Therefore, for unactivated olefins, which are nucleophilic, reactions with nonstabilized alkyl radicals fail because of mismatched polarity.

To provide a broadly applicable solution to *anti*-Markovnikov hydroalkylations of unactivated olefins with unactivated tertiary alkyl electrophiles, we envisioned a photoredox-catalyzed strategy that integrates hydrometalation and radical functionalization. Inspiration for our mechanistic design came from recent reports of constructing C­(sp^3^)–C­(sp^3^) bonds through bimolecular homolytic substitution (S_H_2),^14^ which allows selective cross-coupling of differentially substituted radicals through a radical sorting regime.^10a,15^ Selectivity arises due to the preferential capture of the less hindered primary radical with a transition metal catalyst to form an organometallic complex capable of undergoing outer-sphere S_H_2 with the more hindered radical.^10a,14^ We hypothesized that this powerful method could be harnessed to couple readily accessible tertiary alkyl radicals with primary alkyl radicals generated from unactivated olefins via hydroboration and radical deboronation.^16^ Herein, we describe the development of a deboronative S_H_2 cross-coupling of primary alkyl boronic acids with *N*-hydroxyphthalimide (NHPI) esters enabled by nickel/photoredox dual catalysis (Figure 1c).^17^ When integrated with olefin hydroboration, this provides a unique *anti*-Markovnikov hydroalkylation of unactivated olefins with carboxylic acids, which represents a formal polarity mismatched addition of nucleophilic radicals to nucleophilic olefins.

## Results and Discussion

We began our investigations by establishing the nickel/photoredox-catalyzed deboronative cross-coupling of boronic acids with tertiary alkyl NHPI esters, using phenethylboronic acid (**1a**), the hydroboration product of styrene, and NHPI ester **2** as model substrates (Table 1). After extensive optimization (see Section 2.3 of the Supporting Information for further details), we found that a combination of catechol (1.0 equiv), [Ir­(*d*F­(Me)­ppy)_2_(*dt*bbpy)]­PF_6_ (**Ir-1**, 2.0 mol %), Ni­(acac)^_2_^ (20 mol %), and 3-quinuclidinol (1.0 equiv) in DMSO (*c* = 0.33 M) under irradiation with blue light gave the desired quaternary product **3** in 63% yield. These conditions provided effective radical sorting, since <10% of 1,4-diphenylbutane, the product of homocoupling of boronic acid **1a**, was observed. Evaluation of other reaction parameters, including solvent, nickel catalyst, photocatalyst, and base, did not lead to any improvements in the yield of **3** (entries 2–6 and Tables S2–S5). An iron-porphyrin catalyst (Fe­(OEP)­Cl),^14a^ and Ni­(acac)_2_ combined with scorpionate ligand K­[Tp*] (potassium tri­(3,5-dimethyl-1-pyrazolyl)­borohydride),^14b^ both previously reported to facilitate radical-sorting processes, resulted in diminished yields (entry 4 and Table S5). Notably, the inexpensive organic photoredox catalyst 4CzIPN was also effective but gave a slightly reduced yield compared to **Ir-1** (entry 6). The nature of the organoboron species had a substantial influence on the reaction outcome, with catechol-derived boronic ester **1b** enabling productive cross-coupling,^18^ whereas the pinacol-derived ester **1c**, arylboronate complex **1d**,^19^ and trifluoroborate salt **1e** exhibited no reactivity.^20^ The enhanced reactivity of catechol-derived boronic esters compared to pinacol boronic esters is likely due to their increased Lewis acidity and lower oxidation potentials, which facilitate deboronative radical formation.^21^ Finally, control experiments confirmed the essential roles of catechol, base, nickel, and the photocatalyst (entries 7–10). The formation of only trace amounts of **3** under irradiation in the absence of photocatalyst suggests that an alternative electron donor–acceptor (EDA) complex pathway for alkyl radical formation is unlikely to be operative.^22^

**Table 1:**
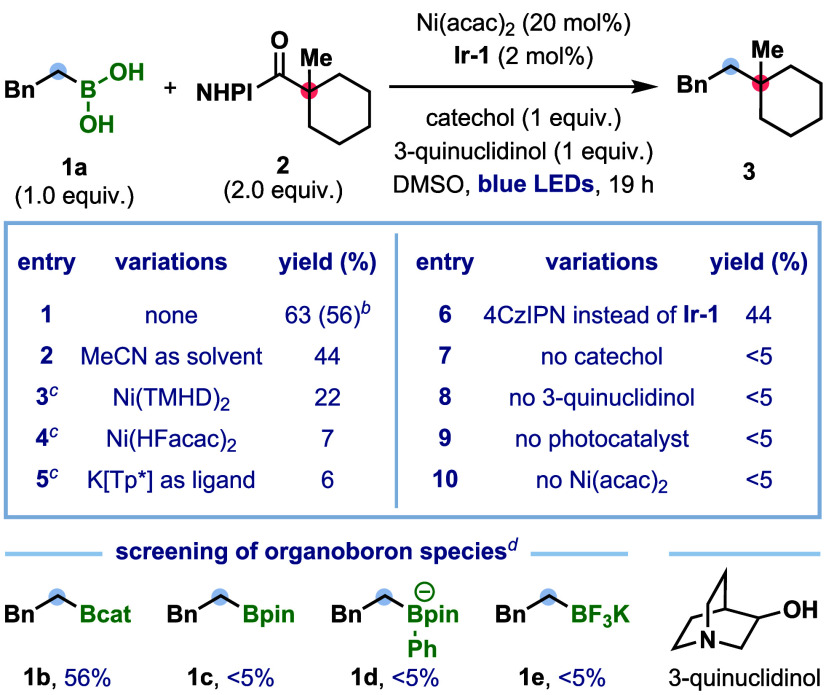
Reaction Optimization^a^

a Reactions were performed on 0.10 mmol scale, yield determined by GC-FID analysis using 1,3,5-trimethoxybenzene as an internal standard.

b Yield of isolated product for a 0.20 mmol scale reaction.

c With MeCN as the solvent.

d Reactions performed in the absence of catechol. **Ir-1** = [Ir­(*d*F­(Me)­ppy)_2_(*dt*bbpy)]­PF_6_, Ni­(TMHD)_2_ = nickel­(II) bis­(2,2,6,6-tetramethyl-3,5-heptanedionate), Ni­(HFacac)_2_ = bis­(hexa-fluoroacetylacetonato)-nickel­(II).

With optimized conditions in hand, we next explored the scope of this deboronative cross-coupling with respect to the carboxylic acid (Figure 2). The reaction was found to be applicable to NHPI esters derived from a wide range of tertiary carboxylic acids, providing access to quaternary carbon centers (**4**–**12**). Cyclic carboxylic acids, including cyclohexyl (**4**–**5**), cyclopropyl (**6**), and piperidinyl (**7**), delivered the corresponding products in good yields. Notably, successful coupling of an α-fluoro carboxylic acid (**8**) demonstrated that electrophilic radicals readily participate in the S_H_2 alkylation. Acyclic tertiary carboxylic acid derivatives were also compatible, with substrates containing alkene (**9**) or bromide (**11**) functionalities undergoing productive coupling. Furthermore, α-oxy NHPI esters engaged effectively in cross-coupling to provide sterically hindered ethers (**12**). An array of cyclic secondary carboxylic acids was also successfully coupled to primary alkyl boronic acids, producing tertiary alkyl products bearing cyclic ether (**13**, **16**), difluoromethyl (**14**), carbamate (**15**), and unprotected alcohol (**17**). Pleasingly, the use of α-amino acids (**18**–**23**) allowed α-amino tertiary and quaternary carbon centers to be constructed in good yields, indicating that highly nucleophilic radicals can also engage in the S_H_2 alkylation. Altogether, these results show that radical polarity exerts minimal influence on reaction performance.

**Figure 2:**
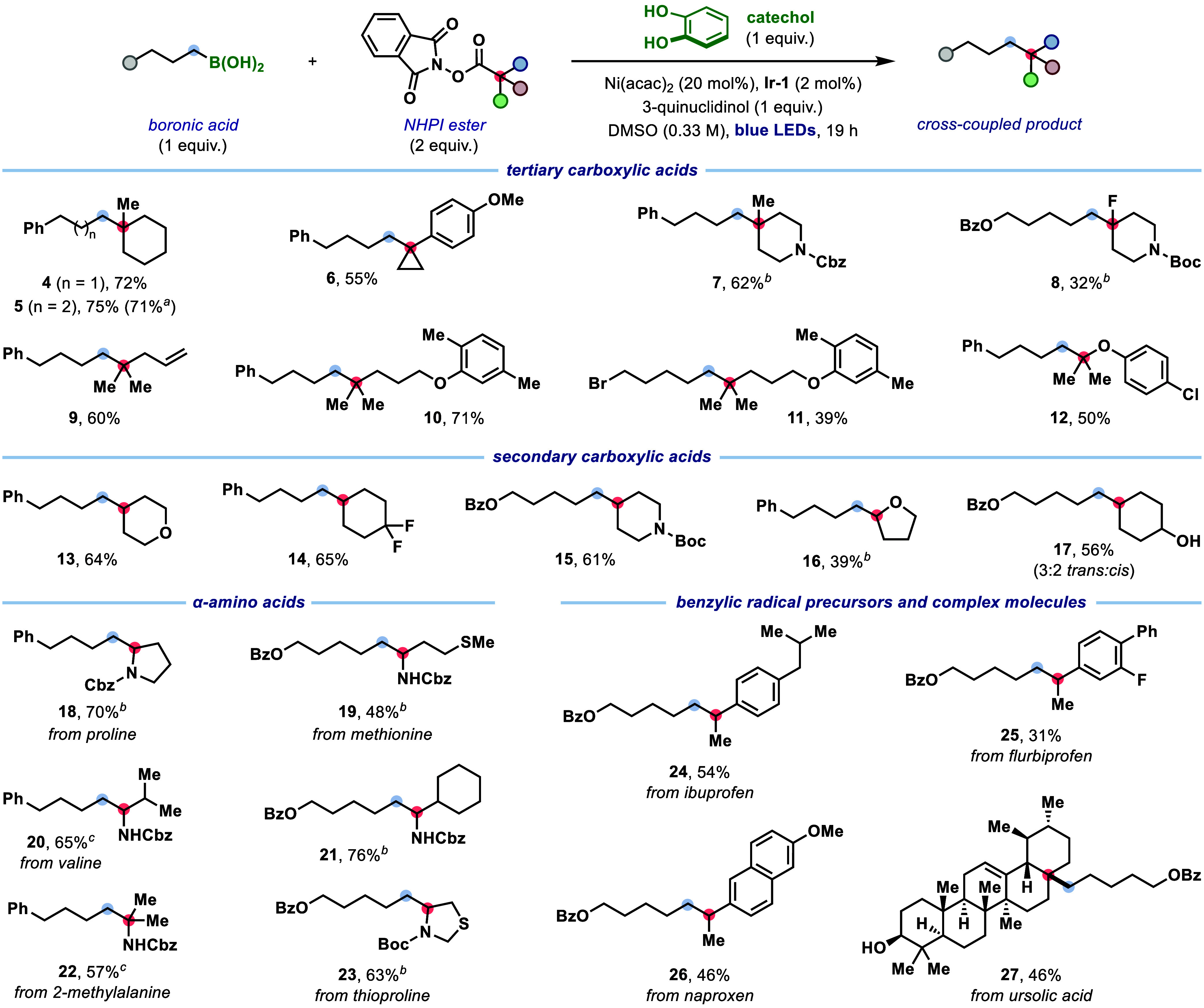
Scope of carboxylic acid derivatives. Reaction conditions: boronic acid (0.20 mmol), NHPI ester (2.0 equiv), **Ir-1** (2.0 mol %), Ni­(acac)_2_ (20 mol %), catechol (1 equiv), and 3-quinuclidinol (1.0 equiv) in DMSO (*c* = 0.33 M) with blue LED irradiation for 19 h at 35–40 °C. Yields are of isolated products after chromatographic purification. *^a^* Reaction performed on 1.0 mmol scale; see SI for details. *^b^* Using [Ir­(*p*F­(Me)­ppy)_2_-(4,4’-*dt*bbpy)]­PF_6_ (**Ir-2**, 2.0 mol %) as the photocatalyst in DMSO (*c* = 0.10 M). *^c^* Using **Ir-2** (2.0 mol %) and Ni­(acac)_2_ (40 mol %) in DMSO (*c* = 0.10 M).

To further highlight the utility of this decarboxylative hydroalkylation protocol for late-stage functionalization, we applied it to a range of pharmaceutical and natural product derivatives (Figure 2). Benzylic carboxylic acids derived from ibuprofen (**24**), flurbiprofen (**25**), and naproxen (**26**) underwent effective cross-coupling. These results are notable considering the lower reactivity of benzylic radicals and the propensity of secondary benzylic substrates to favor β-hydride elimination pathways. Additionally, the bioactive tertiary carboxylic acid ursolic acid was converted to the corresponding alkylated product **27** in 46% yield, demonstrating that the cross-coupling can forge highly sterically congested C­(sp^3^)–C­(sp^3^) bonds within fused polycyclic frameworks. Finally, the scalability of the reaction was demonstrated through the synthesis of product **5** in comparable yield on a 1.0 mmol scale (see Section 2.8 in the Supporting Information).

We next turned our attention to the direct *anti*-Markovnikov hydroalkylation of unactivated terminal olefins with tertiary NHPI esters through a hydroboration/deboronative cross-coupling sequence (Figure 3). To achieve this, a Rh-catalyzed hydroboration of the olefin with catecholborane (HBcat) was first performed to give a primary alkyl catechol boronic ester, which was then subjected to our deboronative S_H_2 cross-coupling. We were delighted to find that this protocol provided product **5** with only a moderate reduction in yield compared to that obtained with the isolated alkyl boronic acid. No purification of the boronic ester intermediate was required, although removal of the solvent after hydroboration was found to be beneficial, since using a mixed DMSO/CH_2_Cl_2_ solvent in the cross-coupling led to reduced yields (see Supporting Information, Table S10). Furthermore, the generation of catechol boronic esters in the hydroboration eliminated the need for the addition of catechol in the subsequent S_H_2 alkylation. The broad applicability of this platform was demonstrated through the successful hydroalkylation of a diverse set of functionalized terminal olefins with tertiary NHPI ester **2**. Substrates bearing ester (**28**), phthalimide (**29**), nitrile (**30**), ketone (**31**), and amide (**32**) substituents were readily transformed to the corresponding cross-coupled products. Importantly, various useful synthetic handles were tolerated, including aryl bromide (**33**), triflate (**34**) and pinacol boronic ester (**35**). These motifs are often susceptible to reaction with *in situ*–generated low-valent nickel species,^23^ demonstrating the broad functional-group compatibility of our protocol as well as indicating that the deboronative alkylation likely proceeds via the proposed S_H_2 mechanism (*vide infra*). Olefins containing heteroaromatics, such as furan (**36**), thiophene (**37**), and pyridine (**38**), also underwent coupling to furnish the hydroalkylation products in moderate yields. Pleasingly, the cross-coupling exhibited high selectivity for reaction of the *in situ*-generated catechol boronic ester over less Lewis acidic pinacol boronic esters (**39**). To further highlight the utility of this hydroalkylation reaction, it was applied to the late-stage installation of quaternary carbon centers onto derivatives of the antipsychotic drug aripiprazole (**40**) and the fungicide fludioxonil (**41**).

**Figure 3:**
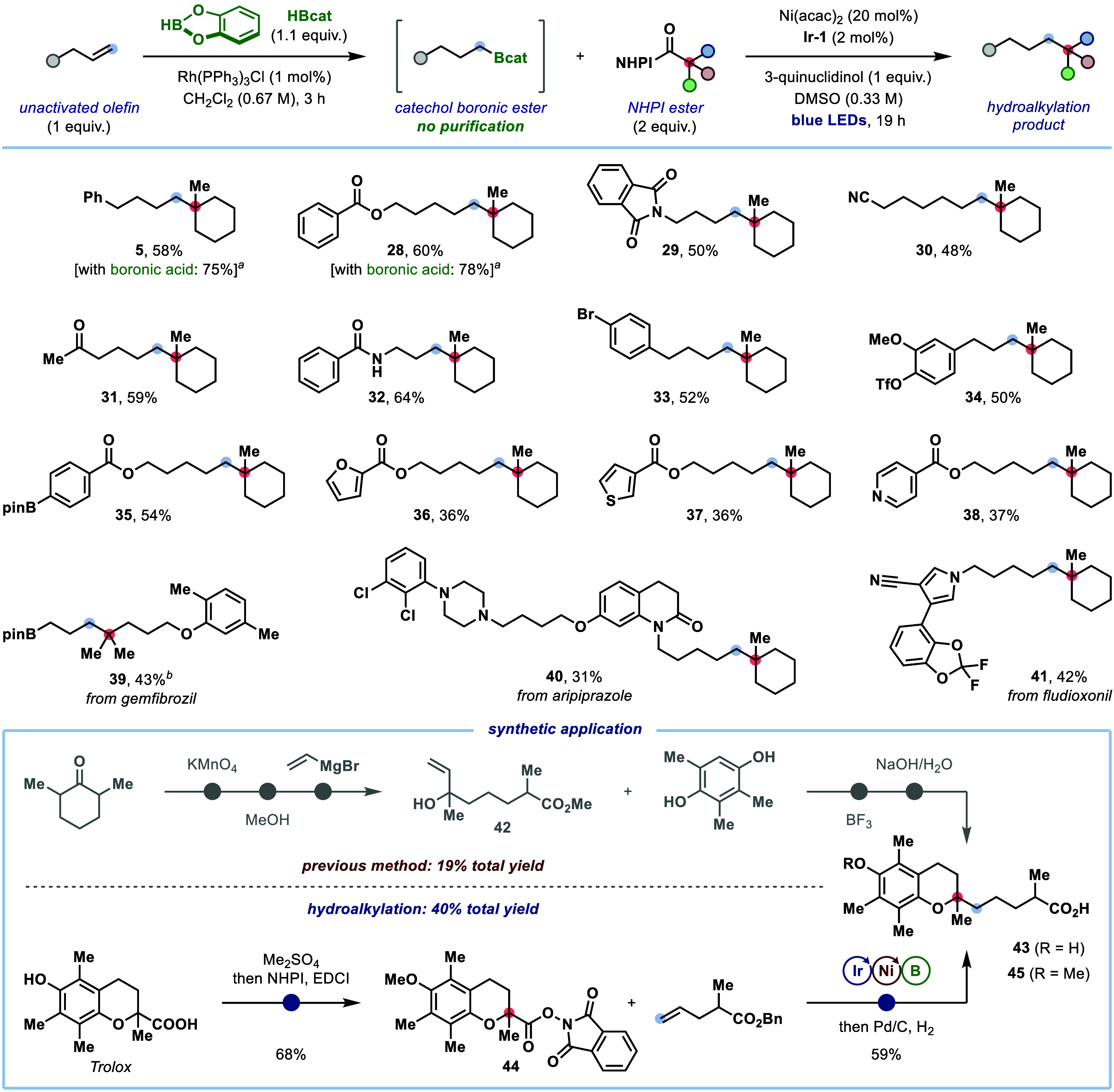
Scope of unactivated terminal alkenes. Reaction conditions: alkene (1.0 equiv), HBcat (1.1 equiv), Rh­(PPh_3_)_3_Cl (1.0 mol %) in DCM (*c* = 0.67 M), then NHPI ester (2.0 equiv), **Ir-1** (2.0 mol %), Ni­(acac)_2_ (20 mol %), and 3-quinuclidinol (1.0 equiv) in DMSO (*c* = 0.33 M) with blue LED irradiation for 19 h at 35–40 °C. Yields are of isolated products after chromatographic purification. *^a^* Using isolated boronic acids instead of alkenes under the conditions shown in Figure 2. *^b^* Yield of corresponding alcohol following boronic ester oxidation with H_2_O_2_/NaOH (see SI for details).

The ability of this *anti*-Markovnikov hydroalkylation protocol to streamline the synthesis of complex molecules was demonstrated through the synthesis of an analog of the vitamin E metabolite α-CMBHC (**43**). The previously reported route to **43** assembled the 2,2-dialkyl chromane core from allylic alcohol **42**, which required a 3-step synthesis from 2,6-dimethylcyclohexanone.^24^ In contrast, our hydroalkylation strategy enabled construction of the α-oxy quaternary center through decarboxylative cross-coupling of the inexpensive precursor Trolox, which provided a concise route to methyl α-CMBHC (**45**).^8c^ This approach not only presents a more straightforward and cost-efficient synthesis but also offers a versatile entry point to α-CMBHC analogues for structure–activity relationship studies.

A series of experiments were conducted to gain a deeper understanding of the mechanism of this nickel/photoredox dual catalysis process. For reactions of alkyl boronic acids (see Figure 2), we hypothesized that the role of catechol was to generate catechol boronic esters. These are more Lewis acidic than boronic acids and readily react with Lewis bases to form alkyl boronate complexes that are easily oxidized to trigger deboronative alkyl radical formation.^18b^ Support for the formation of a boronate complex between catechol boronic esters and 3-quinuclidinol was provided by ^11^B NMR studies (Figure 4a and Supporting Information, Figure S4). For a solution of boronic acid **46** in DMSO, a broad signal was observed at 31 ppm that remained unchanged in the presence of an equivalent of 3-quinuclidinol. However, the addition of catechol resulted in almost complete conversion of the boronic acid signal into a new signal at 9 ppm, which is characteristic of tetra-coordinate boronate complexes, such as catechol–quinuclidinol complex **47**. The important roles of catechol and the base in activating boronic acids for deboronative alkyl radical formation was further supported by luminescence quenching experiments, which showed that reductive quenching of the excited state of **Ir-1** was most efficient in the presence of complex **47**, formed from a mixture of **46**, 3-quinuclidinol, and catechol (Figure 4b). While 3-quinuclidinol and catechol both quenched the excited state of **Ir-1**, albeit less effectively than **47** (see Section 3.4 of the Supporting Information for details), no reduction in luminescence intensity was observed with NHPI ester **2**, thus ruling out an oxidative quenching cycle.^25^ The intermediacy of both primary and tertiary alkyl radical species was confirmed by performing the reaction of boronic acid **46** and NHPI ester **2** in the presence of (2,2,6,6-tetramethylpiperidin-1-yl)­oxyl (TEMPO) (Figure 4c). Although no cross-coupled product **5** was formed, the primary alkyl radical adduct **48** was isolated in 45% yield, and the tertiary alkyl radical adduct **49** was detected by GC-MS. In addition, a radical clock experiment with hexenyl boronic acid **50** confirmed the generation of primary alkyl radicals, since intramolecular 5*-exo*-trig cyclization occurred to give the cyclopentyl product **52** in a 7:1 ratio with the acyclic isomer **51**.

**Figure 4:**
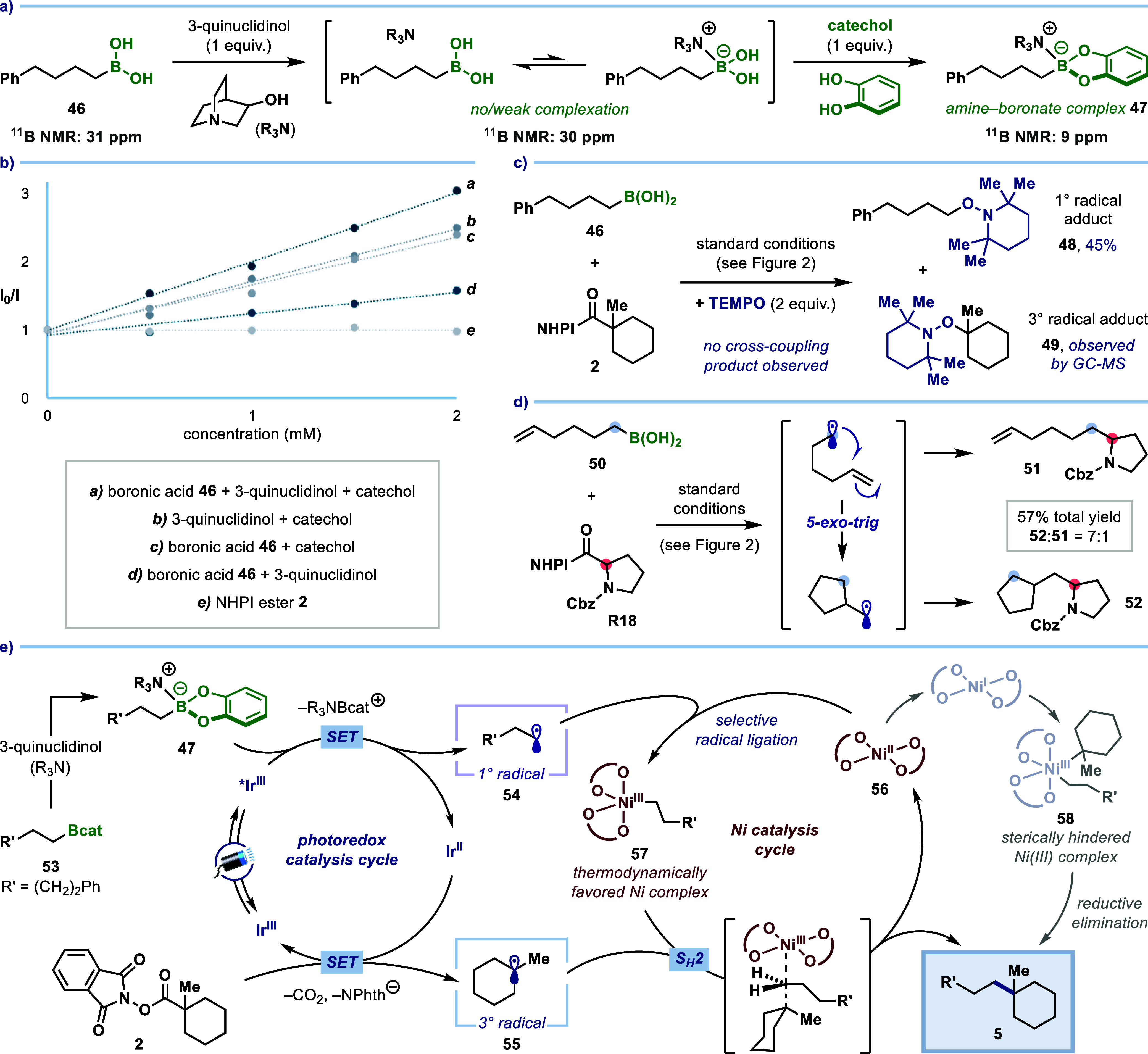
Mechanistic studies. (**a**) ^11^B NMR studies of amine–boron complexation. (**b**) Luminescence quenching (Stern–Volmer) analysis, indicating reductive quenching of the photocatalyst by boronate complex **47**. (**c**) TEMPO trapping evidences the formation of two different alkyl radicals. (**d**) Radical clock experiment: 5-*exo*-trig cyclization/coupling further indicates the radical pathway. (**e**) Proposed mechanism.

Based on these results, our proposed mechanism for this dual nickel/photoredox-catalyzed cross-coupling is presented in Figure 4e. Initial reaction of catechol boronic ester **53** (generated by olefin hydroboration or esterification of boronic acid **46** with catechol) with 3-quinuclidinol provides boronate complex **47**. Catechol boronate complexes have previously been reported to have low oxidation potentials (*E* ∼ 0.5 V vs SCE in MeCN),^18a^ thus enabling exergonic single-electron transfer (SET) between **47** and the excited state of photocatalyst **Ir-1** (*E*_1/2_ [*Ir^III^/Ir^II^] = 0.97 V vs SCE in MeCN),^26^ resulting in deboronation and formation of primary alkyl radical **54**. Single-electron reduction of NHPI ester **2** by the reduced photocatalyst ([Ir^III^/Ir^II^]= −1.43 V vs SCE in MeCN)^24a^ completes the photoredox cycle and furnishes tertiary alkyl radical **55** upon fragmentation and extrusion of CO_2_. At this stage, the primary alkyl radical **54** can be selectively captured by nickel­(II) catalyst **56** to form thermodynamically favored, Ni­(III)–alkyl complex **57**.^14b‑14c^ Finally, an S_H_2 reaction between tertiary radical **55** and **57** provides cross-coupled product **5** and regenerates Ni­(II) catalyst **56**. An alternative pathway involving oxidative addition to a Ni­(I) species and reductive elimination of dialkyl-Ni­(III) complex **58** was also considered. However, this was ruled out based on the negligible change in yield observed when the cross-coupling reaction was performed in the presence of an electron-deficient aryl bromide, which are known to rapidly react with low-valent nickel complexes (see Section 3.5 of the Supporting Information for details).^14b^

## Conclusions

In conclusion, we have established a boron-mediated *anti*-Markovnikov hydroalkylation of unactivated olefins using readily available carboxylic acid derivatives as alkylating reagents. The reaction is enabled by a hydroboration followed by a deboronative S_H_2-type radical coupling mechanism under nickel/photoredox dual catalysis. This protocol allows the efficient construction of densely substituted carbon centers from terminal alkenes, providing a novel platform for constructing quaternary carbon centers from readily available precursors. The mild reaction conditions and broad functional-group tolerance highlight its potential for the late-stage functionalization of complex organic molecules. By overcoming the long-standing challenge of olefin hydroalkylation with tertiary alkyl electrophiles, this approach offers a general and practical method for the rapid assembly of sterically congested sp^3^-rich structures.

## Supplementary Material



## Notes

Additional experimental details, including procedures, photographs of the experimental setup, characterization data, spectra, and computational details (PDF)
