# Supplementary material for: Boron-Mediated Hydroalkylation of Unactivated Olefins: An Anti-Markovnikov Approach to Congested Carbon Centers
Source: J Am Chem Soc. 2026 Mar 13;148(11):12227–34. doi: 10.1021/jacs.6c01091 (PMC13022885; doi:10.1021/jacs.6c01091)
Supplement: Supplementary file 1 [file ja6c01091_si_001.pdf]

SUPPLEMENTARY INFORMATION

Boron-Mediated Hydroalkylation of Unactivated Olefins: An *anti*-Markovnikov Approach to Congested Carbon Centers

Hanwen Zhang†, Ruocheng Sang†, Gianluca Simionato†, Jasper L. Tyler, Adam Noble* and Varinder K. Aggarwal*

School of Chemistry, University of Bristol, Cantock's Close, Bristol BS8 1TS, United Kingdom

*e-mail: a.noble@bristol.ac.uk; v.aggarwal@bristol.ac.uk

TABLE OF CONTENTS

LIST OF SUPPLEMENTARY SCHEMES, FIGURES AND TABLES 3

LIST OF CHARACTERISED PRODUCTS 4

1. MATERIALS AND GENERAL METHODS 6

1.1. Glassware, Solvents and Reagents 6

1.2. Chromatography and Instrumentation 6

1.3. Naming of Compounds 6

2. EXPERIMENTAL DATA 7

2.1. General Procedures 7

2.1.1. General Procedure A: Cross-coupling Reaction between Boronic Acid and Redox-active Ester .7

2.1.2. General Procedure B: Hydroalkylation of Unactivated Terminal Alkene 8

2.2. Photochemical Equipment and Setup 9

2.3. Reaction Optimization 10

2.3.1. Standard procedure for reaction optimization 10

2.3.2. Organoboron species screening 10

2.3.3. Base screening 11

2.3.4. Solvent screening 11

2.3.5. Photocatalyst screening 12

2.3.6. Metal catalyst screening 14

2.3.7. Stoichiometry of each component screening 15

2.3.8. Additional base screening 15

2.3.9. Control experiments 16

2.3.10. Reaction optimization with (4-phenylbutyl)boronic acid 17

2.3.11. Reaction optimization with *α*-*N*-carboxylic redox-active ester 17

2.3.12. Reaction optimization with alkene as reactant 18

2.3.13. Comparison with reported nickel-catalyzed reductive olefin hydroalkylation protocol 19

2.4. Synthesis of redox-active esters 20

2.4.1. Successful redox-active ester coupling partners 20

2.4.2. Synthesis of unreported redox-active esters 21

2.5. Synthesis of terminal alkenes 23

2.5.1. Successful terminal alkene coupling partners 23

2.5.2. Synthesis of unreported terminal alkenes 24

2.6. Substrate Scope 28

2.7. Scale-up Reaction 53

2.8. Synthesis Application 54

2.9. Unsuccessful Substrates 56

3. MECHANISTIC STUDIES 57

3.1. ^{11}B NMR experiment 57

3.2. Radical trap experiment....58
3.3. Radical clock experiment 60
3.4. Stern-Volmer experiments....62
3.5. Probing intermediacy of low-valent Ni species....68

4. NMR SPECTROSCOPIC DATA 69

5. REFERENCES 128

LIST OF SUPPLEMENTARY SCHEMES, FIGURES AND TABLES

Table S1. Boronic compound screening 10
Table S2. Base screening 11
Table S3. Solvent screening 11
Table S4. Photocatalyst screening 12
Table S5. Metal catalyst screening 14
Table S6. Stoichiometry of each component screening 15
Table S7. Additional base screening 15
Table S8. Control experiments 16
Table S9. Reaction optimization with (4-phenylbutyl)boronic acid 17
Table S10. Reaction optimization with α-*N*-carboxylic redox-active ester 17
Table S11. Reaction optimization with alkene as reactant 18
Figure S1. Successful redox-active ester coupling partners. 20
Figure S2. Successful terminal alkene coupling partners 23
Figure S3. Unsuccessful substrates 56
Figure S4. ^{11}B NMR spectra of different combinations with (4-phenylbutyl)boronic acid 46 58
Figure S5. Fluorescence emission quenching of [Ir(*d*F(Me)ppy)$_2$(*d*tbbpy)]PF$_6$ (10μM) in DMSO using (4-phenylbutyl)boronic acid as quencher (left) and relative Stern-Volmer plot (right) of fluorescence intensity ratio I$_0$/I as a function of quencher concentration. 63
Figure S6. Fluorescence emission quenching of [Ir(*d*F(Me)ppy)$_2$(*d*tbbpy)]PF$_6$ (10μM) in DMSO using 3-quinuclidinol as quencher (left) and relative Stern-Volmer plot (right) of fluorescence intensity ratio I$_0$/I as a function of quencher concentration. 63
Figure S7. Fluorescence emission quenching of [Ir(*d*F(Me)ppy)$_2$(*d*tbbpy)]PF$_6$ (10μM) in DMSO using catechol as quencher (left) and relative Stern-Volmer plot (right) of fluorescence intensity ratio I$_0$/I as a function of quencher concentration. 64
Figure S8. Fluorescence emission quenching of [Ir(*d*F(Me)ppy)$_2$(*d*tbbpy)]PF$_6$ (10μM) in DMSO using a 1:1 mixture of (4-phenylbutyl)boronic acid and 3-quinuclidinol as quencher (left) and relative Stern-Volmer plot (right) of fluorescence intensity ratio I$_0$/I as a function of quencher concentration. 64
Figure S9. Fluorescence emission quenching of [Ir(*d*F(Me)ppy)$_2$(*d*tbbpy)]PF$_6$ (10μM) in DMSO using a 1:1 mixture of (4 -phenylbutyl)boronic acid and catechol as quencher (left) and relative Stern-Volmer plot (right) of fluorescence intensity ratio I$_0$/I as a function of quencher concentration. 65
Figure S10. Fluorescence emission quenching of [Ir(*d*F(Me)ppy)$_2$(*d*tbbpy)]PF$_6$ (10μM) in DMSO using a 1:1:1 mixture of (4 -phenylbutyl)boronic acid, 3-quinuclidinol and catechol as quencher (left) and relative Stern-Volmer plot (right) of fluorescence intensity ratio I$_0$/I as a function of quencher concentration. 65
Figure S11. Fluorescence emission quenching of [Ir(*d*F(Me)ppy)$_2$(*d*tbbpy)]PF$_6$ (10μM) in DMSO using a 1:1 mixture of 3-quinuclidinol and catechol as quencher (left) and relative Stern-Volmer plot (right) of fluorescence intensity ratio I$_0$/I as a function of quencher concentration. 66
Figure S12. Fluorescence emission quenching of [Ir(*d*F(Me)ppy)$_2$(*d*tbbpy)]PF$_6$ (10μM) in DMSO using NHPI redox-active ester as quencher (left) and relative Stern-Volmer plot (right) of fluorescence intensity ratio I$_0$/I as a function of quencher concentration. 66
Figure S13. Comparison of the linear fittings of all the Stern-Volmer plots. 67

LIST OF CHARACTERISED PRODUCTS

1,3-Dioxoisoindolin-2-yl ((benzyloxy)carbonyl)-*L*-valinate (**R20**) 21
1,3-Dioxoisoindolin-2-yl (1*S*,2*R*,4a*S*,6a*S*,6b*R*,8a*R*,10*S*,12a*R*,12b*R*,14b*S*)-10-hydroxy-1,2,6a,6b,9,9,12a-heptamethyl-1,3,4,5,6,6a,6b,7,8,8a,9,10,11,12,12a,12b,13,14b-octadecahydropicene-4a(2*H*)-carboxylate (**R27**) 21
Pent-4-en-1-yl 4-(4,4,5,5-tetramethyl-1,3,2-dioxaborolan-2-yl)benzoate (**35a**) 24
Pent-4-en-1-yl thiophene-3-carboxylate (**37a**) 24
Pent-4-en-1-yl isonicotinate (**38a**) 25
7-(4-(4-(2,3-Dichlorophenyl)piperazin-1-yl)butoxy)-1-(pent-4-en-1-yl)-3,4-dihydroquinolin-2(1H)-one (**40a**) ... 26
4-(2,2-Difluorobenzo[*d*][1,3]dioxol-4-yl)-1-(pent-4-en-1-yl)-1*H*-pyrrole-3-carbonitrile (**41a**) 26
(2-(1-Methylcyclohexyl)ethyl)benzene (**3**) 28
(3-(1-Methylcyclohexyl)propyl)benzene (**4**) 28
(4-(1-Methylcyclohexyl)butyl)benzene (**5**) 29
1-Methoxy-4-(1-(4-phenylbutyl)cyclopropyl)benzene (**6**) 29
Benzyl 4-methyl-4-(4-phenylbutyl)piperidine-1-carboxylate (**7**) 30
tert-Butyl 4-(5-(benzoyloxy)pentyl)-4-fluoropiperidine-1-carboxylate (**8**) 31
(5,5-Dimethyloct-7-en-1-yl)benzene (**9**) 31
2-((4,4-Dimethyl-8-phenyloctyl)oxy)-1,4-dimethylbenzene (**10**) 32
2-((9-Bromo-4,4-dimethylnonyl)oxy)-1,4-dimethylbenzene (**11**) 32
1-Chloro-4-((2-methyl-6-phenylhexan-2-yl)oxy)benzene (**12**) 33
4-(4-Phenylbutyl)tetrahydro-2*H*-pyran (**13**) 34
(4-(4,4-Difluorocyclohexyl)butyl)benzene (**14**) 34
tert-Butyl 4-(5-(benzoyloxy)pentyl)piperidine-1-carboxylate (**15**) 35
2-(4-Phenylbutyl)tetrahydrofuran (**16**) 35
(*Cis* and *trans*) 5-(4-hydroxycyclohexyl)pentyl benzoate (**17**) 36
Benzyl-2-(4-phenylbutyl)pyrrolidine-1-carboxylate (**18**) 37
Benzyl-(1-(methylthio)-7-phenylheptan-3-yl)carbamate (**19**) 37
Benzyl-(2-methyl-7-phenylheptan-3-yl)carbamate (**20**) 38
tert-Butyl-(1-cyclohexyl-5-phenylpentyl)carbamate (**21**) 38
Benzyl (2-methyl-6-phenylhexan-2-yl)carbamate (**22**) 39
tert-Butyl-4-(5-(benzoyloxy)pentyl)thiazolidine-3-carboxylate (**23**) 40
6-(4-Isobutylphenyl)heptyl benzoate (**24**) 40
6-(2-Fluoro-[1,1'-biphenyl]-4-yl)heptyl benzoate (**25**) 41
6-(6-Methoxynaphthalen-2-yl)heptyl benzoate (**26**) 42
5-((1*S*,2*R*,6a*S*,6b*R*,8a*R*,10*S*,12a*R*,12b*R*,14b*R*)-10-hydroxy-1,2,6a,6b,9,9,12a-heptamethyl-1,3,4,5,6,6a,6b,7,8,8a,9,10,11,12,12a,12b,13,14b-octadecahydropicen-4a(2*H*)-yl)pentyl benzoate (**27**) 42
5-(1-Methylcyclohexyl)pentyl benzoate (**28**) 43
2-(4-(1-Methylcyclohexyl)butyl)isoindoline-1,3-dione (**29**) 44
7-(1-Methylcyclohexyl)heptanenitrile (**30**) 44
6-(1-Methylcyclohexyl)hexan-2-one (**31**) 45
N-(3-(1-methylcyclohexyl)propyl)benzamide (**32**) 45
1-Bromo-4-(4-(1-methylcyclohexyl)butyl)benzene (**33**) 46
2-Methoxy-4-(3-(1-methylcyclohexyl)propyl)phenyl trifluoromethanesulfonate (**34**) 47
5-(1-Methylcyclohexyl)pentyl 4-(4,4,5,5-tetramethyl-1,3,2-dioxaborolan-2-yl)benzoate (**35**) 47
5-(1-Methylcyclohexyl)pentyl furan-2-carboxylate (**36**) 48
5-(1-Methylcyclohexyl)pentyl thiophene-3-carboxylate (**37**) 49
5-(1-Methylcyclohexyl)pentyl isonicotinate (**38**) 49

7-(2,5-Dimethylphenoxy)-4,4-dimethylheptan-1-ol (**39**)........ 50
7-(4-(4-(2,3-Dichlorophenyl)piperazin-1-yl)butoxy)-1-(5-(1-methylcyclohexyl)pentyl)-3,4-dihydroquinolin-2(1*H*)-one (**40**) 51
4-(2,2-Difluorobenzo[*d*][1,3]dioxol-4-yl)-1-(5-(1-methylcyclohexyl)pentyl)-1*H*-pyrrole-3-carbonitrile (**41**)........ 52
Synthesis of **AP-1** 54
Synthesis of 1,3-dioxoisoindolin-2-yl 6-methoxy-2,5,7,8-tetramethylchromane-2-carboxylate (**44**) 54
Synthesis of **45S**........ 55
Synthesis of 5-(6-methoxy-2,5,7,8-tetramethylchroman-2-yl)-2-methylpentanoic acid (**45**) 55

1. MATERIALS AND GENERAL METHODS

1.1. Glassware, Solvents and Reagents

All anhydrous solvents were commercially supplied or dried using an Anhydrous Engineering alumina column drying system [THF, toluene, Et_2O, DCM (dichloromethane)]. Reagents were purchased from commercial sources and used as received. DMSO was anhydrous, purchased from ACROS and used as received.

1.2. Chromatography and Instrumentation

Thin layer chromatography (TLC) was performed using Merck Kieselgel 60 F254 fluorescent treated silica, which was visualised under UV light, or by staining with aqueous basic potassium permanganate followed by heating, *p*-anisaldehyde solution followed by heating, Hanessian's stain (CAM stain) followed by heating, or an ethanolic solution of phosphomolybdic acid followed by heating, as stated.

Flash column chromatography (FCC) was carried out using Sigma-Aldrich silica gel (60 Å, 230–400 mesh, 40–63 µm) or a Biotage Isolera^{TM} flash purification system. In cases where automated column chromatography was employed the solvent gradient and flow rate are indicated.

NMR spectra were recorded at various field strengths, as indicated, using Bruker 400 MHz, Varian VNMR 400 MHz, Varian VNMR 500 MHz, or Bruker Cryo 500 MHz for ^{1}H, ^{11}B, ^{13}C and ^{19}F acquisitions. All NMR spectra were recorded at 25 ℃ unless otherwise stated. Chemical shifts (δ) are reported in parts per million (ppm) and referenced $CDCl_3$ (^{1}H: 7.26 ppm; ^{13}C: 77.16 ppm). Coupling constants (*J*) are given in Hertz (Hz) and refer to apparent multiplicities (s = singlet, d = doublet, t = triplet, q = quartet, quin = quintet, sex = hextet, h = heptet, m = multiplet, br = broad signal, dd = doublet of doublets, *etc.*). The ^{1}H NMR spectra are reported as follows: chemical shift (multiplicity, coupling constants, number of protons).

High resolution mass spectra (HRMS) were recorded on a Bruker Daltonics MicrOTOF II by Electrospray Ionisation (ESI); a Thermo Scientific QExactive by Electron Ionisation (EI); a Thermo Scientific Orbitrap Elite by ESI or Atmospheric Pressure Chemical Ionisation (APCI); or a Bruker UltrafleXtreme by Matrix-assisted Laser Desorption/Ionisation (MALDI).

IR spectra were recorded neat as a thin film on a Perkin Elmer Spectrum One FT-IR. Selected absorption maxima (ν_{max}) are reported in wavenumbers (cm^{-1}).

Steady-state emission spectroscopy studies were performed at room temperature on a FluoroMax$^{@}$ spectrophotometer.

1.3. Naming of Compounds

Compound names are those generated by ChemDraw Professional 20.0 software (PerkinElmer), following the IUPAC nomenclature.

2. EXPERIMENTAL DATA

2.1. General Procedures

2.1.1. General Procedure A: Cross-coupling Reaction between Boronic Acid and Redox-active Ester

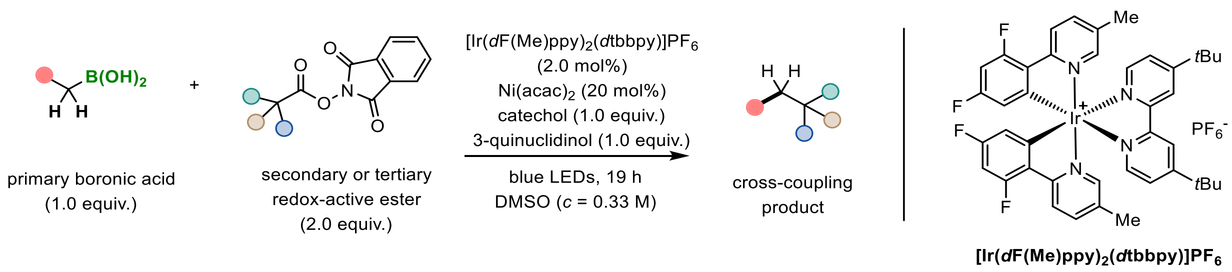

Under an ambient atmosphere, a flame dried 8 mL Biotage® microwave reaction vial equipped with a magnetic stir bar was transferred into an anhydrous, argon-filled glovebox and charged with [Ir(*d*F(Me)ppy)$_2$(*d*tbbpy)]PF_6 (4.0 mg, 4.0 μmol, 2.0 mol%) or [Ir(*p*-F(Me)ppy)$_2$-(4,4'-*d*tbbpy)]PF_6 (4.0 mg, 4.0 μmol, 2.0 mol%), Ni(acac)$_2$ (10 mg, 0.040 mmol, 20 mol%), boronic acid (0.20 mmol, 1.0 equiv.), redox-active ester (0.40 mmol, 2.0 equiv.), catechol (22 mg, 0.20 mmol, 1.0 equiv), 3-quinuclidinol (25 mg, 0.20 mmol, 1.0 equiv.) sequentially, followed by the anhydrous DMSO (0.60 mL, c = 0.33 M) or (2.0 mL, c = 1.0 M). The vial was sealed with a cap with septum, removed from the glovebox. The mixture was pre-stirred for 10 min without irradiation until a homogeneous solution formed and subsequently positioned 3 cm from a blue LED light source (Kessil Tuna Blue LEDs). After stirring at 1200 rpm under irradiation for 19 h with fan cooling, the reaction mixture was diluted with wet EtOAc (1 mL), washed with deionized water (5 mL) and brine (5 mL), and extracted with EtOAc (3 × 10 mL). The combined organic layers were washed with brine (10 mL), dried over $MgSO_4$, filtered, and concentrated *in vacuo*. The residue was purified by flash column chromatography or Biotage Isolera® flash purification on silica gel.

2.1.2. General Procedure B: Hydroalkylation of Unactivated Terminal Alkene

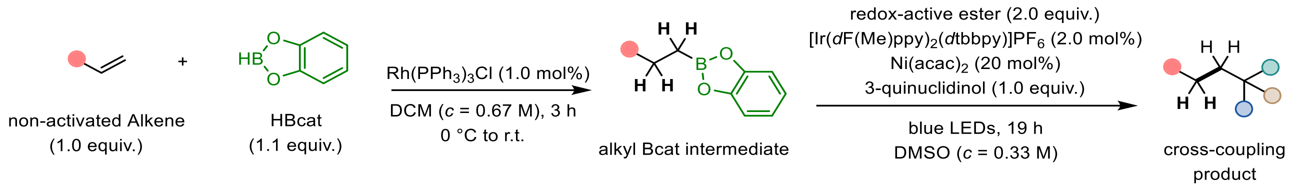

Under an ambient atmosphere, a flame dried 8 mL Biotage® microwave reaction vial equipped with a magnetic stir bar was transferred into an anhydrous, argon-filled glovebox and charged with alkene (0.20 mmol, 1.0 equiv.), $Rh(PPh_3)_3Cl$ (2.0 mg, 2.0 μmol, 1.0 mol%) and anhydrous DCM (0.10 mL, c = 2.0 M), then cooled to 0 ℃, HBcat (26.4 mg, 0.220 mmol, 1.10 equiv.) was added. The vial was sealed with a cap with septum, removed from the glovebox and stirred at room temperature for 3 h. After removal of the solvent *in vacuo*, the vial was backfilled with N_2 and transferred back into the glovebox. $[Ir(dF(Me)ppy)_2(dtbbpy)]PF_6$ (4.0 mg, 4.0 μmol, 2.0 mol%), $Ni(acac)_2$ (10 mg, 0.040 mmol, 20 mol%), redox-active ester (0.40 mmol, 2.0 equiv.) and 3-quinuclidinol (25 mg, 0.20 mmol, 1.0 equiv.) were added sequentially, followed by the anhydrous DMSO (0.60 mL, c = 0.33 M). The vial was sealed with a cap with septum, removed from the glovebox. The mixture was pre-stirred for 10 min without irradiation until a homogeneous solution formed and subsequently positioned 3 cm from a blue LED light source (Kessil Tuna Blue LEDs). After stirring at 1200 rpm under irradiation for 19 h with fan cooling, the reaction mixture was diluted with wet EtOAc (1 mL), washed with deionized water (5 mL) and brine (5 mL), and extracted with EtOAc (3 × 10 mL). The combined organic layers were washed with brine (10 mL), dried over $MgSO_4$, filtered, and concentrated *in vacuo*. The residue was purified by flash column chromatography or Biotage Isolera® flash purification on silica gel.

2.2. Photochemical Equipment and Setup

A PR160L-tuna blue Kessil LED lamps was used with the intensity dial set to 100. The reaction vials were positioned approximately 3 cm from an LED lamp (see image below). The temperatures during irradiation were typically approximately 35–40 ℃.

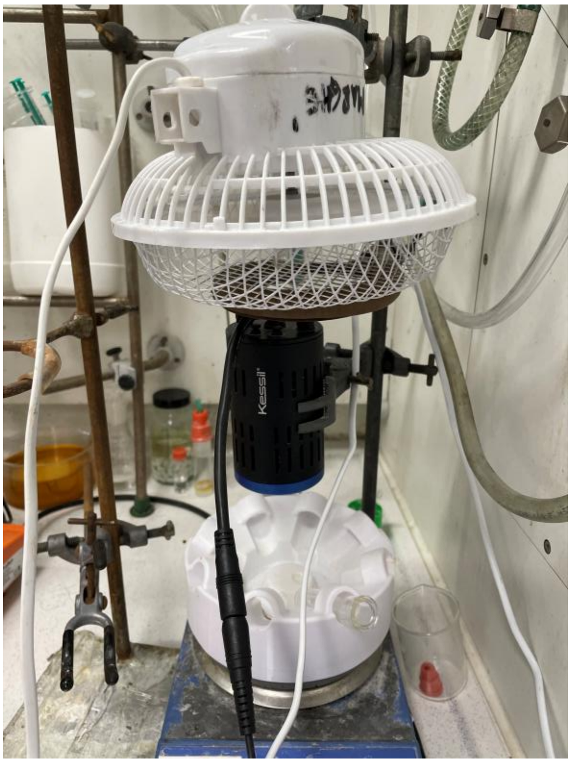

2.3. Reaction Optimization

2.3.1. Standard procedure for reaction optimization

Under an ambient atmosphere, a flame dried 8 mL Biotage® microwave reaction vial equipped with a magnetic stir bar was transferred into an anhydrous, argon-filled glovebox and charged with photocatalyst, metal catalyst, boronic acid (0.10 mmol, 1.0 equiv.), redox-active ester (0.20 mmol, 2.0 equiv.), catechol (11 mg, 0.10 mmol, 1.0 equiv), Lewis basic additive (0.10 mmol, 1.0 equiv.) sequentially, followed by the anhydrous solvent. The vial was sealed with a cap with septum, removed from the glovebox. The mixture was pre-stirred for 10 min without irradiation until a homogeneous solution formed and subsequently positioned 3 cm from a blue LED light source (Kessil Tuna Blue LEDs). After stirring at 1200 rpm under irradiation for 19 h with fan cooling, the reaction mixture was diluted with wet EtOAc (1 mL) and 1,3,5-trimethoxybenzene (17 mg, 0.10 mmol, 1.0 equiv.) was added as internal standard. The solution was washed with deionized water (5 mL), extracted with EtOAc (3 × 5 mL) and dried by anhydrous $MgSO_4$, concentrated *in vacuo* and the yield was determined by GC-FID analysis.

2.3.2. Organoboron species screening

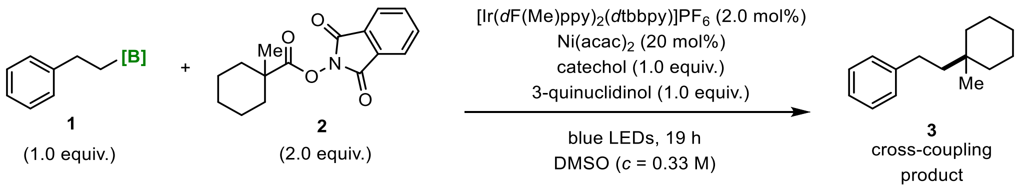

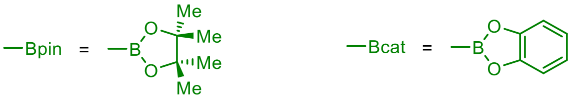

Entry	Boronic reagent	Changes from above	GC-FID yield[a]
**1**	**-B(OH)$_2$**	**-**	**63% (56%)[b]**
2	-Bcat	no catechol	56%
3[c]	-Bpin	no catechol	< 5%
4[c]	-Bpin	PhLi (1.1 eq)	< 5%
5[c]	-BF$_3$K	no catechol	< 5%

Table S1. Boronic compound screening

[a] Reactions were set up at 0.1 mmol scale, yield determined by GC-FID analysis using 1,3,5-trimethoxybenzene (17 mg, 0.10 mmol, 1.0 equiv.) as an internal standard. [b] Reactions were set up at 0.2 mmol scale, Isolated yield. [c] MeCN (0.1 M) as solvent.

2.3.3. Base screening

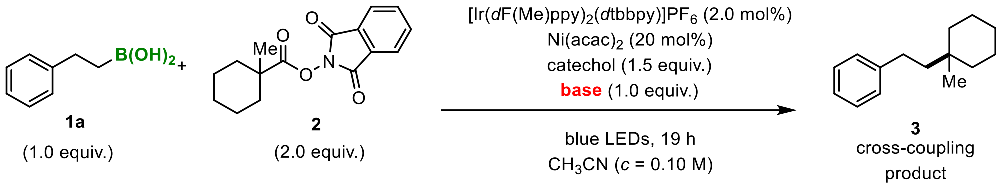

Entry	Base	GC-FID yield[a]
**1**	**3-quinuclidinol**	**26%**
2	Et_3N	23%
3	DIPEA	22%
4	DABCO	18%
5	pyridine	< 5%
6	PPh_3	6%

Table S2. Base screening

[a] Reactions were set up at 0.1 mmol scale, yield determined by GC-FID analysis using 1,3,5-trimethoxybenzene (17 mg, 0.10 mmol, 1.0 equiv.) as an internal standard.

2.3.4. Solvent screening

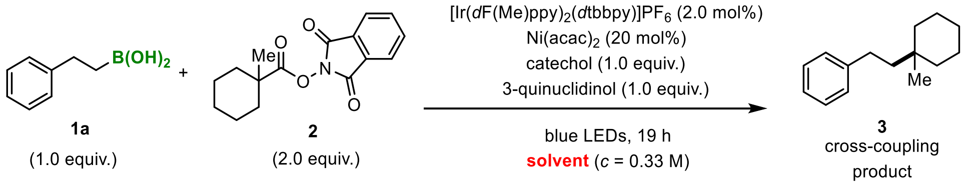

Entry	Solvent	GC-FID yield[a]
**1**	**DMSO**	**63%**
2	CH_3CN	44%
3	DMA	28%
4	1,4-dioxane	36%
5	THF	29%
6	MTBE	9%
7[b]	DCM	7%
8[b]	EtOAc	13%

Table S3. Solvent screening

[a] Reactions were set up at 0.1 mmol scale, yield determined by GC-FID analysis using 1,3,5-trimethoxybenzene (17 mg, 0.10 mmol, 1.0 equiv.) as an internal standard. [b] DIPEA (1.0 eq.) as Lewis basic additive.

2.3.5. Photocatalyst screening

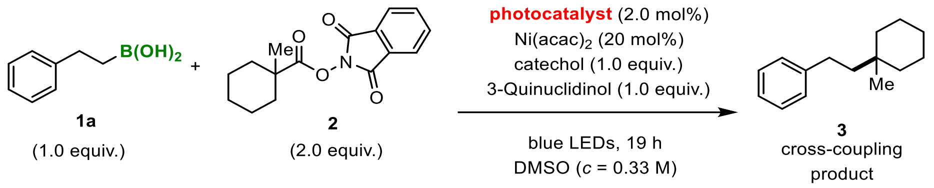

Entry	Photocatalyst	GC-FID yield[a]
**1**	**[Ir(*d*F(Me)ppy)$_2$(*d*tbbpy)]PF$_6$**	**63%**
2	[Ir(*p*-F(Me)ppy)$_2$-(4,4'-*d*tbbpy)]PF$_6$	58%
3	(Ir[*d*F(CF$_3$)ppy]$_2$(*d*tbpy))PF$_6$	50%
4	[Ir[*d*F(CF$_3$)$_2$ppy]$_2$(bpy)]PF$_6$	54%
5	[Ir(*d*FCF$_3$ppy)$_2$-(5,5'-*d*CF$_3$bpy)]PF$_6$	37%
6[b]	4CzIPN	44%
7[b]	3DPAFIPN	50%
8[b]	3CzClIPN	45%
9[b]	3DPA2FBN	44%

Table S4. Photocatalyst screening

[a] Reactions were set up at 0.1 mmol scale, yield determined by GC-FID analysis using 1,3,5-trimethoxybenzene (17 mg, 0.10 mmol, 1.0 equiv.) as an internal standard. [b] 5.0 mol% photocatalyst.

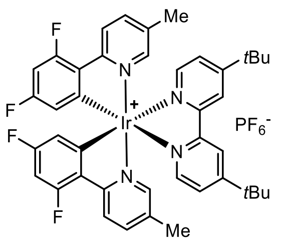

[Ir(*d*F(Me)ppy)$_2$(*d*tbbpy)]PF$_6$
CAS: 1335047-34-1

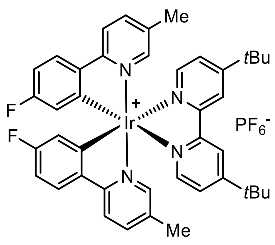

[Ir(*p*-F(Me)ppy)$_2$-(4,4′-*d*tbbpy)]PF$_6$
CAS: 808142-88-3

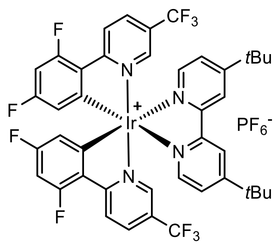

(Ir[*d*F(CF$_3$)ppy]$_2$(*d*tbpy))PF$_6$
CAS: 870987-63-6

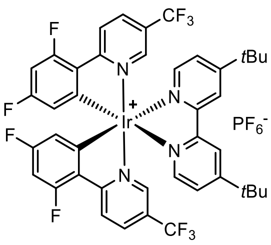

[Ir[*d*F(CF$_3$)$_2$ppy]$_2$(bpy)]PF$_6$
CAS: 1092775-62-6

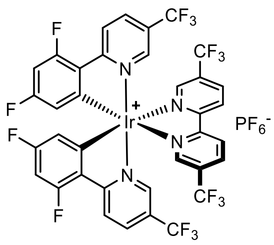

[Ir(*d*FCF$_3$ppy)$_2$-(5,5’-*d*CF$_3$bpy)]PF$_6$
CAS: 1973375-72-2

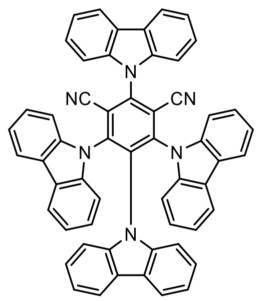

4CzIPN (5 mol%)
CAS: 1416881-52-1

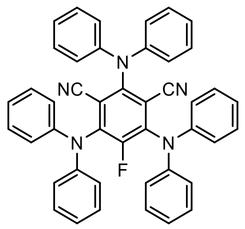

3DPAFIPN (5 mol%)
CAS: 2260543-73-3

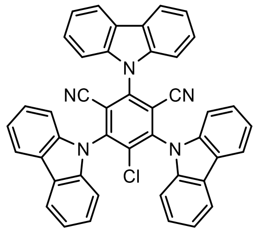

3CzClIPN (5 mol%)
CAS: 1469704-61-7

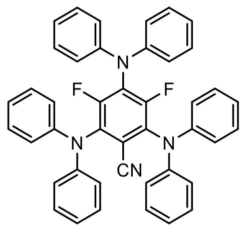

3DPA2FBN (5 mol%)
CAS: 1403850-00-9

2.3.6. Metal catalyst screening

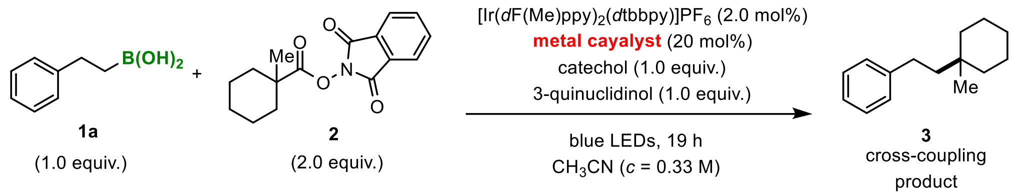

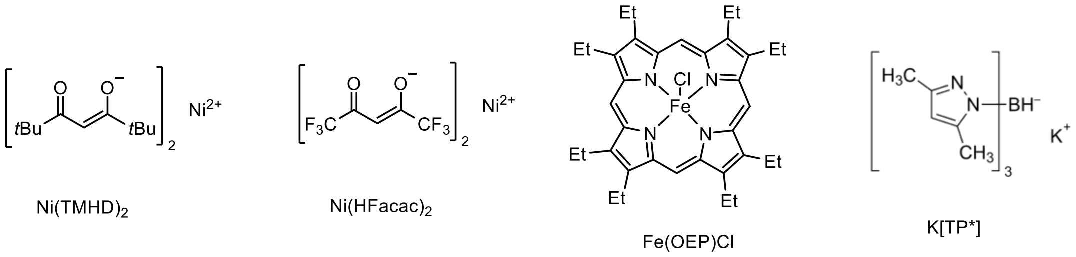

Entry	Metal catalyst	GC-FID yield[a]
**1**	**Ni(acac)2**	**44%**
2[b]	Ni(TMHD)2	22%
3	Ni(HFacac)2	7%
4	Fe(OEP)Cl (2 mol%)	n.d.
5	Ni(acac)2 (10 mol%) + K[TP*] (10 mol%)	6%

Table S5. Metal catalyst screening

[a] Reactions were set up at 0.1 mmol scale, yield determined by GC-FID analysis using 1,3,5-trimethoxybenzene (17 mg, 0.10 mmol, 1.0 equiv.) as an internal standard. [b] 1,4-Dioxane (0.10 M) as solvent.

2.3.7. Stoichiometry of each component screening

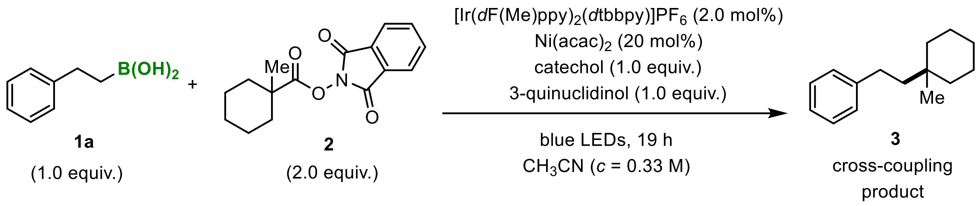

Entry	Changes from above	GC-FID yield[a]
**1**	**1.0 eq. catechol, DMSO as solvent**	**63%**
2	-	44%
3	2.0 eq. boronic acid, 1.0 eq. redox-active ester **2**, 2.0 eq. catechol	40%
4	2.5 eq. redox active ester **2**	44%
5	0.5 eq. 3-quinuclidinol	15%
6	2.0 eq. 3-quinuclidinol	30%

Table S6. Stoichiometry of each component screening

[a] Reactions were set up at 0.1 mmol scale, yield determined by GC-FID analysis using 1,3,5-trimethoxybenzene (17 mg, 0.10 mmol, 1.0 equiv.) as an internal standard.

2.3.8. Additional base screening

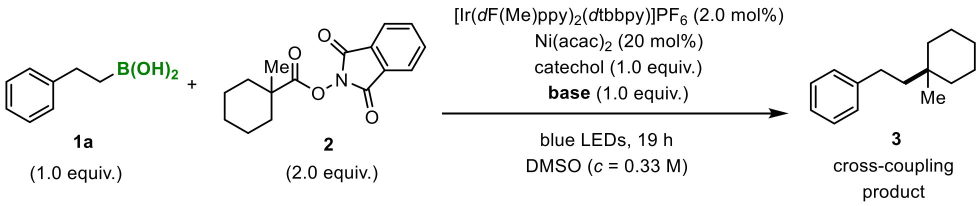

Entry	Base	GC-FID yield[a]
**1**	**3-quinuclidinol**	**63%**
2	quinuclidine	41%
3	DBU	40%
4	DIPEA (*i*-Pr_2NEt)	41%
5	NaOH	36%
6	pyridine	<5%
7	2,6-lutidine	5%

Table S7. Additional base screening

[a] Reactions were set up at 0.1 mmol scale, yield determined by GC-FID analysis using 1,3,5-trimethoxybenzene (17 mg, 0.10 mmol, 1.0 equiv.) as an internal standard.

2.3.9. Control experiments

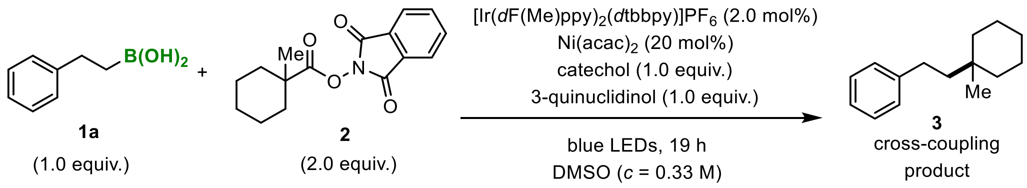

Entry	Changes from above	GC-FID yield[a]
**1**	**none**	**63%**
2	no photocatalyst	<5%
3	no $Ni(acac)_2$	<5%
4	no catechol	<5%
5	no 3-quinuclidinol	<5%

Table S8. Control experiments

[a] Reactions were set up at 0.1 mmol scale, yield determined by GC-FID analysis using 1,3,5-trimethoxybenzene (17 mg, 0.10 mmol, 1.0 equiv.) as an internal standard.

In the absence of $Ni(acac)_2$, the major product observed by GC-MS (see below) was ethyl toluene, formed by hydrodeboronation of **1a**. Cross-coupled product **3** and homo-coupled product 1,4-diphenylbutane were only observed in trace amounts (<5%).

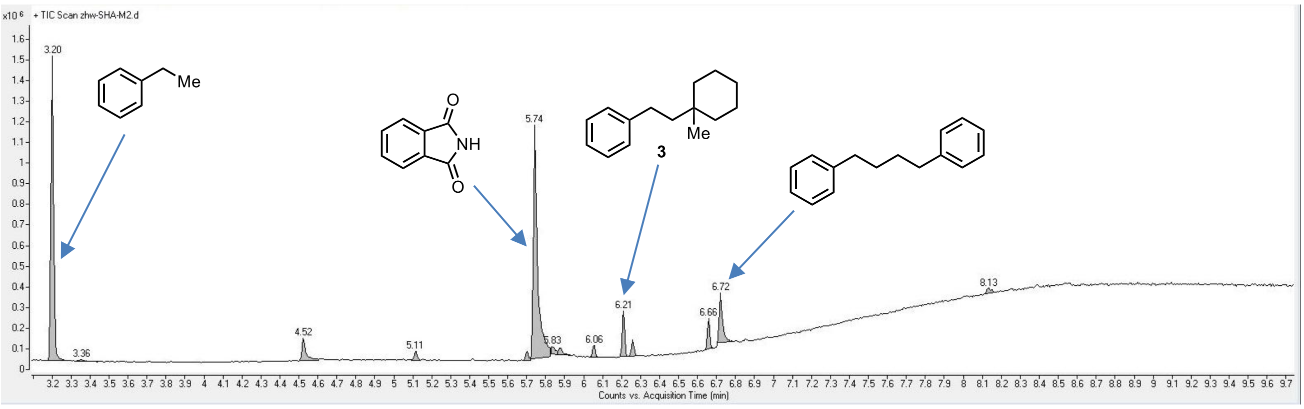

2.3.10. Reaction optimization with (4-phenylbutyl)boronic acid

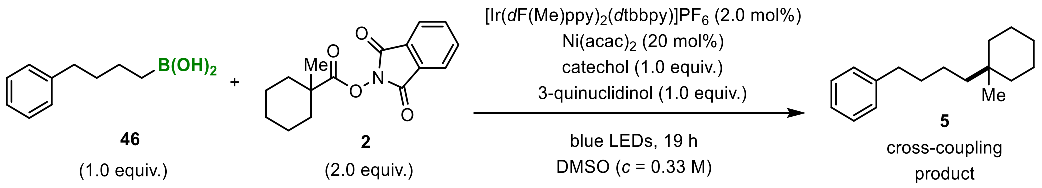

Entry	Changes from above	GC-FID yield[a]
**1**	**-**	**80% (75%)[b]**
2	*c* = 0.13 M	67%
3	*c* = 0.20 M	78%
4	[Ir(*d*F(Me)ppy)$_2$(*d*tbbpy)]PF$_6$ (1 mol%)	58%

Table S9. Reaction optimization with (4-phenylbutyl)boronic acid

[a] Reactions were set up at 0.1 mmol scale, yield determined by GC-FID analysis using 1,3,5-trimethoxybenzene (17 mg, 0.10 mmol, 1.0 equiv.) as an internal standard. [b] Reactions were set up at 0.2 mmol scale, Isolated yield.

2.3.11. Reaction optimization with *α*-*N*-carboxylic redox-active ester

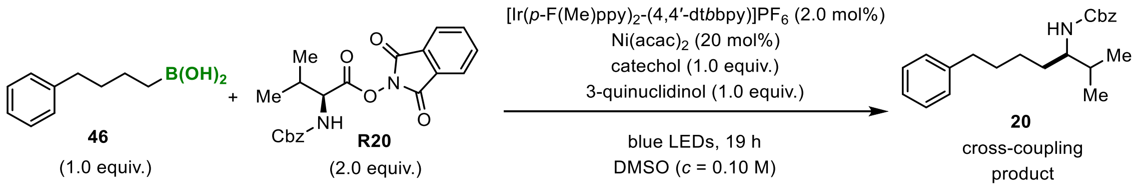

Entry	Changes from above	GC-FID yield[a]
**1**	**none**	**65%[b]**
2	[Ir(*d*F(Me)ppy)$_2$(*d*tbbpy)]PF$_6$ as photo catalyst [c]	40%
3	[Ir[*d*F(CF$_3$)$_2$ppy]$_2$(bpy)]PF$_6$ as photo catalyst [c]	29%
4	*c* = 0.33 M	45%[b]
5	10 mol% Ni(acac)$_2$	54%

Table S10. Reaction optimization with *α*-*N*-carboxylic redox-active ester

[a] Reactions were set up at 0.1 mmol scale, yield determined by GC-FID analysis using 1,3,5-trimethoxybenzene (17 mg, 0.10 mmol, 1.0 equiv.) as an internal standard. [b] Reaction was set up at 0.2 mmol scale, isolated yield. [c] Structure of photo catalyst is in Table S4.

2.3.12. Reaction optimization with alkene as reactant

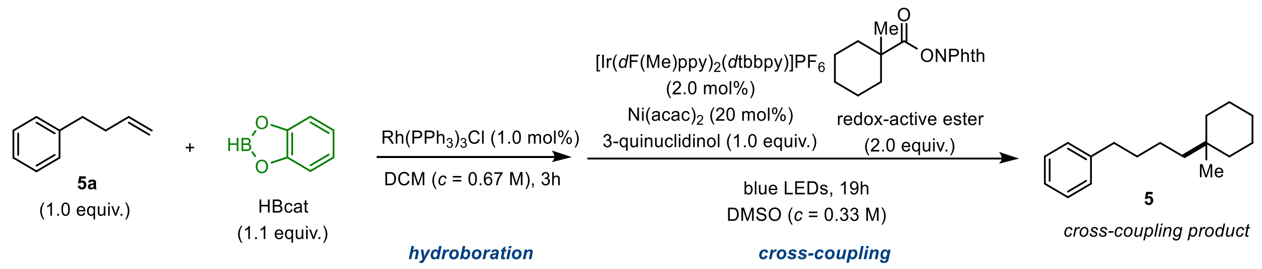

Entry	Changes from above	GC-FID yield[a]
**1**	**none**	**60% (58%)[b]**
2	2.0 eq. HBcat	5%
3	without solvent exchange	50%
4	20 mol% catechol as additive	53%
5	0.33 M concentration in hydroboration	35%
6	1h in hydroboration	50%
7	DMA (10 mol%) instead Rh catalyst in hydroboration	30%
8	no Rh catalyst and DCM in hydroboration	27%
9[c]	9-*H*-BBN instead HBcat	23%

Table S11. Reaction optimization with alkene as reactant

[a] Reactions were set up at 0.1 mmol scale, yield determined by GC-FID analysis using 1,3,5-trimethoxybenzene as an internal standard. [b] Reaction was set up at 0.2 mmol scale, isolated yield. [c] 9-*H*-BBN = 9-Borabicyclo[3.3.1]nonane (0.5 M solution in THF), reaction set up without $Rh(PPh_3)_3Cl$ and DCM.

2.3.13. Comparison with reported nickel-catalyzed reductive olefin hydroalkylation protocol

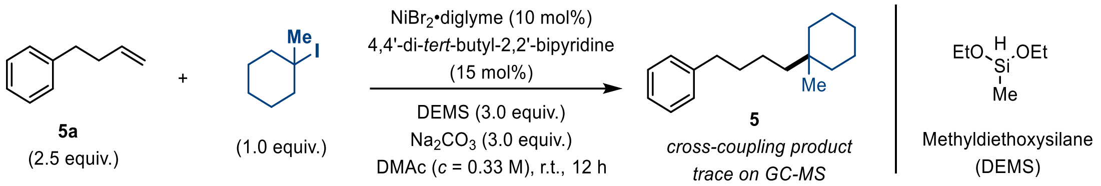

The reaction was set up according to reported protocol. Under an ambient atmosphere, a flame dried 8 mL Biotage® microwave reaction vial equipped with a magnetic stir bar was transferred into an anhydrous, argon-filled glovebox and charged with $NiBr_2$·diglyme (7.0 mg, 20 μmol, 10 mol%), 4,4'-di-*tert*-butyl-2,2'-bipyridine (8.0 mg, 30 μmol, 15 mol%) and Na_2CO_3 (64 mg, 0.60 mmol, 3.0 equiv.), followed by the anhydrous DMAc (0.60 mL, *c* = 0.33 M). The mixture was stirred at room temperature for 30 seconds in glovebox, then 1-iodo-1-methylcyclohexane (45mg, 0.2 mmol, 1.0 equiv.), (4-phenylbutyl)boronic acid **5a** (90 mg, 0.50 mmol, 2.5 equiv.) and DEMS (diethoxymethylsilane, 81 mg, 0.60 mmol, 3.0 equiv.) were added sequentially. The vial was sealed with a cap with septum, removed from the glovebox. After stirring at 1200 rpm for 12 h at room temperature, the reaction mixture was washed with deionized water (10 mL) and brine (10 mL), then extracted with EtOAc (3 × 20 mL). The combined organic layers were dried over $MgSO_4$, filtered, and concentrated *in vacuo* to get the crude sample, which was analyzed by GC-FID.

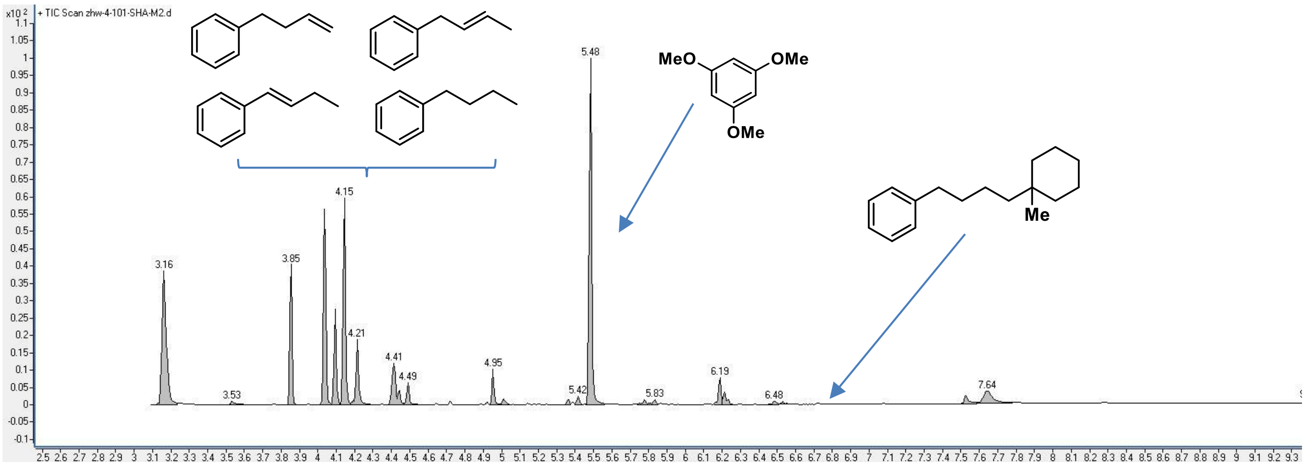

2.4. Synthesis of redox-active esters

2.4.1. Successful redox-active ester coupling partners

Redox-active esters were prepared according to literature procedures[1].

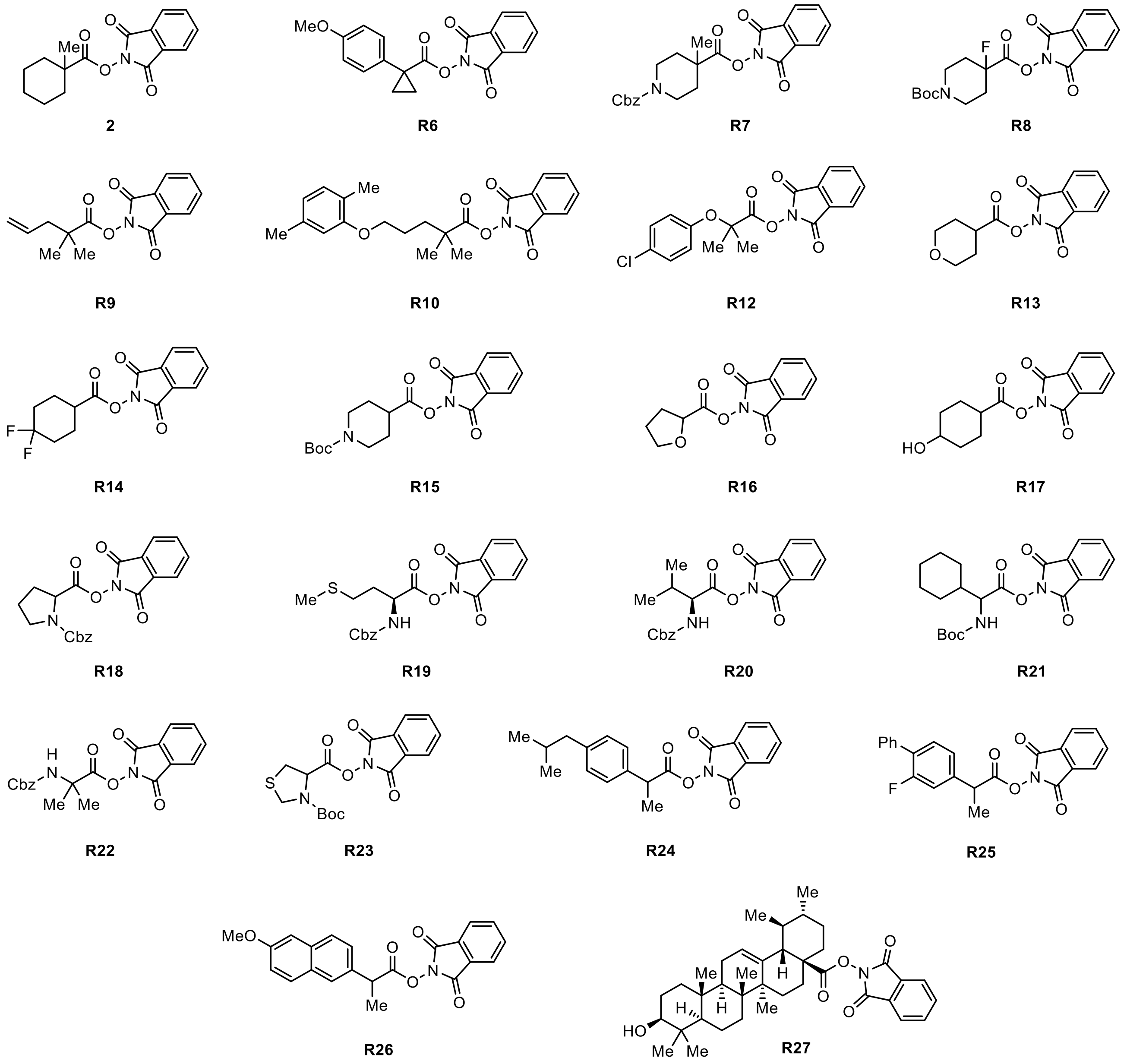

Figure S1. Successful redox-active ester coupling partners.

2.4.2. Synthesis of unreported redox-active esters

1,3-Dioxoisoindolin-2-yl ((benzyloxy)carbonyl)-*L*-valinate (R20)

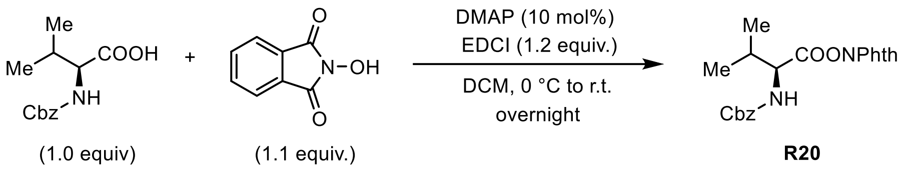

(Benzyloxy)carbonyl)-*L*-valine (1.26 g, 5.00 mmol, 1.00 equiv.) and 4-(dimethylamino)pyridine (DMAP, 61 mg, 0.50 mmol, 10 mol%) were added to a solution of *N*-hydroxyphthalimide (0.90 g, 5.5 mmol, 1.1 equiv.) in anhydrous DCM (20 mL, *c* = 0.050 M) at room temperature, followed by the portionwise addition of 1-ethyl-3-(3'-dimethylaminopropyl)carbodiimide hydrochloride (EDCI, 0.93 g, 6.0 mmol, 1.2 equiv.) at 0 ℃. The reaction warmed up to room temperature after 2 h and then stirred for 10 h. Subsequently, the solvent was removed *in vacuo* and a mixture of EtOAc/H_2O (v/v = 3:1) was added. The layers were separated, and the aqueous layer was extracted with EtOAc (2 × 30 mL). The combined organic layers were washed with saturated aqueous $NaHCO_3$ solution, dried over $MgSO_4$, filtered, and concentrated *in vacuo*. The residue was purified by flash column chromatography, eluting with EtOAc/pentane (10 – 40%, v/v) to give **R20** (1.4 g, 72%) as a white solid.

R*f* = 0.4 (1:20 EtOAc/pentane, UV, $KMnO_4$)

NMR Spectroscopy (*see spectra*):

^{1}**H NMR** (500 MHz, $CDCl_3$): δ_H 7.92 – 7.85 (m, 2H), 7.81 – 7.76 (m, 2H), 7.42 – 7.29 (m, 5H), 5.35 – 5.28 (m, 1H), 5.26 – 5.08 (m, 2H), 1.76 (s, 6H) ppm;

^{13}**C NMR** (126 MHz, $CDCl_3$) δ_C 171.0, 161.7, 154.9, 136.3, 134.9, 129.1, 128.7, 128.4, 128.3, 124.1, 67.3, 56.3, 31.1 ppm.

IR (film): ν_{max} 2978, 1745, 1699, 1390, 1368, 1159, 878, 697 cm^{-1}.

HRMS (ESI$^+$): calcd. for $C_{21}H_{20}O_6N_2Na$ [M+Na]$^+$, 419.1214, found 419.1217.

1,3-Dioxoisoindolin-2-yl (1*S*,2*R*,4a*S*,6a*S*,6b*R*,8a*R*,10*S*,12a*R*,12b*R*,14b*S*)-10-hydroxy-1,2,6a,6b,9,9,12a-heptamethyl-1,3,4,5,6,6a,6b,7,8,8a,9,10,11,12,12a,12b,13,14b-octadecahydropicene-4a(2*H*)-carboxylate (R27)

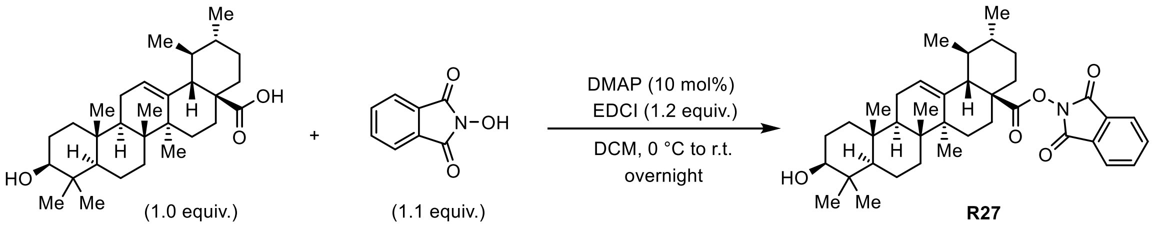

Ursolic acid (2.3 g, 5.0 mmol, 1.0 equiv.) and 4-(dimethylamino)pyridine (DMAP, 61 mg, 0.50 mmol, 10 mol%) were added to a solution of *N*-hydroxyphthalimide (0.90 g, 5.5 mmol, 1.1 equiv.) in anhydrous DCM (20 mL, *c* = 0.050 M) at room temperature, followed by the portionwise addition of 1-ethyl-3-(3'-dimethylaminopropyl)carbodiimide hydrochloride (EDCI, 0.93 g, 6.0 mmol, 1.2 equiv.) at 0 ℃. The reaction warmed up to room temperature after 2 h and then stirred for 10 h. Subsequently, the solvent was removed *in*

vacuo and a mixture of EtOAc/H_2O (v/v = 3:1) was added. The layers were separated, and the aqueous layer was extracted with EtOAc (2 × 30 mL). The combined organic layers were washed with saturated aqueous $NaHCO_3$ solution, dried over $MgSO_4$, filtered, and concentrated *in vacuo*. The residue was purified by flash column chromatography, eluting with EtOAc/pentane (2 – 30%, v/v) to give **R27** (2.0 g, 65%) as a white solid.

R_f = 0.3 (1:5 EtOAc/pentane, UV, $KMnO_4$)

NMR Spectroscopy (*see spectra*):

^{1}H NMR (500 MHz, $CDCl_3$): δ_H 7.89 – 7.82 (m, 2H), 7.79 – 7.73 (m, 2H), 5.34 – 5.28 (m, 1H), 3.27 – 3.19 (m, 1H), 2.32 – 2.27 (m, 1H), 2.25 – 2.17 (m, 1H), 2.15 – 2.06 (m, 2H), 1.96 – 1.89 (m, 3H), 1.69 – 1.29 (m, 15H), 1.13 (s, 3H), 1.02 – 0.85 (m, 16H), 0.80 – 0.77 (m, 3H), 0.76 – 0.71 (m, 1H) ppm;

^{13}C NMR (126 MHz, $CDCl_3$) δ_C 173.7, 162.3, 137.0, 134.7, 129.2, 126.8, 123.9, 79.2, 60.6, 55.4, 53.2, 48.9, 47.8, 42.6, 39.8, 39.6, 39.3, 38.9, 38.8, 37.1, 37.0, 33.4, 30.9, 28.6, 28.3, 27.4, 24.6, 23.5, 23.3, 21.3, 18.5, 17.4, 17.1, 17.1, 15.8, 15.7, 15.6, 14.4 ppm.

IR (film): ν_{max} 2927, 1746, 1371, 976, 697 cm^{-1}.

HRMS (MALDI$^+$): calcd. for $C_{38}H_{51}NO_3Na$ $[M+Na]^+$, 624.3659, found 624.3662.

2.5. Synthesis of terminal alkenes

2.5.1. Successful terminal alkene coupling partners

Terminal alkenes were prepared according to literature procedures or purchased from the suppliers indicated below.

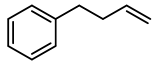

5a
commercially available
Sigma-Aldrich
[CAS 768-56-9]

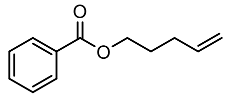

28a
Org. Lett.
2021, *23,* 6105.

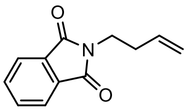

29a
Org. Lett.
2016, *18,* **5368.**

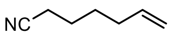

30a
commercially available
Alfa Aesar
[CAS 5048-25-9]

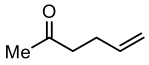

31a
commercially available
Sigma-Aldrich
[CAS 109-49-9]

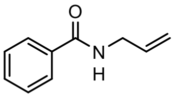

32a
J. Am. Chem. Soc.
2022, *144,* 5762.

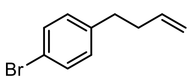

33a
commercially available
Apollo
[CAS 15451-32-8]

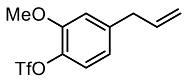

34a
J. Am. Chem. Soc.
2017, *139,* 5998.

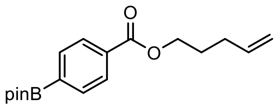

35a
see supporting information

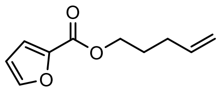

36a
Angew. Chem. Int. Ed.
2014, *53,* 8654.

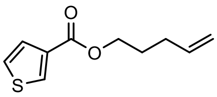

37a
see supporting information

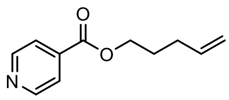

38a
see supporting information

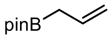

39a
commercially available
Sigma-Aldrich
[CAS 72824-04-5]

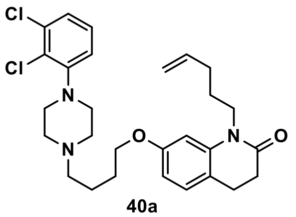

40a
see supporting information

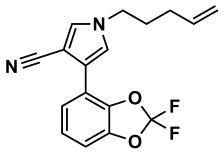

41a
see supporting information

Figure S2. Successful terminal alkene coupling partners

2.5.2. Synthesis of unreported terminal alkenes

Pent-4-en-1-yl 4-(4,4,5,5-tetramethyl-1,3,2-dioxaborolan-2-yl)benzoate (35a)

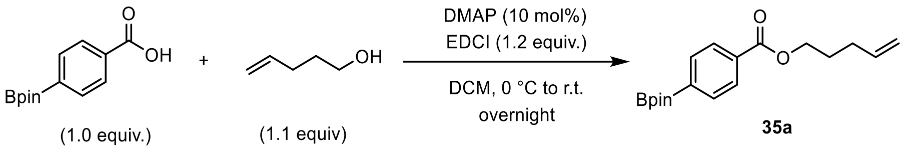

35a

4-(4,4,5,5-Tetramethyl-1,3,2-dioxaborolan-2-yl)benzoic acid (1.24 g, 5.00 mmol, 1.00 equiv.) and 4-(dimethylamino)pyridine (DMAP, 61 mg, 0.50 mmol, 10 mol%) were added to a solution of pent-4-en-1-ol (474 mg, 5.50 mmol, 1.10 equiv.) in anhydrous DCM (20 mL, *c* = 0.050 M) at room temperature, followed by the portionwise addition of 1-ethyl-3-(3'-dimethylaminopropyl)carbodiimide hydrochloride (EDCI, 0.93 g, 6.0 mmol, 1.2 equiv.) at 0 ℃. The reaction warmed up to room temperature after 2 h and then stirred for 10 h. Subsequently, the solvent was removed *in vacuo* and a mixture of EtOAc/H_2O (v/v = 3:1) was added. The layers were separated, and the aqueous layer was extracted with EtOAc (2 × 30 mL). The combined organic layers were washed with saturated aqueous $NaHCO_3$ solution, dried over $MgSO_4$, filtered, and concentrated *in vacuo*. The residue was purified by flash column chromatography, eluting with EtOAc/pentane (0 – 10%, v/v) to give **35a** (1.3 g, 81%) as a colorless oil.

$\mathbf{R}_f$ = 0.70 (1:10 EtOAc/pentane, UV, $KMnO_4$)

NMR Spectroscopy (*see spectra*):

^{1}H NMR (400 MHz, $CDCl_3$) δ_H 8.03 – 7.99 (m, 2H), 7.90 – 7.83 (m, 2H), 5.91 – 5.79 (m, 1H), 5.11 – 4.99 (m, 2H), 4.34 (t, J = 6.6 Hz, 2H), 2.26 – 2.19 (m, 2H), 1.93 – 1.84 (m, 2H), 1.36 (s, 12H) ppm;

^{13}C NMR (101 MHz, $CDCl_3$) δ_C 166.8, 137.6, 134.8, 132.7, 128.7, 115.5, 84.3, 64.6, 30.3, 28.0, 25.0 ppm.

^{11}B NMR (128 MHz, $CDCl_3$) δ 29.5 ppm.

IR (film): ν_{max} 2979, 1718, 1399, 1358, 1265, 1110, 963, 858, 709, 651 cm^{-1}.

HRMS (EI^+): calcd. for $C_{18}H_{25}O_4B$ $[M]^+$, 316.1840, found 316.1837.

Pent-4-en-1-yl thiophene-3-carboxylate (37a)

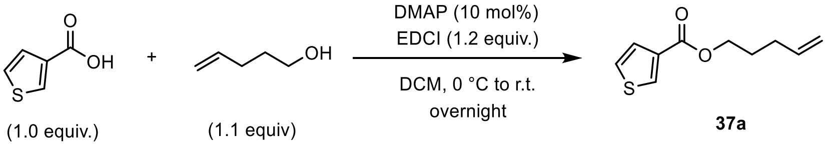

37a

3-Thiophene carboxylic acid (641 mg, 5.00 mmol, 1.00 equiv.) and 4-(dimethylamino)pyridine (DMAP, 61 mg, 0.50 mmol, 10 mol%) were added to a solution of pent-4-en-1-ol (474 mg, 5.50 mmol, 1.10 equiv.) in anhydrous DCM (20 mL, *c* = 0.050 M) at room temperature, followed by the portionwise addition of 1-ethyl-3-(3'-dimethylaminopropyl)carbodiimide hydrochloride (EDCI, 0.93 g, 6.0 mmol, 1.2 equiv.) at 0 ℃. The reaction warmed up to room temperature after 2 h and then stirred for 10 h. Subsequently, the solvent was removed *in vacuo* and a mixture of EtOAc/H_2O (v/v = 3:1) was added. The layers were separated, and the aqueous layer was extracted with EtOAc (2 × 30 mL). The combined organic layers were washed with saturated aqueous

$NaHCO_3$ solution, dried over $MgSO_4$, filtered, and concentrated *in vacuo*. The residue was purified by flash column chromatography, eluting with EtOAc/pentane (0 – 10%, v/v) to give **37a** (0.73 g, 74%) as a colorless oil.

R*f* = 0.50 (1:20 EtOAc/pentane, UV, $KMnO_4$)

NMR Spectroscopy (*see spectra*):

^{1}**H NMR** (400 MHz, $CDCl_3$) δ_H 8.10 (dd, *J* = 3.0, 1.2 Hz, 1H), 7.53 (dd, *J* = 5.1, 1.2 Hz, 1H), 7.30 (dd, *J* = 5.1, 3.0 Hz, 1H), 5.90 – 5.78 (m, 1H), 5.11 – 4.98 (m, 2H), 4.29 (t, *J* = 6.6 Hz, 2H), 2.25 – 2.16 (m, 2H), 1.91 – 1.79 (m, 2H) ppm;

^{13}**C NMR** (101 MHz, $CDCl_3$) δ_C 162.9, 137.6, 134.0, 132.7, 128.0, 126.1, 115.5, 64.2, 30.3, 28.1 ppm.

IR (film): ν_{max} 3113, 2955, 1711, 1410, 1253, 1188, 1101, 913, 745, 699 cm^{-1}.

HRMS (EI$^+$): calcd. for $C_{10}H_{12}O_2S$ [M]$^+$, 196.0553, found 196.0550.

Pent-4-en-1-yl isonicotinate (38a)

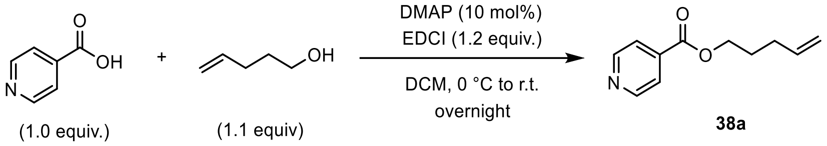

p-pyridinecarboxylic acid (616 mg, 5.00 mmol, 1.00 equiv.) and 4-(dimethylamino)pyridine (DMAP, 61 mg, 0.50 mmol, 10 mol%) were added to a solution of pent-4-en-1-ol (474 mg, 5.50 mmol, 1.10 equiv.) in anhydrous DCM (20 mL, *c* = 0.050 M) at room temperature, followed by the portionwise addition of 1-ethyl-3-(3'-dimethylaminopropyl)carbodiimide hydrochloride (EDCI, 0.93 g, 6.0 mmol, 1.2 equiv.) at 0 ℃. The reaction warmed up to room temperature after 2 h and then stirred for 10 h. Subsequently, the solvent was removed *in vacuo* and a mixture of EtOAc/H_2O (v/v = 3:1) was added. The layers were separated, and the aqueous layer was extracted with EtOAc (2 × 30 mL). The combined organic layers were washed with saturated aqueous $NaHCO_3$ solution, dried over $MgSO_4$, filtered, and concentrated *in vacuo*. The residue was purified by flash column chromatography, eluting with EtOAc/pentane (0 – 10%, v/v) to give **38a** (0.67 g, 70%) as a colorless oil.

R*f* = 0.50 (1:20 EtOAc/pentane, UV, $KMnO_4$)

NMR Spectroscopy (*see spectra*):

^{1}**H NMR** (400 MHz, $CDCl_3$) δ_H 8.83 – 8.73 (m, 2H), 7.87 – 7.81 (m, 2H), 5.90 – 5.77 (m, 1H), 5.11 – 4.99 (m, 2H), 4.37 (t, *J* = 6.6 Hz, 2H), 2.26 – 2.18 (m, 2H), 1.93 – 1.85 (m, 2H) ppm;

^{13}**C NMR** (101 MHz, $CDCl_3$) δ_C 165.3, 150.7, 137.7, 137.3, 123.0, 115.7, 65.3, 30.2, 27.9 ppm.

IR (film): ν_{max} 3078, 2958, 1726, 1562, 1407, 1275, 1118, 914, 756, 676 cm^{-1}.

HRMS (EI$^+$): calcd. for $C_{11}H_{12}NO_2$ [M-H]$^+$, 190.0863, found 190.0860.

7-(4-(4-(2,3-Dichlorophenyl)piperazin-1-yl)butoxy)-1-(pent-4-en-1-yl)-3,4-dihydroquinolin-2(1H)-one (40a)

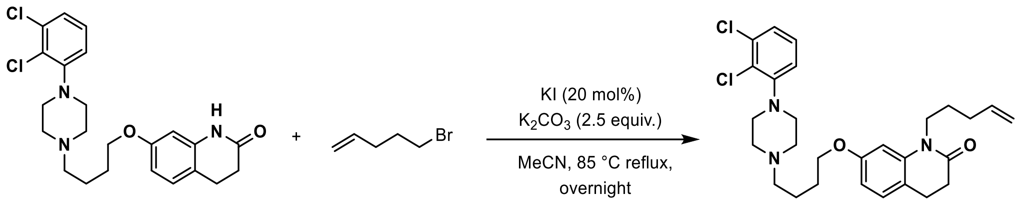

(1.0 equiv.) (2.0 equiv) **40a**

Bromopentene (1.49 g, 10.0 mmol, 2.00 equiv.), potassium iodide (KI, 166 mg, 1.00 mmol, 20.0 mol%) and potassium carbonate (K_2CO_3, 1.73 g, 12.5 mmol, 2.50 equiv.) were added to a solution of Aripiprazole (2.24 g, 5.00 mmol, 1.00 equiv.) in anhydrous acetonitrile (20 mL, *c* = 0.25 M) at room temperature. The resulting suspension was stirred at 85 ℃ reflux for overnight. Subsequently, the solution was quenched by saturated aqueous NH_4Cl and a mixture of EtOAc/H_2O (v/v = 3:1) was added. The layers were separated, and the aqueous layer was extracted with EtOAc (2 × 30 mL). The combined organic layers were washed with brine, dried over $MgSO_4$, filtered, and concentrated *in vacuo*. The residue was purified by flash column chromatography, eluting with EtOAc/pentane (30% – 100%, v/v) to give **40a** (1.9 g, 74%) as a pale-yellow oil.

R*f* = 0.30 (3:1 EtOAc/pentane, UV, $KMnO_4$)

NMR Spectroscopy (*see spectra*):

^{1}**H NMR** (400 MHz, $CDCl_3$) δ_H 7.18 – 7.12 (m, 2H), 7.06 – 7.02 (m, 1H), 6.98 – 6.93 (m, 1H), 6.58 – 6.49 (m, 2H), 5.90 – 5.80 (m, 1H), 5.09 – 4.98 (m, 2H), 3.99 (t, *J* = 6.3 Hz, 2H), 3.94 – 3.88 (m, 2H), 3.08 (s, 4H), 2.84 – 2.77 (m, 2H), 2.76 – 2.56 (m, 6H), 2.54 – 2.46 (m, 2H), 2.18 – 2.11 (m, 2H), 1.88 – 1.81 (m, 2H), 1.80 – 1.69 (m, 4H) ppm;

^{13}**C NMR** (126 MHz, $CDCl_3$) δ_C 170.4, 158.8, 151.4, 140.7, 137.8, 134.2, 128.6, 127.7, 127.6, 124.7, 118.91, 118.7, 115.4, 107.2, 103.2, 68.1, 58.4, 53.5, 51.5, 41.9, 32.4, 31.2, 27.5, 26.4, 25.0, 23.6 ppm.

IR (film): ν_{max} 2942, 2817, 1671, 1578, 1357, 1136, 1044, 961, 779, 735, 713 cm^{-1}.

HRMS (ESI$^+$): calcd. for $C_{28}H_{36}O_2N_3Cl_2$ [M+H]$^+$, 516.2179, found 516.2184.

4-(2,2-Difluorobenzo[*d*][1,3]dioxol-4-yl)-1-(pent-4-en-1-yl)-1*H*-pyrrole-3-carbonitrile (41a)

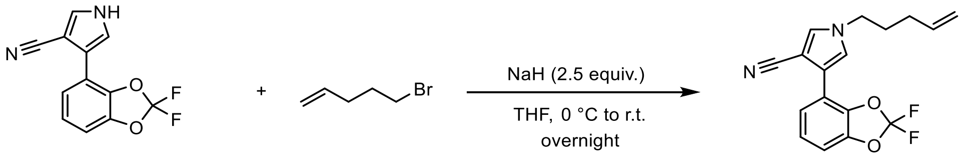

(1.0 equiv.) (1.5 equiv) **41a**

A flame dried 100 mL round bottom flask equipped with a magnetic stir bar was charged with sodium hydride (NaH, 60.0 % dispersion in mineral oil, 300 mg, 12.5 mmol, 2.50 equiv.) in anhydrous THF (20 mL, *c* = 0.25 M). The solution was cooled to 0 ℃, Fludioxonil (1.24 g, 5.00 mmol, 1.00 equiv.) was added in one portion, then stirred at 0 ℃ for 30 min. Then bromopentene (1.49 g, 10.0 mmol, 1.00 equiv.) was added dropwise at 0 ℃.

The resulting suspension was stirred at room temperature overnight. Subsequently, the solution was quenched by saturated aqueous NH_4Cl and a mixture of EtOAc/H_2O (v/v = 3:1) was added. The layers were separated, and the aqueous layer was extracted with EtOAc (2 × 30 mL). The combined organic layers were washed with brine, dried over $MgSO_4$, filtered, and concentrated *in vacuo*. The residue was purified by flash column chromatography, eluting with EtOAc/pentane (20% – 100%, v/v) to give **41a** (1.1 g, 66%) as a white solid.

R*f* = 0.30 (3:1 EtOAc/pentane, UV, $KMnO_4$)

NMR Spectroscopy (*see spectra*):

> **^{1}H NMR** (400 MHz, $CDCl_3$) δ_H 7.76 – 7.68 (m, 1H), 7.25 – 7.22 (m, 1H), 7.19 – 7.10 (m, 2H), 7.00 – 6.93 (m, 1H), 5.85 – 5.71 (m, 1H), 5.13 – 5.04 (m, 2H), 3.96 (t, *J* = 7.1 Hz, 2H), 2.15 – 2.05 (m, 2H), 1.99 – 1.90 (m, 2H) ppm;

> **^{13}C NMR** (101 MHz, $CDCl_3$) δ_C 144.0, 139.9, 136.5, 131.5 (t, *J* = 255.5 Hz), 129.6, 124.2, 122.5, 121.3, 118.7, 116.67, 116.65, 116.62, 107.9, 91.2, 49.9, 30.4, 30.0 ppm.

IR (film): ν_{max} 2926, 2223, 1456, 1246, 1150, 1033, 920, 721 cm^{-1}.

HRMS (EI$^+$): calcd. for $C_{17}H_{14}N_2O_2F_2$ [M]$^+$, 316.1018, found 316.1014.

2.6. Substrate Scope

(2-(1-Methylcyclohexyl)ethyl)benzene (3)

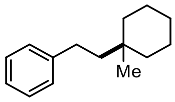

3

Prepared following **General Procedure A**, using (2-phenylethyl)boronic acid (30 mg, 0.20 mmol, 1.0 equiv.), 1,3-dioxoisoindolin-2-yl 1-methylcyclohexane-1-carboxylate **2** (115 mg, 0.400 mmol, 2.00 equiv.), [Ir(*d*F(Me)ppy)$_2$(*d*tbbpy)]PF$_6$ (4.0 mg, 4.0 μmol, 2.0 mol%), Ni(acac)$_2$ (10 mg, 0.040 mmol, 20 mol%), catechol (22 mg, 0.20 mmol, 1.0 equiv), 3-quinuclidinol (25 mg, 0.20 mmol, 1.0 equiv.) in anhydrous DMSO (0.60 mL, *c* = 0.33 M). Biotage Isolera™ flash purification on silica gel (SNAP 5 g silica cartridge), eluting with pentane (100%), gave **3** (23 mg, 56%) as a colorless oil.

R*f* = 0.8 (pentane, UV, $KMnO_4$)

NMR Spectroscopy (*see spectra*):

> **^{1}H NMR** (500 MHz, $CDCl_3$): δ_H 7.30 – 7.26 (m, 2H), 7.21 – 7.15 (m, 3H), 2.57 – 2.51 (m, 2H), 1.54 – 1.40 (m, 7H), 1.38 – 1.24 (m, 5H), 0.95 (s, 3H) ppm;
>
> **^{13}C NMR** (126 MHz, $CDCl_3$): δ_C 143.8, 128.4, 128.3, 125.5, 44.3, 37.8, 32.8, 30.0, 26.6, 25.1, 22.1 ppm.

IR (film) ν_{max}: 2919, 2851, 1464, 695 cm^{-1}.

HRMS (EI$^+$): calcd. for $C_{15}H_{22}$ [M]$^+$, 202.1716, found 202.1712.

(3-(1-Methylcyclohexyl)propyl)benzene (4)

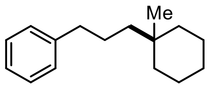

4

Prepared following **General Procedure A**, using (3-phenylpropyl)boronic acid (33 mg, 0.20 mmol, 1.0 equiv.), 1,3-dioxoisoindolin-2-yl 1-methylcyclohexane-1-carboxylate **2** (115 mg, 0.400 mmol, 2.00 equiv.), [Ir(*d*F(Me)ppy)$_2$(*d*tbbpy)]PF$_6$ (4.0 mg, 4.0 μmol, 2.0 mol%), Ni(acac)$_2$ (10 mg, 0.040 mmol, 20 mol%), catechol (22 mg, 0.20 mmol, 1.0 equiv), 3-quinuclidinol (25 mg, 0.20 mmol, 1.0 equiv.) in anhydrous DMSO (0.60 mL, *c* = 0.33 M). Biotage Isolera™ flash purification on silica gel (SNAP 5 g silica cartridge), eluting with pentane (100%), gave **4** (31 mg, 72%) as a colorless oil.

R*f* = 0.8 (pentane, UV, $KMnO_4$)

NMR Spectroscopy (*see spectra*):

> **^{1}H NMR** (400 MHz, $CDCl_3$): δ_H 7.32 – 7.22 (m, 2H), 7.22 – 7.12 (m, 3H), 2.57 (t, *J* = 7.8 Hz, 2H), 1.63 – 1.49 (m, 2H), 1.46 – 1.34 (m, 5H), 1.32 – 1.17 (m, 7H), 0.83 (s, 3H) ppm;
>
> **^{13}C NMR** (101 MHz, $CDCl_3$): δ_C 143.2, 128.5, 128.4, 125.7, 42.0, 38.0, 37.1, 32.7, 26.7, 25.6, 25.1, 22.2 ppm.

IR (film) v_{max}: 2925, 2850, 1453, 748, 697 cm^{-1}

HRMS (EI$^+$): calcd. for $C_{16}H_{24}$ [M]$^+$, 216.1873, found 216.1870.

(4-(1-Methylcyclohexyl)butyl)benzene (5)

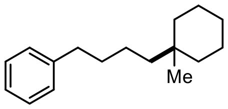

5

Prepared following **General Procedure A**, using (4-phenylbutyl)boronic acid (36 mg, 0.20 mmol, 1.0 equiv.), 1,3-dioxoisoindolin-2-yl 1-methylcyclohexane-1-carboxylate **2** (115 mg, 0.400 mmol, 2.00 equiv.), [Ir(*d*F(Me)ppy)$_2$(*d*tbbpy)]PF$_6$ (4.0 mg, 4.0 μmol, 2.0 mol%), Ni(acac)$_2$ (10 mg, 0.040 mmol, 20 mol%), catechol (22 mg, 0.20 mmol, 1.0 equiv), 3-quinuclidinol (25 mg, 0.20 mmol, 1.0 equiv.) in anhydrous DMSO (0.60 mL, *c* = 0.33 M). Biotage Isolera™ flash purification on silica gel (SNAP 5 g silica cartridge), eluting with pentane (100%), gave **5** (35 mg, 75%) as a colorless oil.

Prepared following **General Procedure B**, using 4-Phenyl-1-butene (26 mg, 0.20 mmol, 1.0 equiv.), HBcat (26.4 mg, 0.220 mmol, 1.10 equiv.), Rh(PPh$_3$)$_3$Cl (2.0 mg, 2.0 μmol, 1.0 mol%) in anhydrous DCM (0.30 mL, *c* = 0.67 M) for hydroboration process. After 3 h stirring, DCM was removed and 1,3-dioxoisoindolin-2-yl 1-methylcyclohexane-1-carboxylate **2** (115 mg, 0.400 mmol, 2.00 equiv.), [Ir(*d*F(Me)ppy)$_2$(*d*tbbpy)]PF$_6$ (4.0 mg, 4.0 μmol, 2.0 mol%), Ni(acac)$_2$ (10 mg, 0.040 mmol, 20 mol%), 3-quinuclidinol (25 mg, 0.20 mmol, 1.0 equiv.) and anhydrous DMSO (0.60 mL, *c* = 0.33 M) were added for cross-coupling process. Biotage Isolera™ flash purification on silica gel (SNAP 5 g silica cartridge), eluting with pentane (100%), gave **5** (27 mg, 58%) as a colorless oil.

R*f* = 0.7 (pentane, UV, $KMnO_4$)

NMR Spectroscopy (*see spectra*):

^{1}**H NMR** (400 MHz, $CDCl_3$): δ_H 7.31 – 7.23 (m, 2H), 7.22 – 7.13 (m, 3H), 2.61 (t, *J* = 8.0, 2H), 1.64 – 1.54 (m, 2H), 1.47 – 1.36 (m, 5H), 1.34 – 1.16 (m, 9H), 0.83 (s, 3H) ppm;

^{13}**C NMR** (101 MHz, $CDCl_3$): δ_C 143.1, 128.5, 128.4, 125.7, 42.1, 38.0, 36.2, 32.8, 32.7, 26.8, 25.2, 23.2, 22.3 ppm.

IR (film) v_{max}: 2924, 2857, 1496, 1453, 1376, 744, 697 cm^{-1}.

HRMS (EI$^+$): calcd. for $C_{17}H_{26}$ [M]$^+$, 230.2029, found 230.2025.

1-Methoxy-4-(1-(4-phenylbutyl)cyclopropyl)benzene (6)

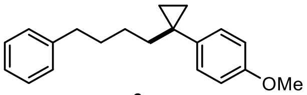

6

Prepared following **General Procedure A**, using (4-phenylbutyl)boronic acid (36 mg, 0.20 mmol, 1.0 equiv.), 1,3-dioxoisoindolin-2-yl 1-(4-methoxyphenyl)cyclopropane-1-carboxylate **R6** (135 mg, 0.400 mmol, 2.00 equiv.),

[Ir(*d*F(Me)ppy)$_2$(*d*tbbpy)]PF_6 (4.0 mg, 4.0 μmol, 2.0 mol%), Ni(acac)$_2$ (10 mg, 0.040 mmol, 20 mol%), catechol (22 mg, 0.20 mmol, 1.0 equiv), 3-quinuclidinol (25 mg, 0.20 mmol, 1.0 equiv.) in anhydrous DMSO (0.60 mL, *c* = 0.33 M). Biotage Isolera™ flash purification on silica gel (SNAP 5 g silica cartridge), eluting with EtOAc/pentane (0 – 5%, v/v) and evacuating with an oil pump for overnight to remove decaboxylative hydrogenation side product, gave **6** (31 mg, 55%) as a colorless oil.

R*f* = 0.5 (1:20 EtOAc/pentane, UV, $KMnO_4$)

NMR Spectroscopy (*see spectra*):

^{1}H NMR (400 MHz, $CDCl_3$): δ_H 7.30 – 7.08 (m, 7H), 6.86 – 6.77 (m, 2H), 3.79 (s, 3H), 2.57 – 2.47 (m, 2H), 1.59 – 1.48 (m, 4H), 1.41 – 1.26 (m, 2H), 0.77 – 0.68 (m, 2H), 0.65 – 0.56 (m, 2H) ppm;

^{13}C NMR (101 MHz, $CDCl_3$): δ_C 157.8, 143.0, 137.8, 130.3, 128.5, 128.4, 125.7, 113.5, 55.4, 40.7, 36.1, 31.8, 27.1, 25.1, 13.0 ppm.

IR (film): ν_{max} 2920, 2852, 1513, 1242, 1175, 1035, 830, 698 cm^{-1}.

HRMS (ESI$^+$): calcd. for $C_{20}H_{24}O$ [M+H]$^+$, 280.1822, found 280.1822.

Benzyl 4-methyl-4-(4-phenylbutyl)piperidine-1-carboxylate (7)

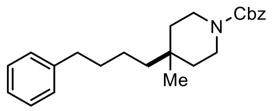

7

Prepared following modified **General Procedure A**, using (4-phenylbutyl)boronic acid (36 mg, 0.20 mmol, 1.0 equiv.), 1-benzyl 4-(1,3-dioxoisoindolin-2-yl) 4-methylpiperidine-1,4-dicarboxylate **R7** (169 mg, 0.400 mmol, 2.00 equiv.), [Ir(*p*-F(Me)ppy)$_2$-(4,4'-*d*tbbpy)]PF_6 (4.0 mg, 4.0 μmol, 2.0 mol%), Ni(acac)$_2$ (10 mg, 0.040 mmol, 20 mol%), catechol (22 mg, 0.20 mmol, 1.0 equiv), 3-quinuclidinol (25 mg, 0.20 mmol, 1.0 equiv.) in anhydrous DMSO (2.0 mL, *c* = 0.10 M). Biotage Isolera™ flash purification on silica gel (SNAP 5 g silica cartridge) eluting with EtOAc/pentane (0 – 30%, v/v), followed by preparative thin-layer chromatography purification with EtOAc/pentane (20%, v/v), gave **7** (46 mg, 62%) as a colorless oil.

R*f* = 0.4 (1:5 EtOAc/pentane, UV, $KMnO_4$)

NMR Spectroscopy (*see spectra*):

^{1}H NMR (400 MHz, $CDCl_3$): δ_H 7.29 – 7.16 (m, 8H), 7.13 – 7.04 (m, 3H), 5.03 (s, 2H), 3.53 (d, *J* = 13.5 Hz, 2H), 3.22 – 3.15 (m, 2H), 2.63 – 2.48 (m, 2H), 1.56 – 1.45 (m, 3H), 1.24 – 1.00 (m, 8H), 0.82 (s, 3H) ppm;

^{13}C NMR (101 MHz, $CDCl_3$): δ_C 155.6, 142.8, 137.2, 128.6, 128.5, 128.4, 128.0, 127.9, 125.8, 67.0, 44.4, 41.7, 40.3, 36.0, 32.4, 31.4, 23.5, 22.9 ppm.

IR (film): ν_{max} 2928, 2857, 1700, 1429, 1382, 1244, 1176, 698 cm^{-1}.

HRMS (ESI$^+$): calcd. for $C_{24}H_{32}O_2N$ [M+H]$^+$, 366.2428, found 366.2427.

tert-Butyl 4-(5-(benzoyloxy)pentyl)-4-fluoropiperidine-1-carboxylate (8)

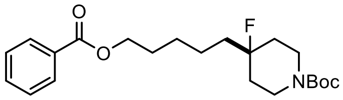

8

Prepared following modified **General Procedure A**, using (5-(benzoyloxy)pentyl)boronic acid (47 mg, 0.20 mmol, 1.0 equiv.), 1-(*tert*-butyl) 4-(1,3-dioxoisoindolin-2-yl) 4-fluoropiperidine-1,4-dicarboxylate **R8** (157 mg, 0.400 mmol, 2.00 equiv.), [Ir(*p*-F(Me)ppy)$_2$-(4,4'-*d*tbbpy)]PF_6 (4.0 mg, 4.0 μmol, 2.0 mol%), Ni(acac)$_2$ (10 mg, 0.040 mmol, 20 mol%), catechol (22 mg, 0.20 mmol, 1.0 equiv), 3-quinuclidinol (25 mg, 0.20 mmol, 1.0 equiv.) in anhydrous DMSO (2.0 mL, *c* = 0.10 M). Biotage Isolera^{TM} flash purification on silica gel (SNAP 5 g silica cartridge), eluting with EtOAc/pentane (0 – 30%, v/v), gave **8** (25 mg, 32%) as a colorless oil.

R*f* = 0.4 (1:5 EtOAc/pentane, UV, $KMnO_4$)

NMR Spectroscopy (*see spectra*):

^{1}**H NMR** (400 MHz, $CDCl_3$): δ_H 8.08 – 8.00 (m, 2H), 7.59 – 7.52 (m, 1H), 7.48 – 7.40 (m, 2H), 4.32 (t, *J* = 6.6 Hz, 2H), 4.06 – 3.77 (m, 2H), 3.06 (t, *J* = 12.5 Hz, 2H), 1.85 – 1.73 (m, 4H), 1.68 – 1.54 (m, 3H), 1.53 – 1.41 (m, 5H), 1.46 (s, 9H) ppm;

^{13}**C NMR** (101 MHz, $CDCl_3$): δ_C 166.8, 154.9, 133.0, 130.6, 129.7, 128.5, 94.9, 93.2, 79.7, 65.0, 40.3 (d, *J* = 22.6 Hz), 34.6 (d, *J* = 22.0 Hz), 28.8, 28.6, 26.5, 22.5 (d, *J* = 4.4 Hz) ppm.

^{19}**F NMR** (377 MHz, $CDCl_3$): δ_F –162.74 ppm.

IR (film): ν_{max} 2929, 1720, 1695, 1422, 1274, 1155, 1114, 713 cm^{-1}.

HRMS (ESI$^+$): calcd. for $C_{22}H_{32}O_4NFNa$ [M+Na]$^+$, 416.2208, found 416.2204.

(5,5-Dimethyloct-7-en-1-yl)benzene (9)

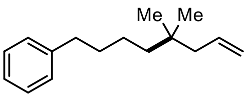

9

Prepared following **General Procedure A**, using (4-phenylbutyl)boronic acid (36 mg, 0.20 mmol, 1.0 equiv.), 1,3-dioxoisoindolin-2-yl 2,2-dimethylpent-4-enoate **R9** (109 mg, 0.400 mmol, 2.00 equiv.), [Ir(*d*F(Me)ppy)$_2$(*d*tbbpy)]PF_6 (4.0 mg, 4.0 μmol, 2.0 mol%), Ni(acac)$_2$ (10 mg, 0.040 mmol, 20 mol%), catechol (22 mg, 0.20 mmol, 1.0 equiv), 3-quinuclidinol (25 mg, 0.20 mmol, 1.0 equiv.) in anhydrous DMSO (0.60 mL, *c* = 0.33 M). Biotage Isolera^{TM} flash purification on silica gel (SNAP 5 g silica cartridge), eluting with pentane (100%), gave **9** (26 mg, 60%) as a colorless oil.

R*f* = 0.5 (pentane, UV, $KMnO_4$)

NMR Spectroscopy (*see spectra*):

^{1}**H NMR** (400 MHz, $CDCl_3$): δ_H 7.31 – 7.24 (m, 3H), 7.21 – 7.14 (m, 3H), 5.84 – 5.77 (m, 1H), 5.06 – 4.91 (m, 2H), 2.65 – 2.57 (m, 2H), 1.94 (dt, J = 7.5, 1.2 Hz, 2H), 1.64 – 1.54 (m, 3H), 1.36 – 1.18 (m, 5H), 0.84

(s, 6H) ppm;

^{13}**C NMR** (101 MHz, $CDCl_3$): δ_C 143.0, 136.0, 128.5, 128.4, 125.7, 116.7, 46.6, 41.9, 36.2, 33.2, 32.6, 27.1, 23.9 ppm.

IR (film): ν_{max} 2929, 2856, 1466, 1365, 912, 744, 698 cm^{-1}.

HRMS (ESI^+): calcd. for $C_{16}H_{24}$ $[M+H]^+$, 216.1873, found 216.1868.

2-((4,4-Dimethyl-8-phenyloctyl)oxy)-1,4-dimethylbenzene (10)

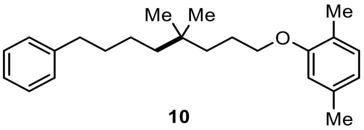

10

Prepared following **General Procedure A**, using (4-phenylbutyl)boronic acid (36 mg, 0.20 mmol, 1.0 equiv.), 1,3-dioxoisoindolin-2-yl 5-(2,5-dimethylphenoxy)-2,2-dimethylpentanoate **R10** (158 mg, 0.400 mmol, 2.00 equiv.), $[Ir(dF(Me)ppy)_2(dtbbpy)]PF_6$ (4.0 mg, 4.0 μmol, 2.0 mol%), $Ni(acac)_2$ (10 mg, 0.040 mmol, 20 mol%), catechol (22 mg, 0.20 mmol, 1.0 equiv), 3-quinuclidinol (25 mg, 0.20 mmol, 1.0 equiv.) in anhydrous DMSO (0.60 mL, *c* = 0.33 M). Biotage Isolera™ flash purification on silica gel (SNAP 5 g silica cartridge), eluting with pentane (100%), gave **10** (48 mg, 71%) as a colorless oil.

R*f* = 0.3 (pentane, UV, $KMnO_4$)

NMR Spectroscopy (*see spectra*):

^{1}**H NMR** (400 MHz, $CDCl_3$): δ_H 7.31 – 7.24 (m, 2H), 7.22 – 7.14 (m, 3H), 7.01 (d, *J* = 8.0 Hz, 1H), 6.71 – 6.59 (m, 2H), 3.91 (t, *J* = 6.4 Hz, 2H), 2.62 (t, *J* = 8.0 Hz, 2H), 2.32 (s, 3H), 2.19 (s, 3H), 1.83 – 1.67 (m, 2H), 1.66 – 1.51 (m, 3H), 1.39 – 1.21 (m, 6H), 0.88 (s, 6H) ppm;

^{13}**C NMR** (101 MHz, $CDCl_3$): δ_C 157.3, 143.0, 136.6, 130.4, 128.5, 128.4, 125.7, 123.7, 120.7, 112.1, 68.8, 41.8, 38.1, 36.2, 32.7, 27.4, 24.5, 23.9, 21.6, 16.0 ppm.

IR (film): ν_{max} 3026, 2930, 2858, 1586, 1509, 1454, 1130, 1038, 802, 698 cm^{-1}.

HRMS (EI^+): calcd. for $C_{24}H_{34}O$ $[M]^+$, 338.2604, found 338.2598.

2-((9-Bromo-4,4-dimethylnonyl)oxy)-1,4-dimethylbenzene (11)

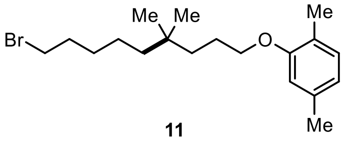

11

Prepared following **General Procedure A**, using (5-bromopentyl)boronic acid (39 mg, 0.20 mmol, 1.0 equiv.), 1,3-dioxoisoindolin-2-yl 5-(2,5-dimethylphenoxy)-2,2-dimethylpentanoate **R11** (158 mg, 0.400 mmol, 2.00 equiv.), $[Ir(dF(Me)ppy)_2(dtbbpy)]PF_6$ (4.0 mg, 4.0 μmol, 2.0 mol%), $Ni(acac)_2$ (10 mg, 0.040 mmol, 20 mol%), catechol (22 mg, 0.20 mmol, 1.0 equiv), 3-quinuclidinol (25 mg, 0.20 mmol, 1.0 equiv.) in anhydrous DMSO (0.60 mL, *c* = 0.33 M). Biotage Isolera™ flash purification on silica gel (SNAP 5 g silica cartridge) eluting with

pentane (100%), followed by preparative thin-layer chromatography purification with pentane (100%), gave **11** (28 mg, 39%) as a colorless oil.

R*f* = 0.3 (pentane, UV, $KMnO_4$)

NMR Spectroscopy (*see spectra*):

> **^{1}H NMR** (400 MHz, $CDCl_3$): δ_H 7.05 – 6.97 (m, 1H), 6.69 – 6.59 (m, 2H), 3.91 (t, *J* = 6.5 Hz, 2H), 3.41 (t, *J* = 6.9 Hz, 2H), 2.31 (s, 3H), 2.18 (s, 3H), 1.93 – 1.82 (m, 2H), 1.78 – 1.68 (m, 2H), 1.45 – 1.31 (m, 4H), 1.30 – 1.19 (m, 4H), 0.88 (s, 6H) ppm;

> **^{13}C NMR** (101 MHz, $CDCl_3$): δ_C 157.2, 136.6, 130.4, 123.7, 120.7, 112.1, 68.7, 41.8, 38.1, 34.2, 33.0, 32.6, 29.3, 27.4, 24.4, 23.4, 21.6, 16.0 ppm.

IR (film) ν_{max}: 2921, 2850, 1465, 1130 cm^{-1}.

HRMS (EI$^+$): calcd. for $C_{19}H_{31}OBr$ [M]$^+$, 354.1553, found 354.1547.

1-Chloro-4-((2-methyl-6-phenylhexan-2-yl)oxy)benzene (12)

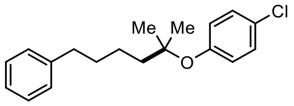

12

Prepared following **General Procedure A**, using (4-phenylbutyl)boronic acid (36 mg, 0.20 mmol, 1.0 equiv.), 1,3-dioxoisoindolin-2-yl 2-(4-chlorophenoxy)-2-methylpropanoate **R12** (144 mg, 0.400 mmol, 2.00 equiv.), [Ir(*d*F(Me)ppy)$_2$(*d*tbbpy)]PF_6 (4.0 mg, 4.0 μmol, 2.0 mol%), Ni(acac)$_2$ (10 mg, 0.040 mmol, 20 mol%), catechol (22 mg, 0.20 mmol, 1.0 equiv), 3-quinuclidinol (25 mg, 0.20 mmol, 1.0 equiv.) in anhydrous DMSO (0.60 mL, *c* = 0.33 M). Biotage Isolera™ flash purification on silica gel (SNAP 5 g silica cartridge), eluting with EtOAc/pentane (0 – 5%, v/v), gave **12** (30 mg, 50%) as a colorless oil.

R*f* = 0.7 (1:20 EtOAc/pentane, UV, $KMnO_4$)

NMR Spectroscopy (*see spectra*):

> **^{1}H NMR** (400 MHz, $CDCl_3$): δ_H 7.34 – 7.14 (m, 7H), 6.93 – 6.83 (m, 2H), 2.65 (t, *J* = 7.7 Hz, 2H), 1.73 – 1.59 (m, 4H), 1.59 – 1.45 (m, 2H), 1.25 (s, 6H) ppm;

> **^{13}C NMR** (101 MHz, $CDCl_3$): δ_C 154.1, 142.7, 129.0, 128.5, 128.4, 125.8, 125.7, 125.4, 81.2, 42.0, 36.1, 32.1, 26.7, 24.1 ppm.

IR (film): ν_{max} 2975, 2935, 2857, 1486, 1367, 1238, 1091, 892, 849, 699 cm^{-1}.

HRMS (APCI$^+$): calcd. for $C_{19}H_{23}OCl$ [M+H]$^+$, 302.1432, found 302.1427.

4-(4-Phenylbutyl)tetrahydro-2*H*-pyran (13)

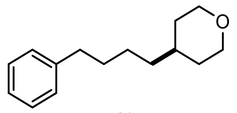

13

Prepared following **General Procedure A**, using (4-phenylbutyl)boronic acid (36 mg, 0.20 mmol, 1.0 equiv.), 1,3-dioxoisoindolin-2-yl tetrahydro-2H-pyran-4-carboxylate **R13** (110 mg, 0.400 mmol, 2.00 equiv.), [Ir(*d*F(Me)ppy)$_2$(*d*tbbpy)]PF$_6$ (4.0 mg, 4.0 μmol, 2.0 mol%), Ni(acac)$_2$ (10 mg, 0.040 mmol, 20 mol%), catechol (22 mg, 0.20 mmol, 1.0 equiv), 3-quinuclidinol (25 mg, 0.20 mmol, 1.0 equiv.) in anhydrous DMSO (0.60 mL, *c* = 0.33 M). Biotage Isolera™ flash purification on silica gel (SNAP 5 g silica cartridge), eluting with EtOAc/pentane (0 – 5%, v/v), gave **13** (28 mg, 64%) as a colorless oil.

R*f* = 0.6 (1:20 EtOAc/pentane, UV, KMnO$_4$)

NMR Spectroscopy (*see spectra*):

^{1}H NMR (400 MHz, CDCl$_3$): δ$_H$ 7.31 – 7.24 (m, 2H), 7.23 – 7.13 (m, 3H), 3.99 – 3.89 (m, 2H), 3.35 (td, *J* = 11.7, 2.1 Hz, 2H), 2.61 (t, *J* = 7.6 Hz, 2H), 1.68 – 1.52 (m, 4H), 1.54 – 1.38 (m, 1H), 1.40 – 1.17 (m, 6H) ppm;

^{13}C NMR (101 MHz, CDCl$_3$): δ$_C$ 142.9, 128.5, 128.4, 125.8, 68.3, 36.9, 36.1, 35.1, 33.4, 31.8, 26.2 ppm.

IR (film): ν_{max} 3026, 2925, 2852, 1454, 1095, 981, 747, 698 cm^{-1}.

HRMS (EI$^+$): calcd. for $C_{15}H_{22}O$ [M]$^+$, 218.1665, found 218.1662.

(4-(4,4-Difluorocyclohexyl)butyl)benzene (14)

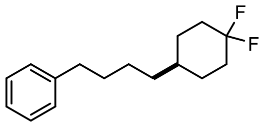

14

Prepared following **General Procedure A**, using (4-phenylbutyl)boronic acid (36 mg, 0.20 mmol, 1.0 equiv.), 1,3-dioxoisoindolin-2-yl 4,4-difluorocyclohexane-1-carboxylate **R14** (124 mg, 0.400 mmol, 2.00 equiv.), [Ir(*d*F(Me)ppy)$_2$(*d*tbbpy)]PF$_6$ (4.0 mg, 4.0 μmol, 2.0 mol%), Ni(acac)$_2$ (10 mg, 0.040 mmol, 20 mol%), catechol (22 mg, 0.20 mmol, 1.0 equiv), 3-quinuclidinol (25 mg, 0.20 mmol, 1.0 equiv.) in anhydrous DMSO (0.60 mL, *c* = 0.33 M). Biotage Isolera™ flash purification on silica gel (SNAP 5 g silica cartridge), eluting with EtOAc/pentane (0 – 5%, v/v), gave **14** (33 mg, 65%) as a colorless oil.

R*f* = 0.7 (1:5 EtOAc/pentane, UV, KMnO$_4$)

NMR Spectroscopy (*see spectra*):

^{1}H NMR (400 MHz, CDCl$_3$): δ$_H$ 7.31 – 7.24 (m, 2H), 7.21 – 7.15 (m, 3H), 2.61 (t, *J* = 7.7 Hz, 2H), 2.12 – 1.99 (m, 2H), 1.80 – 1.54 (m, 6H), 1.41 – 1.16 (m, 7H) ppm;

^{13}C NMR (101 MHz, CDCl3): δ$_C$ 142.8, 128.5, 128.4, 125.8, 36.0, 35.8 (d, *J* = 1.6 Hz), 35.7 (d, *J* = 2.6 Hz),

33.55 (dd, J = 25.5, 22.1 Hz), 31.8, 29.1 (d, J = 2.6 Hz), 26.9 ppm;

^{19}F NMR (377 MHz, $CDCl_3$): δ_F –91.2 (d, J = 233.9 Hz), –101.8 (d, J = 234.6 Hz) ppm.

IR (film): ν_{max} 2929, 2856, 1379, 1358, 1183, 1114, 960, 744, 698, 498 cm^{-1}.

HRMS (EI$^+$): calcd. for $C_{16}H_{22}F_2$ [M]$^+$, 252.1684, found 252.1682.

tert-Butyl 4-(5-(benzoyloxy)pentyl)piperidine-1-carboxylate (15)

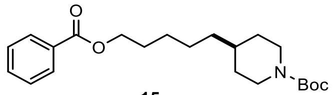

15

Prepared following modified **General Procedure A**, using (5-(benzoyloxy)pentyl)boronic acid (47 mg, 0.20 mmol, 1.0 equiv.), 1-(*tert*-butyl) 4-(1,3-dioxoisoindolin-2-yl) piperidine-1,4-dicarboxylate **R15** (150 mg, 0.400 mmol, 2.00 equiv.), [Ir(*p*-F(Me)ppy)$_2$-(4,4'-*d*tbbpy)]PF_6 (4.0 mg, 4.0 μmol, 2.0 mol%), Ni(acac)$_2$ (10 mg, 0.040 mmol, 20 mol%), catechol (22 mg, 0.20 mmol, 1.0 equiv), 3-quinuclidinol (25 mg, 0.20 mmol, 1.0 equiv.) in anhydrous DMSO (2.0 mL, c = 0.10 M). Biotage Isolera™ flash purification on silica gel (SNAP 5 g silica cartridge), eluting with EtOAc/pentane (0 – 20%, v/v), gave **15** (44 mg, 61%) as a colorless oil.

R*f* = 0.2 (1:10 EtOAc/pentane, UV, $KMnO_4$)

NMR Spectroscopy (*see spectra*):

^{1}H NMR (400 MHz, $CDCl_3$): δ_H 8.06 – 8.01 (m, 2H), 7.58 – 7.51 (m, 1H), 7.47 – 7.40 (m, 2H), 4.31 (t, J = 6.6 Hz, 2H), 4.06 (d, J = 13.2 Hz, 2H), 2.65 (t, J = 12.9 Hz, 2H), 1.81 – 1.71 (m, 2H), 1.68 – 1.59 (m, 2H), 1.49 – 1.31 (m, 5H), 1.45 (s, 9H), 1.29 – 1.20 (m, 2H), 1.13 – 1.00 (m, 2H) ppm;

^{13}C NMR (101 MHz, $CDCl_3$): δ_C 166.8, 155.0, 133.0, 130.6, 129.7, 128.5, 79.3, 65.1, 44.2, 36.5, 36.1, 32.3, 28.8, 28.6, 26.4, 26.3 ppm.

IR (film): ν_{max} 2928, 2854, 1719, 1689, 1421, 1271, 1164, 1111, 711 cm^{-1}.

HRMS (ESI$^+$): calcd. for $C_{22}H_{33}O_4NNa$ [M+Na]$^+$, 398.2302, found 398.2320.

2-(4-Phenylbutyl)tetrahydrofuran (16)

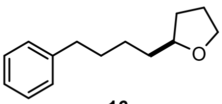

16

Prepared following **General Procedure A**, using (4-phenylbutyl)boronic acid (36 mg, 0.20 mmol, 1.0 equiv.), 1,3-dioxoisoindolin-2-yl tetrahydrofuran-2-carboxylate **R16** (124 mg, 0.400 mmol, 2.00 equiv.), [Ir(*d*F(Me)ppy)$_2$(*d*tbbpy)]PF_6 (4.0 mg, 4.0 μmol, 2.0 mol%), Ni(acac)$_2$ (10 mg, 0.040 mmol, 20 mol%), catechol (22 mg, 0.20 mmol, 1.0 equiv), 3-quinuclidinol (25 mg, 0.20 mmol, 1.0 equiv.) in anhydrous DMSO (0.60 mL, c = 0.33 M). Biotage Isolera™ flash purification on silica gel (SNAP 5 g silica cartridge), eluting with EtOAc/pentane (0 – 10%, v/v), gave **16** (16 mg, 39%) as a colorless oil.

R*f* = 0.9 (1:5 EtOAc/pentane, UV, $KMnO_4$)

NMR Spectroscopy (see spectra):

^{1}**H NMR** (400 MHz, $CDCl_3$): δ_H 7.32 – 7.22 (m, 2H), 7.22 – 7.12 (m, 3H), 3.92 – 3.65 (m, 3H), 2.67 – 2.57 (m, 2H), 2.03 – 1.76 (m, 3H), 1.71 – 1.59 (m, 3H), 1.55 – 1.30 (m, 4H) ppm;

^{13}**C NMR** (101 MHz, $CDCl_3$): δ_C 142.9, 128.5, 128.4, 125.8, 79.5, 67.8, 36.1, 35.8, 31.8, 31.5, 26.3, 25.9 ppm.

All recorded spectroscopic data matched those previously reported in the literature.[2]

(*Cis* and *trans*) 5-(4-hydroxycyclohexyl)pentyl benzoate (17)

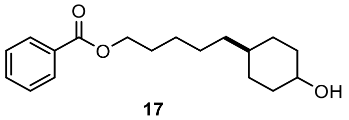

17
cis : trans = 3 : 2

Prepared following modified **General Procedure A**, using (5-(benzoyloxy)pentyl)boronic acid (47 mg, 0.20 mmol, 1.0 equiv.), 1,3-dioxoisoindolin-2-yl 4-hydroxycyclohexane-1-carboxylate **R17** (116 mg, 0.400 mmol, 2.00 equiv.), [Ir(*d*F(Me)ppy)$_2$(*d*tbbpy)]PF_6 (4.0 mg, 4.0 µmol, 2.0 mol%), Ni(acac)$_2$ (10 mg, 0.040 mmol, 20 mol%), catechol (22 mg, 0.20 mmol, 1.0 equiv), 3-quinuclidinol (25 mg, 0.20 mmol, 1.0 equiv.) in anhydrous DMSO (2.0 mL, *c* = 0.10 M). Biotage Isolera™ flash purification on silica gel (SNAP 5 g silica cartridge), eluting with EtOAc/pentane (0 – 20%, v/v), gave **17** (19 + 28 mg, 56%, *cis* : *trans* = 2:3) as a colorless oil.

R_f (18-*trans*) = 0.6 (1:5 EtOAc/pentane, UV, $KMnO_4$)

NMR Spectroscopy of 17-*trans* (see spectra):

^{1}**H NMR** (400 MHz, $CDCl_3$): δ_H 8.09 – 8.00 (m, 2H), 7.57 – 7.48 (m, 1H), 7.48 – 7.40 (m, 2H), 4.32 (t, *J* = 6.7 Hz, 2H), 4.00 – 3.90 (m, 1H), 1.84 – 1.63 (m, 4H), 1.60 – 1.20 (m, 14H) ppm;

^{13}**C NMR** (101 MHz, $CDCl_3$): δ_C 166.9, 132.9, 130.7, 129.7, 128.5, 67.4, 65.2, 36.3, 36.0, 32.4, 28.9, 27.2, 26.8, 26.4 ppm.

IR (film): ν_{max} 2924, 2853, 1720, 1275, 1112, 711 cm^{-1}.

HRMS (ESI$^+$): calcd. for $C_{18}H_{26}O_3Na$ [M+Na]$^+$, 313.1780, found 313.1773.

R_f (18-*cis*) = 0.5 (1:5 EtOAc/pentane, UV, $KMnO_4$)

NMR Spectroscopy of 17-*cis* (see spectra):

^{1}**H NMR** (500 MHz, $CDCl_3$): δ_H 8.08 – 8.01 (m, 2H), 7.58 – 7.53 (m, 1H), 7.46 – 7.41 (m, 2H), 4.31 (t, *J* = 6.7 Hz, 2H), 3.55 – 3.51 (m, 1H), 2.00 – 1.92 (m, 2H), 1.82 – 1.72 (m, 4H), 1.47 – 1.31 (m, 5H), 1.29 – 1.14 (m, 5H), 1.00 – 0.88 (m, 2H) ppm;

^{13}**C NMR** (126 MHz, $CDCl_3$): δ_C 166.8, 133.0, 130.7, 129.8, 129.7, 129.5, 128.6, 128.5, 71.3, 65.2, 36.8, 36.7, 35.8, 31.4, 28.9, 27.0, 26.4 ppm.

IR (film): ν_{max} 3370, 2926, 2855, 1720, 1452, 1274, 1114, 711 cm^{-1}.

HRMS (EI$^+$): calcd. for $C_{18}H_{26}O_3$ [M]$^+$, 290.1876, found 290.1872.

Benzyl-2-(4-phenylbutyl)pyrrolidine-1-carboxylate (18)

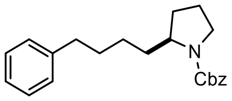

18

Prepared following modified **General Procedure A**, using (4-phenylbutyl)boronic acid (36 mg, 0.20 mmol, 1.0 equiv.), 1-benzyl 2-(1,3-dioxoisoindolin-2-yl) pyrrolidine-1,2-dicarboxylate **R18** (158 mg, 0.400 mmol, 2.00 equiv.), [Ir(*p*-F(Me)ppy)$_2$-(4,4'-*d*tbbpy)]PF$_6$ (4.0 mg, 4.0 μmol, 2.0 mol%), Ni(acac)$_2$ (21 mg, 0.080 mmol, 40 mol%), catechol (22 mg, 0.20 mmol, 1.0 equiv), 3-quinuclidinol (25 mg, 0.20 mmol, 1.0 equiv.) in anhydrous DMSO (2.0 mL, *c* = 0.10 M). Biotage Isolera^{TM} flash purification on silica gel (SNAP 5 g silica cartridge), eluting with EtOAc/pentane (0 – 20%, v/v), gave **18** (47 mg, 70%) as a colorless oil.

R*f* = 0.30 (1:5 EtOAc/pentane, UV, KMnO$_4$)

NMR Spectroscopy (*see spectra*):

^{1}**H NMR** (400 MHz, CDCl$_3$): δ$_H$ 7.41 – 7.22 (m, 7H), 7.21 – 7.08 (m, 3H), 5.22 – 5.06 (m, 2H), 3.91 – 3.76 (m, 1H), 3.53 – 3.34 (m, 2H), 2.68 – 2.48 (m, 2H), 1.97 – 1.76 (m, 3H), 1.74 – 1.52 (m, 4H), 1.42 – 1.21 (m, 3H) ppm;

^{13}**C NMR** (126 MHz, CDCl$_3$): δ$_C$ 155.2+155.0 (amide rotamers), 142.8+142.7 (amide rotamers), 137.3+137.3 (amide rotamers), 128.6, 128.5, 128.4, 127.9, 125.7, 66.8+66.5 (amide rotamers), 58.1+57.3 (amide rotamers), 46.7, 46.4 (amide rotamers), 36.0+35.9 (amide rotamers), 34.5+33.8 (amide rotamers), 31.6+31.4 (amide rotamers), 30.7+29.9 (amide rotamers), 26.1+25.9 (amide rotamers), 23.9+23.2(amide rotamers) ppm.

IR (film): v_{max} 2927, 2857, 1701, 1411, 1357, 1100, 698 cm^{-1}.

HRMS (EI$^+$): calcd. for $C_{22}H_{27}O_2N$ [M]$^+$, 337.2036, found 337.2031.

Benzyl-(1-(methylthio)-7-phenylheptan-3-yl)carbamate (19)

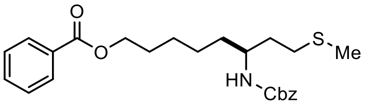

19

Prepared following modified **General Procedure A**, using (5-(benzoyloxy)pentyl)boronic acid (47 mg, 0.20 mmol, 1.0 equiv.), 1,3-dioxoisoindolin-2-yl ((benzyloxy)carbonyl)-*L*-methioninate **R19** (171 mg, 0.400 mmol, 2.00 equiv.), [Ir(*p*-F(Me)ppy)$_2$-(4,4'-*d*tbbpy)]PF$_6$ (4.0 mg, 4.0 μmol, 2.0 mol%), Ni(acac)$_2$ (10 mg, 0.040 mmol, 20 mol%), catechol (22 mg, 0.20 mmol, 1.0 equiv), 3-quinuclidinol (25 mg, 0.20 mmol, 1.0 equiv.) in anhydrous DMSO (2.0 mL, *c* = 0.10 M). Biotage Isolera^{TM} flash purification on silica gel (SNAP 5 g silica cartridge), eluting with EtOAc/pentane (0 – 30%, v/v), gave **19** (41 mg, 48%) as a white solid.

R*f* = 0.6 (1:10 EtOAc/pentane, UV, KMnO$_4$)

NMR Spectroscopy (*see spectra*):

^{1}H NMR (400 MHz, $CDCl_3$): δ_H 7.98 – 7.93 (m, 2H), 7.50 – 7.44 (m, 1H), 7.38 – 7.31 (m, 2H), 7.29 – 7.19 (m, 5H), 5.00 (s, 2H), 4.48 (d, *J* = 9.3 Hz, 1H), 4.21 (t, *J* = 6.6 Hz, 2H), 3.72 – 3.58 (m, 1H), 2.48 – 2.38 (m, 2H), 2.00 (s, 3H), 1.77 – 1.49 (m, 5H), 1.61 – 1.30 (m, 5H) ppm;

^{13}C NMR (101 MHz, $CDCl_3$): δ_C 166.7, 156.1, 136.6, 132.9, 130.5, 129.6, 128.6, 128.4, 128.2, 128.1, 66.7, 64.9, 50.8, 35.3, 35.0, 30.7, 28.7, 26.0, 25.6, 15.7 ppm.

IR (film): ν_{max} 3339, 2920, 2854, 1718, 1528, 1276, 1115, 713 cm^{-1}.

HRMS (ESI$^+$): calcd. for $C_{24}H_{32}O_4NS$ [M+H]$^+$, 430.2047, found 430.2044.

Benzyl-(2-methyl-7-phenylheptan-3-yl)carbamate (20)

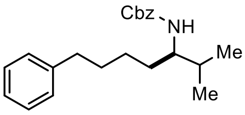

20

Prepared following modified **General Procedure A**, using (4-phenylbutyl)boronic acid (36 mg, 0.20 mmol, 1.0 equiv.), 1,3-dioxoisoindolin-2-yl ((benzyloxy)carbonyl)-*L*-valinate **R20** (158 mg, 0.400 mmol, 2.00 equiv.), [Ir(*p*-F(Me)ppy)$_2$-(4,4'-*d*tbbpy)]PF_6 (4.0 mg, 4.0 μmol, 2.0 mol%), Ni(acac)$_2$ (21 mg, 0.080 mmol, 40 mol%), catechol (22 mg, 0.20 mmol, 1.0 equiv), 3-quinuclidinol (25 mg, 0.20 mmol, 1.0 equiv.) in anhydrous DMSO (2.0 mL, *c* = 0.10 M). Biotage Isolera™ flash purification on silica gel (SNAP 5 g silica cartridge) eluting with EtOAc/pentane (0 – 20%, v/v), followed by preparative thin-layer chromatography purification with EtOAc/pentane (10%, v/v), gave **20** (50 mg, 65%) as a colorless oil.

R*f* = 0.4 (1:3 EtOAc/pentane, UV, $KMnO_4$)

NMR Spectroscopy (*see spectra*):

^{1}H NMR (600 MHz, $CDCl_3$): δ_H 7.47 – 7.38 (m, 6H), 7.37 – 7.31 (m, 2H), 7.27 – 7.22 (m, 2H), 5.23 – 5.15 (m, 2H), 4.56 (d, *J* = 9.8 Hz, 1H), 3.62 – 3.49 (m, 1H), 2.67 (t, *J* = 7.8 Hz, 2H), 1.84 – 1.66 (m, 3H), 1.62 – 1.55 (m, 1H), 1.54 – 1.33 (m, 3H), 0.96 (dd, *J* = 25.2, 6.8 Hz, 6H) ppm;

^{13}C NMR (151 MHz, $CDCl_3$): δ_C 156.6, 142.7, 136.8, 136.3, 128.7, 128.6, 128.51, 128.47, 128.4, 128.2, 125.8, 117.8, 113.3, 67.2, 56.3, 36.0, 32.5 + 32.1 (amide rotamers), 31.5, 26.0, 22.3, 19.3, 17.6 ppm.

IR (film): ν_{max} 3332, 2931, 2857, 1702, 1497, 1234, 1028, 748, 698 cm^{-1}.

HRMS (ESI$^+$): calcd. for $C_{22}H_{30}O_2N$ [M+H]$^+$, 340.2271, found 340.2268.

***tert*-Butyl-(1-cyclohexyl-5-phenylpentyl)carbamate (21)**

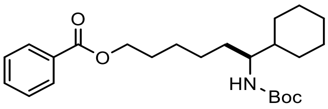

21

Prepared following modified **General Procedure A**, using (5-(benzoyloxy)pentyl)boronic acid (47 mg, 0.20 mmol, 1.0 equiv.), 1,3-dioxoisoindolin-2-yl 2-((*tert*-butoxycarbonyl)amino)-2-cyclohexylacetate **R21** (161 mg,

0.400 mmol, 2.00 equiv.), [Ir(*p*-F(Me)ppy)$_2$-(4,4'-*d*tbbpy)]PF$_6$ (4.0 mg, 4.0 µmol, 2.0 mol%), Ni(acac)$_2$ (10 mg, 0.040 mmol, 20 mol%), catechol (22 mg, 0.20 mmol, 1.0 equiv), 3-quinuclidinol (25 mg, 0.20 mmol, 1.0 equiv.) in anhydrous DMSO (2.0 mL, *c* = 0.10 M). Biotage Isolera™ flash purification on silica gel (SNAP 5 g silica cartridge), eluting with EtOAc/pentane (0 – 30%, v/v), gave **21** (61 mg, 76%) as a white grease.

R*f* = 0.5 (1:5 EtOAc/pentane, UV, $KMnO_4$)

NMR Spectroscopy (*see spectra*):

^{1}**H NMR** (400 MHz, $CDCl_3$): δ_H 8.08 – 7.99 (m, 2H), 7.59 – 7.49 (m, 1H), 7.47 – 7.38 (m, 2H), 4.34 – 4.24 (m, 3H), 3.48 – 3.35 (m, 1H), 1.80 – 1.60 (m, 8H), 1.43 (s, 9H), 1.48 – 0.97 (m, 10H) ppm;

^{13}**C NMR** (101 MHz, $CDCl_3$): δ_C 166.8, 156.1, 132.9, 130.6, 129.7, 128.4, 78.9, 65.1, 55.1, 42.5, 32.6, 29.8, 28.9, 28.6, 28.3, 26.6, 26.4, 26.2, 26.0 ppm.

IR (film): ν_{max} 3364, 2925, 2853, 1719, 1520, 1451, 1274, 1173, 1114, 712 cm^{-1}.

HRMS (ESI$^+$): calcd. for $C_{24}H_{38}O_4N$ [M+H]$^+$, 404.2795, found 404.2791.

Benzyl (2-methyl-6-phenylhexan-2-yl)carbamate (22)

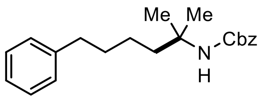

22

Prepared following modified **General Procedure A**, using (4-phenylbutyl)boronic acid (36 mg, 0.20 mmol, 1.0 equiv.), 1,3-dioxoisoindolin-2-yl 2-(((benzyloxy)carbonyl)amino)-2-methylpropanoate **R22** (153 mg, 0.400 mmol, 2.00 equiv.), [Ir(*p*-F(Me)ppy)$_2$-(4,4'-*d*tbbpy)]PF$_6$ (4.0 mg, 4.0 µmol, 2.0 mol%), Ni(acac)$_2$ (21 mg, 0.080 mmol, 40 mol%), catechol (22 mg, 0.20 mmol, 1.0 equiv), 3-quinuclidinol (25 mg, 0.20 mmol, 1.0 equiv.) in anhydrous DMSO (2.0 mL, *c* = 0.10 M). Biotage Isolera™ flash purification on silica gel (SNAP 5 g silica cartridge), eluting with EtOAc/pentane (0 – 30%, v/v), gave **22** (37 mg, 57%) as a colorless oil.

R*f* = 0.40 (1:4 EtOAc/pentane, UV, $KMnO_4$)

NMR Spectroscopy (*see spectra*):

^{1}**H NMR** (400 MHz, $CDCl_3$): δ_H 7.42 – 7.23 (m, 7H), 7.21 – 7.14 (m, 3H), 5.05 (s, 2H), 4.63 (s, 1H), 2.61 (t, *J* = 7.8 Hz, 2H), 1.73 – 1.55 (m, 4H), 1.39 – 1.25 (m, 2H), 1.28 (s, 6H).

^{13}**C NMR** (101 MHz, $CDCl_3$): δ_C 154.7, 142.7, 136.9, 128.7, 128.5, 128.4, 128.2, 128.1, 125.8, 66.1, 53.0, 40.5, 36.0, 31.9, 27.2, 23.9 ppm.

IR (film): ν_{max} 3350, 2930, 2859, 1726, 1498, 1454, 1259, 1072, 749, 697 cm^{-1}.

HRMS (ESI$^+$): calcd. for $C_{21}H_{28}O_2N$ [M+H]$^+$, 326.2115, found 326.2117.

tert-Butyl-4-(5-(benzoyloxy)pentyl)thiazolidine-3-carboxylate (23)

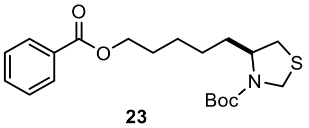

23

Prepared following modified **General Procedure A**, using (5-(benzoyloxy)pentyl)boronic acid (47 mg, 0.20 mmol, 1.0 equiv.), 3-(*tert*-butyl) 4-(1,3-dioxoisoindolin-2-yl) thiazolidine-3,4-dicarboxylate **R23** (151 mg, 0.400 mmol, 2.00 equiv.), [Ir(*p*-F(Me)ppy)$_2$-(4,4'-*d*tbbpy)]PF$_6$ (4.0 mg, 4.0 µmol, 2.0 mol%), Ni(acac)$_2$ (10 mg, 0.040 mmol, 20 mol%), catechol (22 mg, 0.20 mmol, 1.0 equiv), 3-quinuclidinol (25 mg, 0.20 mmol, 1.0 equiv.) in anhydrous DMSO (2.0 mL, *c* = 0.10 M). Biotage Isolera™ flash purification on silica gel (SNAP 5 g silica cartridge), eluting with EtOAc/pentane (0 – 30%, v/v), gave **23** (48 mg, 63%) as a colorless oil.

R*f* = 0.5 (1:5 EtOAc/pentane, UV, $KMnO_4$)

NMR Spectroscopy (*see spectra*):

^{1}**H NMR** (500 MHz, $CDCl_3$): δ_H 8.10 – 7.99 (m, 2H), 7.60 – 7.55 (m, 1H), 7.52 – 7.40 (m, 2H), 4.69 (s, 1H), 4.34 (t, *J* = 6.6 Hz, 3H), 4.19 (d, *J* = 9.1 Hz, 1H), 3.16 – 3.07 (m, 1H), 2.74 (dd, *J* = 11.1, 2.4 Hz, 1H), 1.84 – 1.68 (m, 3H), 1.64 – 1.54 (m, 1H), 1.53 – 1.49 (m, 2H), 1.48 (s, 9H), 1.44 – 1.39 (m, 2H) ppm;

^{13}**C NMR** (126 MHz, $CDCl_3$): δ_C 166.8, 153.6, 133.0, 130.6, 129.7, 128.5, 80.5, 65.0, 60.0, 47.5, 33.26, 28.9, 28.5, 28.5, 26.2, 26.1 ppm.

IR (film): ν_{max} 2933, 2860, 1717, 1692, 1382, 1366, 1271, 1110, 871, 711 cm^{-1}.

HRMS (ESI$^+$): calcd. for $C_{20}H_{29}O_4NSNa$ [M+Na]$^+$, 402.1710, found 402.1701.

6-(4-Isobutylphenyl)heptyl benzoate (24)

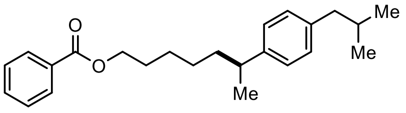

24

Prepared following **General Procedure A**, using (5-(benzoyloxy)pentyl)boronic acid (47 mg, 0.20 mmol, 1.0 equiv.), 1,3-dioxoisoindolin-2-yl 2-(4-isobutylphenyl)propanoate **R24** (141 mg, 0.400 mmol, 2.00 equiv.), [Ir(*d*F(Me)ppy)$_2$(*d*tbbpy)]PF$_6$ (4.0 mg, 4.0 µmol, 2.0 mol%), Ni(acac)$_2$ (10 mg, 0.040 mmol, 20 mol%), catechol (22 mg, 0.20 mmol, 1.0 equiv), 3-quinuclidinol (25 mg, 0.20 mmol, 1.0 equiv.) in anhydrous DMSO (0.60 mL, *c* = 0.33 M). Biotage Isolera™ flash purification on silica gel (SNAP 5 g silica cartridge), eluting with EtOAc/pentane (0 – 10%, v/v), gave **24** (38 mg, 54%) as a colorless oil.

R*f* = 0.2 (1:20 EtOAc/pentane, UV, $KMnO_4$)

NMR Spectroscopy (*see spectra*):

^{1}**H NMR** (400 MHz, $CDCl_3$): δ_H 8.07 – 7.99 (m, 2H), 7.61 – 7.50 (m, 1H), 7.48 – 7.39 (m, 2H), 7.12 – 7.01 (m, 4H), 4.28 (t, *J* = 6.7 Hz, 2H), 2.73 – 2.59 (m, 1H), 2.43 (d, *J* = 7.2 Hz, 2H), 1.92 – 1.67 (m, 3H), 1.63 – 1.51 (m, 2H), 1.48 – 1.25 (m, 4H), 1.23 (d, *J* = 7.0 Hz, 3H), 0.90 (d, *J* = 6.6 Hz, 6H) ppm;

^{13}C NMR (101 MHz, $CDCl_3$): δ_C 166.8, 145.0, 139.2, 132.9, 130.7, 129.7, 129.1, 128.5, 126.7, 65.2, 45.2, 39.6, 38.5, 30.4, 28.8, 27.6, 26.3, 22.6, 22.5 ppm.

IR (film): ν_{max} 2954, 2926, 2868, 1720, 1452, 1272, 1112, 710 cm^{-1}.

HRMS (EI$^+$): calcd. for $C_{24}H_{32}O_2$ [M]$^+$, 352.2397, found 352.2390.

6-(2-Fluoro-[1,1'-biphenyl]-4-yl)heptyl benzoate (25)

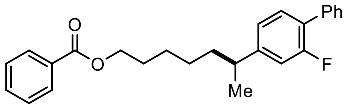

25

Prepared following **General Procedure A**, using (5-(benzoyloxy)pentyl)boronic acid (47 mg, 0.20 mmol, 1.0 equiv.), 1,3-dioxoisoindolin-2-yl 2-(2-fluoro-[1,1'-biphenyl]-4-yl)propanoate **R25** (156 mg, 0.400 mmol, 2.00 equiv.), [Ir(*d*F(Me)ppy)$_2$(*d*tbbpy)]PF_6 (4.0 mg, 4.0 μmol, 2.0 mol%), Ni(acac)$_2$ (10 mg, 0.040 mmol, 20 mol%), catechol (22 mg, 0.20 mmol, 1.0 equiv), 3-quinuclidinol (25 mg, 0.20 mmol, 1.0 equiv.) in anhydrous DMSO (0.60 mL, *c* = 0.33 M). Biotage Isolera™ flash purification on silica gel (SNAP 5 g silica cartridge) eluting with EtOAc/pentane (0 – 10%, v/v), followed by preparative thin-layer chromatography purification with EtOAc/pentane (10%, v/v), gave **25** (24 mg, 31%) as a colorless oil.

R*f* = 0.3 (1:10 EtOAc/pentane, UV, $KMnO_4$)

NMR Spectroscopy (*see spectra*):

^{1}H NMR (500 MHz, $CDCl_3$): δ_H 8.09 – 8.02 (m, 2H), 7.61 – 7.54 (m, 3H), 7.49 – 7.43 (m, 4H), 7.39 – 7.34 (m, 2H), 7.05 (dd, *J* = 7.9, 1.7 Hz, 1H), 7.00 (dd, *J* = 12.1, 1.7 Hz, 1H), 4.32 (t, *J* = 6.6 Hz, 2H), 2.75 (h, *J* = 7.0 Hz, 1H), 1.82 – 1.72 (m, 2H), 1.71 – 1.61 (m, 2H), 1.53 – 1.42 (m, 2H), 1.42 – 1.31 (m, 2H), 1.29 (d, J = 6.9 Hz, 3H) ppm;

^{13}C NMR (126 MHz, $CDCl_3$): δ_C 166.8, 159.9 (d, *J* = 247.9 Hz), 149.6 (d, *J* = 6.4 Hz), 136.0 (d, *J* = 1.3 Hz), 132.9, 130.6 (d, *J* = 2.3 Hz), 130.5, 129.6, 129.1(d, *J* = 6.4 Hz), 128.5 (d, *J* = 2.9 Hz), 127.48, 126.4 (d, *J* = 13.6 Hz), 123.1 (d, *J* = 3.1 Hz), 114.5 (d, *J* = 22.6 Hz), 65.1, 39.6 (d, *J* = 1.4 Hz), 38.2, 28.8, 27.5, 26.3, 22.3 ppm;

^{19}F NMR (377 MHz, $CDCl_3$): δ_F –118.5 ppm (^{19}F NMR was obtained on Varian 400MHz NMR spectrometer).

IR (film): ν_{max} 3034, 2928, 2857, 1717, 1451, 1272, 1112, 767, 711, 698 cm^{-1}.

HRMS (MALDI$^+$): calcd. for $C_{26}H_{27}O_2FNa$ [M+Na]$^+$, 413.1887, found 413.1881.

6-(6-Methoxynaphthalen-2-yl)heptyl benzoate (26)

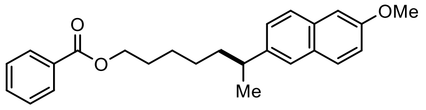

26

Prepared following **General Procedure A**, using (5-(benzoyloxy)pentyl)boronic acid (47 mg, 0.20 mmol, 1.0 equiv.), 1,3-dioxoisoindolin-2-yl 2-(4-isobutylphenyl)propanoate **R26** (150 mg, 0.400 mmol, 2.00 equiv.), [Ir(*d*F(Me)ppy)$_2$(*d*tbbpy)]PF$_6$(4.0 mg, 4.0 μmol, 2.0 mol%), Ni(acac)$_2$ (10 mg, 0.040 mmol, 20 mol%), catechol (22 mg, 0.20 mmol, 1.0 equiv), 3-quinuclidinol (25 mg, 0.20 mmol, 1.0 equiv.) in anhydrous DMSO (0.60 mL, *c* = 0.33 M). Biotage Isolera™ flash purification on silica gel (SNAP 5 g silica cartridge), eluting with EtOAc/pentane (0 – 10%, v/v), gave **26** (35 mg, 46%) as a colorless oil.

R*f* = 0.3 (1:10 EtOAc/pentane, UV, $KMnO_4$)

NMR Spectroscopy (*see spectra*):

^{1}H NMR (400 MHz, $CDCl_3$): δ_H 8.09 – 8.02 (m, 2H), 7.74 – 7.67 (m, 2H), 7.62 – 7.53 (m, 2H), 7.50 – 7.41 (m, 2H), 7.36 – 7.31 (m, 1H), 7.19 – 7.12 (m, 2H), 4.30 (t, *J* = 6.6 Hz, 2H), 3.94 (s, 3H), 2.91 – 2.78 (m, 1H), 1.80 – 1.63 (m, 4H), 1.34 (d, *J* = 7.3 Hz, 3H), 1.55 – 1.22 (m, 4H) ppm;

^{13}C NMR (101 MHz, $CDCl_3$): δ_C 166.8, 157.3, 142.9, 133.2, 132.9, 130.6, 129.6, 129.2, 129.1, 128.4, 126.9, 126.4, 125.1, 118.7, 105.8, 65.2, 55.4, 40.0, 38.4, 28.8, 27.6, 26.3, 22.6 ppm.

IR (film): ν_{max} 2929, 2856, 1717, 1606m 1452, 1271, 1114, 1028, 852, 711 cm^{-1}.

HRMS (MALDI$^+$): calcd. for $C_{25}H_{28}O_3Na$ [M+Na]$^+$, 399.1931, found 399.1937.

5-((1*S*,2*R*,6a*S*,6b*R*,8a*R*,10*S*,12a*R*,12b*R*,14b*R*)-10-hydroxy-1,2,6a,6b,9,9,12a-heptamethyl-1,3,4,5,6,6a,6b,7,8,8a,9,10,11,12,12a,12b,13,14b-octadecahydropicen-4a(2*H*)-yl)pentyl benzoate (27)

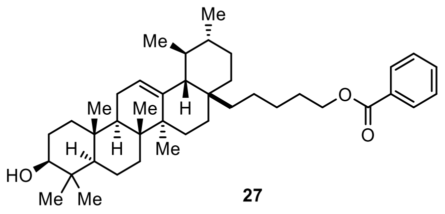

27

Prepared following modified **General Procedure A**, using (5-(benzoyloxy)pentyl)boronic acid (47 mg, 0.20 mmol, 1.0 equiv.), Ursolic phthalimide ester **R27** (241 mg, 0.400 mmol, 2.00 equiv.), [Ir(*p*-F(Me)ppy)$_2$-(4,4'-*d*tbbpy)]PF$_6$ (4.0 mg, 4.0 μmol, 2.0 mol%), Ni(acac)$_2$ (10 mg, 0.040 mmol, 20 mol%), catechol (22 mg, 0.20 mmol, 1.0 equiv), 3-quinuclidinol (25 mg, 0.20 mmol, 1.0 equiv.) in anhydrous DMSO (2.0 mL, *c* = 0.10 M). Biotage Isolera™ flash purification on silica gel (SNAP 5 g silica cartridge), eluting with EtOAc/pentane (0 – 30%, v/v), gave **27** (55 mg, 46%) as a white solid.

R*f* = 0.6 (1:5 EtOAc/pentane, UV, $KMnO_4$)

NMR Spectroscopy (*see spectra*):

^{1}H NMR (400 MHz, $CDCl_3$): δ_H 8.08 – 8.00 (m, 2H), 7.59 – 7.51 (m, 1H), 7.47 – 7.39 (m, 2H), 5.10 (t, *J* = 3.6 Hz, 1H), 4.30 (t, *J* = 6.6 Hz, 2H), 3.26 – 3.18 (m, 1H), 1.95 – 1.83 (m, 3H), 1.80 – 1.47 (m, 11H), 1.44 – 1.13 (m, 11H), 1.10 – 0.69 (m, 28H) ppm;

^{13}C NMR (101 MHz, $CDCl_3$): δ_C 166.8, 139.5, 132.9, 130.7, 129.7, 128.4, 124.8, 79.2, 65.2, 59.1, 55.3, 47.9, 42.1, 40.2, 40.1, 39.7, 39.6, 38.9, 37.6, 37.0, 35.8, 32.9, 31.2, 28.9, 28.3, 27.4, 27.1, 26.4, 24.7, 23.5, 22.0, 21.5, 18.4, 17.8, 16.9, 15.8, 15.7 ppm.

IR (film): ν_{max} 3426, 2923, 2854, 1720, 1452, 1272, 1113, 1027, 738, 710 cm^{-1}.

HRMS (MALDI$^+$): calcd. for $C_{41}H_{62}O_3Na$ $[M+Na]^+$, 625.4591, found 625.4597.

5-(1-Methylcyclohexyl)pentyl benzoate (28)

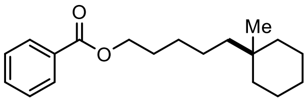

28

Prepared following **General Procedure A**, using (5-(benzoyloxy)pentyl)boronic acid (47 mg, 0.20 mmol, 1.0 equiv.), 1,3-dioxoisoindolin-2-yl 1-methylcyclohexane-1-carboxylate **2** (115 mg, 0.400 mmol, 2.00 equiv.), $[Ir(dF(Me)ppy)_2(dtbbpy)]PF_6$ (4.0 mg, 4.0 μmol, 2.0 mol%), $Ni(acac)_2$ (10 mg, 0.040 mmol, 20 mol%), catechol (22 mg, 0.20 mmol, 1.0 equiv), 3-quinuclidinol (25 mg, 0.20 mmol, 1.0 equiv.) in anhydrous DMSO (0.60 mL, *c* = 0.33 M). Biotage Isolera™ flash purification on silica gel (SNAP 5 g silica cartridge), eluting with EtOAc/pentane (0 – 5%, v/v), gave **28** (45 mg, 78%) as a colorless oil.

Prepared following **General Procedure B**, using pent-4-en-1-yl benzoate **5a** (38 mg, 0.20 mmol, 1.0 equiv.), HBcat (26.4 mg, 0.220 mmol, 1.10 equiv.), $Rh(PPh_3)_3Cl$ (2.0 mg, 2.0 μmol, 1.0 mol%) in anhydrous DCM (0.30 mL, *c* = 0.67 M) for hydroboration process. After 3 h stirring, DCM was removed and 1,3-dioxoisoindolin-2-yl 1-methylcyclohexane-1-carboxylate **2** (115 mg, 0.400 mmol, 2.00 equiv.), $[Ir(dF(Me)ppy)_2(dtbbpy)]PF_6$ (4.0 mg, 4.0 μmol, 2.0 mol%), $Ni(acac)_2$ (10 mg, 0.040 mmol, 20 mol%), 3-quinuclidinol (25 mg, 0.20 mmol, 1.0 equiv.) and anhydrous DMSO (0.60 mL, *c* = 0.33 M) were added for cross-coupling process. Biotage Isolera™ flash purification on silica gel (SNAP 5 g silica cartridge), eluting with pentane (100%), gave **28** (35 mg, 60%) as a colorless oil.

R_f = 0.4 (1:20 EtOAc/pentane, UV, $KMnO_4$)

NMR Spectroscopy (*see spectra*):

^{1}H NMR (400 MHz, $CDCl_3$): δ_H 8.09 – 8.01 (m, 2H), 7.61 – 7.50 (m, 1H), 7.48 – 7.40 (m, 2H), 4.32 (t, *J* = 6.7 Hz, 2H), 1.78 (p, *J* = 6.9 Hz, 2H), 1.48 – 1.16 (m, 16H), 0.84 (s, 3H) ppm;

^{13}C NMR (101 MHz, $CDCl_3$): δ_C 166.7, 132.8, 130.6, 129.6, 128.3, 65.2, 42.0, 37.9, 32.6, 28.9, 27.1, 26.6, 25.0, 23.0, 22.1 ppm.

IR (film): ν_{max} 2926, 2858, 1721, 1452, 1273, 1113, 750, 710 cm^{-1}.

HRMS (APCI$^+$): calcd. for $C_{19}H_{28}O_2Na$ $[M+Na]^+$, 311.1987, found 311.1993.

2-(4-(1-Methylcyclohexyl)butyl)isoindoline-1,3-dione (29)

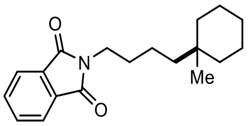

29

Prepared following **General Procedure B**, using 1-phthalimido-3-butene **29a** (26 mg, 0.20 mmol, 1.0 equiv.), HBcat (26.4 mg, 0.220 mmol, 1.10 equiv.), $Rh(PPh_3)_3Cl$ (2.0 mg, 2.0 μmol, 1.0 mol%) in anhydrous DCM (0.30 mL, c = 0.67 M) for hydroboration process. After 3 h stirring, DCM was removed and 1,3-dioxoisoindolin-2-yl 1-methylcyclohexane-1-carboxylate **2** (115 mg, 0.400 mmol, 2.00 equiv.), $[Ir(dF(Me)ppy)_2(dtbbpy)]PF_6$ (4.0 mg, 4.0 μmol, 2.0 mol%), $Ni(acac)_2$ (10 mg, 0.040 mmol, 20 mol%), 3-quinuclidinol (25 mg, 0.20 mmol, 1.0 equiv.) and anhydrous DMSO (0.60 mL, c = 0.33 M) were added for cross-coupling process. Biotage Isolera™ flash purification on silica gel (SNAP 5 g silica cartridge), eluting with EtOAc/pentane (0 – 10%, v/v), gave **29** (30 mg, 50%) as a colorless oil.

$\mathbf{R}_f$ = 0.8 (1:10 EtOAc/pentane, UV, $KMnO_4$)

NMR Spectroscopy (*see spectra*):

^{1}H NMR (600 MHz, $CDCl_3$): δ_H 7.86 – 7.82 (m, 2H), 7.73 – 7.67 (m, 2H), 3.68 (t, J = 7.5, 2H), 1.67 – 1.62 (m, 2H), 1.45 – 1.36 (m, 5H), 1.33 – 1.18 (m, 9H), 0.82 (s, 3H) ppm;

^{13}C NMR (151 MHz, $CDCl_3$): δ_C 168.6, 134.0, 132.4, 123.3, 38.3, 37.9, 32.7, 29.8, 26.7, 25.0, 22.2, 20.8 ppm.

IR (film): ν_{max} 2925, 2860, 1711, 1395, 720 cm^{-1}.

HRMS (EI$^+$): calcd. for $C_{19}H_{25}O_2N$ $[M]^+$, 299.1880, found 299.1873.

7-(1-Methylcyclohexyl)heptanenitrile (30)

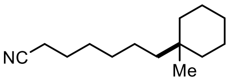

30

Prepared following **General Procedure B**, using 6-cyano-1-hexene **30a** (22 mg, 0.20 mmol, 1.0 equiv.), HBcat (26.4 mg, 0.220 mmol, 1.10 equiv.), $Rh(PPh_3)_3Cl$ (2.0 mg, 2.0 μmol, 1.0 mol%) in anhydrous DCM (0.30 mL, c = 0.67 M) for hydroboration process. After 3 h stirring, DCM was removed and 1,3-dioxoisoindolin-2-yl 1-methylcyclohexane-1-carboxylate **2** (115 mg, 0.400 mmol, 2.00 equiv.), $[Ir(dF(Me)ppy)_2(dtbbpy)]PF_6$ (4.0 mg, 4.0 μmol, 2.0 mol%), $Ni(acac)_2$ (10 mg, 0.040 mmol, 20 mol%), 3-quinuclidinol (25 mg, 0.20 mmol, 1.0 equiv.) and anhydrous DMSO (0.60 mL, c = 0.33 M) were added for cross-coupling process. Biotage Isolera™ flash purification on silica gel (SNAP 5 g silica cartridge), eluting with EtOAc/pentane (0 – 10%, v/v), gave **30** (20 mg, 48%) as a colorless oil.

$\mathbf{R}_f$ = 0.6 (1:10 EtOAc/pentane, UV, $KMnO_4$)

NMR Spectroscopy (*see spectra*):

^{1}H NMR (400 MHz, $CDCl_3$): δ_H 2.33 (t, J = 7.1 Hz, 2H), 1.72 – 1.60 (m, 2H), 1.50 – 1.37 (m, 7H), 1.33 – 1.13 (m, 11H), 0.83 (s, 3H) ppm;

^{13}C NMR (126 MHz, $CDCl_3$): δ_C 120.0, 42.1, 38.0, 32.7, 29.9, 28.9, 26.7, 25.6, 25.1, 23.1, 22.2, 17.3, 14.3 ppm.

IR (film): ν_{max} 2926, 2858, 2246, 1464, 726 cm^{-1}.

HRMS (EI$^+$): calcd. for $C_{14}H_{24}N$ [M-H]$^+$, 206.1903, found 206.1901.

6-(1-Methylcyclohexyl)hexan-2-one (31)

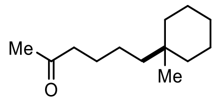

31

Prepared following **General Procedure B**, using 1-hexen-5-one **31a** (20 mg, 0.20 mmol, 1.0 equiv.), HBcat (26.4 mg, 0.220 mmol, 1.10 equiv.), $Rh(PPh_3)_3Cl$ (2.0 mg, 2.0 µmol, 1.0 mol%) in anhydrous DCM (0.30 mL, *c* = 0.67 M) for hydroboration process. After 3 h stirring, DCM was removed and 1,3-dioxoisoindolin-2-yl 1-methylcyclohexane-1-carboxylate **2** (115 mg, 0.400 mmol, 2.00 equiv.), [Ir(*d*F(Me)ppy)$_2$(*d*tbbpy)]PF_6 (4.0 mg, 4.0 µmol, 2.0 mol%), $Ni(acac)_2$ (10 mg, 0.040 mmol, 20 mol%), 3-quinuclidinol (25 mg, 0.20 mmol, 1.0 equiv.) and anhydrous DMSO (0.60 mL, *c* = 0.33 M) were added for cross-coupling process. Biotage Isolera™ flash purification on silica gel (SNAP 5 g silica cartridge), eluting with EtOAc/pentane (0 – 10%, v/v), gave **31** (23 mg, 59%) as a colorless oil.

R*f* = 0.4 (1:10 EtOAc/pentane, UV, $KMnO_4$)

NMR Spectroscopy (*see spectra*):

^{1}H NMR (400 MHz, $CDCl_3$): δ_H 2.43 (t, J = 7.5 Hz, 2H), 2.13 (s, 3H), 1.58 – 1.48 (m, 2H), 1.45 – 1.36 (m, 5H), 1.31 – 1.15 (m, 9H), 0.81 (s, 3H) ppm;

^{13}C NMR (101 MHz, $CDCl_3$): δ_C 209.6, 44.1, 42.0, 38.0, 32.7, 30.0, 26.7, 25.0, 23.1, 22.2 ppm.

IR (film): ν_{max} 2924, 2856, 1717, 1453, 1162, 722 cm^{-1}.

HRMS (EI$^+$): calcd. for $C_{13}H_{24}O$ [M]$^+$, 196.1822, found 196.1818.

***N*-(3-(1-methylcyclohexyl)propyl)benzamide (32)**

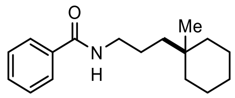

32

Prepared following **General Procedure C**, using *N*-allylbenzamide **32a** (32 mg, 0.20 mmol, 1.0 equiv.), HBcat (26.4 mg, 0.220 mmol, 1.10 equiv.), $Rh(PPh_3)_3Cl$ (2.0 mg, 2.0 µmol, 1.0 mol%) in anhydrous DCM (0.30 mL, *c* = 0.67 M) for hydroboration process. After 3 h stirring, DCM was removed and 1,3-dioxoisoindolin-2-yl 1-methylcyclohexane-1-carboxylate **2** (115 mg, 0.400 mmol, 2.00 equiv.), [Ir(*d*F(Me)ppy)$_2$(*d*tbbpy)]PF_6 (4.0 mg,

4.0 μmol, 2.0 mol%), Ni(acac)$_2$ (10 mg, 0.040 mmol, 20 mol%), 3-quinuclidinol (25 mg, 0.20 mmol, 1.0 equiv.) and anhydrous DMSO (0.60 mL, c = 0.33 M) were added for cross-coupling process. Biotage Isolera™ flash purification on silica gel (SNAP 5 g silica cartridge), eluting with EtOAc/pentane (0 – 15%, v/v), gave **32** (34 mg, 64%) as a colorless oil.

R$_f$ = 0.4 (1:10 EtOAc/pentane, UV, $KMnO_4$)

NMR Spectroscopy (*see spectra*):

^{1}H NMR (500 MHz, $CDCl_3$): δ_H 7.80 – 7.73 (m, 2H), 7.52 – 7.46 (m, 1H), 7.45 – 7.40 (m, 2H), 6.17 (s, 1H), 3.42 (td, J = 7.3, 5.8 Hz, 2H), 1.60 – 1.52 (m, 2H), 1.47 – 1.37 (m, 5H), 1.35 – 1.21 (m, 7H), 0.86 (s, 3H) ppm;

^{13}C NMR (126 MHz, $CDCl_3$): δ_C 167.6, 135.1, 131.4, 128.7, 127.0, 41.1, 39.4, 37.9, 32.6, 26.6, 25.0, 23.9, 22.2 ppm.

IR (film): ν_{max} 3316, 2924, 2852, 1637, 1543, 1308, 695 cm^{-1}.

HRMS (EI$^+$): calcd. for $C_{17}H_{25}NO$ [M]$^+$, 259.1931, found 259.1928.

1-Bromo-4-(4-(1-methylcyclohexyl)butyl)benzene (33)

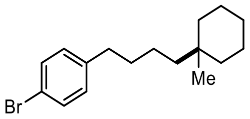

33

Prepared following **General Procedure B**, using 1-(3-butenyl)-4-bromobenzene **33a** (42 mg, 0.20 mmol, 1.0 equiv.), HBcat (26.4 mg, 0.220 mmol, 1.10 equiv.), $Rh(PPh_3)_3Cl$ (2.0 mg, 2.0 μmol, 1.0 mol%) in anhydrous DCM (0.30 mL, c = 0.67 M) for hydroboration process. After 3 h stirring, DCM was removed and 1,3-dioxoisoindolin-2-yl 1-methylcyclohexane-1-carboxylate **2** (115 mg, 0.400 mmol, 2.00 equiv.), [Ir(dF(Me)ppy)$_2$($d$tbbpy)]PF$_6$ (4.0 mg, 4.0 μmol, 2.0 mol%), Ni(acac)$_2$ (10 mg, 0.040 mmol, 20 mol%), 3-quinuclidinol (25 mg, 0.20 mmol, 1.0 equiv.) and anhydrous DMSO (0.60 mL, c = 0.33 M) were added for cross-coupling process. Biotage Isolera™ flash purification on silica gel (SNAP 5 g silica cartridge), eluting with pentane (100%), gave **33** (32 mg, 52%) as a colorless oil.

R$_f$ = 0.5 (pentane, UV, $KMnO_4$)

NMR Spectroscopy (*see spectra*):

^{1}H NMR (400 MHz, $CDCl_3$): δ_H 7.41 – 7.33 (m, 2H), 7.07 – 7.00 (m, 2H), 2.55 (t, J = 8.9, 2H), 1.59 – 1.50 (m, 2H), 1.45 – 1.15 (m, 14H), 0.81 (s, 3H) ppm;

^{13}C NMR (126 MHz, $CDCl_3$): δ_C 142.0, 131.4, 130.3, 119.4, 42.0, 38.0, 35.6, 32.7, 32.5, 26.7, 25.1, 23.0, 22.3 ppm.

IR (film): ν_{max} 2925, 2856, 1488, 1464, 1073, 1012, 800, 517 cm^{-1}.

HRMS (EI$^+$): calcd. for $C_{17}H_{25}Br$ [M]$^+$, 308.1134, found 308.1128.

2-Methoxy-4-(3-(1-methylcyclohexyl)propyl)phenyl trifluoromethanesulfonate (34)

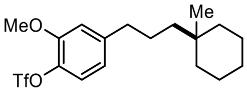

34

Prepared following **General Procedure B**, using 4-allyl-2-methoxyphenyl trifluoromethanesulfonate **34a** (59 mg, 0.20 mmol, 1.0 equiv.), HBcat (26.4 mg, 0.220 mmol, 1.10 equiv.), $Rh(PPh_3)_3Cl$ (2.0 mg, 2.0 μmol, 1.0 mol%) in anhydrous DCM (0.30 mL, *c* = 0.67 M) for hydroboration process. After 3 h stirring, DCM was removed and 1,3-dioxoisoindolin-2-yl 1-methylcyclohexane-1-carboxylate **2** (115 mg, 0.400 mmol, 2.00 equiv.), [Ir(*d*F(Me)ppy)$_2$(*d*tbbpy)]PF_6 (4.0 mg, 4.0 μmol, 2.0 mol%), Ni(acac)$_2$ (10 mg, 0.040 mmol, 20 mol%), 3-quinuclidinol (25 mg, 0.20 mmol, 1.0 equiv.) and anhydrous DMSO (0.60 mL, *c* = 0.33 M) were added for cross-coupling process. Biotage Isolera™ flash purification on silica gel (SNAP 5 g silica cartridge), eluting with EtOAc/pentane (0 – 20%, v/v), gave **34** (25 mg, 32%) as a colorless oil.

$\mathbf{R}_f$ = 0.6 (1:5 EtOAc/pentane, UV, $KMnO_4$)

NMR Spectroscopy (*see spectra*):

^{1}H NMR (500 MHz, $CDCl_3$): δ_H 7.10 (d, *J* = 8.3 Hz, 1H), 6.84 (d, *J* = 2.0 Hz, 1H), 6.78 (dd, *J* = 8.3, 2.0 Hz, 1H), 3.91 (s, 3H), 2.57 (t, *J* = 7.8 Hz, 2H), 1.61 – 1.52 (m, 2H), 1.45 – 1.37 (m, 5H), 1.31 – 1.20 (m, 7H), 0.85 (s, 3H) ppm;

^{13}C NMR (126 MHz, $CDCl_3$): δ_C 151.2, 144.9, 136.9, 122.1, 120.7, 117.63 (q, *J* = 321.0 Hz), 113.3, 56.3, 41.9, 38.0, 37.0, 32.7, 26.7, 25.4, 25.1, 22.2 ppm;

^{19}F NMR (377 MHz, $CDCl_3$): δ_F –73.9 ppm (^{19}F NMR was obtained on Varian 400 MHz NMR spectrometer).

IR (film): ν_{max} 2927, 2850, 1606, 1506, 1419, 1204, 1140, 1106, 877, 616 cm^{-1}.

HRMS (EI^+): calcd. for $C_{18}H_{25}O_4SF_3$ $[M]^+$, 394.1420, found 394.1412.

5-(1-Methylcyclohexyl)pentyl 4-(4,4,5,5-tetramethyl-1,3,2-dioxaborolan-2-yl)benzoate (35)

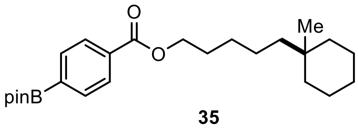

35

Prepared following **General Procedure B**, using pent-4-en-1-yl 4-(4,4,5,5-tetramethyl-1,3,2-dioxaborolan-2-yl)benzoate **35a** (63 mg, 0.20 mmol, 1.0 equiv.), HBcat (26.4 mg, 0.220 mmol, 1.10 equiv.), $Rh(PPh_3)_3Cl$ (2.0 mg, 2.0 μmol, 1.0 mol%) in anhydrous DCM (0.30 mL, *c* = 0.67 M) for hydroboration process. After 3 h stirring, DCM was removed and 1,3-dioxoisoindolin-2-yl 1-methylcyclohexane-1-carboxylate **2** (115 mg, 0.400 mmol, 2.00 equiv.), [Ir(*d*F(Me)ppy)$_2$(*d*tbbpy)]PF_6 (4.0 mg, 4.0 μmol, 2.0 mol%), Ni(acac)$_2$ (10 mg, 0.040 mmol, 20 mol%), 3-quinuclidinol (25 mg, 0.20 mmol, 1.0 equiv.) and anhydrous DMSO (0.60 mL, *c* = 0.33 M) were added for cross-coupling process. Biotage Isolera™ flash purification on silica gel (SNAP 5 g silica cartridge), eluting with EtOAc/pentane (0 – 10%, v/v), gave **35** (45 mg, 54%) as a colorless oil.

R$_f$ = 0.4 (1:10 EtOAc/pentane, UV, $KMnO_4$)

NMR Spectroscopy (see spectra):

^{1}H NMR (400 MHz, $CDCl_3$): δ_H 8.04 – 7.98 (m, 2H), 7.88 – 7.82 (m, 2H), 4.30 (t, J = 6.7 Hz, 2H), 1.82 – 1.71 (m, 2H), 1.45 – 1.37 (m, 6H), 1.34 (s, 12H), 1.32 – 1.16 (m, 10H), 0.82 (s, 3H) ppm;

^{13}C NMR (126 MHz, $CDCl_3$): δ_C 166.9, 134.8, 132.9, 128.7, 84.3, 65.5, 42.1, 38.0, 32.7, 28.9, 27.2, 26.7, 25.2, 25.0, 23.1, 22.3 ppm. The carbon attached to boron was not observed due to quadrupolar relaxation.

^{11}B NMR (128 MHz, $CDCl_3$): δ_B 31.5 ppm.

IR (film): ν_{max} 2926, 2858, 1721, 1400, 1360, 1267, 1112, 1021, 859, 710, 652 cm^{-1}.

HRMS (MALDI$^+$): calcd. for $C_{25}H_{39}O_4BNa$ [M+Na]$^+$, 437.2838, found 437.2833.

5-(1-Methylcyclohexyl)pentyl furan-2-carboxylate (36)

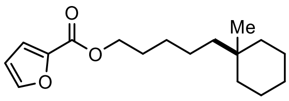

36

Prepared following **General Procedure B**, using pent-4-en-1-yl furan-2-carboxylate **36a** (36 mg, 0.20 mmol, 1.0 equiv.), HBcat (26.4 mg, 0.220 mmol, 1.10 equiv.), $Rh(PPh_3)_3Cl$ (2.0 mg, 2.0 µmol, 1.0 mol%) in anhydrous DCM (0.30 mL, c = 0.67 M) for hydroboration process. After 3 h stirring, DCM was removed and 1,3-dioxoisoindolin-2-yl 1-methylcyclohexane-1-carboxylate **2** (115 mg, 0.400 mmol, 2.00 equiv.), [Ir(dF(Me)ppy)$_2$($d$tbbpy)]PF_6 (4.0 mg, 4.0 µmol, 2.0 mol%), $Ni(acac)_2$ (10 mg, 0.040 mmol, 20 mol%), 3-quinuclidinol (25 mg, 0.20 mmol, 1.0 equiv.) and anhydrous DMSO (0.60 mL, c = 0.33 M) were added for cross-coupling process. Biotage Isolera™ flash purification on silica gel (SNAP 5 g silica cartridge), eluting with EtOAc/pentane (0 – 10%, v/v), followed by preparative thin-layer chromatography purification with EtOAc/pentane (5%, v/v), gave **36** (20 mg, 36%) as a colorless oil.

R$_f$ = 0.6 (1:10 EtOAc/pentane, UV, $KMnO_4$)

NMR Spectroscopy (see spectra):

^{1}H NMR (500 MHz, $CDCl_3$): δ_H 7.57 (dd, J = 1.7, 0.9 Hz, 1H), 7.17 (dd, J = 3.5, 0.9 Hz, 1H), 6.50 (dd, J = 3.5, 1.7 Hz, 1H), 4.30 (t, J = 6.8 Hz, 2H), 1.81 – 1.71 (m, 2H), 1.45 – 1.36 (m, 7H), 1.32 – 1.25 (m, 3H), 1.24 – 1.17 (m, 6H), 0.83 (s, 3H) ppm;

^{13}C NMR (126 MHz, $CDCl_3$): δ_C 159.0, 146.3, 145.1, 117.8, 111.9, 65.3, 42.1, 38.0, 32.7, 28.9, 27.1, 26.7, 25.2, 23.1, 22.3 ppm.

IR (film): ν_{max} 2925, 2855, 1721, 1471, 1296, 1180, 1119, 764 cm^{-1}.

HRMS (EI$^+$): calcd. for $C_{17}H_{26}O_3$ [M]$^+$, 278.1876, found 278.1874.

5-(1-Methylcyclohexyl)pentyl thiophene-3-carboxylate (37)

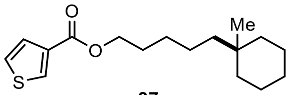

37

Prepared following **General Procedure B**, using pent-4-en-1-yl thiophene-3-carboxylate **37a** (39 mg, 0.20 mmol, 1.0 equiv.), HBcat (26.4 mg, 0.220 mmol, 1.10 equiv.), $Rh(PPh_3)_3Cl$ (2.0 mg, 2.0 µmol, 1.0 mol%) in anhydrous DCM (0.30 mL, *c* = 0.67 M) for hydroboration process. After 3 h stirring, DCM was removed and 1,3-dioxoisoindolin-2-yl 1-methylcyclohexane-1-carboxylate **2** (115 mg, 0.400 mmol, 2.00 equiv.), [Ir(*d*F(Me)ppy)$_2$(*d*tbbpy)]PF_6 (4.0 mg, 4.0 µmol, 2.0 mol%), Ni(acac)$_2$ (10 mg, 0.040 mmol, 20 mol%), 3-quinuclidinol (25 mg, 0.20 mmol, 1.0 equiv.) and anhydrous DMSO (0.60 mL, *c* = 0.33 M) were added for cross-coupling process. Biotage Isolera™ flash purification on silica gel (SNAP 5 g silica cartridge), eluting with EtOAc/pentane (0 – 10%, v/v), gave **37** (21 mg, 36%) as a colorless oil.

$\mathbf{R}_f$ = 0.7 (1:10 EtOAc/pentane, UV, $KMnO_4$)

NMR Spectroscopy of (*see spectra*):

^{1}**H NMR** (500 MHz, $CDCl_3$): δ_H 8.10 (dd, *J* = 3.0, 1.2 Hz, 1H), 7.53 (dd, *J* = 5.1, 1.2 Hz, 1H), 7.30 (dd, *J* = 5.1, 3.0 Hz, 1H), 4.27 (t, *J* = 6.7 Hz, 2H), 1.79 – 1.71 (m, 2H), 1.46 – 1.35 (m, 7H), 1.33 – 1.26 (m, 3H), 1.25 – 1.17 (m, 6H), 0.83 (s, 3H) ppm;

^{13}**C NMR** (126 MHz, $CDCl_3$): δ_C 163.1, 134.2, 132.6, 128.1, 126.0, 65.1, 42.1, 38.0, 32.7, 29.0, 27.2, 26.7, 25.2, 23.1, 22.2 ppm.

IR (film) ν_{max} 2926, 2858, 1717, 1524, 1260, 1188, 1104, 748 cm^{-1}.

HRMS (EI$^+$): calcd. for $C_{17}H_{26}O_2S$ [M]$^+$, 294.1648, found 294.1646.

5-(1-Methylcyclohexyl)pentyl isonicotinate (38)

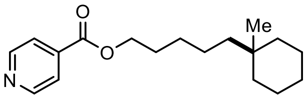

38

Prepared following **General Procedure B**, using pent-4-en-1-yl isonicotinate **38a** (38 mg, 0.20 mmol, 1.0 equiv.), HBcat (26.4 mg, 0.220 mmol, 1.10 equiv.), $Rh(PPh_3)_3Cl$ (2.0 mg, 2.0 µmol, 1.0 mol%) in anhydrous DCM (0.30 mL, *c* = 0.67 M) for hydroboration process. After 3 h stirring, DCM was removed and 1,3-dioxoisoindolin-2-yl 1-methylcyclohexane-1-carboxylate **2** (115 mg, 0.400 mmol, 2.00 equiv.), [Ir(*d*F(Me)ppy)$_2$(*d*tbbpy)]PF_6 (4.0 mg, 4.0 µmol, 2.0 mol%), Ni(acac)$_2$ (10 mg, 0.040 mmol, 20 mol%), 3-quinuclidinol (25 mg, 0.20 mmol, 1.0 equiv.) and anhydrous DMSO (0.60 mL, *c* = 0.33 M) were added for cross-coupling process. Biotage Isolera™ flash purification on silica gel (SNAP 5 g silica cartridge), eluting with EtOAc/pentane (0 – 10%, v/v), gave **38** (21 mg, 37%) as a colorless oil.

$\mathbf{R}_f$ = 0.4 (1:10 EtOAc/pentane, UV, $KMnO_4$)

NMR Spectroscopy (*see spectra*):

^{1}H NMR (400 MHz, $CDCl_3$): δ_H 8.82 – 8.70 (m, 2H), 7.88 – 7.78 (m, 2H), 4.35 (t, J = 6.7 Hz, 2H), 1.82 – 1.76 (m, 2H), 1.46 – 1.36 (m, 7H), 1.34 – 1.26 (m, 3H), 1.25 – 1.19 (m, 6H), 0.84 (s, 3H) ppm;

^{13}C NMR (126 MHz, $CDCl_3$): δ_C 165.3, 150.7, 137.8, 123.0, 66.2, 42.1, 38.0, 32.7, 28.8, 27.1, 26.7, 25.1, 23.1, 22.2 ppm.

IR (film) ν_{max} 2926, 2858, 1729, 1408, 1279, 1122, 757, 707 cm^{-1}.

HRMS (EI$^+$): calcd. for $C_{18}H_{26}O_2N$ [M-H]$^+$, 288.1958, found 288.1955.

7-(2,5-Dimethylphenoxy)-4,4-dimethylheptan-1-ol (39)

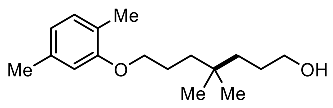

39

Prepared following **General Procedure B**, using allylboronic acid pinacol ester **39a** (34 mg, 0.20 mmol, 1.0 equiv.), HBcat (26.4 mg, 0.220 mmol, 1.10 equiv.), $Rh(PPh_3)_3Cl$ (2.0 mg, 2.0 μmol, 1.0 mol%) in anhydrous DCM (0.30 mL, c = 0.67 M) for hydroboration process. After 3 h stirring, DCM was removed and 1,3-dioxoisoindolin-2-yl 5-(2,5-dimethylphenoxy)-2,2-dimethylpentanoate **R11** (158 mg, 0.400 mmol, 2.00 equiv.), $[Ir(d\text{F(Me)ppy})_2(d\text{tbbpy})]PF_6$ (4.0 mg, 4.0 μmol, 2.0 mol%), $Ni(acac)_2$ (10 mg, 0.040 mmol, 20 mol%), 3-quinuclidinol (25 mg, 0.20 mmol, 1.0 equiv.) and anhydrous DMSO (0.60 mL, c = 0.33 M) were added for cross-coupling process. After irradiation, the work-up residue was dissolved into THF (2.0 mL), NaOH aqueous (c = 2 M, 0.9 mL) and H_2O_2 aqueous (30%, 0.45 mL) were added stepwise at 0 ℃, then the solution was warm to room temperature and stirred for 2 h. Afterwards the reaction was diluted by 1 mL wet EtOAc and washed by 5 mL deionized water and 5 mL brine, extracted with EtOAc (3 × 10 mL). The combined organic layers were washed with brine (10 mL), dried over $MgSO_4$, filtered, and concentrated *in vacuo*. The residue was purified by flash column chromatography or Biotage Isolera™ flash purification on silica gel, gave **39** (23 mg, 43%) as a colorless oil.

R*f* = 0.4 (1:10 EtOAc/pentane, UV, $KMnO_4$)

NMR Spectroscopy (*see spectra*):

^{1}H NMR (500 MHz, $CDCl_3$): δ_H 7.03 – 6.98 (m, 1H), 6.68 – 6.60 (m, 2H), 3.92 (t, J = 6.5 Hz, 2H), 3.63 (t, J = 6.7 Hz, 2H), 2.31 (s, 3H), 2.18 (s, 3H), 1.78 – 1.70 (m, 2H), 1.56 – 1.50 (m, 2H), 1.41 – 1.33 (m, 2H), 1.31 – 1.24 (m, 2H), 0.91 (s, 6H) ppm;

^{13}C NMR (126 MHz, $CDCl_3$): δ_C 157.2, 136.6, 130.4, 123.7, 120.7, 112.2, 68.7, 64.0, 38.1, 37.8, 32.4, 27.66, 27.3, 24.4, 21.6, 15.9 ppm.

IR (film) ν_{max}: 3352, 2926, 2855, 1509, 1468, 1265, 1130, 1057, 802 cm^{-1}.

HRMS (ESI$^+$): calcd. for $C_{17}H_{29}O_2$ [M+H]$^+$, 265.2162, found 265.2163.

7-(4-(4-(2,3-Dichlorophenyl)piperazin-1-yl)butoxy)-1-(5-(1-methylcyclohexyl)pentyl)-3,4-dihydroquinolin-2(1*H*)-one (40)

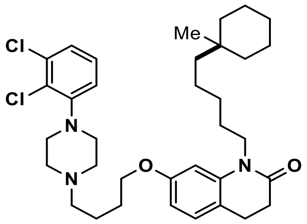

40

Prepared following **General Procedure B**, using 7-(4-(4-(2,3-dichlorophenyl)piperazin-1-yl)butoxy)-1-(pent-4-en-1-yl)-3,4-dihydroquinolin-2(1H)-one **40a** (103 mg, 0.200 mmol, 1.00 equiv.), HBcat (26.4 mg, 0.220 mmol, 1.10 equiv.), $Rh(PPh_3)_3Cl$ (2.0 mg, 2.0 μmol, 1.0 mol%) in anhydrous DCM (0.30 mL, c = 0.67 M) for hydroboration process. After 3 h stirring, DCM was removed and 1,3-dioxoisoindolin-2-yl 1-methylcyclohexane-1-carboxylate **2** (115 mg, 0.400 mmol, 2.00 equiv.), $[Ir(dF(Me)ppy)_2(dtbbpy)]PF_6$ (4.0 mg, 4.0 μmol, 2.0 mol%), $Ni(acac)_2$ (10 mg, 0.040 mmol, 20 mol%), 3-quinuclidinol (25 mg, 0.20 mmol, 1.0 equiv.) and anhydrous DMSO (0.60 mL, c = 0.33 M) were added for cross-coupling process. Biotage Isolera™ flash purification on silica gel (SNAP 5 g silica cartridge), eluting with EtOAc/pentane (0 – 10%, v/v), followed by preparative thin-layer chromatography purification with EtOAc/pentane (5%, v/v), gave **40** (38 mg, 31%) as a colorless oil.

R_f = 0.4 (1:10 EtOAc/pentane, UV, $KMnO_4$)

NMR Spectroscopy (*see spectra*):

> **^{1}H NMR** (500 MHz, $CDCl_3$): δ_H 7.18 – 7.11 (m, 2H), 7.07 – 7.01 (m, 1H), 6.98 – 6.93 (m, 1H), 6.58 – 6.48 (m, 2H), 3.99 (t, J = 6.3 Hz, 2H), 3.88 (t, J = 7.7 Hz, 2H), 3.15 – 3.01 (m, 4H), 2.84 – 2.77 (m, 2H), 2.74 – 2.63 (m, 4H), 2.62 – 2.58 (m, 2H), 2.52 (t, J = 7.7 Hz, 2H), 1.91 – 1.81 (m, 3H), 1.78 – 1.70 (m, 2H), 1.68 – 1.60 (m, 2H), 1.44 – 1.15 (m, 16H), 0.82 (s, 3H) ppm;

> **^{13}C NMR** (126 MHz, $CDCl_3$): δ_C 170.4, 158.7, 151.3, 140.8, 134.2, 128.5, 127.7, 127.6, 124.8, 119.0, 118.7, 107.1, 103.3, 68.0, 58.3, 53.4, 51.4, 42.5, 42.1, 38.0, 32.7, 32.4, 28.1, 27.4, 26.7, 25.1, 25.0, 23.5, 23.1, 22.4, 22.2 ppm.

IR (film): ν_{max} 2925, 2854, 1674, 1616, 1448, 1357, 1139, 963, 780, 713 cm^{-1}.

HRMS (ESI^+): calcd. for $C_{35}H_{50}O_2N_3Cl_2$ $[M+H]^+$, 614.3275, found 614.3260.

4-(2,2-Difluorobenzo[*d*][1,3]dioxol-4-yl)-1-(5-(1-methylcyclohexyl)pentyl)-1*H*-pyrrole-3-carbonitrile (41)

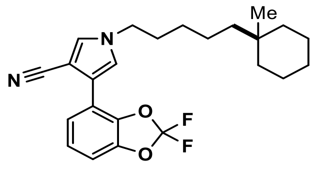

41

Prepared following **General Procedure B**, using 4-(2,2-difluorobenzo[*d*][1,3]dioxol-4-yl)-1-(pent-4-en-1-yl)-1H-pyrrole-3-carbonitrile **41a** (63 mg, 0.20 mmol, 1.0 equiv.), HBcat (26.4 mg, 0.220 mmol, 1.10 equiv.), $Rh(PPh_3)_3Cl$ (2.0 mg, 2.0 μmol, 1.0 mol%) in anhydrous DCM (0.30 mL, *c* = 0.67 M) for hydroboration process. After 3 h stirring, DCM was removed and 1,3-dioxoisoindolin-2-yl 1-methylcyclohexane-1-carboxylate **2** (115 mg, 0.400 mmol, 2.00 equiv.), $[Ir(dF(Me)ppy)_2(dtbbpy)]PF_6$ (4.0 mg, 4.0 μmol, 2.0 mol%), $Ni(acac)_2$ (10 mg, 0.040 mmol, 20 mol%), 3-quinuclidinol (25 mg, 0.20 mmol, 1.0 equiv.) and anhydrous DMSO (0.60 mL, *c* = 0.33 M) were added for cross-coupling process. Biotage Isolera™ flash purification on silica gel (SNAP 5 g silica cartridge), eluting with EtOAc/pentane (0 – 10%, v/v), gave **41** (35 mg, 42%) as a colorless oil.

R*f* = 0.4 (1:10 EtOAc/pentane, UV, $KMnO_4$)

NMR Spectroscopy (*see spectra*):

^{1}H NMR (500 MHz, $CDCl_3$): δ_H 7.72 (dd, *J* = 8.2, 1.1 Hz, 1H), 7.23 (d, *J* = 2.3 Hz, 1H), 7.17 – 7.11 (m, 2H), 6.98 – 6.94 (m, 1H), 3.94 (t, *J* = 7.2 Hz, 2H), 1.89 – 1.81 (m, 2H), 1.45 – 1.38 (m, 5H), 1.32 – 1.25 (m, 5H), 1.24 – 1.16 (m, 6H), 0.83 (s, 3H) ppm;

^{13}C NMR (126 MHz, $CDCl_3$): δ_C 144.0, 139.9, 131.5 (t, *J* = 252.0 Hz), 129.6, 124.2, 122.5, 121.3, 118.6, 116.8, 116.7, 107.8, 91.1, 50.5, 42.0, 37.9, 32.6, 31.2, 27.6, 26.7, 25.0, 22.9, 22.2 ppm;

^{19}F NMR (377 MHz, $CDCl_3$): δ_F –49.5 ppm (^{19}F NMR was obtained on Varian 400 MHz NMR spectrometer).

IR (film): v_{max} 2927, 2858, 2223, 1528, 1455, 1245, 1152, 1033, 920, 783, 720 cm^{-1}.

HRMS ($MALDI^+$): calcd. for $C_{24}H_{28}O_2N_2F_2Na$ $[M+Na]^+$, 437.2011, found 437.2017.

2.7. Scale-up Reaction

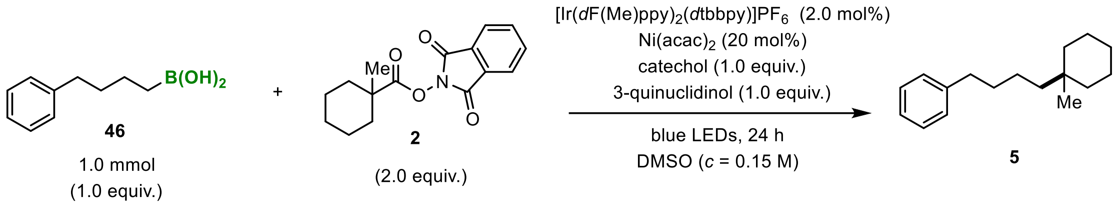

Under an ambient atmosphere, a flame dried 20 mL reaction vial equipped with a magnetic stir bar was transferred into an anhydrous, argon-filled glovebox and charged with [Ir(*d*F(Me)ppy)$_2$(*d*tbbpy)]PF$_6$ (21 mg, 20 μmol, 2.0 mol%), Ni(acac)$_2$ (52 mg, 0.20 mmol, 20 mol%), (4-phenylbutyl)boronic acid **46** (178 mg, 1.00 mmol, 1.00 equiv.), 1,3-dioxoisoindolin-2-yl 1-methylcyclohexane-1-carboxylate **2** (575 mg, 2.00 mmol, 2.00 equiv.), catechol (110 mg, 1.00 mmol, 1.00 equiv), 3-quinuclidinol (127 mg, 1.00 mmol, 1.00 equiv.) sequentially, followed by the anhydrous DMSO (6.6 mL, *c* = 0.15 M). The vial was sealed with a cap and reaction mixture was pre-stirred in dark for 30 minutes, then the vial removed from the glovebox, and placed 4 cm away from two blue LED lamps (Kessil Tuna Blue LEDs). After stirring at 1200 rpm under irradiation for 19 h with fan cooling, the reaction mixture was diluted with wet EtOAc (5 mL), washed with deionized water (30 mL) and brine (30 mL), then extracted with EtOAc (3 × 30 mL). The combined organic layers were washed with brine (20 mL), dried over MgSO$_4$, filtered, and concentrated *in vacuo*. Biotage Isolera^{TM} flash purification on silica gel (SNAP 10 g silica cartridge), eluting with pentane (100%), gave **5** (164 mg, 71%) as a colorless oil.

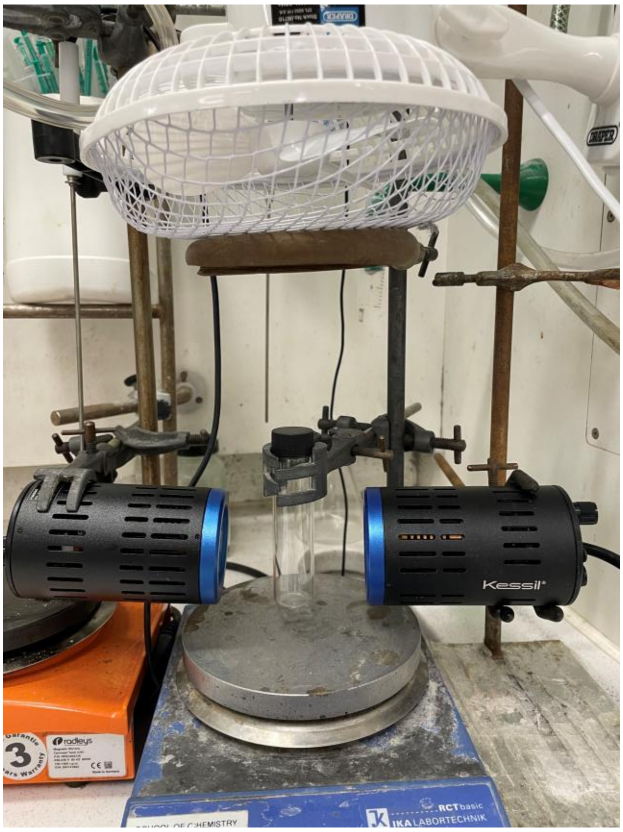

2.8. Synthesis Application

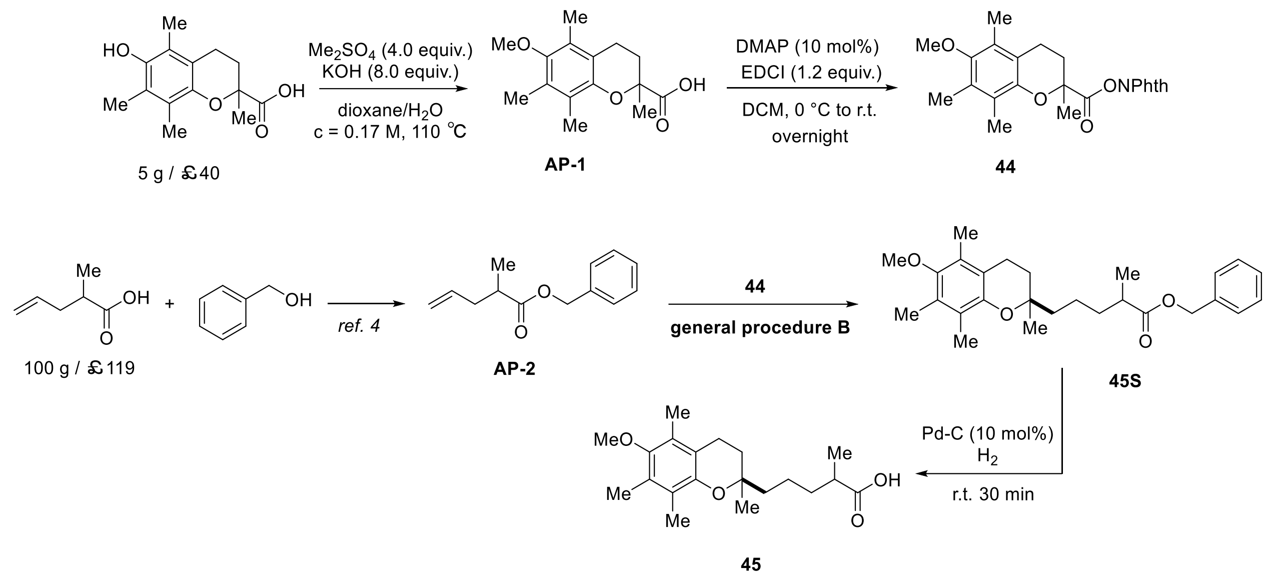

Synthesis of AP-1

AP-1 was prepared according to modified literature procedure[3]. A mixture of Trolox (1.25 g, 5.00 mmol, 1.00 equiv) and KOH (1.12 g, 20.0 mmol, 4.00 equiv) in dioxane/H_2O (10 mL/20 mL, *c* = 0.17 M) was stirred under a N_2 atmosphere. Me_2SO_4 (2.52 g, 20.0 mmol, 4.00 equiv) was added, and the reaction mixture was stirred at 110 ℃ for 3 h. After cooling to room temperature, additional KOH (1.12 g, 20.0 mmol, 4.00 equiv) was added, and the mixture was stirred at 110 ℃ for 2 h. Then the mixture was cooled to room temperature, diluted with H_2O (30 mL) and washed with Et_2O (3 × 30 mL). The aqueous layer was separated, acidified to pH 1 with 1 M HCl aqueous, and extracted with DCM (3 × 20 mL). The combined organic extracts were washed with brine (20 mL), dried over anhydrous $MgSO_4$, and concentrated *in vacuo* to afford **AP-1**, which was used directly in the next step without further purification.

Synthesis of 1,3-dioxoisoindolin-2-yl 6-methoxy-2,5,7,8-tetramethylchromane-2-carboxylate (44)

AP-1 (5.0 mmol, 1.0 equiv.) and 4-(dimethylamino)pyridine (DMAP, 61 mg, 0.50 mmol, 10 mol%) were added to a solution of *N*-hydroxyphthalimide (0.90 g, 5.5 mmol, 1.1 equiv.) in anhydrous DCM (20 mL, *c* = 0.050 M) at room temperature, followed by the portionwise addition of 1-ethyl-3-(3'-dimethylaminopropyl)carbodiimide hydrochloride (EDCI, 0.93 g, 6.0 mmol, 1.2 equiv.) at 0 ℃. The reaction warmed up to room temperature after 2 h and then stirred for 10 h. Subsequently, the solvent was removed *in vacuo* and a mixture of EtOAc/H_2O (v/v = 3:1) was added. The layers were separated, and the aqueous layer was extracted with EtOAc (2 × 30 mL). The combined organic layers were washed with saturated aqueous $NaHCO_3$ solution, dried over $MgSO_4$, filtered, and concentrated *in vacuo*. The residue was purified by flash column chromatography, eluting with EtOAc/pentane (0 – 15%, v/v) to give **44** (1.4 g, 68% for 2 steps) as a white solid.

R_f = 0.3 (1:5 EtOAc/pentane, UV, $KMnO_4$)

NMR Spectroscopy (*see spectra*):

^{1}H NMR (400 MHz, $CDCl_3$): δ_H 7.88 – 7.81 (m, 2H), 7.79 – 7.73 (m, 2H), 3.62 (s, 3H), 2.93 – 2.81 (m, 1H), 2.79 – 2.70 (m, 1H), 2.68 – 2.59 (m, 1H), 2.17 (s, 3H), 2.154 (s, 3H), 2.149 (s, 3H), 2.11 – 2.02 (m, 1H),

1.88 (s, 3H) ppm;

^{13}C NMR (101 MHz, $CDCl_3$): δ_C 170.1, 161.9, 150.7, 147.2, 134.9, 129.1, 128.4, 126.1, 124.1, 123.0, 117.2, 77.1, 60.5, 31.3, 25.8, 20.8, 12.7, 11.9, 11.8 ppm.

All recorded spectroscopic data matched those previously reported in the literature.[3]

Synthesis of 45S

Prepared following modified **General Procedure B**. Under an ambient atmosphere, a flame dried 8 mL Biotage® microwave reaction vial equipped with a magnetic stir bar was transferred into an anhydrous, argon-filled glovebox and charged with alkene **AP-2** (41 mg, 0.20 mmol, 1.0 equiv.), $Rh(PPh_3)_3Cl$ (2.0 mg, 2.0 μmol, 1.0 mol%) and anhydrous DCM (0.10 mL, *c* = 2.0 M), then cooled to 0 ℃, HBcat (26.4 mg, 0.220 mmol, 1.10 equiv.) was added. The vial was sealed with a cap with septum, removed from the glovebox and stirred at room temperature for 3 h. After removal of the solvent *in vacuo*, the vial was backfilled with N_2 and transferred back into the glovebox. [Ir(*p*-F(Me)ppy)$_2$-(4,4'-*d*tbbpy)]PF_6 (4 mg, 0.004 mmol, 2 mol%), $Ni(acac)_2$ (10 mg, 0.040 mmol, 0.20 equiv.), redox-active ester **44** (164 mg, 0.400 mmol, 2.00 equiv.) and 3-quinuclidinol (25 mg, 0.20 mmol, 1.0 equiv.) were added sequentially, followed by the anhydrous DMSO (2.0 mL, *c* = 0.10 M). The mixture was pre-stirred for 10 min without irradiation until a homogeneous solution formed and subsequently positioned 3 cm from a blue LED light source (Kessil Tuna Blue LEDs). After stirring at 1200 rpm under irradiation for 19 h with fan cooling, the reaction mixture was diluted with wet EtOAc (1 mL), washed with deionized water (5 mL) and brine (5 mL), and extracted with EtOAc (3 × 10 mL). The combined organic layers were washed with brine (10 mL), dried over $MgSO_4$, filtered, and concentrated *in vacuo* to afford crude **45S** as a yellow oil which was used for the deprotection of benzyl group without further purification.

Synthesis of 5-(6-methoxy-2,5,7,8-tetramethylchroman-2-yl)-2-methylpentanoic acid (45)

45S (0.10 mmol, 1.0 equiv), Pd–C (10 wt%, 2.1 mg) and MeOH (10 mL, *c* = 0.10 M) were charged in a flame-dried 25 mL round-bottom flask with a magnetic stir bar. The resulting suspension was stirred under a hydrogen atmosphere at room temperature for 30 min. The reaction mixture was filtered through a pad of celite, and the filtrate was concentrated *in vacuo*. Biotage Isolera™ flash purification on silica gel (SNAP 5 g silica cartridge), eluting with EtOAc/pentane (0 – 40%, v/v), gave **45** (26 mg, 59% for 2 steps) as a colorless oil.

R_f = 0.3 (1:3 EtOAc/pentane, UV, $KMnO_4$)

NMR Spectroscopy (<u>*see spectra*</u>):

^{1}H NMR (400 MHz, $CDCl_3$): δ_H 3.63 (s, 3H), 2.58 (t, *J* = 6.9 Hz, 2H), 2.52 – 2.46 (m, 1H), 2.18 (s, 3H), 2.13 (s, 3H), 2.07 (s, 3H), 1.83 – 1.66 (m, 3H), 1.64 – 1.42 (m, 5H), 1.24 – 1.21 (m, 3H), 1.19 (dd, *J* = 7.0, 2.8 Hz, 3H) ppm;

^{13}C NMR (126 MHz, $CDCl_3$): δ_C 182.2, 149.6, 147.8, 128.0, 125.9, 123.0, 117.6, 74.7, 60.6, 39.8 (d, *J* = 12.9 Hz), 39.3 (d, *J* = 9.7 Hz), 34.1 (d, *J* = 1.5 Hz), 31.5, 23.9 (d, *J* = 2.1 Hz), 21.4 (d, *J* = 9.3 Hz), 20.7, 17.0 (d, *J* = 8.7 Hz), 12.7, 11.9 (d, *J* = 1.2 Hz), 11.8 ppm.

IR (film): ν_{max} 2928, 1705, 1458, 1404, 1257, 1089, 1014, 927 cm^{-1}.

HRMS ($Nano^-$): calcd. for $C_{20}H_{29}O_4$ $[M-H]^-$, 333.2066, found 333.2060.

2.9. Unsuccessful Substrates

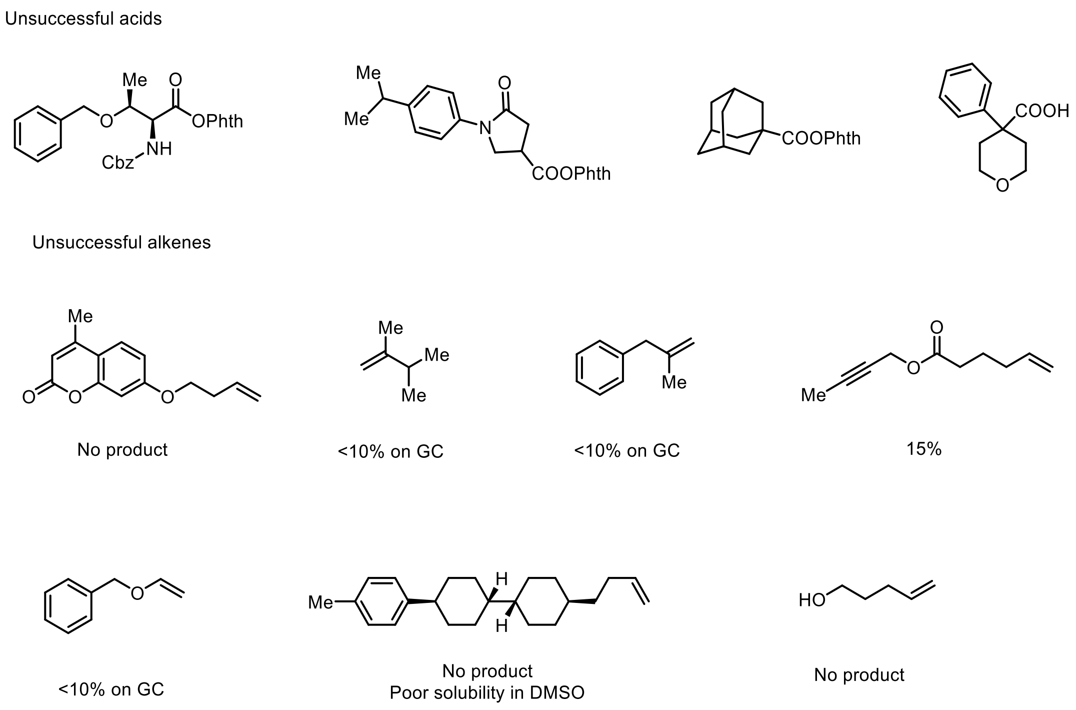

Figure S3. Unsuccessful substrates

3. MECHANISTIC STUDIES

3.1. ^{11}B NMR experiment

Under an ambient atmosphere, flame dried 8 mL Biotage® microwave reaction vials equipped with magnetic stir bars and transferred into an anhydrous, argon-filled glovebox where charged with specific chemicals (1.0 mmol, 1.0 equiv.) and anhydrous solvents (3.0 mL, *c* = 0.33 M) mentioned below. The vials were sealed with caps with septum and stirred at a speed of 1200 rpm for 1 h in glovebox. After that, the portions of these reaction solutions were transferred into different J. Young NMR tubes separately, removed from the glovebox for ^{11}B NMR analysis (Figure S4, **a-f**).

Figure S4 shows the ^{11}B NMR spectra of the (4-phenylbutyl)boronic **46** (0.18 g, 1.0 mmol, 1.0 equiv.) solution with different reaction components. These experiments aim to figure out the boron species exist during the standard reaction. Figure **S4a** and **S4b** refer to the **46** in $CDCl_3$ (1 mL, *c* = 0.33 M) and DMSO (1 mL, *c* = 0.33 M) separately. Figure **S4c** refers to the mixture of **46** and catechol (0.11 g, 1.0 mmol, 1.0 equiv.) in DMSO (1 mL, *c* = 0.33 M), Figure **S4d** refers to the mixture of **46** and 3-quinuclidinol (0.13 g, 1.0 mmol, 1.0 equiv.) in DMSO (1 mL, *c* = 0.33 M), Figure **S4e** refers to the mixture of **46**, catechol (0.11 g, 1.0 mmol, 1.0 equiv.) and 3-quinuclidinol (0.13 g, 1.0 mmol, 1.0 equiv.) in DMSO (1 mL, *c* = 0.33 M), Figure **S4f** refers to the mixture of **46**, catechol (0.11 g, 1.0 mmol, 1.0 equiv.) and DIPEA (*N*,*N*-diisopropylethylamine, 0.13 g, 1.0 mmol, 1.0 equiv.) in DMSO (1 mL, *c* = 0.33 M).

Boronic acid **52** gives slightly different chemical shifts in $CDCl_3$ and DMSO (Figure **S4a** and **S4b**). **46** combined with catechol (Figure **S4c**) or 3-quinuclidinol (Figure **S4d**) give close chemical shifts with Figure **S4b**. When **46** stirred with both catechol and 3-quinuclidinol in DMSO for 1 h (Figure **S4e**), the boronic acid peak disappeared with a new peak formed at 9.12 ppm, indicating **46** is fully converted to a new boronated complex. The boronic acid stirred with catechol and another additive DIPEA were also tested and provided a similar boronated complex peak in 10.85 ppm but still had boronic acid left (Figure **S4f**).

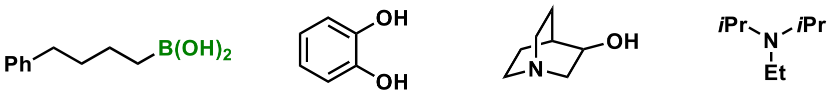

46 *catechol* *3-quinuclidinol* *DIPEA*

Entry	Components
a	**46** in $CDCl_3$
b	**46** in DMSO
c	**46** + catechol in DMSO
d	**46** + 3-quinuclidinol in DMSO
e	**46** + 3-quinuclidinol +catechol in DMSO
f	**46** + DIPEA +catechol in DMSO

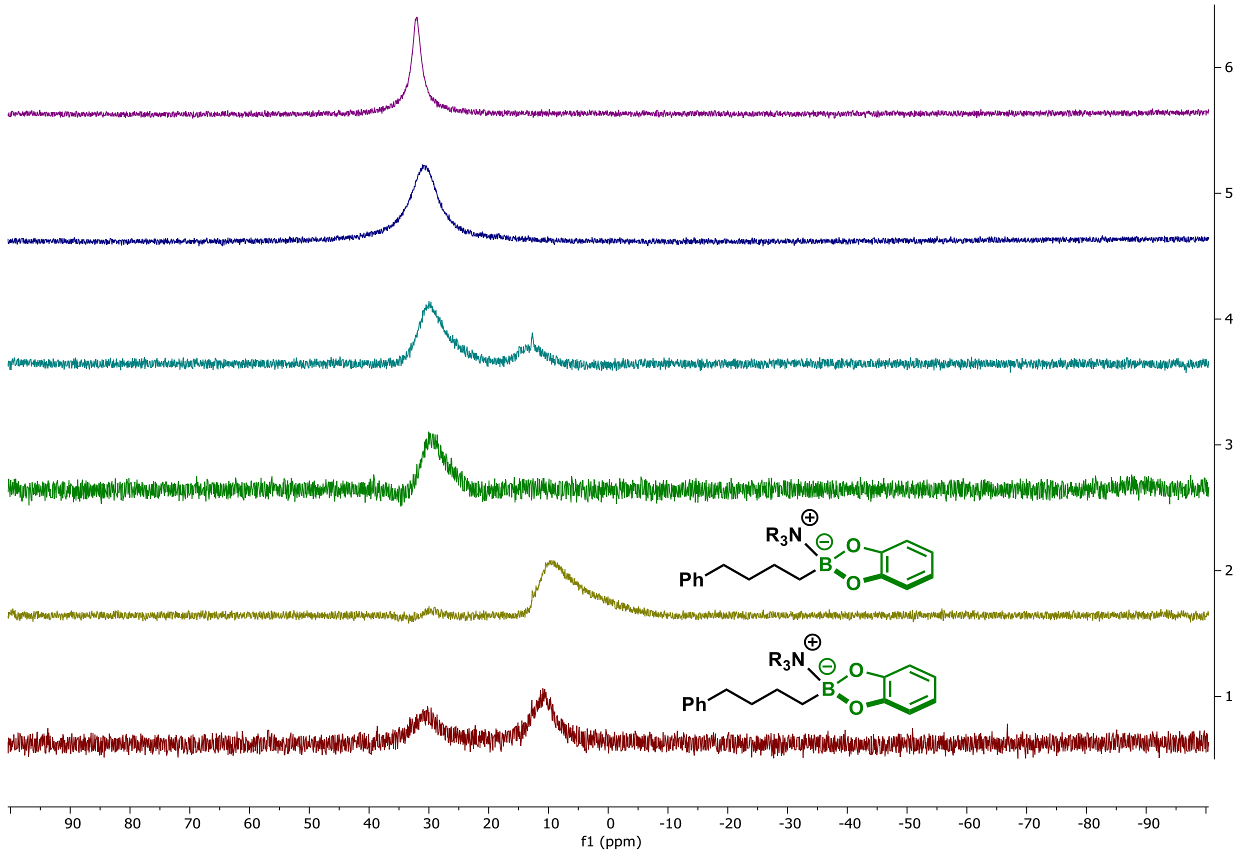

Figure S4. ^{11}**B NMR spectra of different combinations with (4-phenylbutyl)boronic acid 46**

3.2. Radical trap experiment

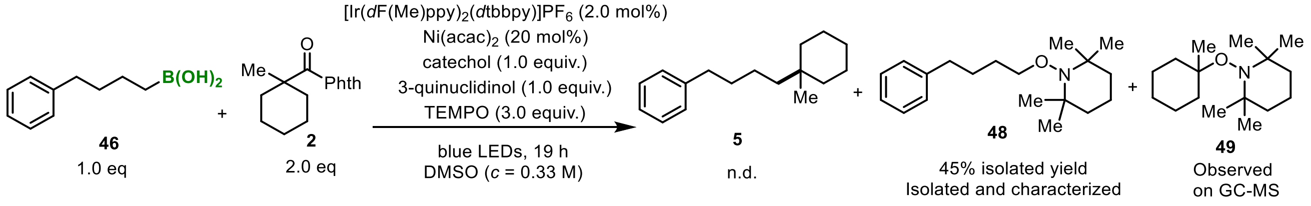

Under an ambient atmosphere, a flame dried 8 mL Biotage® microwave reaction vial equipped with a magnetic stir bar was transferred into an anhydrous, argon-filled glovebox and charged with (4-phenylbutyl)boronic acid **46** (36 mg, 0.20 mmol, 1.0 equiv.), 1,3-dioxoisoindolin-2-yl 1-methylcyclohexane-1-carboxylate **2** (115 mg, 0.400 mmol, 2.00 equiv.), [Ir(*d*F(Me)ppy)$_2$(*d*tbbpy)]PF$_6$ (4.0 mg, 4.0 μmol, 2.0 mol%), Ni(acac)$_2$ (10 mg, 0.040 mmol, 20 mol%), catechol (22 mg, 0.20 mmol, 1.0 equiv), 3-quinuclidinol (25 mg, 0.20 mmol, 1.0 equiv.) and 2,2,6,6-tetramethyl-1-piperidinyloxy free radical (TEMPO, 94 mg, 0.60 mmol, 3.0 equiv.) sequentially, followed by the anhydrous DMSO (0.60 mL, c = 0.33 M). The vial was sealed with a cap with septum, removed from the glovebox. The mixture was pre-stirred for 10 min without irradiation until a homogeneous solution formed and subsequently positioned 3 cm from a blue LED light source (Kessil Tuna Blue LEDs). After stirring at 1200 rpm under irradiation for 19 h with fan cooling, the reaction mixture was diluted with wet EtOAc (1 mL), washed with deionized water (5 mL) and brine (5 mL), and extracted with EtOAc (3 × 10 mL). The combined organic layers were washed with brine (10 mL), dried over MgSO$_4$, filtered, and concentrated *in vacuo,* then monitored by GC-

MS and two radical trap adducts **48** and **49** were observed (see GC-FID trace below). Then the crude mixture was purified by Biotage Isolera^{TM} flash purification on silica gel with EtOAc/pentane (0 – 10%, v/v) to give **48** (26 mg, 45%) as a colorless oil.

R*f* = 0.6 (1:10 EtOAc/pentane, UV, $KMnO_4$)

NMR Spectroscopy of 48 (*see spectra*):

> ^{1}**H NMR** (400 MHz, $CDCl_3$): δ_H 7.31 – 7.25 (m, 2H), 7.23 – 7.15 (m, 3H), 3.76 (t, J = 6.5 Hz, 2H), 2.67 – 2.60 (m, 2H), 1.75 – 1.67 (m, 2H), 1.60 – 1.54 (m, 2H), 1.47 – 1.43 (m, 2H), 1.36 – 1.23 (m, 4H), 1.15 (s, 6H), 1.09 (s, 6H) ppm;

> ^{13}**C NMR** (101 MHz, $CDCl_3$): δ_C 142.8, 128.6, 128.4, 125.8, 76.7, 59.8, 39.8, 36.2, 33.2, 28.6, 28.6, 20.3, 17.3 ppm.

IR (film) ν_{max}: 2930, 2870, 1738, 1454, 1373, 1133, 964, 698 cm^{-1}.

HRMS (ESI$^+$): calcd. for $C_{19}H_{32}ON$ [M+H]$^+$, 290.2478, found 290.2478.

GC-MS trace of radical trap experiment:

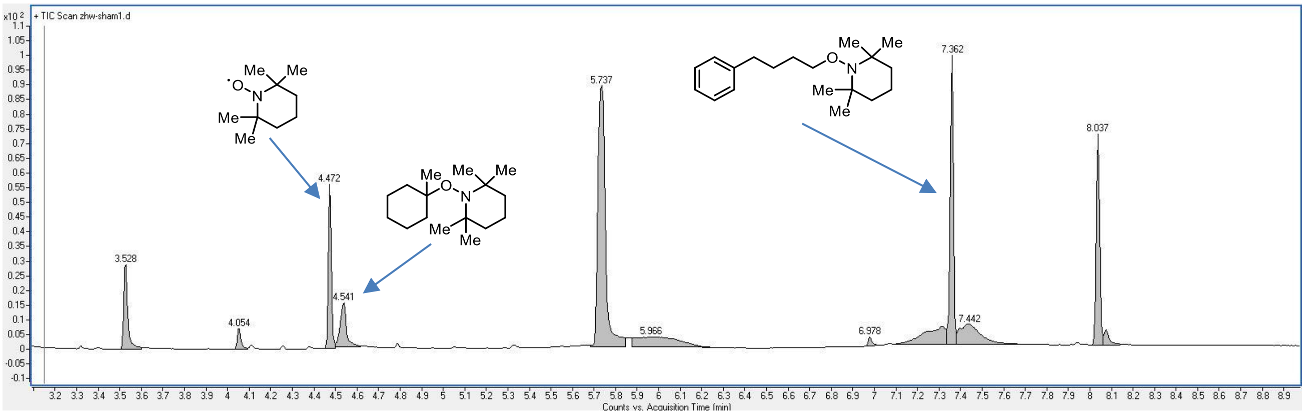

3.3. Radical clock experiment

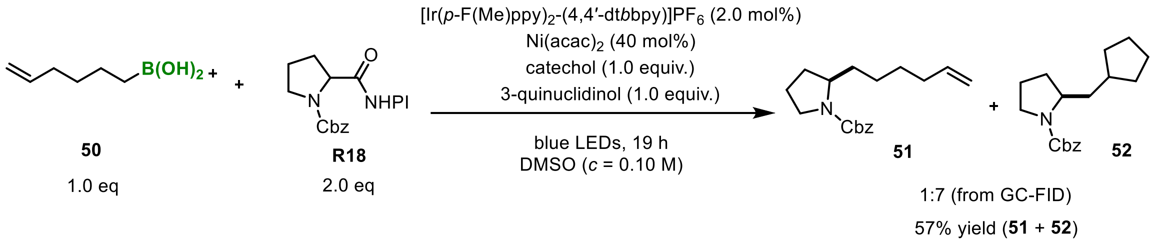

Under an ambient atmosphere, a flame dried 8 mL Biotage® microwave reaction vial equipped with a magnetic stir bar was transferred into an anhydrous, argon-filled glovebox and charged with [Ir(*p*-F(Me)ppy)$_2$-(4,4'-*d*tbbpy)]PF_6 (4.0 mg, 4.0 µmol, 2.0 mol%), Ni(acac)$_2$ (10 mg, 0.040 mmol, 0.20 equiv.), hex-5-en-1-ylboronic acid **50** (25.6 mg, 0.200 mmol, 1.00 equiv.), 1-benzyl 2-(1,3-dioxoisoindolin-2-yl) pyrrolidine-1,2-dicarboxylate **R18** (158 mg, 0.400 mmol, 2.00 equiv.), catechol (22 mg, 0.20 mmol, 1.0 equiv.), 3-quinuclidinol (25 mg, 0.20 mmol, 1.0 equiv.) sequentially, followed by the anhydrous DMSO (2.0 mL, *c* = 0.10 M). The vial was sealed with a cap with septum, removed from the glovebox. The mixture was pre-stirred for 10 min without irradiation until a homogeneous solution formed and subsequently positioned 3 cm from a blue LED light source (Kessil Tuna Blue LEDs). After stirring at 1200 rpm under irradiation for 19 h with fan cooling, the reaction mixture was diluted with wet EtOAc (1 mL), washed with deionized water (5 mL) and brine (5 mL), and extracted with EtOAc (3 × 10 mL). The combined organic layers were washed with brine (10 mL), dried over $MgSO_4$, filtered, and concentrated *in vacuo*. The r.r. (7:1, **52:51**) was determined by GC-FID analysis of the crude reaction mixture (see below). The crude residue was then purified by Biotage Isolera™ flash purification on silica gel, eluting with EtOAc/pentane (0–30%, v/v), affording a mixture of **51** and **52** (33 mg, 57%). This mixture was further separated using a TELEDYNE® preparative HPLC with EtOAc/pentane (0–30%, v/v) to yield pure **52** (20 mg, 35%) and **51** (4.0 mg, 6.0%) as colorless oils.

R*f* (56) = 0.6 (1:10 EtOAc/pentane, UV, $KMnO_4$)

NMR Spectroscopy of 51 (*see spectra*):

> **^{1}H NMR** (400 MHz, $CDCl_3$): δ_H 7.39 – 7.28 (m, 5H), 5.86 – 5.67 (m, 1H), 5.21 – 5.07 (m, 2H), 5.05 – 4.85 (m, 2H), 3.83 (s, 1H), 3.54 – 3.33 (m, 2H), 2.11 – 1.61 (m, 7H), 1.46 – 1.18 (m, 6H) ppm;
>
> **^{13}C NMR** (101 MHz, $CDCl_3$): δ_C 155.21 + 154.99 (amide rotamers), 139.12 + 139.00 (amide rotamers), 137.37 + 137.22 (amide rotamers), 128.6, 128.4, 128.0, 114.5, 66.78 + 66.53 (amide rotamers), 58.14 + 57.41 (amide rotamers), 46.72 + 46.41 (amide rotamers), 34.52 + 33.82 (amide rotamers), 30.72 + 29.96 (amide rotamers), 29.8, 29.01 + 28.90 (amide rotamers), 25.86 + 25.78 (amide rotamers), 23.97 + 23.20 (amide rotamers) ppm.

IR (film) ν_{max}: 2929, 1702, 1411, 1357, 1102, 912, 698 cm^{-1}.

HRMS (ESI$^+$): calcd. for $C_{18}H_{29}O_2N$ [M+H]$^+$, 288.1958, found 288.1959.

R*f* (57) = 0.6 (1:10 EtOAc/pentane, UV, $KMnO_4$)

NMR Spectroscopy of 52 (*see spectra*):

> **^{1}H NMR** (500 MHz, $CDCl_3$): δ_H 7.42 – 7.27 (m, 5H), 5.19 – 5.06 (m, 2H), 4.00 – 3.77 (m, 1H), 3.52 – 3.33

(m, 2H), 1.98 – 1.40 (m, 12H), 1.39 – 0.97 (m, 3H) ppm;

^{13}C NMR (126 MHz, $CDCl_3$): δ_C 155.0, 137.2, 128.5, 128.37 + 128.36 (amide rotamers), 128.0, 66.78 + 66.53 (amide rotamers), 57.48 + 56.92 (amide rotamers), 46.36, 41.11 + 40.40 (amide rotamers), 37.5, 33.7, 32.0, 30.72 + 30.11 (amide rotamers), 25.3, 25.2, 23.91 + 23.18 (amide rotamers) ppm.

All recorded spectroscopic data matched those previously reported in the literature.[3]

GC-FID trace of radical clock experiment:

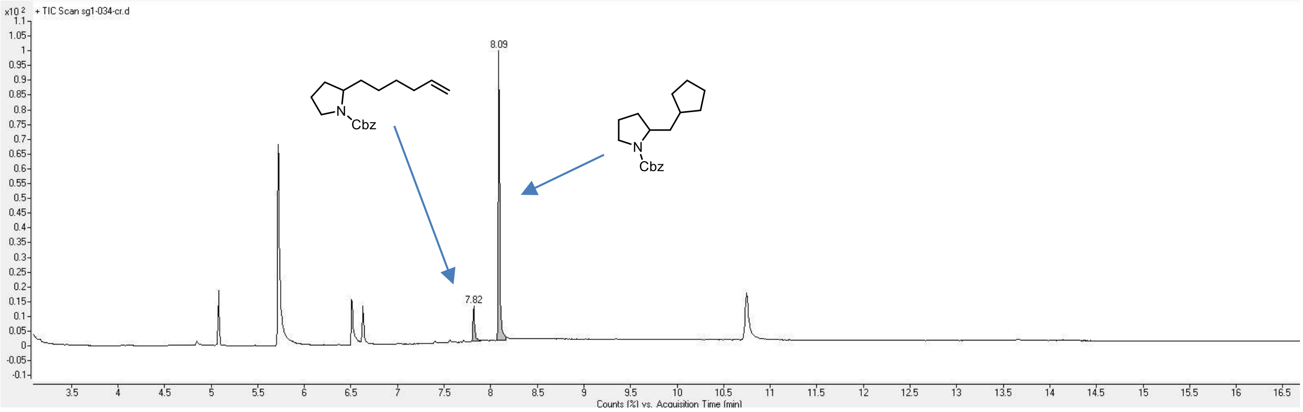

3.4. Stern-Volmer experiments

The visible light luminescence intensities were recorded using a FluoroMax spectrofluorometer. All luminescence measurements were recorded using a screw-top quartz cuvette (10 × 10 mm, 3.5 mL). All solutions of [Ir(*d*F(Me)ppy)$_2$(*d*tbbpy)]PF_6 (200 mg, 100 μmol), (4-phenylbutyl)boronic acid **46** (17.8 mg, 100 μmol), 3-quinuclidinol (12.7 mg, 100 μmol), catechol (11.0 mg, 100 μmol) and redox-active ester (28.7 mg, 100 μmol) were prepared in DMSO (10 mL, *c* = 10 mM), in an argon-filled glovebox. The solutions were transferred to a screw-top cuvette, sealed, and then brought out of the glovebox for visible light luminescence measurements.

In a typical procedure, (4-phenylbutyl)boronic acid **46** (17.8 mg, 100 μmol) was dissolved in 10 mL anhydrous DMSO (10 mL, *c* = 10 mM) as boronic acid quencher solution. Next 150 μL boronic acid quencher solution was transferred to the cuvette and diluted by anhydrous DMSO to 3 mL, 30 μL stock solution (*c* = 10 μM [Ir(*d*F(Me)ppy)$_2$(*d*tbbpy)]PF_6) was added to get a test sample (*c* = 0.3 μM [Ir(*d*F(Me)ppy)$_2$(*d*tbbpy)]PF_6) with boronic acid **46** (*c* = 15 μM 50 equiv.) as quencher (Figure **S5**, 50 equiv.). The cuvettes were then removed from the glovebox and sealed with parafilm for analysis. All subsequent solutions were prepared by diluting to the volume of 3 mL with same quartz cuvette. All solutions were excited at 380 nm, and the emission was measured from 400 to 700 nm. The fluorescence emission was recorded at 520 nm.

Quenching was analyzed by plotting I_0/I according to the Stern-Volmer relationship:

$I_0/I = k_q\tau_0[Q]+1$

where I_0 represents the integral of the luminescence over the range of 455 to 600 nm in the absence of a quencher, I is the integral of luminescence over the range of 455 to 600 nm in the presence of a quencher, k_q represents the quenching rate constant, [Q] is the concentration of a given quencher, and τ_0 is the excited state lifetime of the emissive photocatalyst in the absence of quencher.

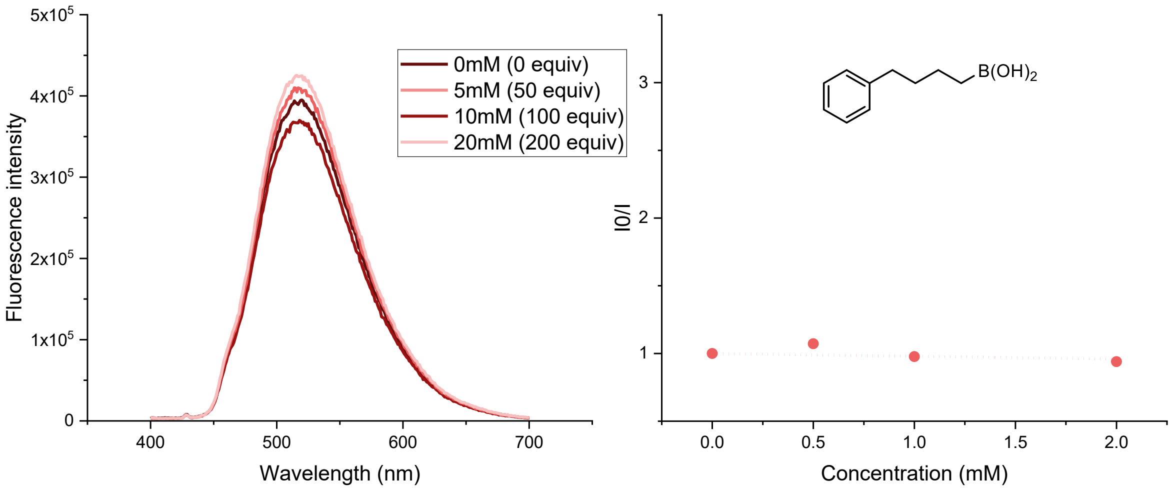

Figure S5. Fluorescence emission quenching of [Ir(*d*F(Me)ppy)$_2$(*d*tbbpy)]PF_6 (10µM) in DMSO using (4-phenylbutyl)boronic acid as quencher (left) and relative Stern-Volmer plot (right) of fluorescence intensity ratio I_0/I as a function of quencher concentration.

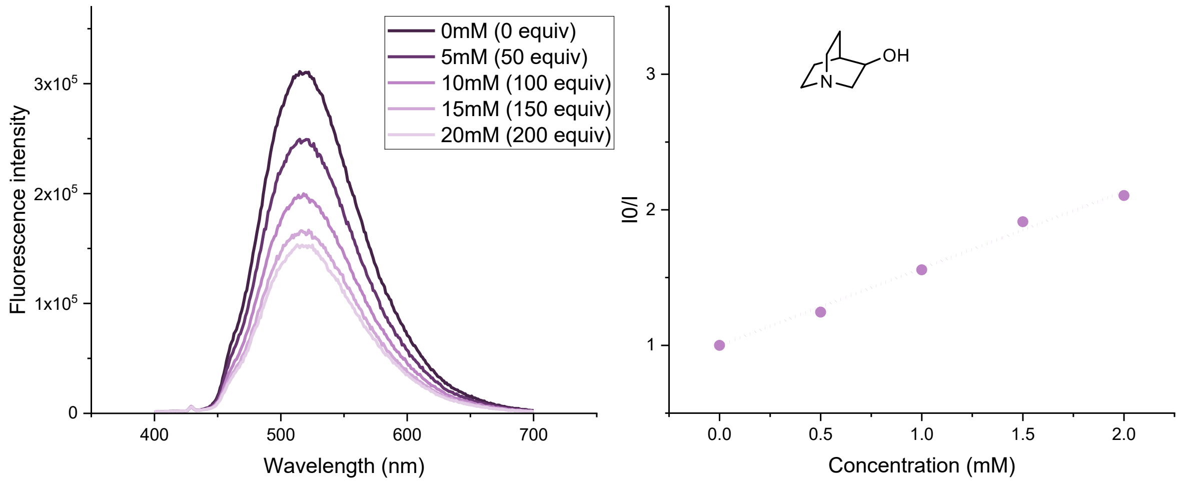

Figure S6. Fluorescence emission quenching of [Ir(*d*F(Me)ppy)$_2$(*d*tbbpy)]PF_6 (10µM) in DMSO using 3-quinuclidinol as quencher (left) and relative Stern-Volmer plot (right) of fluorescence intensity ratio I_0/I as a function of quencher concentration.

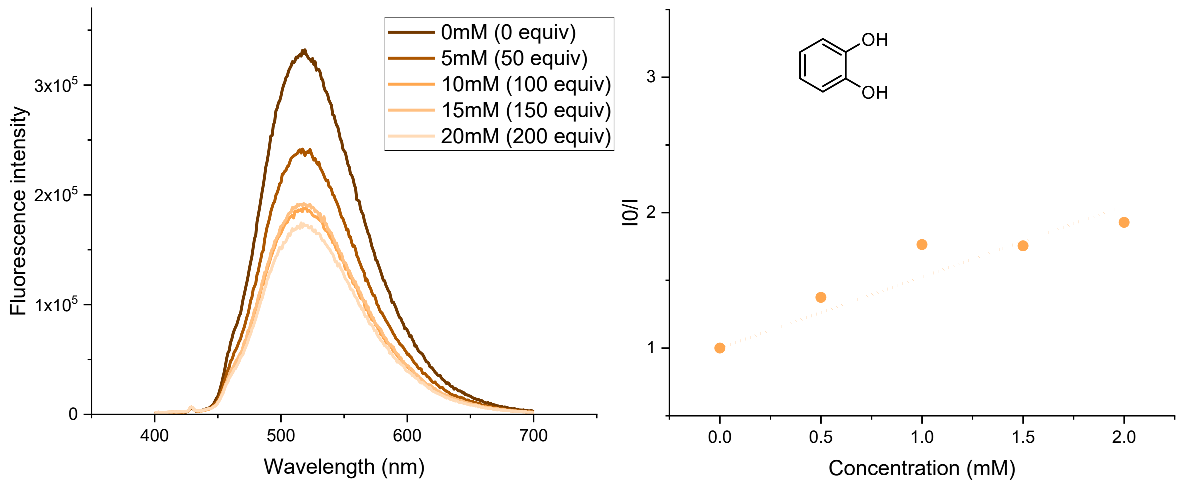

Figure S7. Fluorescence emission quenching of [Ir(*d*F(Me)ppy)$_2$(*d*tbbpy)]PF$_6$ (10μM) in DMSO using catechol as quencher (left) and relative Stern-Volmer plot (right) of fluorescence intensity ratio I_0/I as a function of quencher concentration.

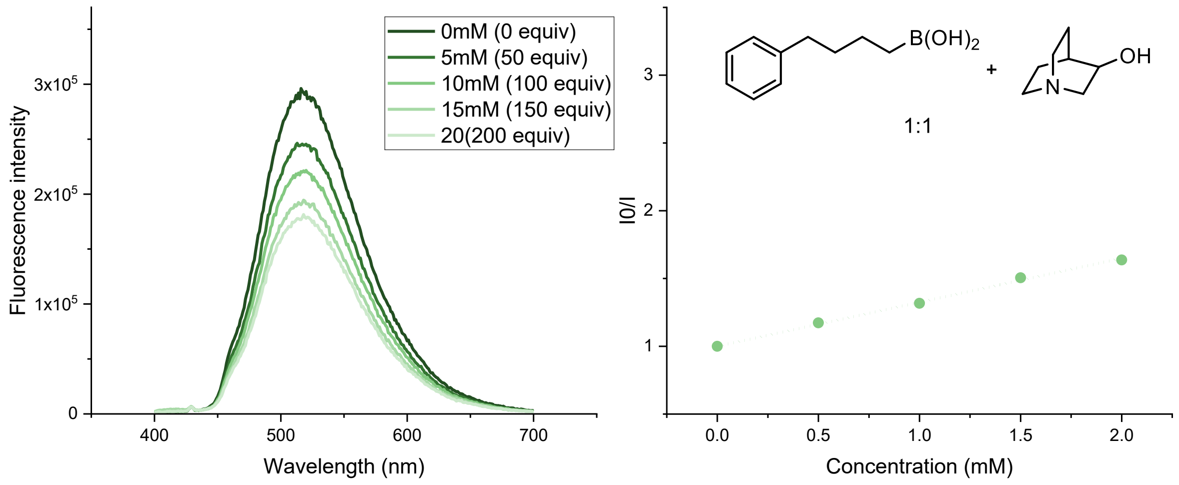

Figure S8. Fluorescence emission quenching of [Ir(*d*F(Me)ppy)$_2$(*d*tbbpy)]PF$_6$ (10μM) in DMSO using a 1:1 mixture of (4-phenylbutyl)boronic acid and 3-quinuclidinol as quencher (left) and relative Stern-Volmer plot (right) of fluorescence intensity ratio I_0/I as a function of quencher concentration.

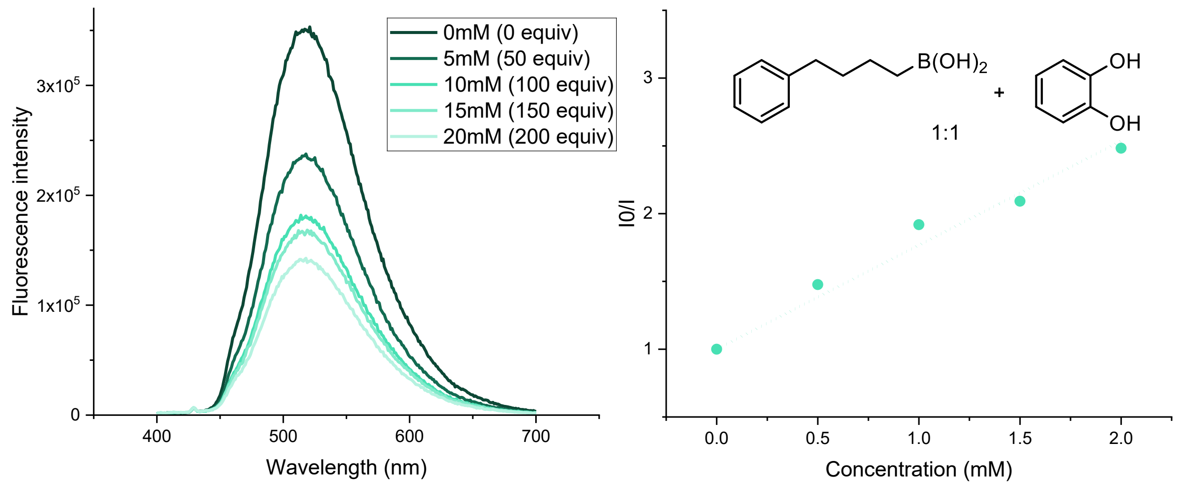

Figure S9. Fluorescence emission quenching of [Ir(*d*F(Me)ppy)$_2$(*d*tbbpy)]PF$_6$ (10µM) in DMSO using a 1:1 mixture of (4 -phenylbutyl)boronic acid and catechol as quencher (left) and relative Stern-Volmer plot (right) of fluorescence intensity ratio I$_0$/I as a function of quencher concentration.

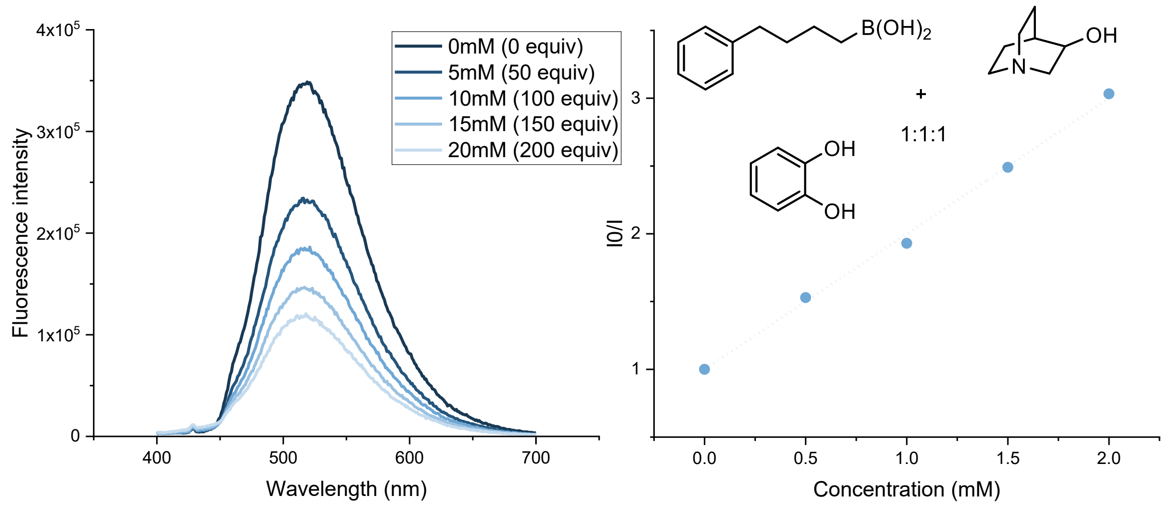

Figure S10. Fluorescence emission quenching of [Ir(*d*F(Me)ppy)$_2$(*d*tbbpy)]PF$_6$ (10µM) in DMSO using a 1:1:1 mixture of (4 -phenylbutyl)boronic acid, 3-quinuclidinol and catechol as quencher (left) and relative Stern-Volmer plot (right) of fluorescence intensity ratio I$_0$/I as a function of quencher concentration.

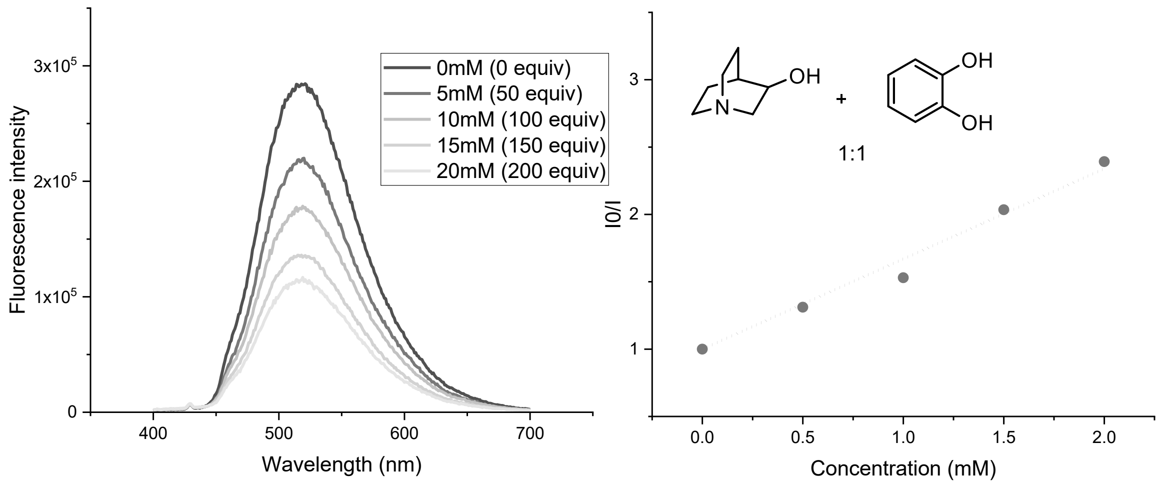

Figure S11. Fluorescence emission quenching of [Ir(*d*F(Me)ppy)$_2$(*d*tbbpy)]PF$_6$ (10μM) in DMSO using a 1:1 mixture of 3-quinuclidinol and catechol as quencher (left) and relative Stern-Volmer plot (right) of fluorescence intensity ratio I_0/I as a function of quencher concentration.

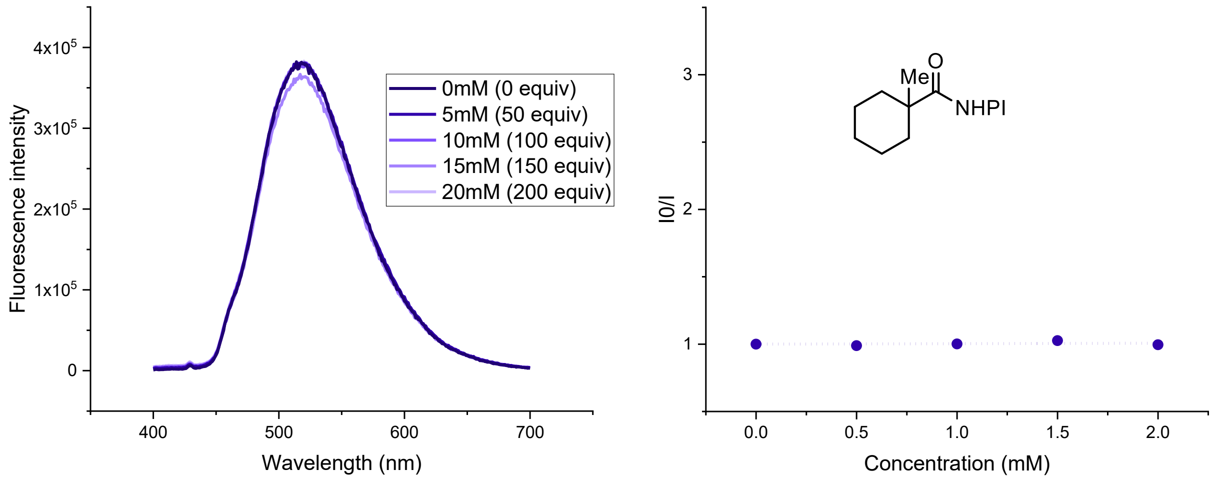

Figure S12. Fluorescence emission quenching of [Ir(*d*F(Me)ppy)$_2$(*d*tbbpy)]PF$_6$ (10μM) in DMSO using NHPI redox-active ester as quencher (left) and relative Stern-Volmer plot (right) of fluorescence intensity ratio I_0/I as a function of quencher concentration.

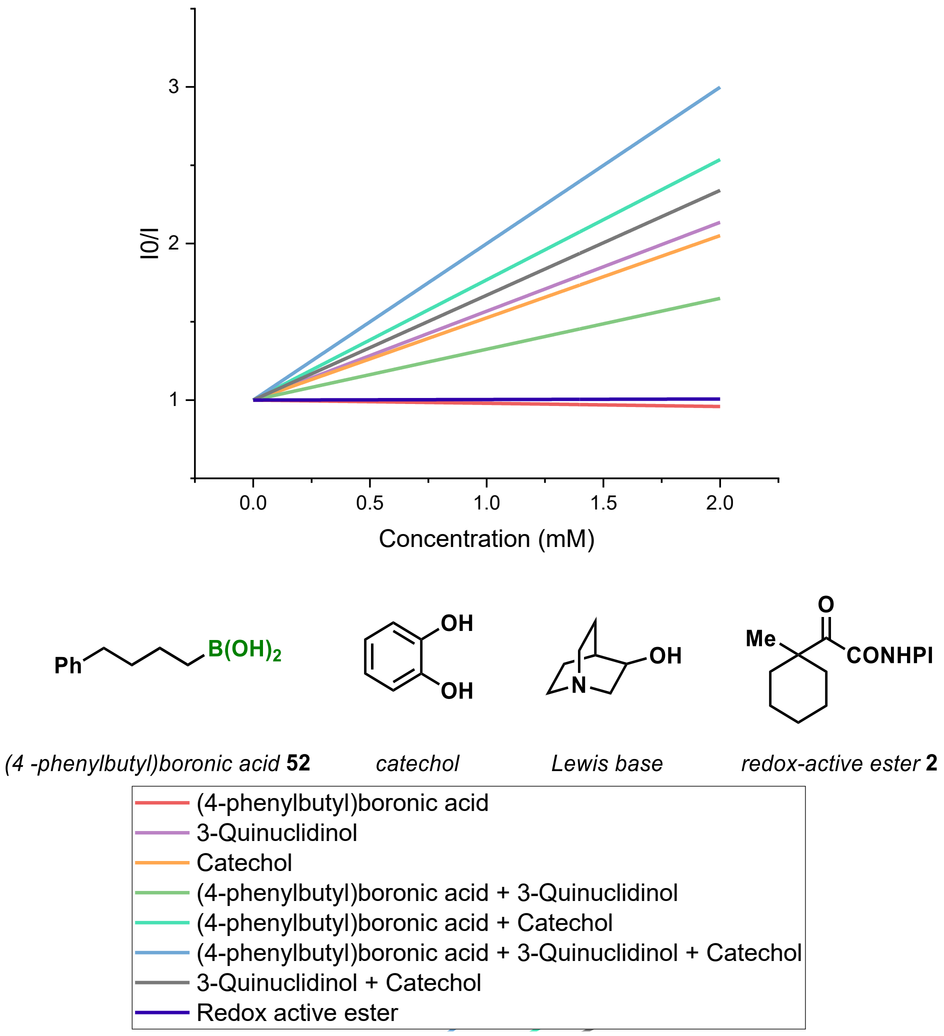

Figure S13. Comparison of the linear fittings of all the Stern-Volmer plots.

3.5. Probing intermediacy of low-valent Ni species

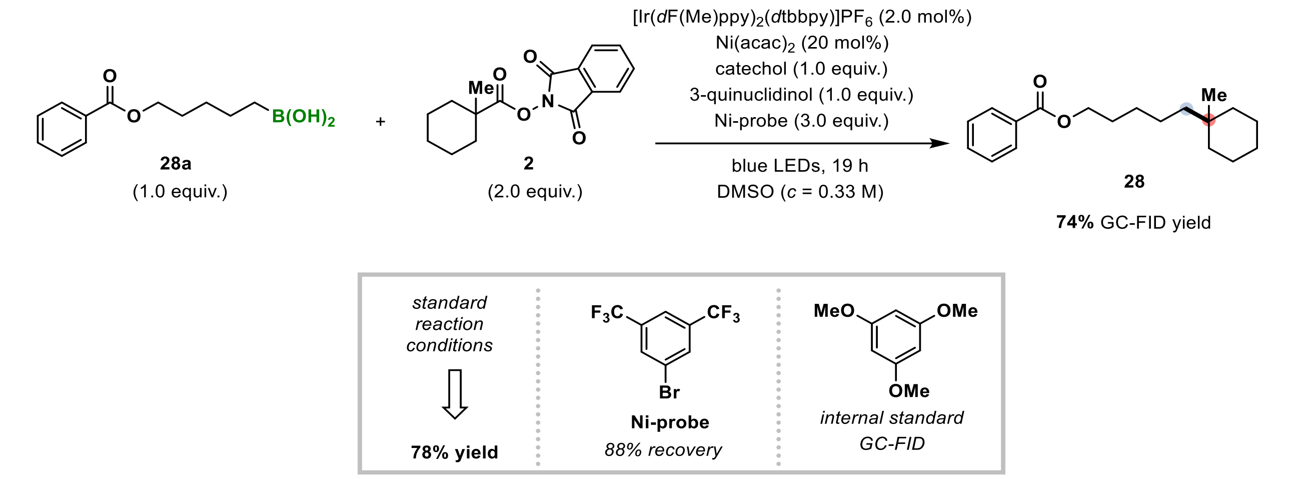

Prepared following **General Procedure A** with 3,6-bis(trifluoromethyl)bromobenzene (88 mg, 0.30 mmol, 3.0 equiv.) added before photo irradiation as low-valent Ni species probe. After reaction, 1,2,3-trimethoxybenzene (17 mg, 0.10 mmol, 1.0 equiv.) was added as internal standard for GC-MS analysis. The yield of cross-coupling products (74%, response factor δ = 0.41, see below) and the recovery of Ni probe (88%, response factor δ = 1.12, see below) were determined by GC-FID analysis.

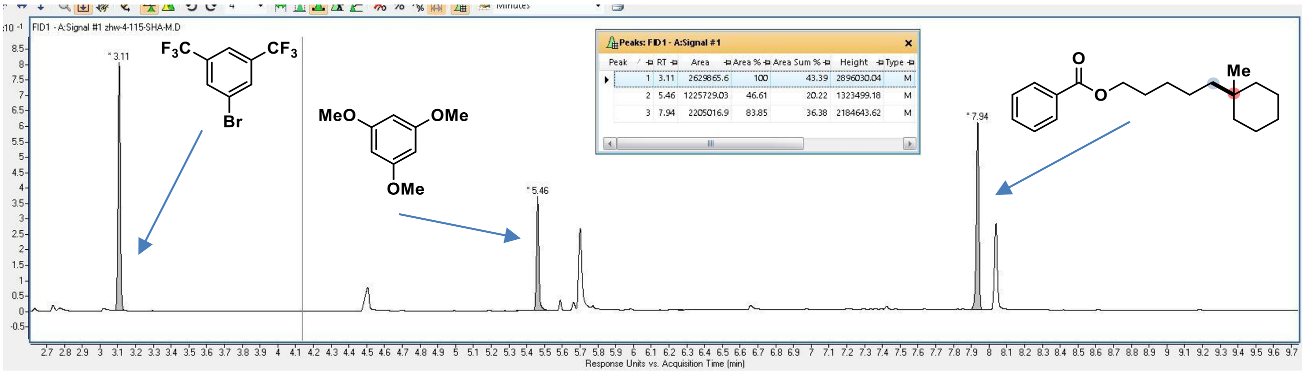

4. NMR SPECTROSCOPIC DATA

^{1}H NMR (400 MHz, $CDCl_3$) of **3** (***see procedure***)

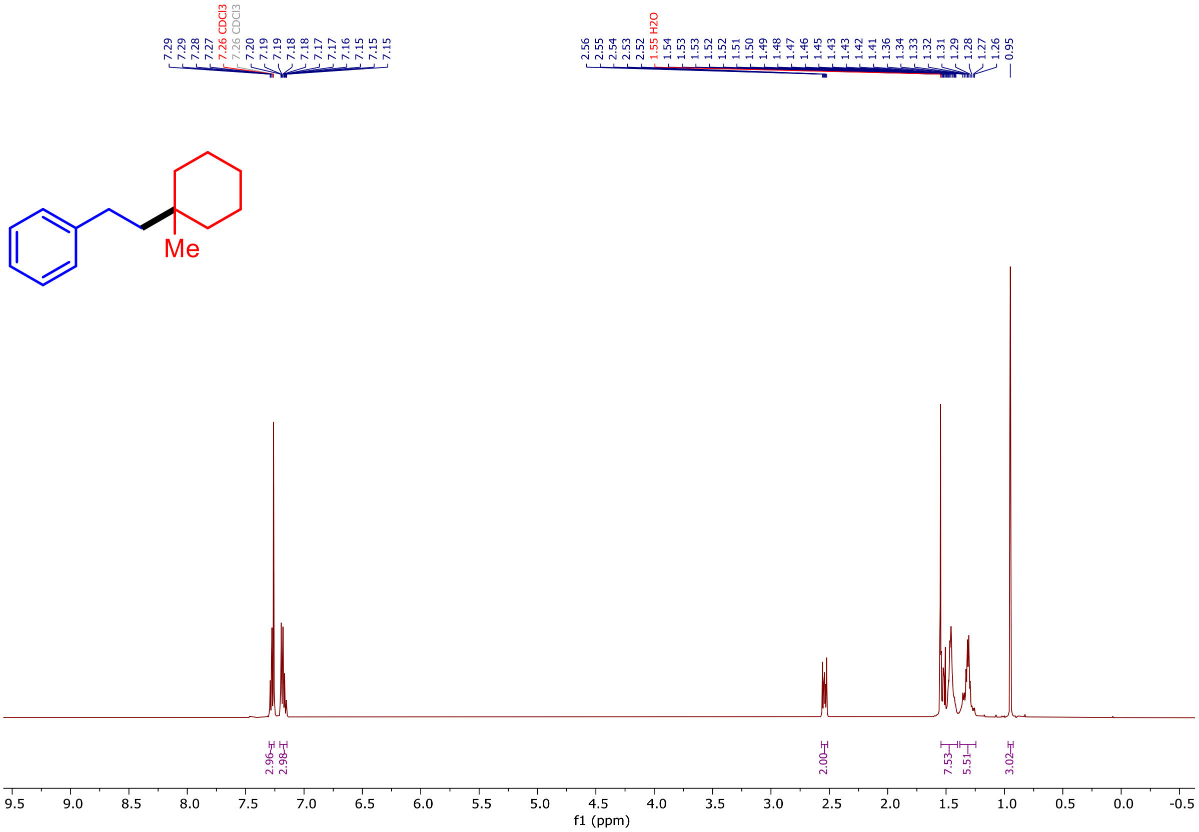

^{13}C NMR (101 MHz, $CDCl_3$) of **3**

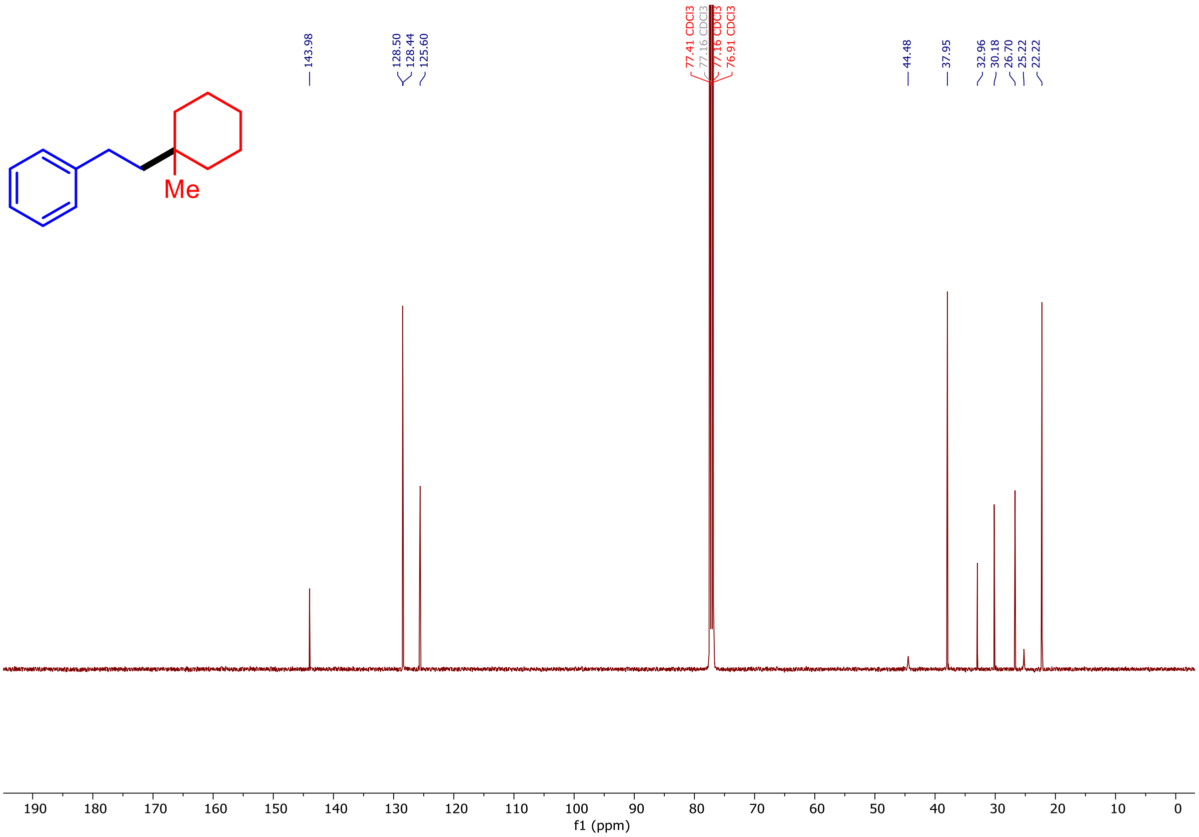

^{1}H NMR (400 MHz, $CDCl_3$) of **4** (***see procedure***)

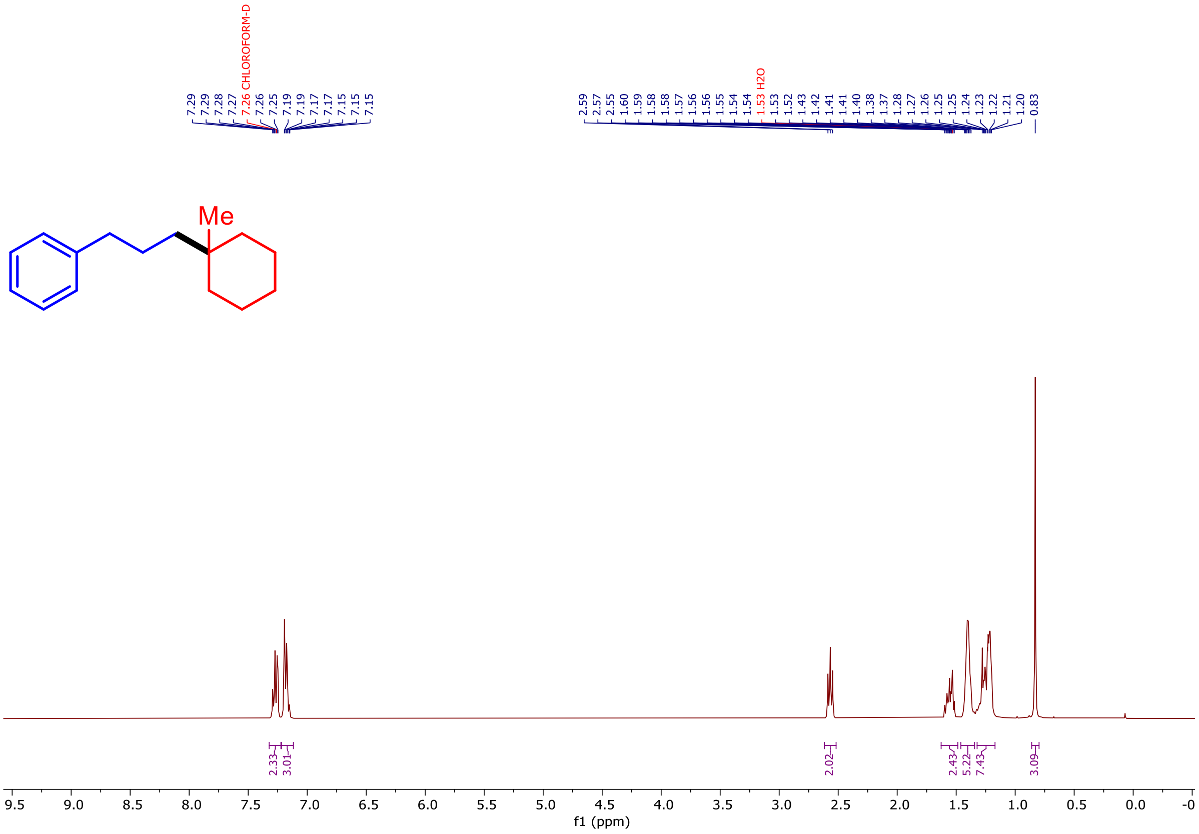

^{13}C NMR (101 MHz, $CDCl_3$) of **4**

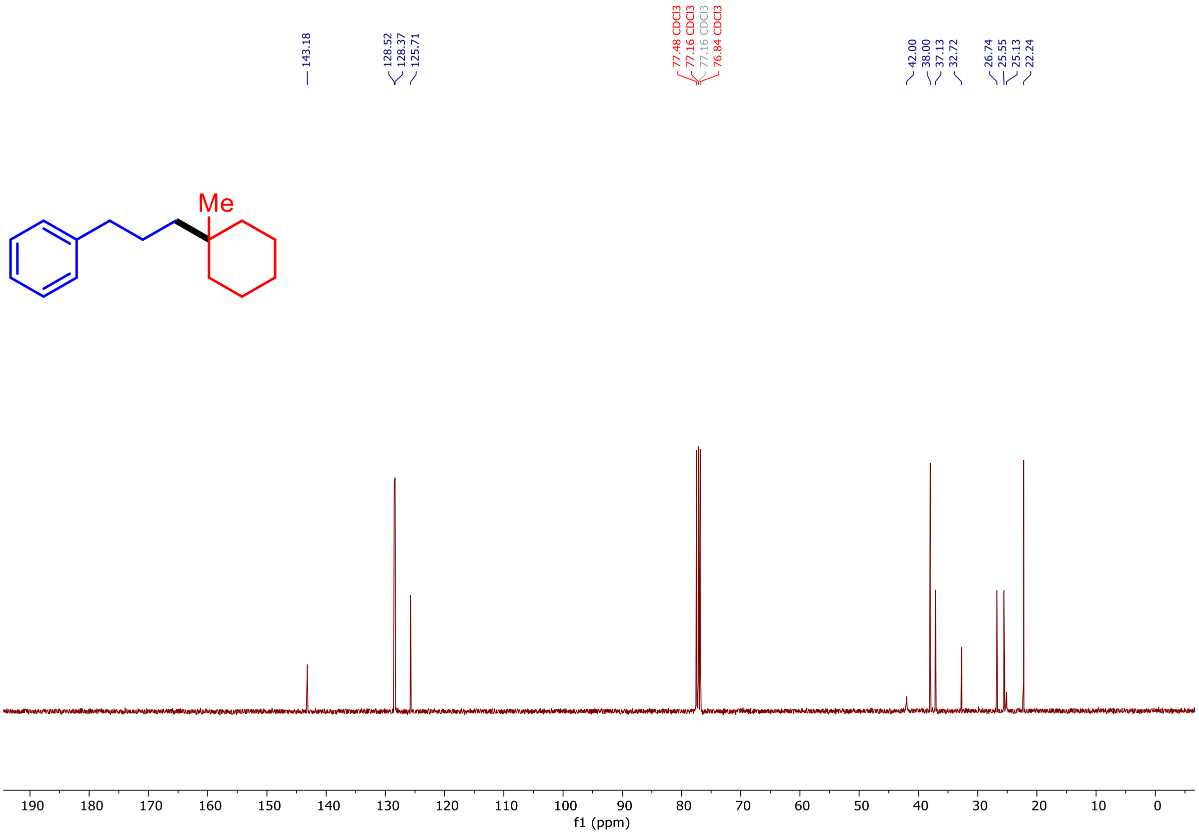

^{1}H NMR (400 MHz, $CDCl_3$) of **5** (*see procedure*)

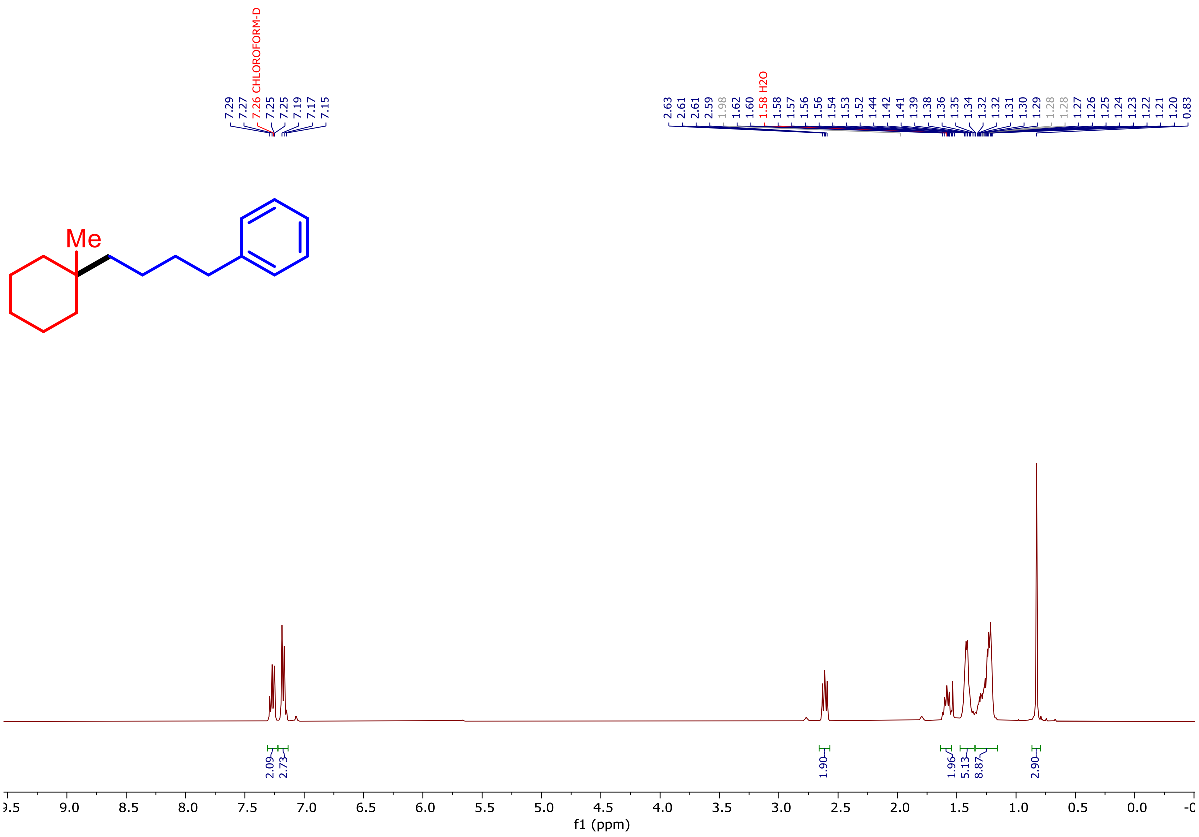

^{13}C NMR (101 MHz, $CDCl_3$) of **5**

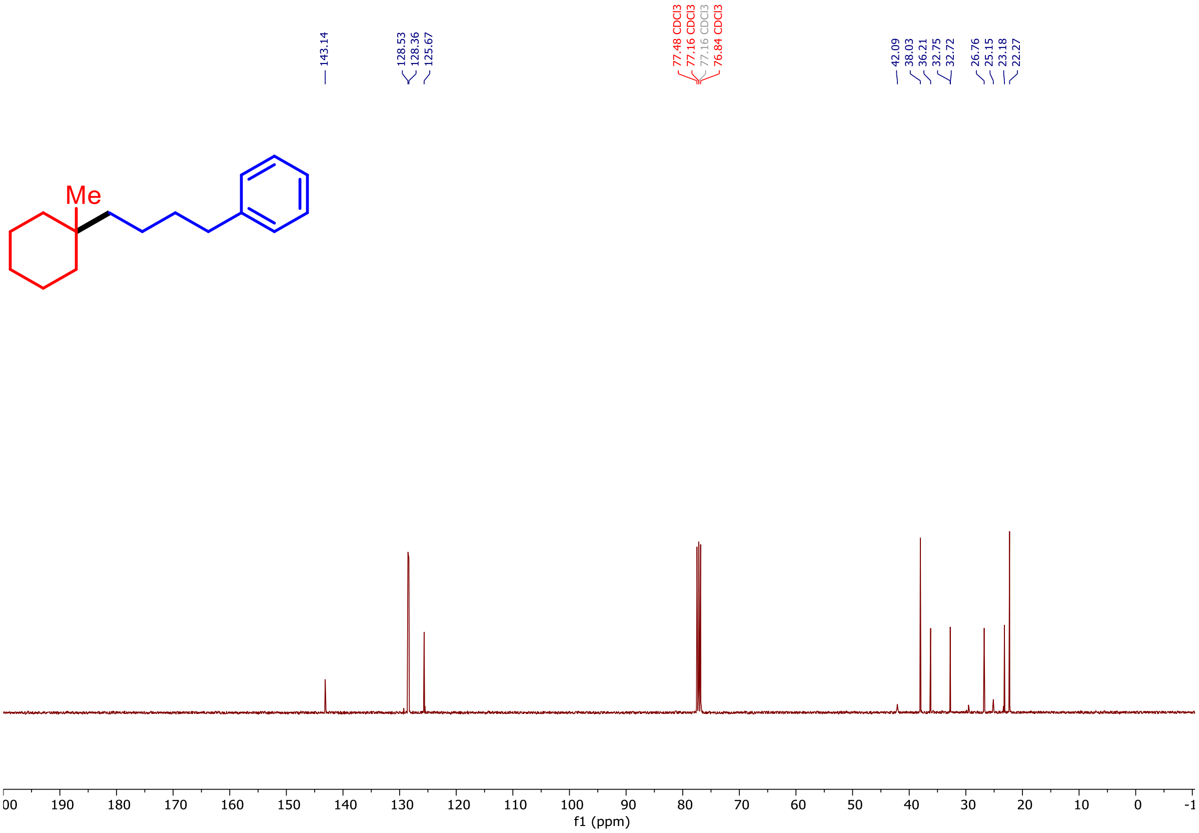

^{1}H NMR (400 MHz, $CDCl_3$) of **6** (*see procedure*)

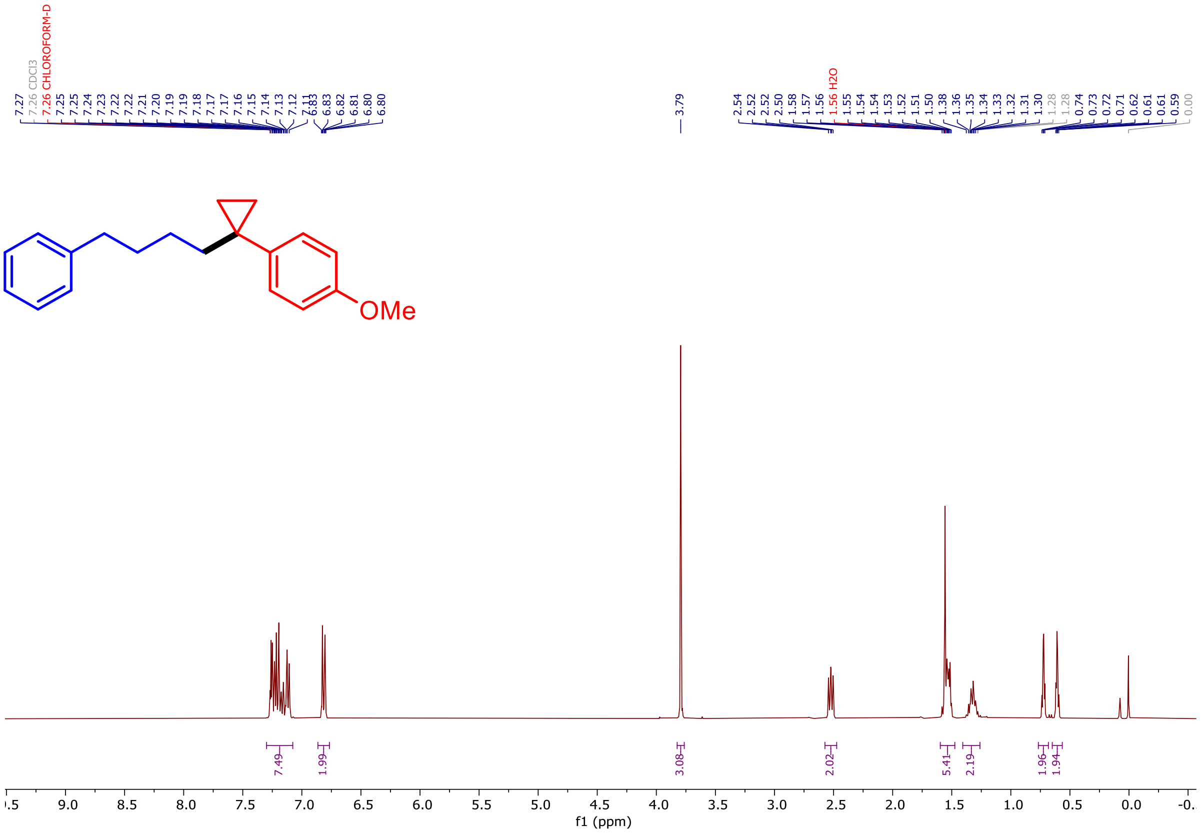

^{13}C NMR (101 MHz, $CDCl_3$) of **6**

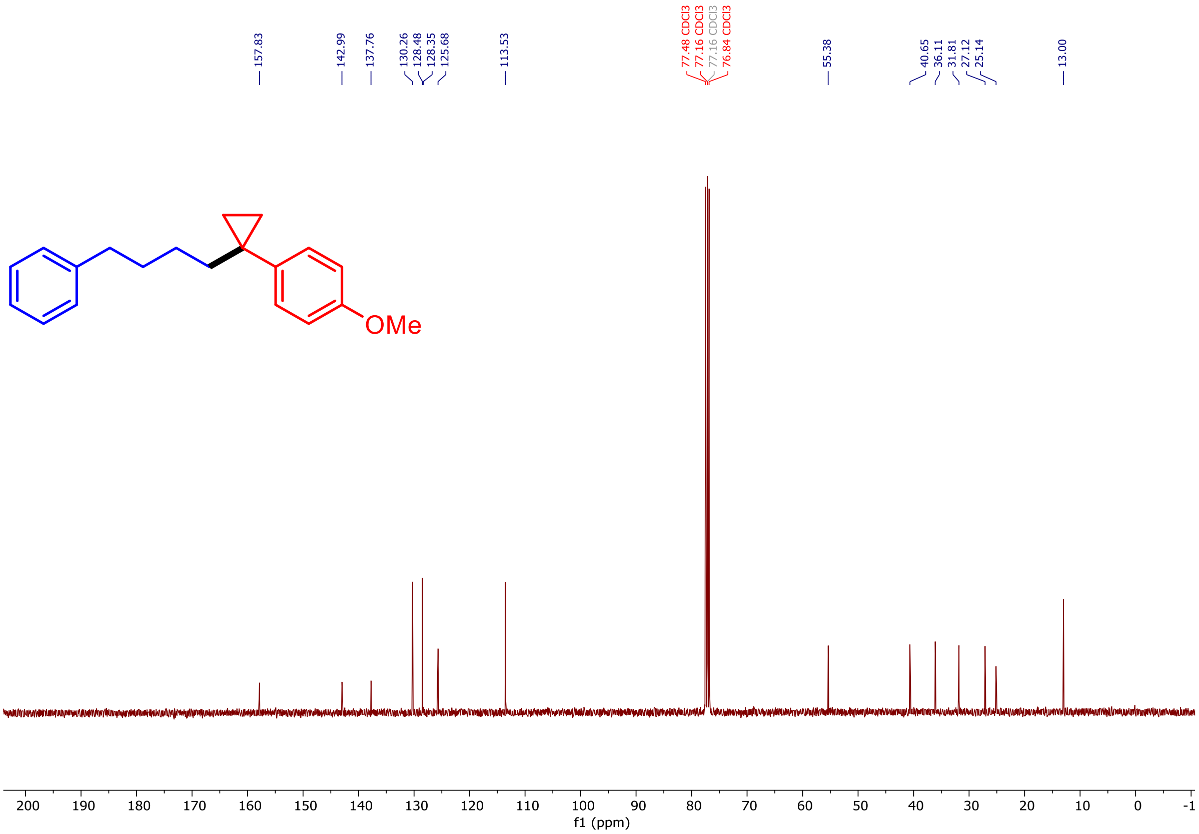

^{1}H NMR (400 MHz, $CDCl_3$) of **7** (*see procedure*)

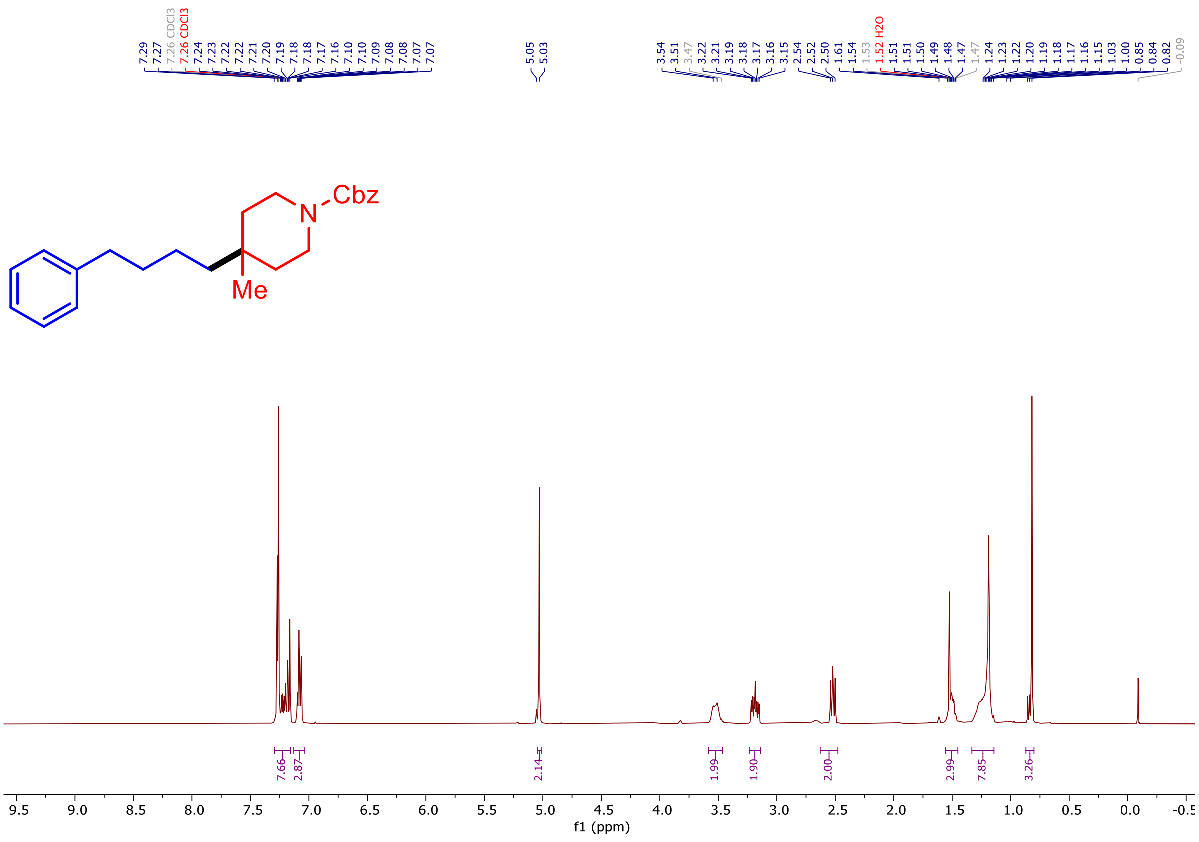

^{13}C NMR (101 MHz, $CDCl_3$) of **7**

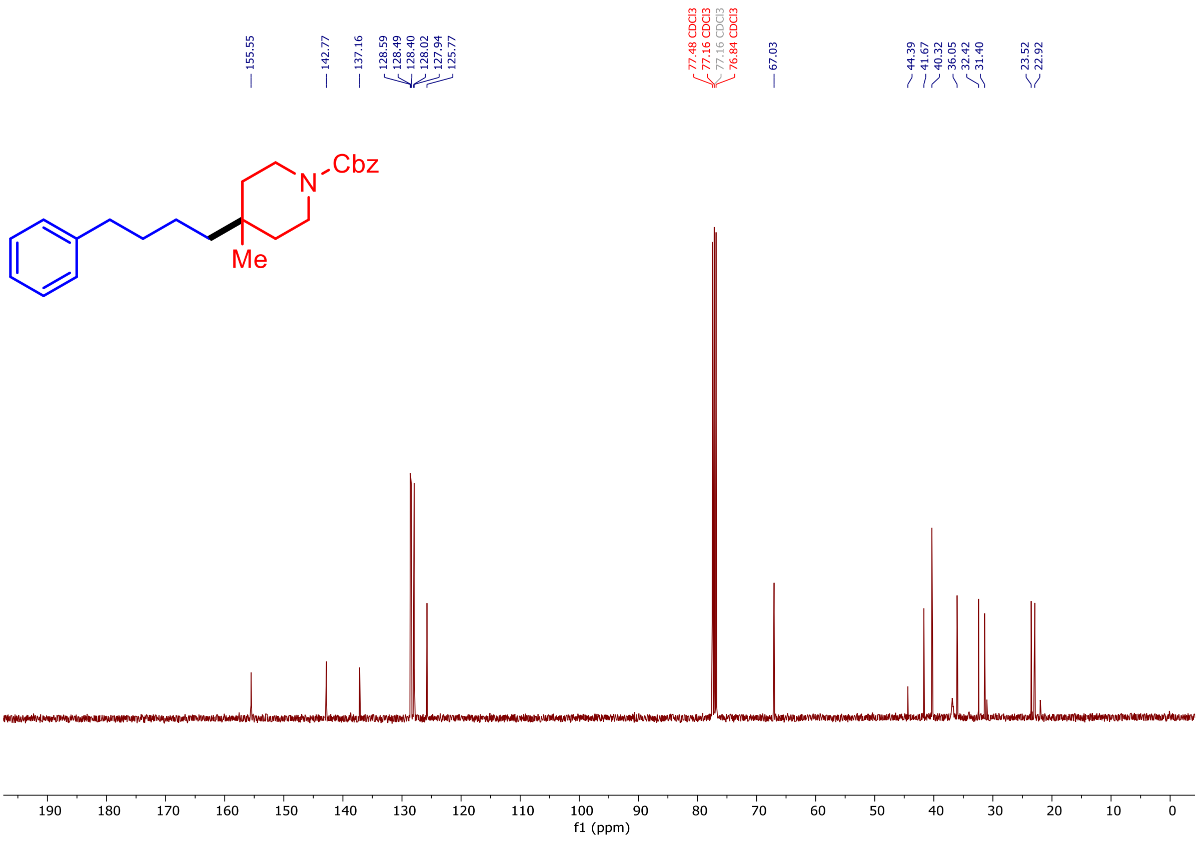

^{1}H NMR (400 MHz, $CDCl_3$) of **8** (*see procedure*)

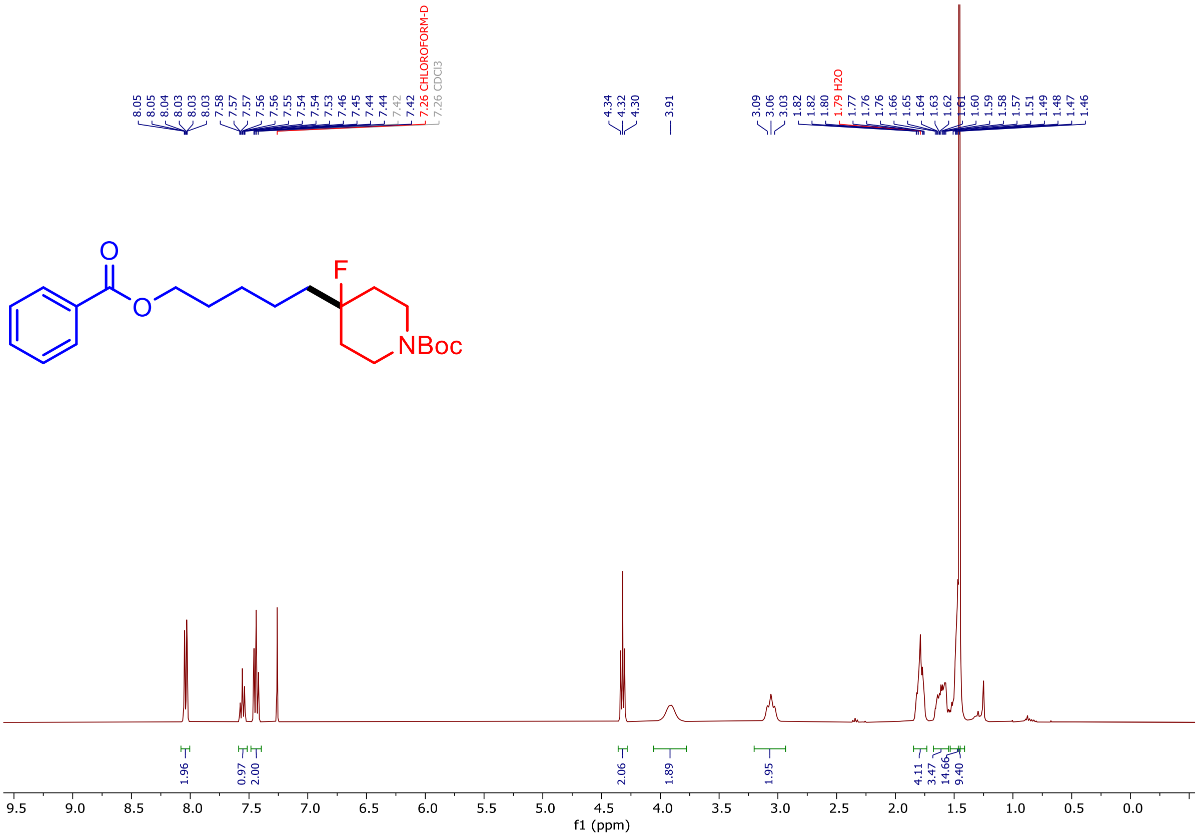

^{13}C NMR (101 MHz, $CDCl_3$) of **8**

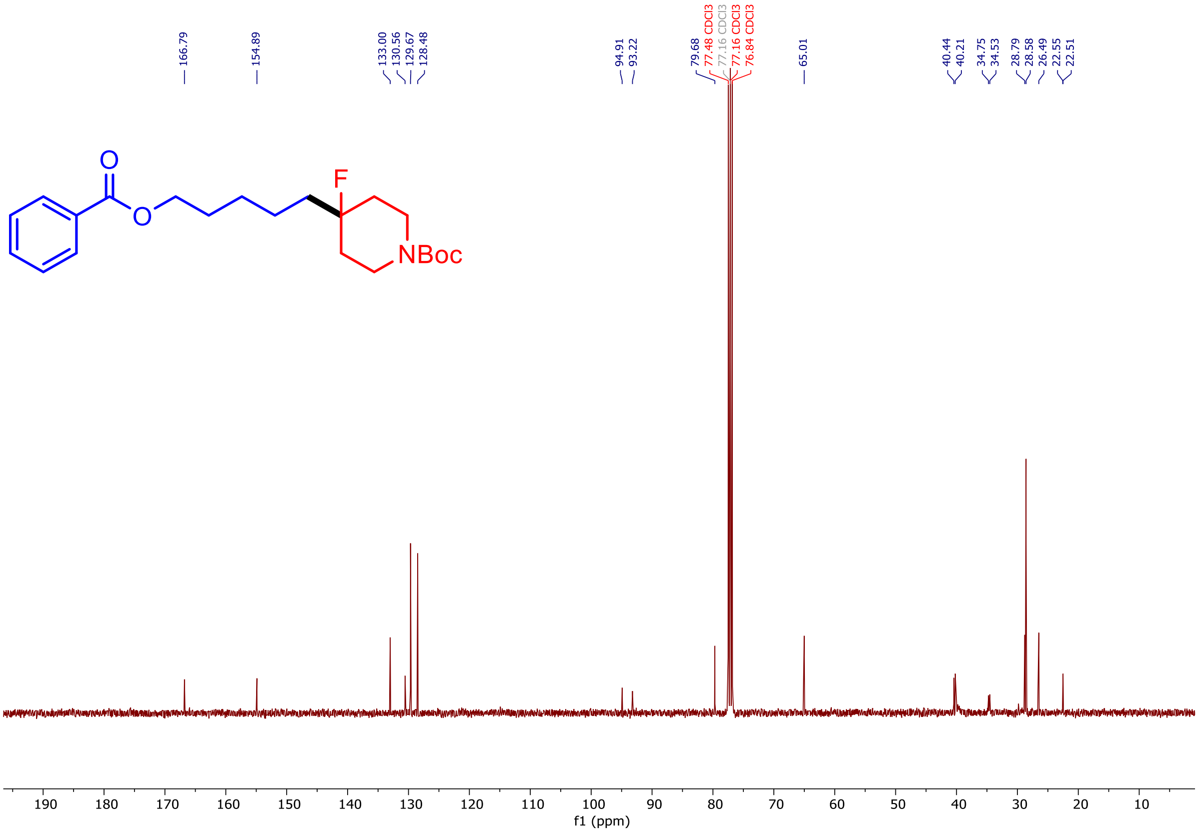

^{19}F NMR (377 MHz, $CDCl_3$) of **8**

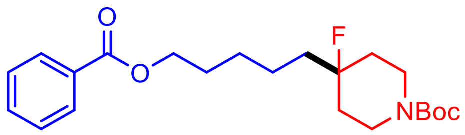

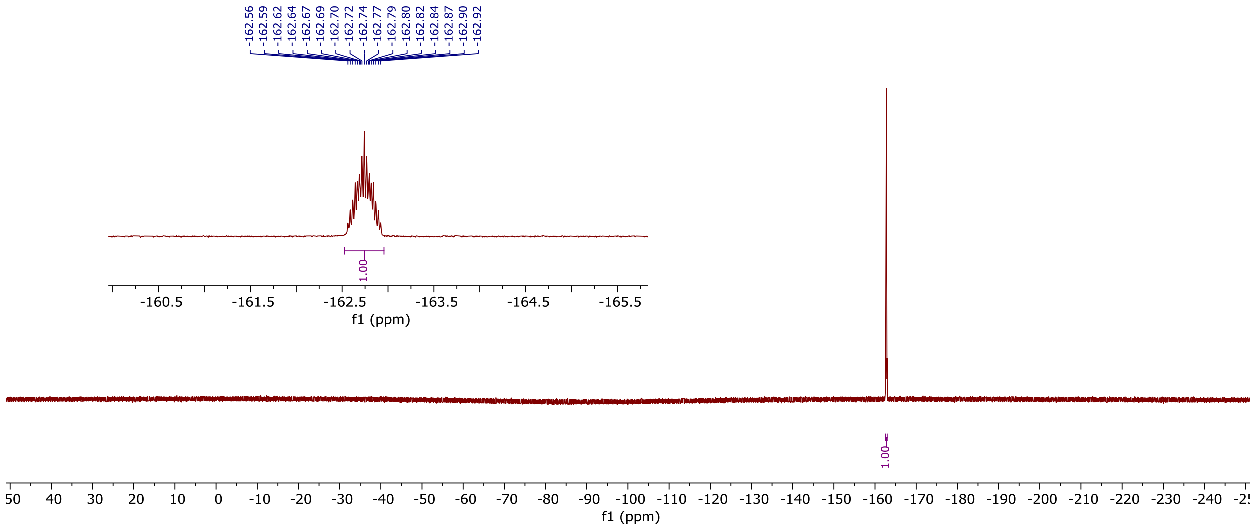

^{1}H NMR (400 MHz, $CDCl_3$) of **9** (***see procedure***)

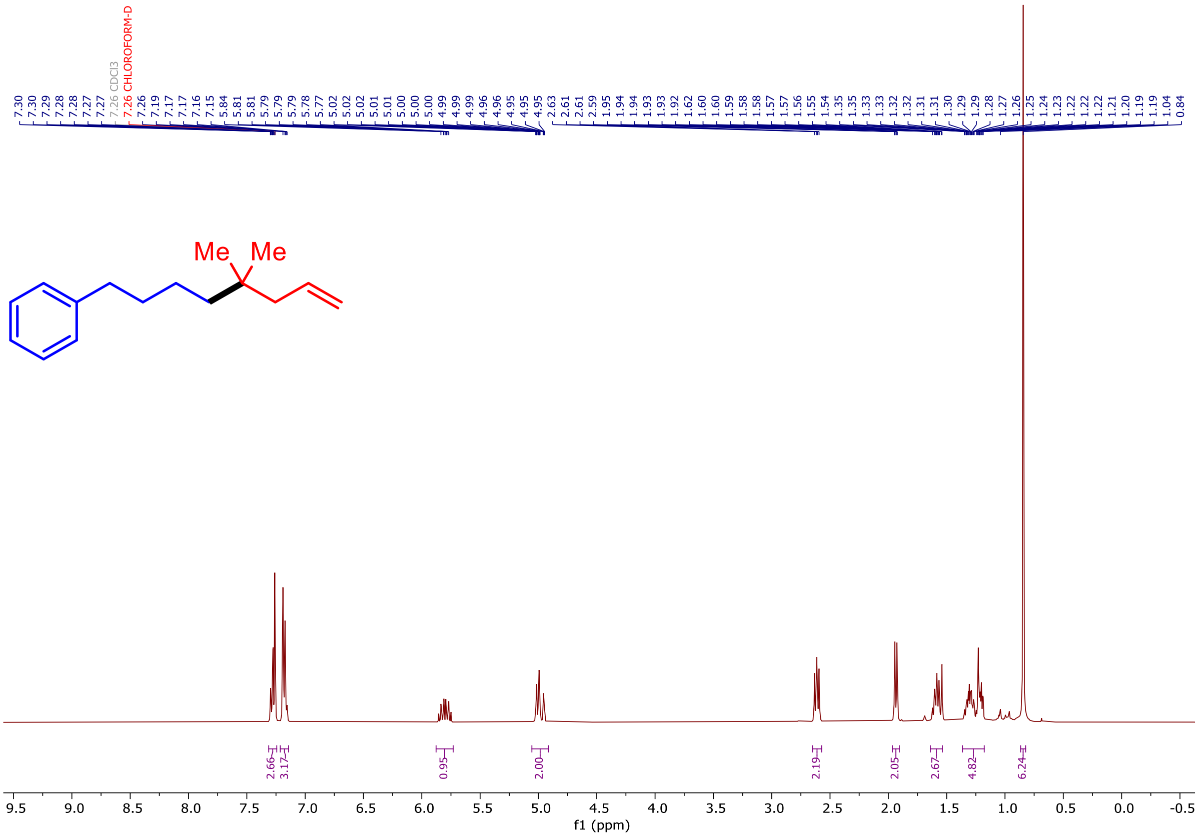

^{13}C NMR (101 MHz, $CDCl_3$) of **9**

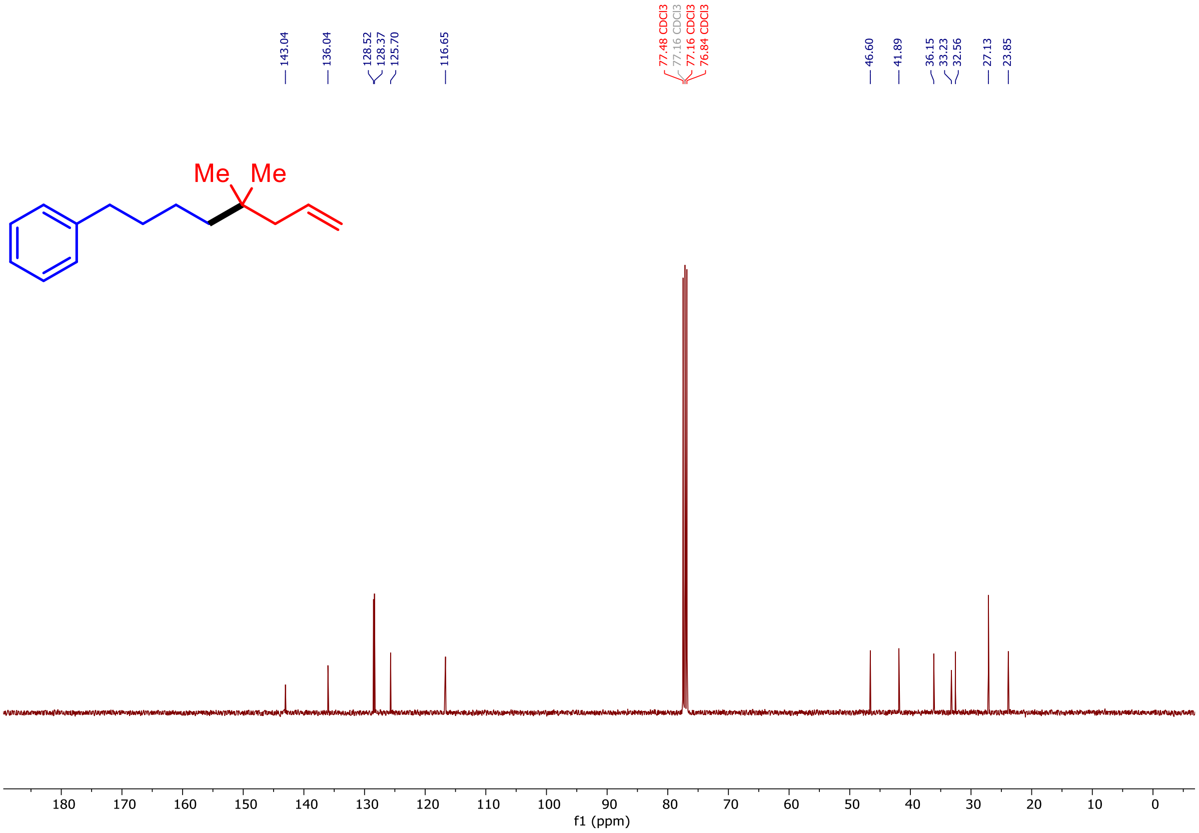

^{1}H NMR (400 MHz, $CDCl_3$) of **10** (*see procedure*)

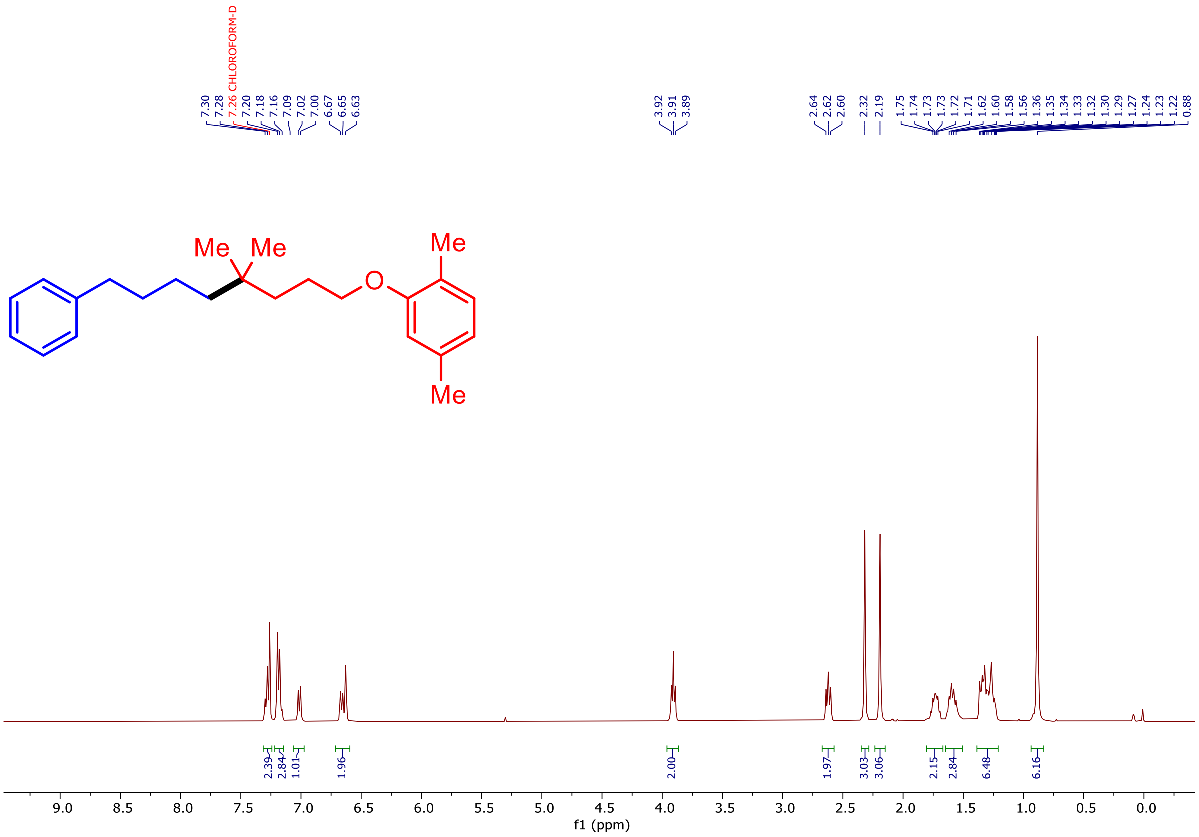

^{13}C NMR (101 MHz, $CDCl_3$) of **10**

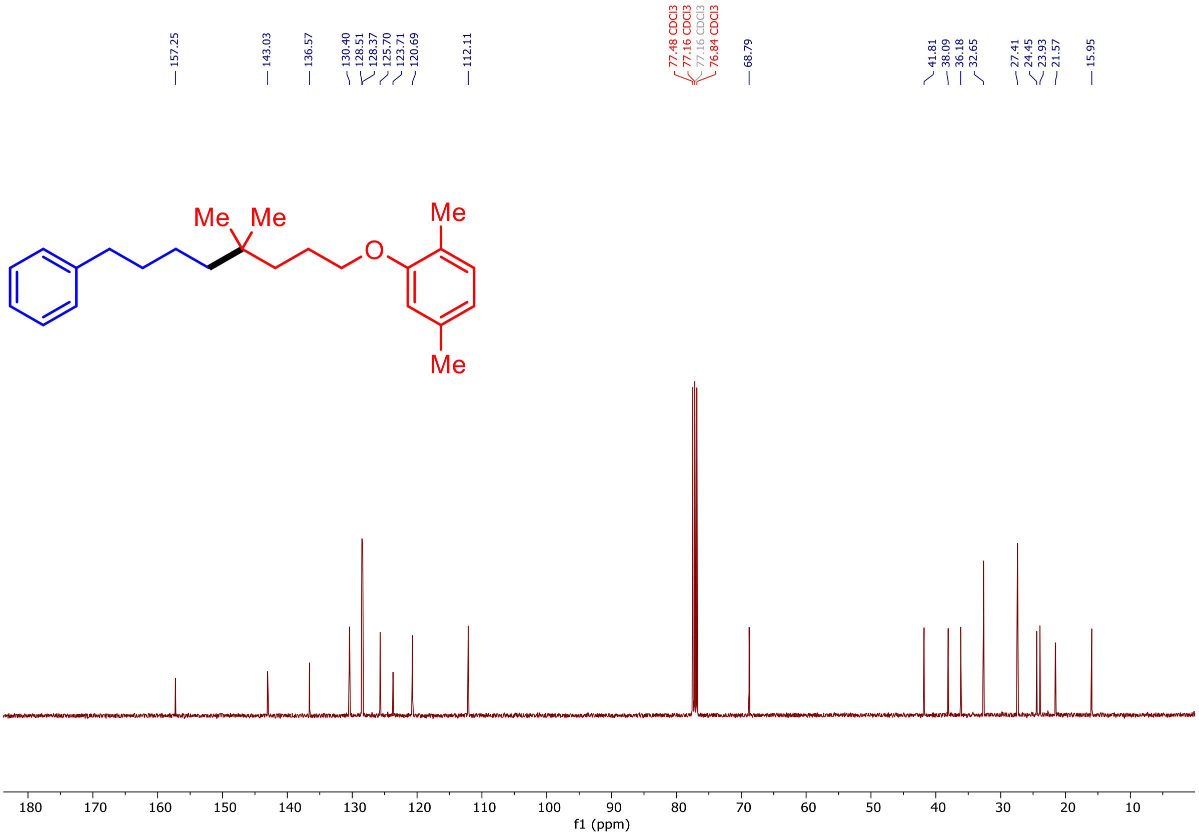

^{1}H NMR (400 MHz, $CDCl_3$) of **11** (*see procedure*)

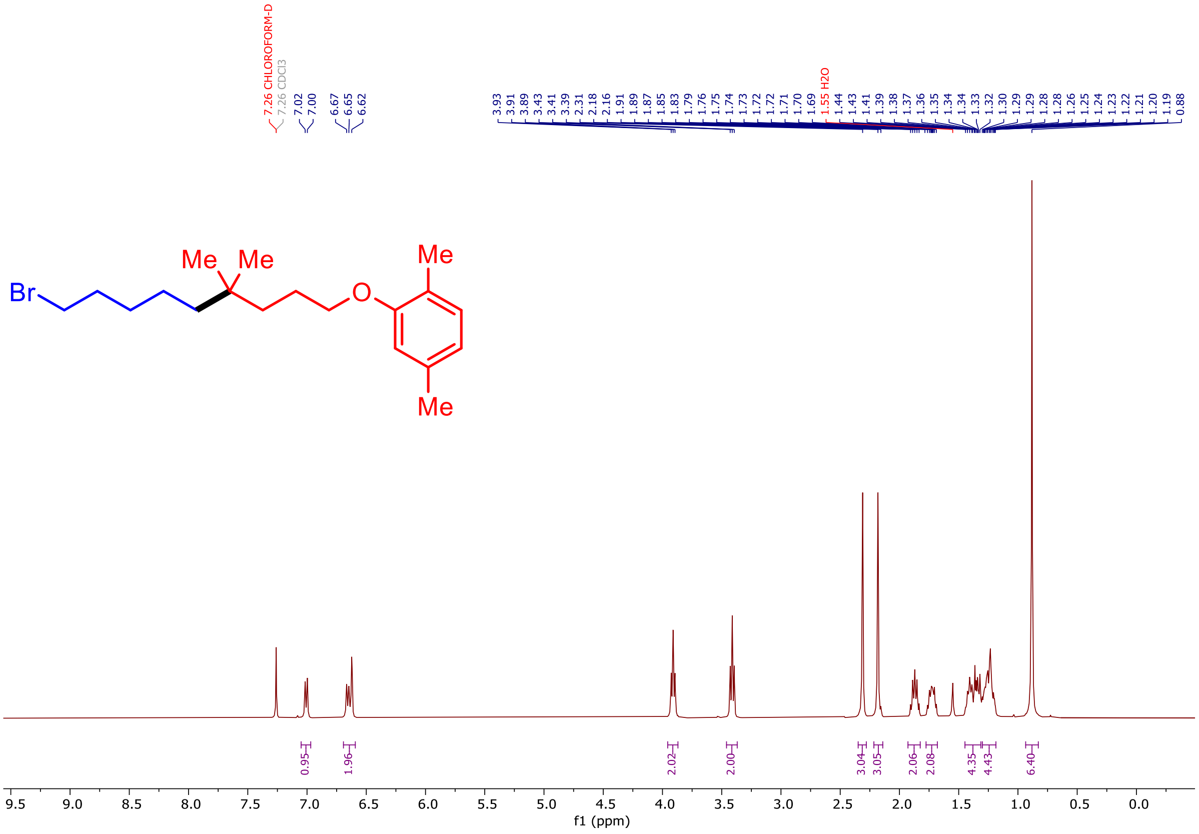

^{13}C NMR (101 MHz, $CDCl_3$) of **11**

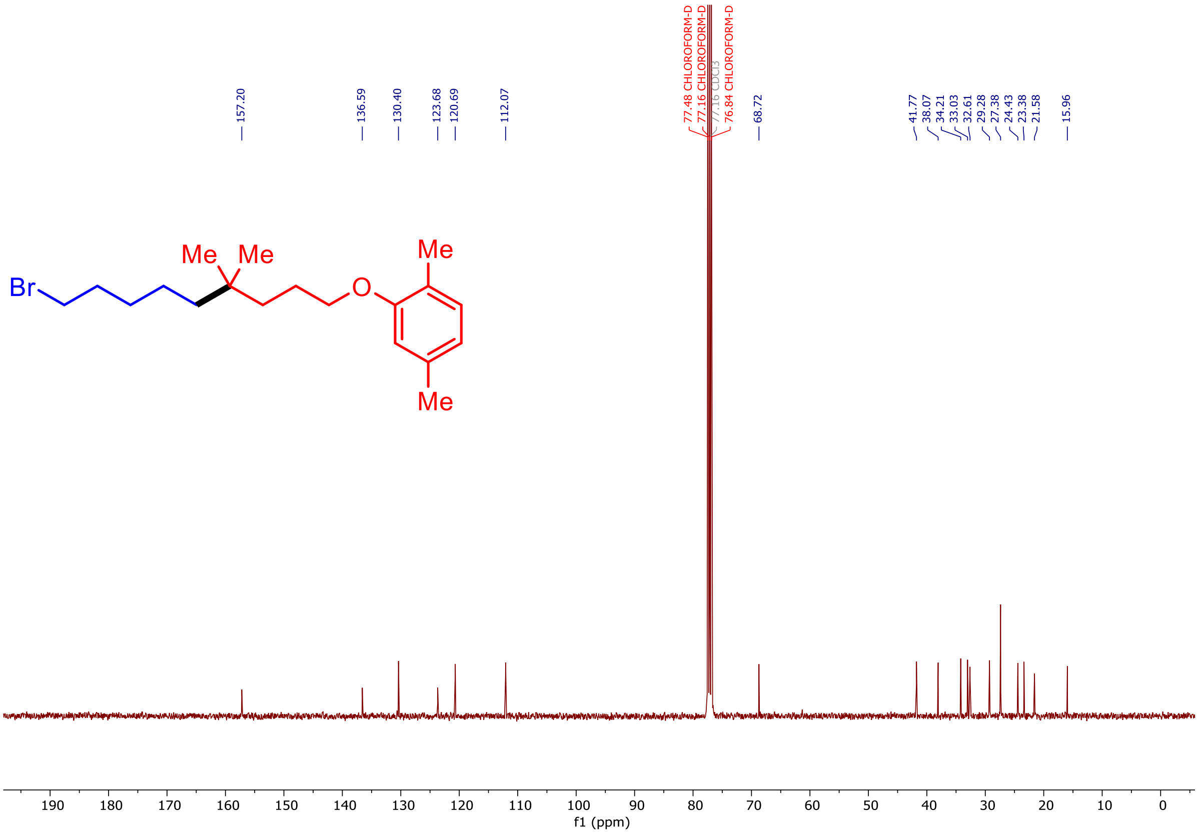

^{1}H NMR (400 MHz, $CDCl_3$) of **12** (***see procedure***)

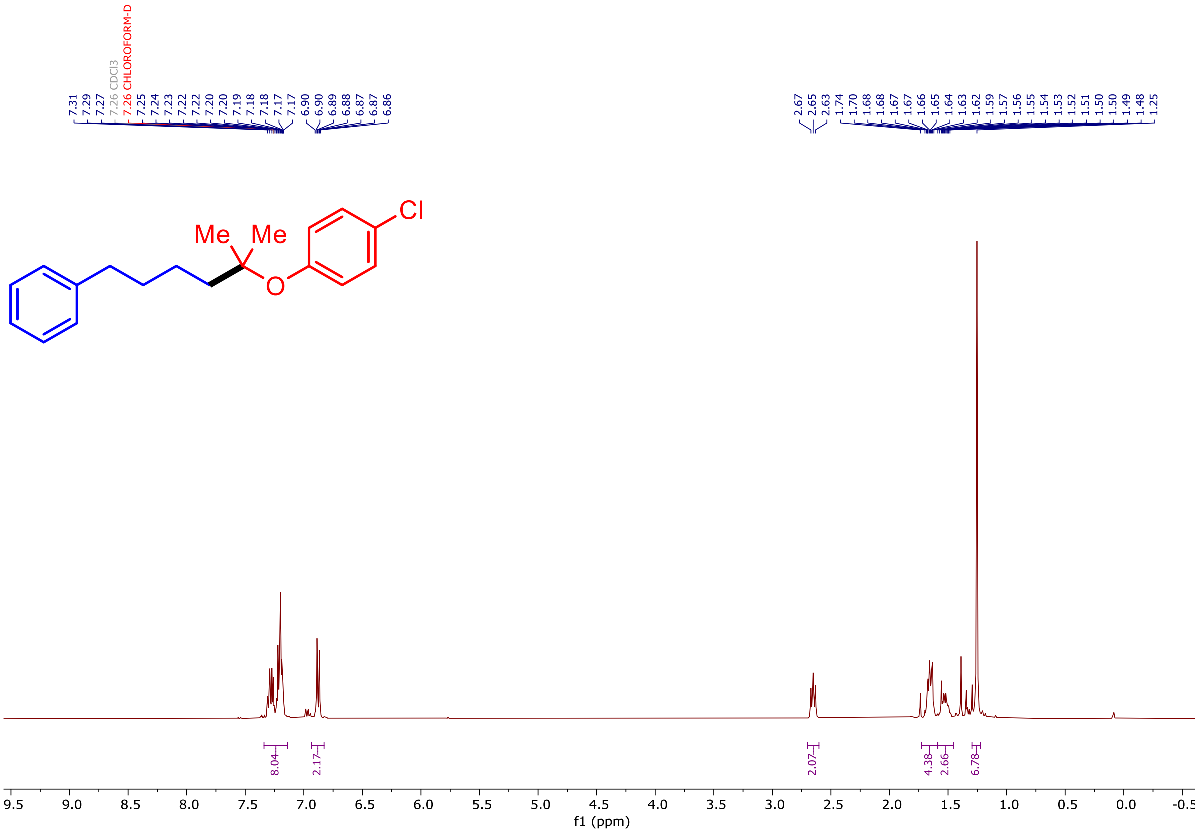

^{13}C NMR (101 MHz, $CDCl_3$) of **12**

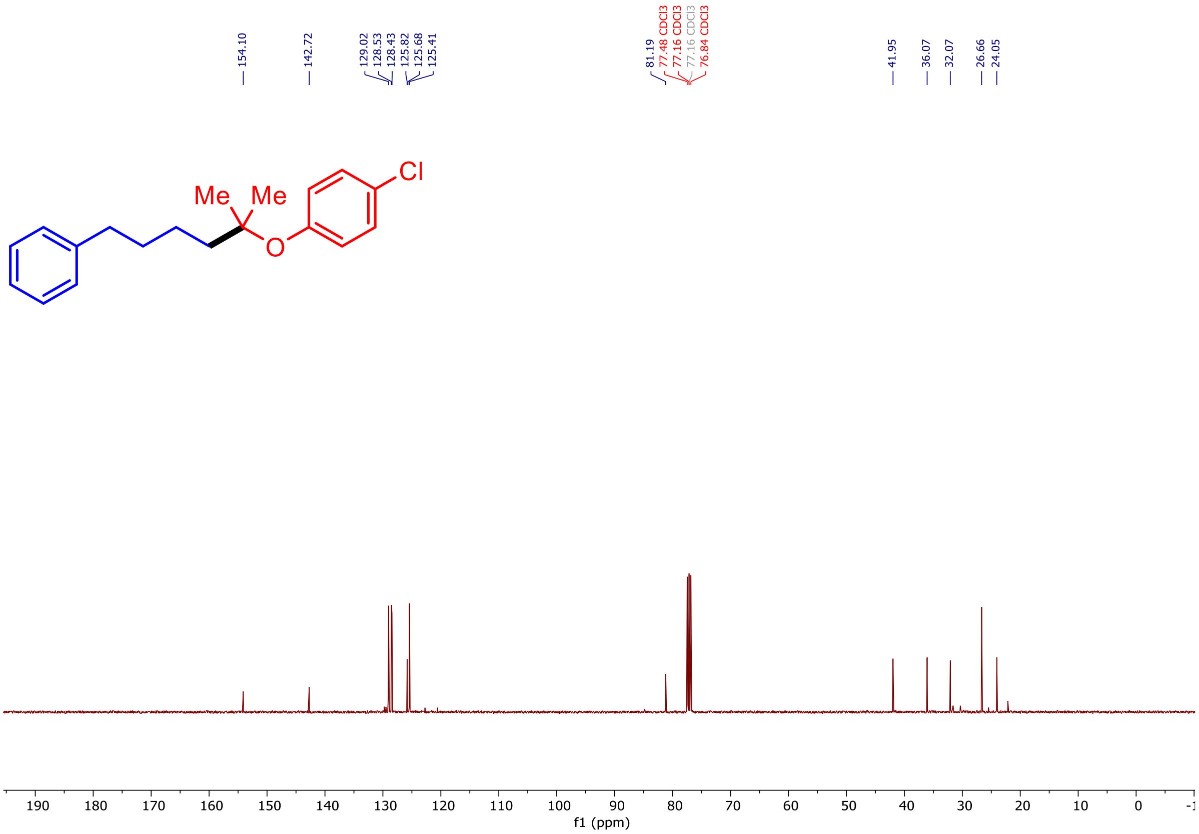

^{1}H NMR (400 MHz, $CDCl_3$) of **13** (***see procedure***)

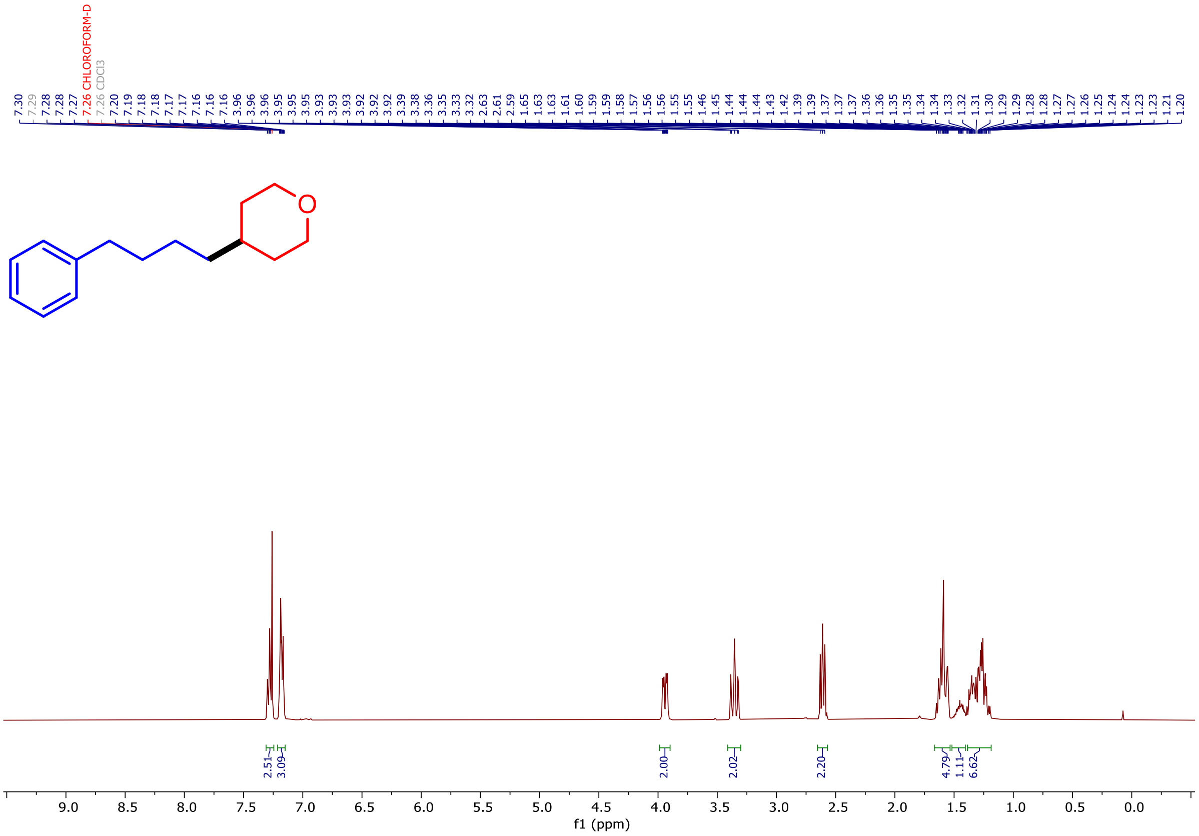

^{13}C NMR (101 MHz, $CDCl_3$) of **13**

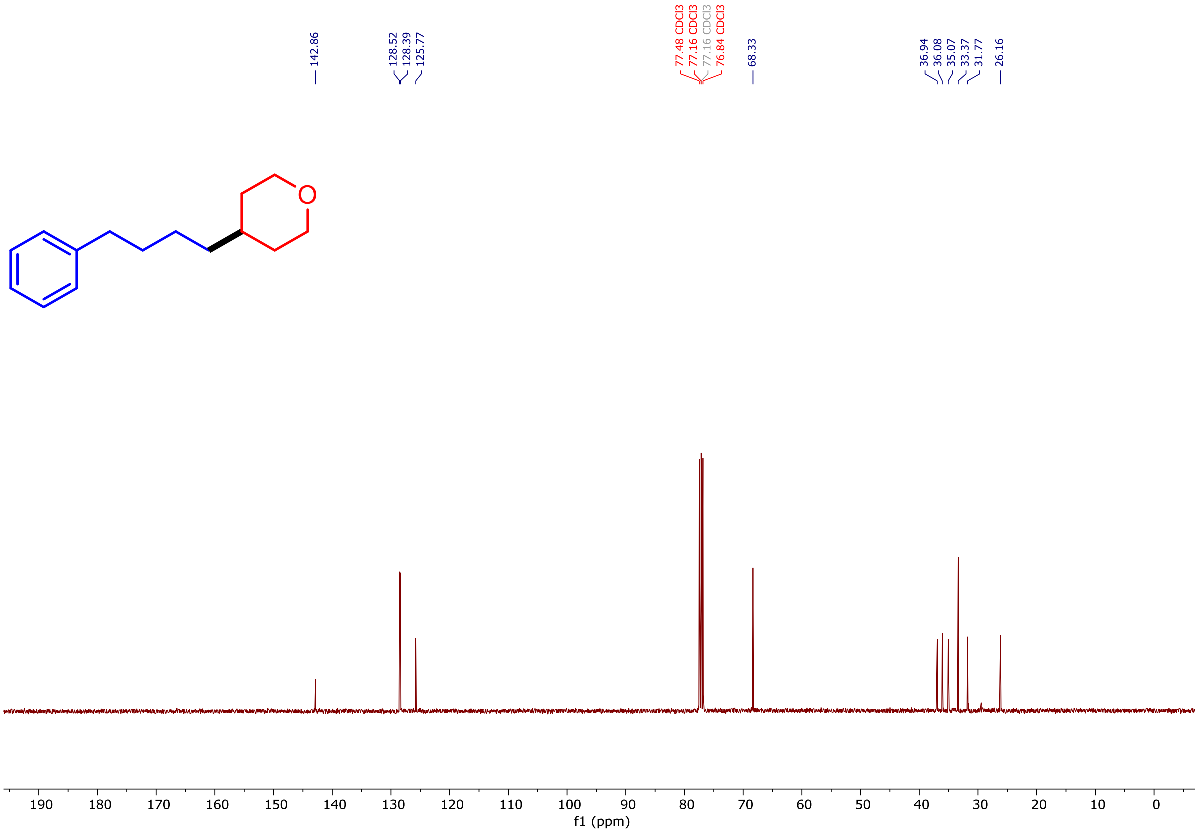

^{1}H NMR (400 MHz, $CDCl_3$) of **14** (*see procedure*)

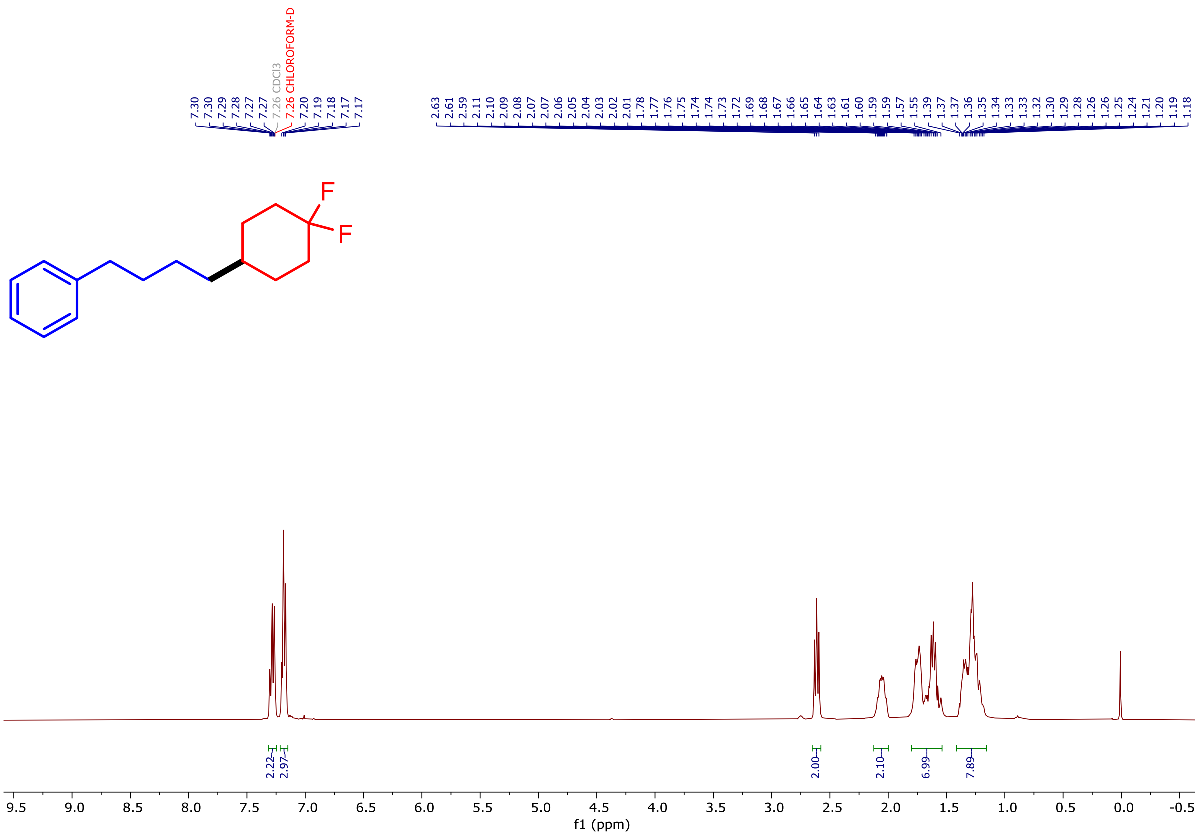

^{13}C NMR (101 MHz, $CDCl_3$) of **14**

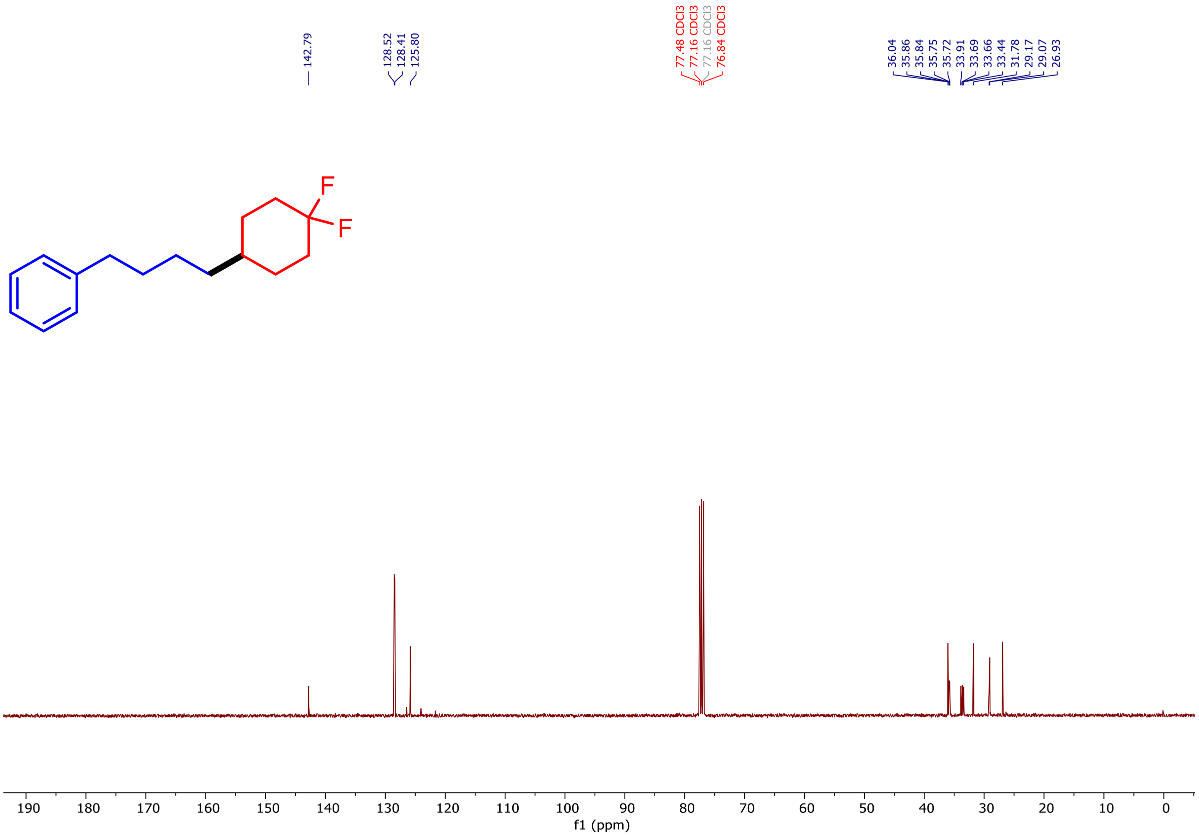

^{19}F NMR (377 MHz, $CDCl_3$) of **14**

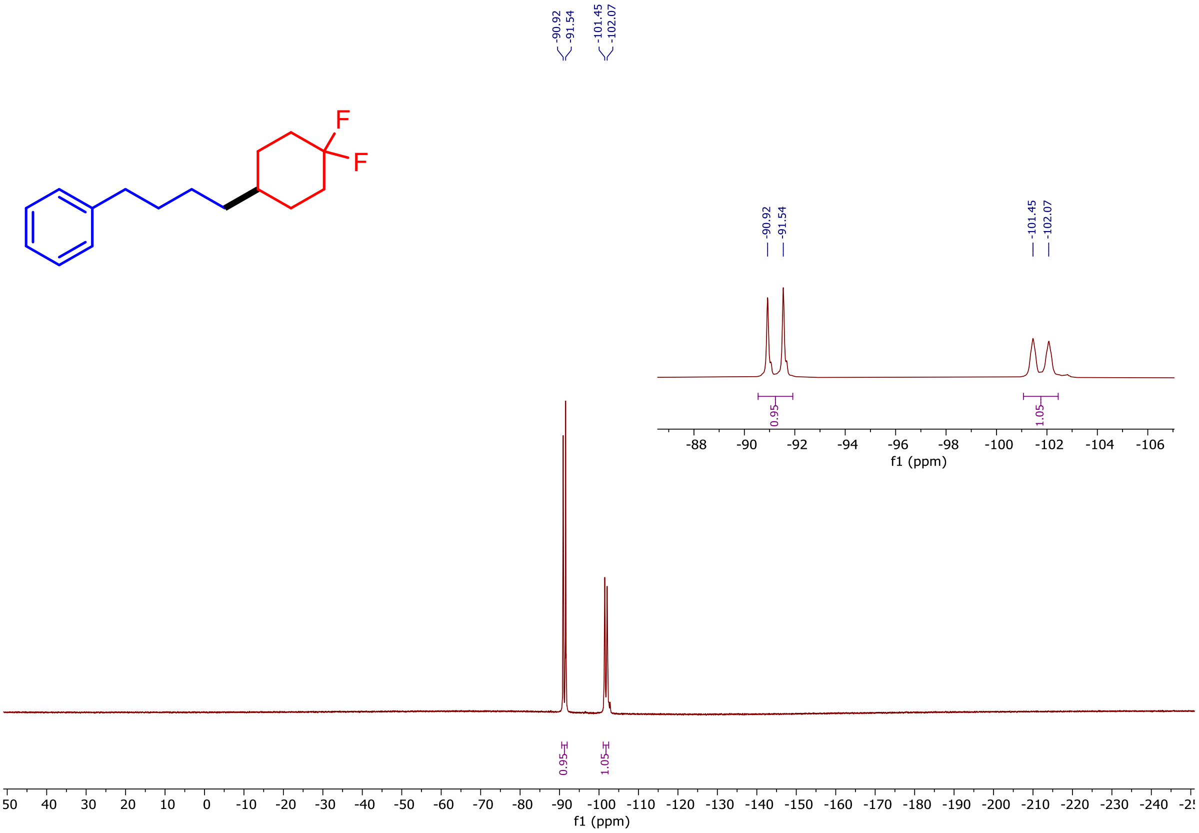

^{1}H NMR (400 MHz, $CDCl_3$) of **15** (*see procedure*)

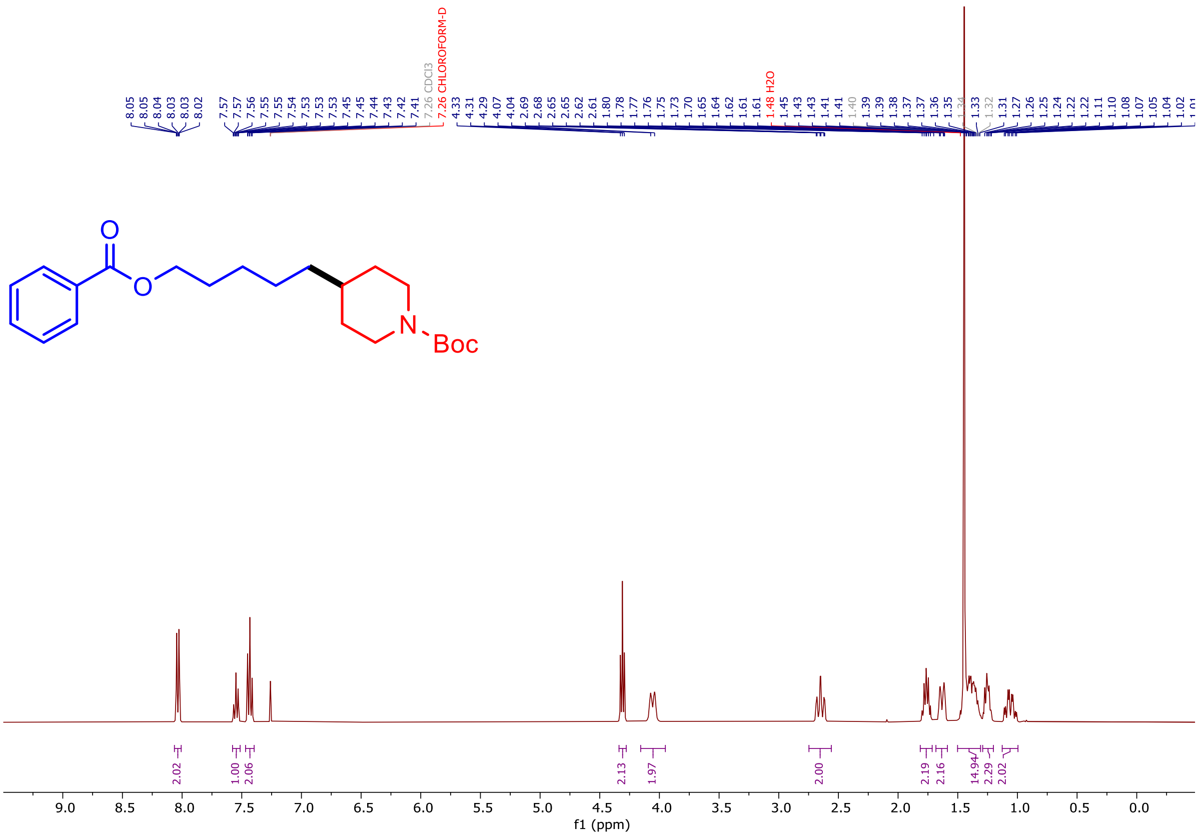

^{13}C NMR (101 MHz, $CDCl_3$) of **15**

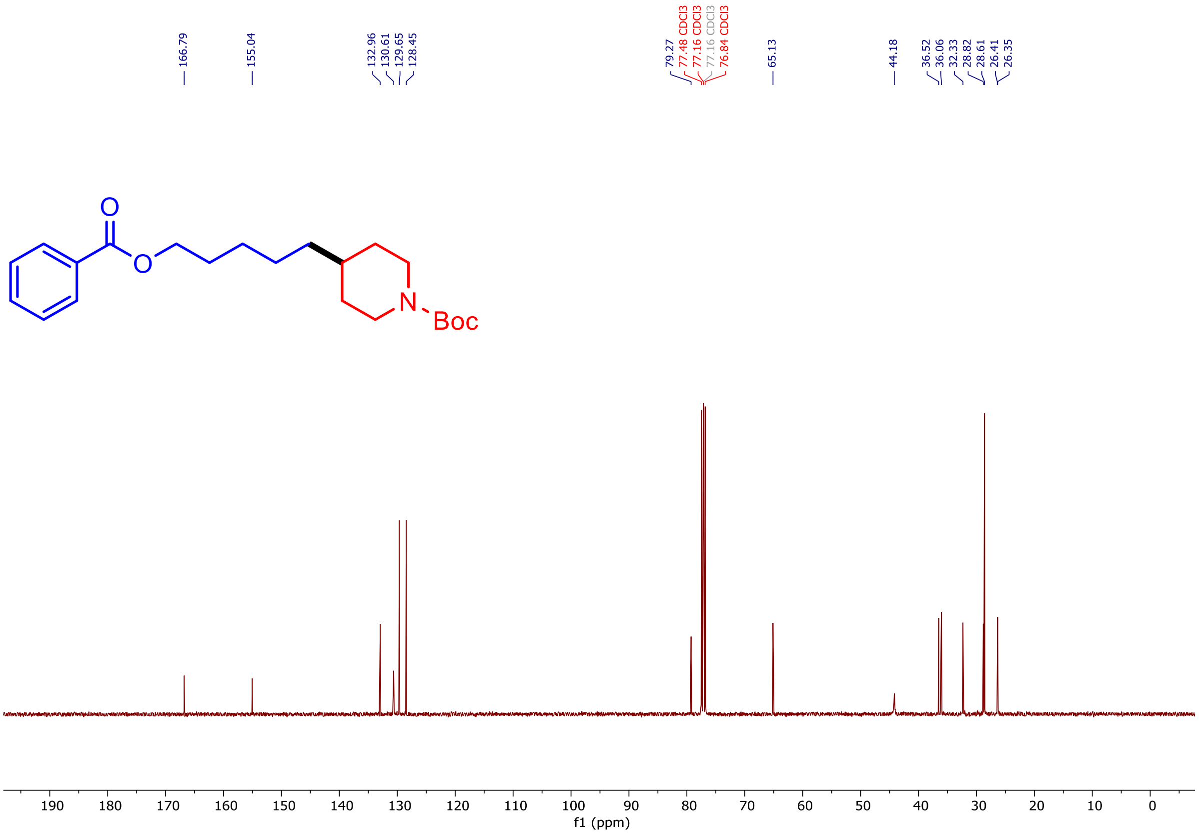

^{1}H NMR (400 MHz, $CDCl_3$) of **16** (***see procedure***)

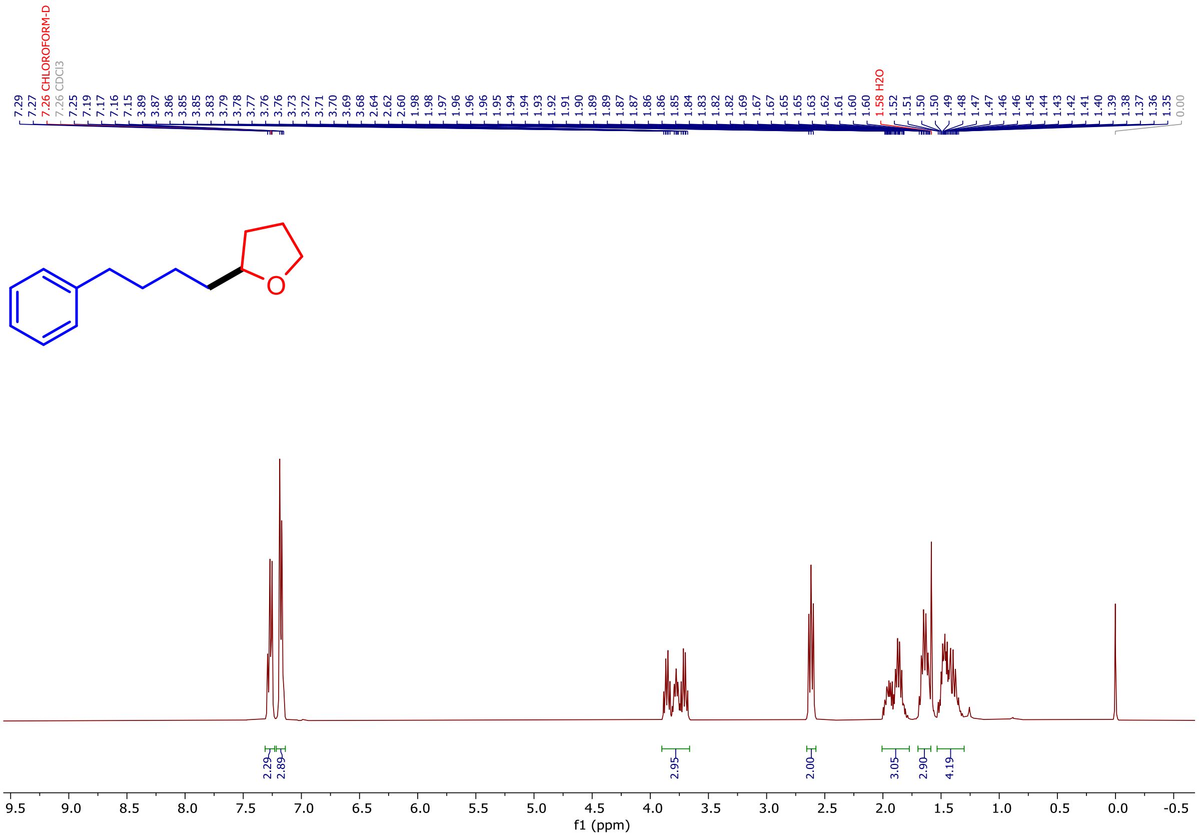

^{13}C NMR (101 MHz, $CDCl_3$) of **16**

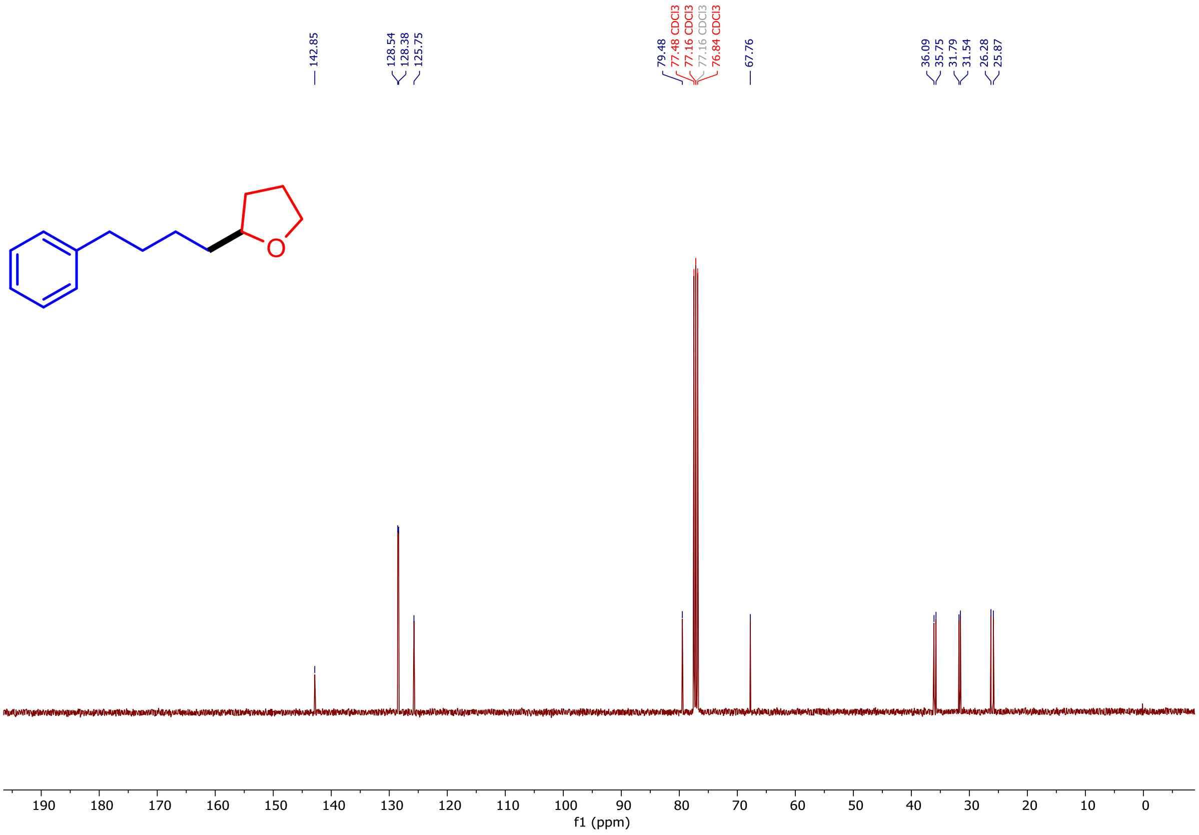

^{1}H NMR (400 MHz, $CDCl_3$) of **17-trans** (***see procedure***)

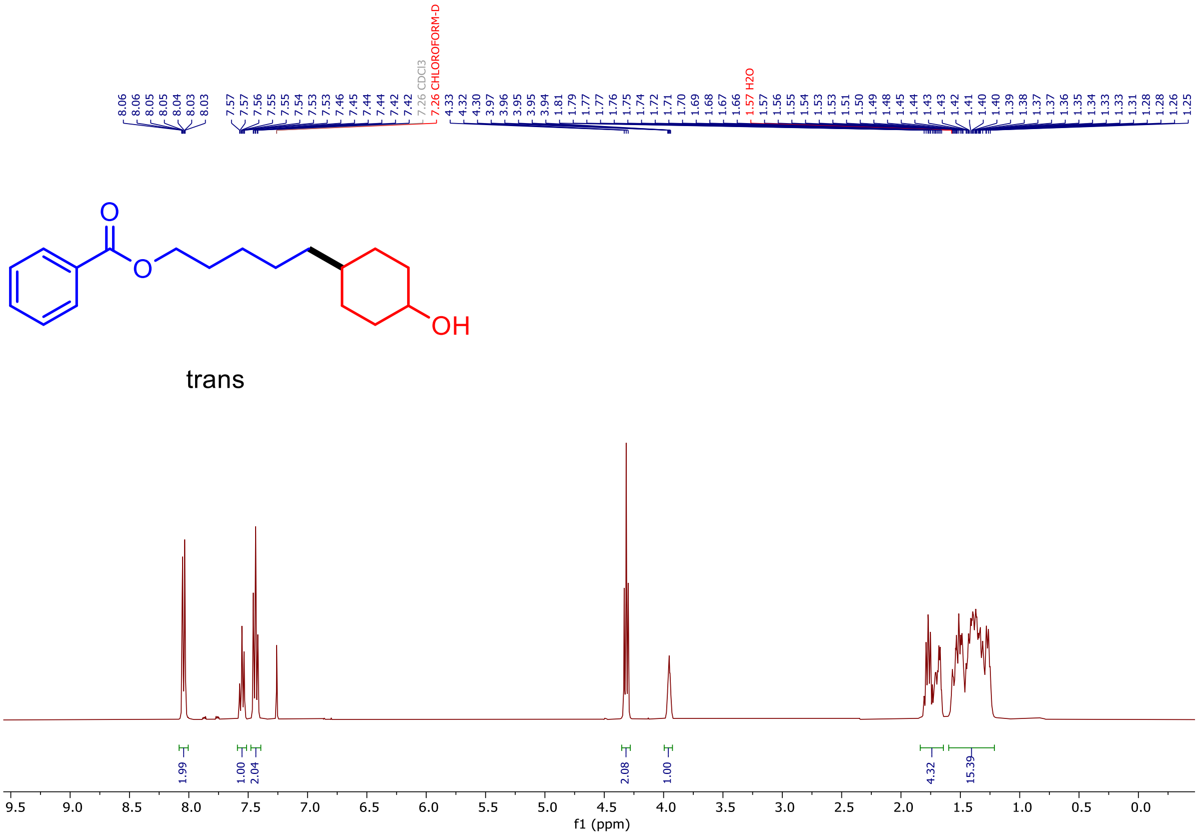

^{13}C NMR (101 MHz, $CDCl_3$) of **17-trans**

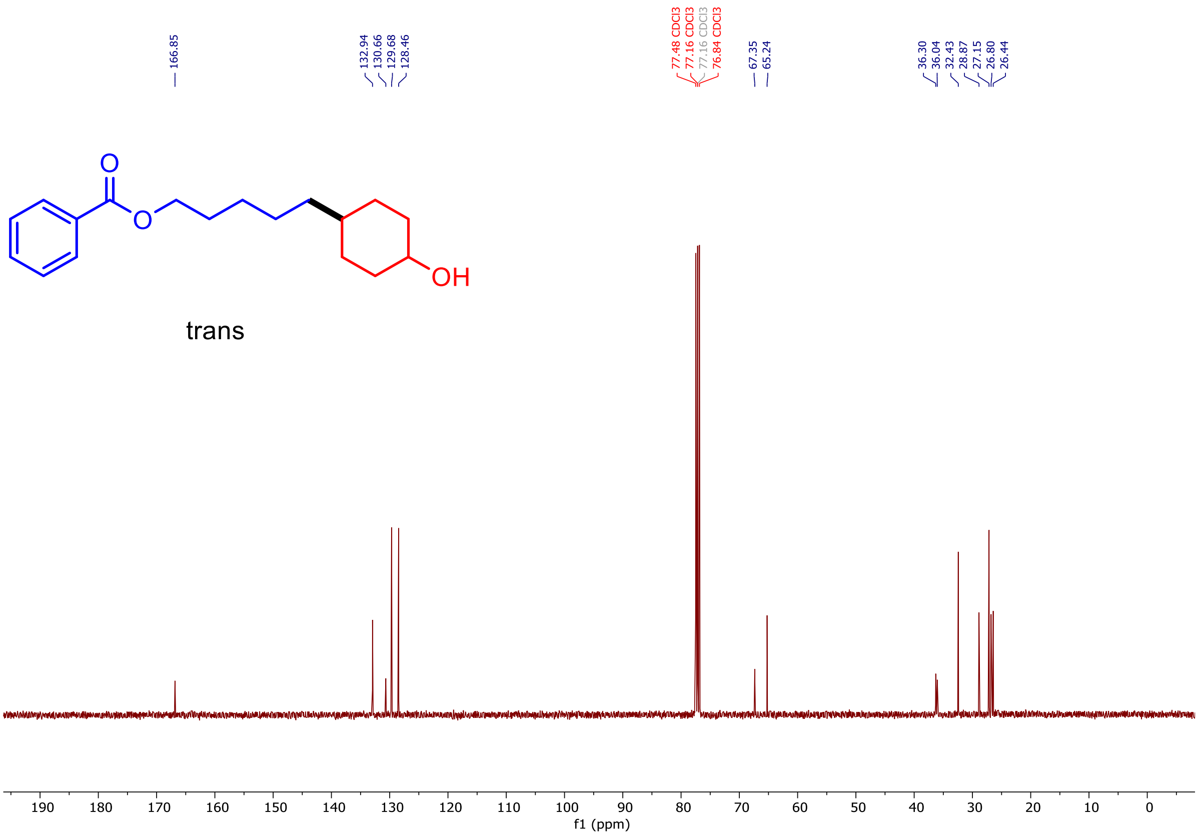

^{1}H NMR (400 MHz, $CDCl_3$) of **17-cis** (***see procedure***)

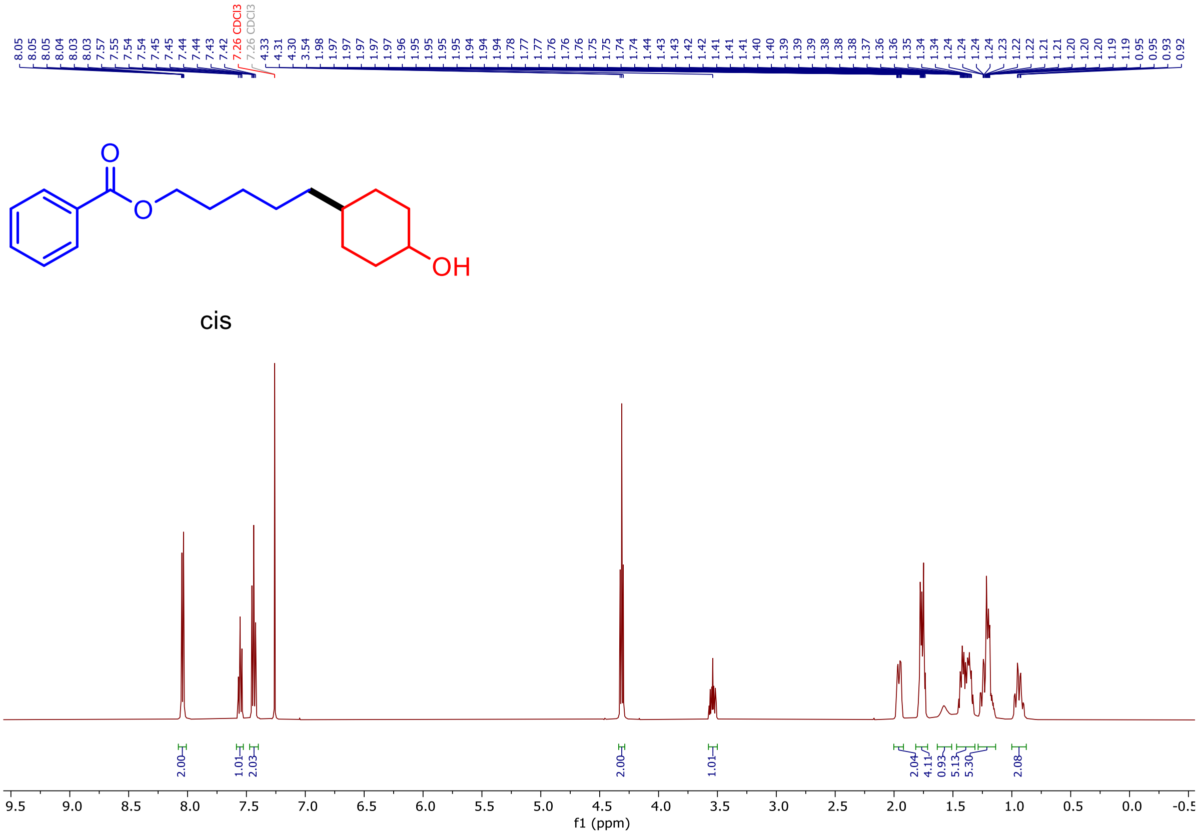

^{13}C NMR (101 MHz, $CDCl_3$) of **17-cis**

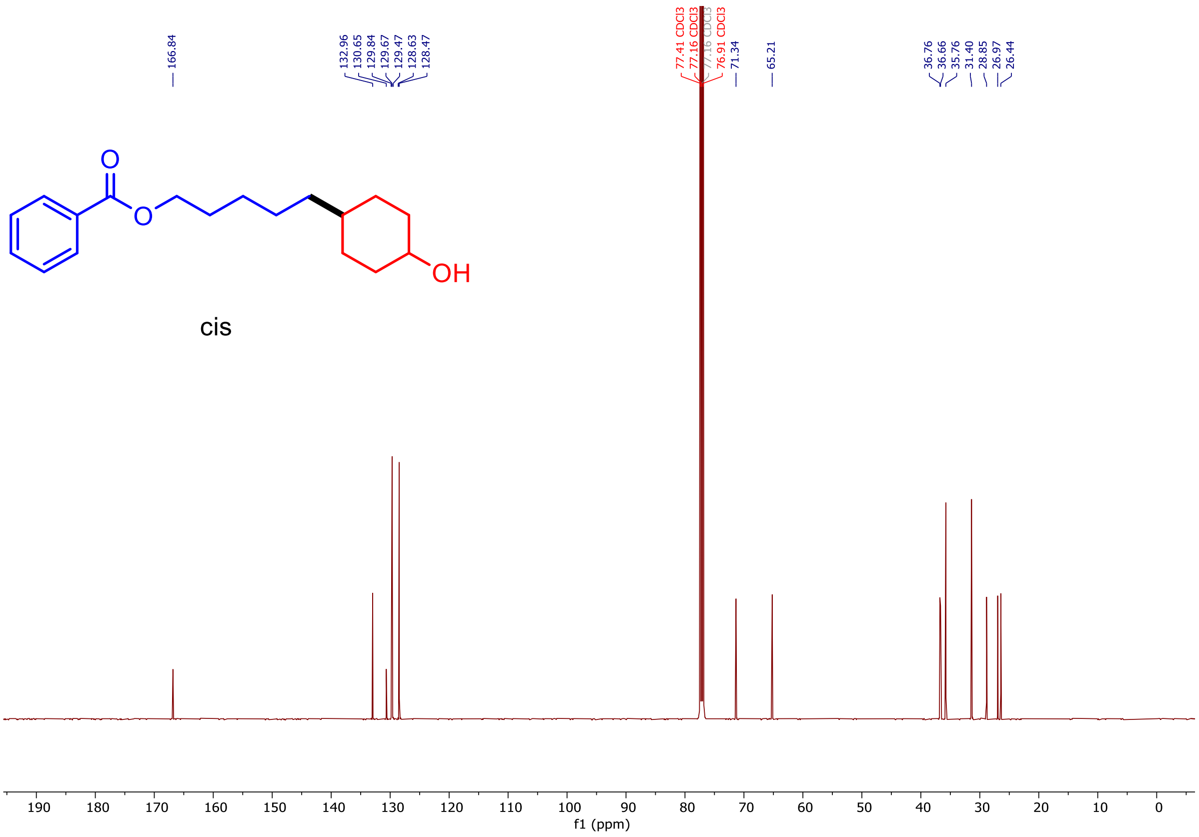

^{1}H NMR (400 MHz, $CDCl_3$) of **18** (***see procedure***)

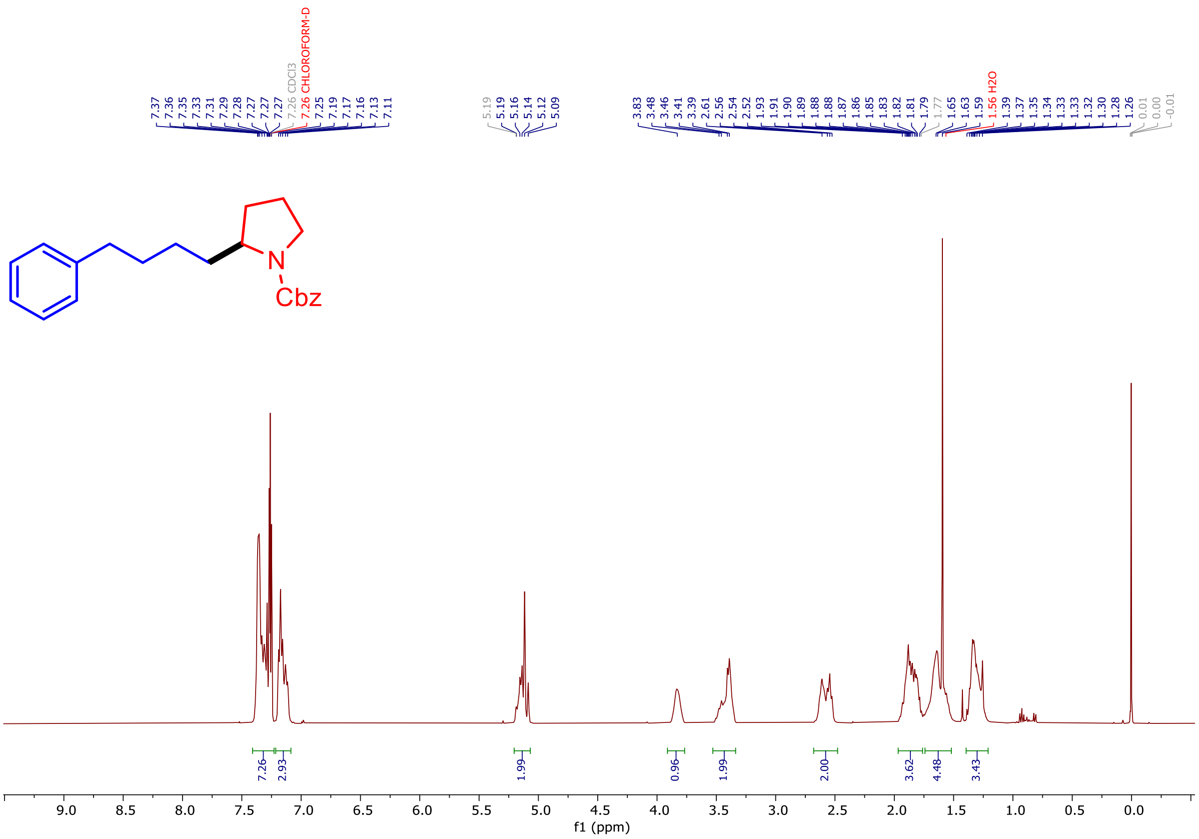

^{13}C NMR (101 MHz, $CDCl_3$) of **18**

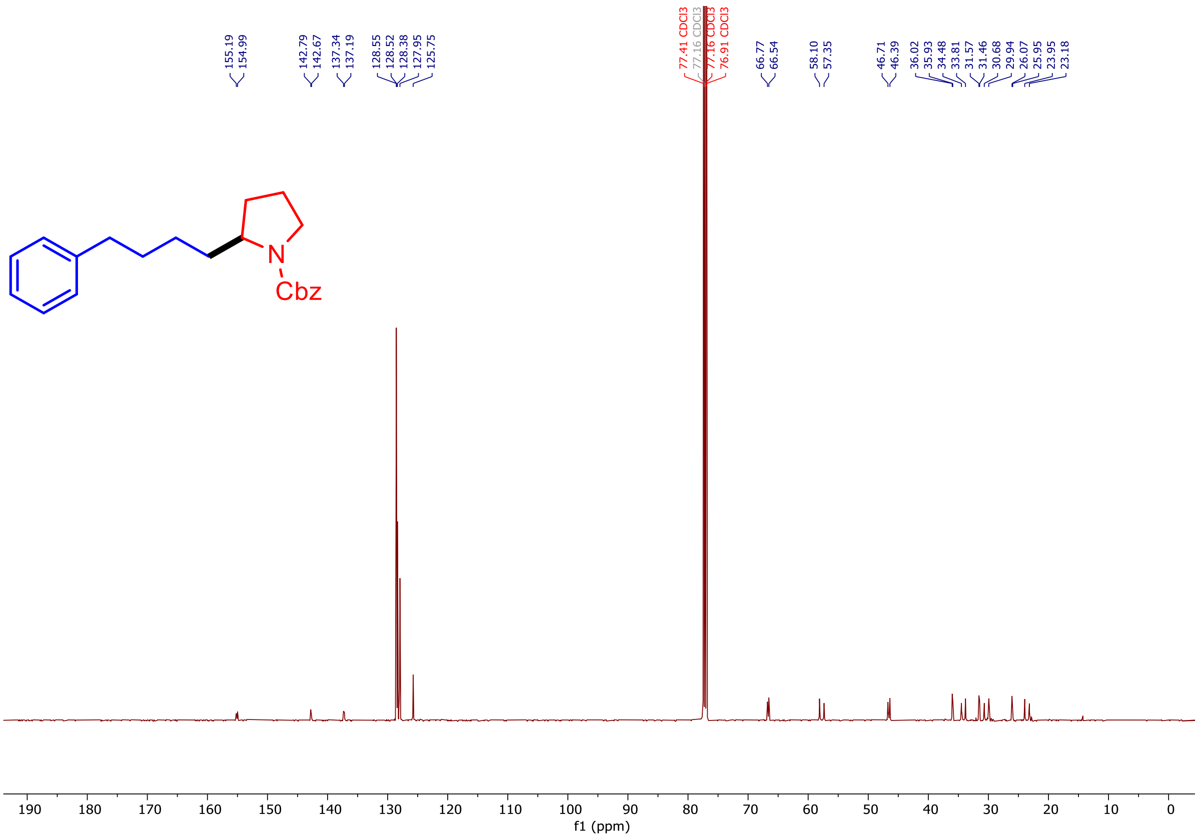

^{1}H NMR (400 MHz, $CDCl_3$) of **19** (***see procedure***)

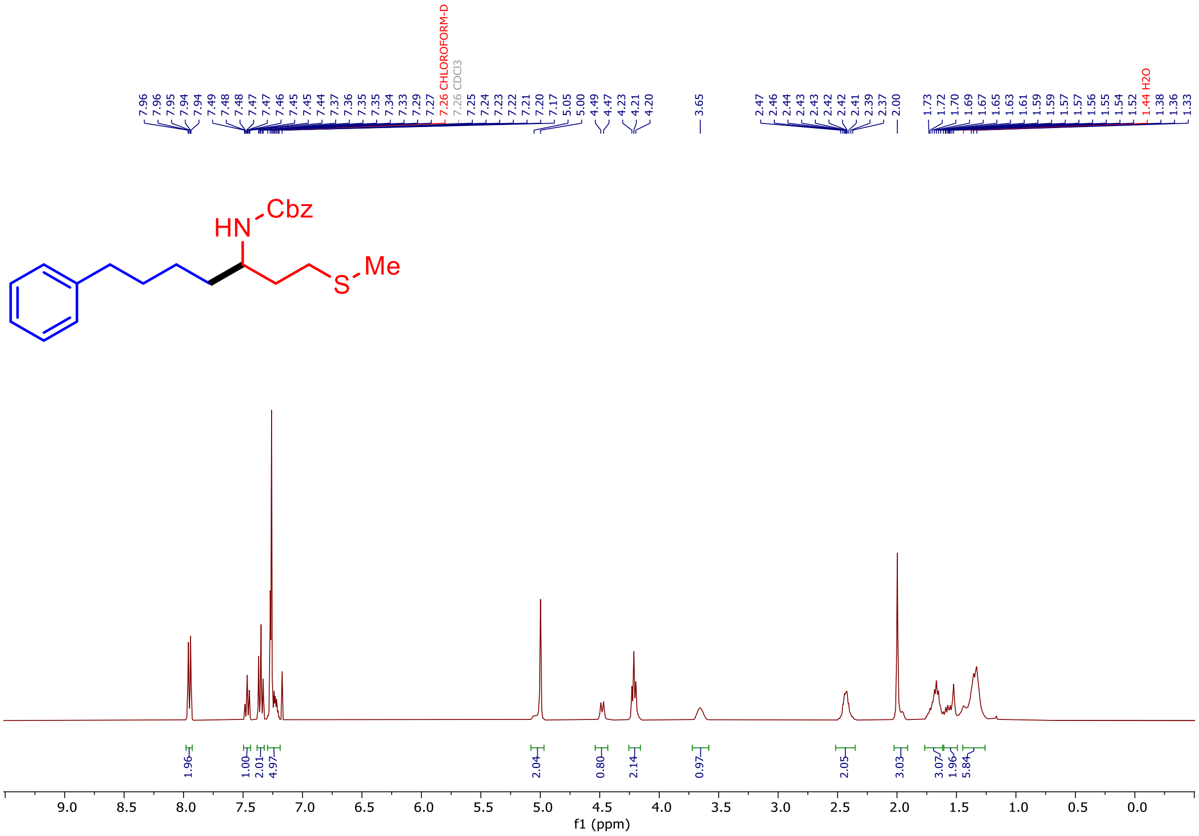

^{13}C NMR (101 MHz, $CDCl_3$) of **19**

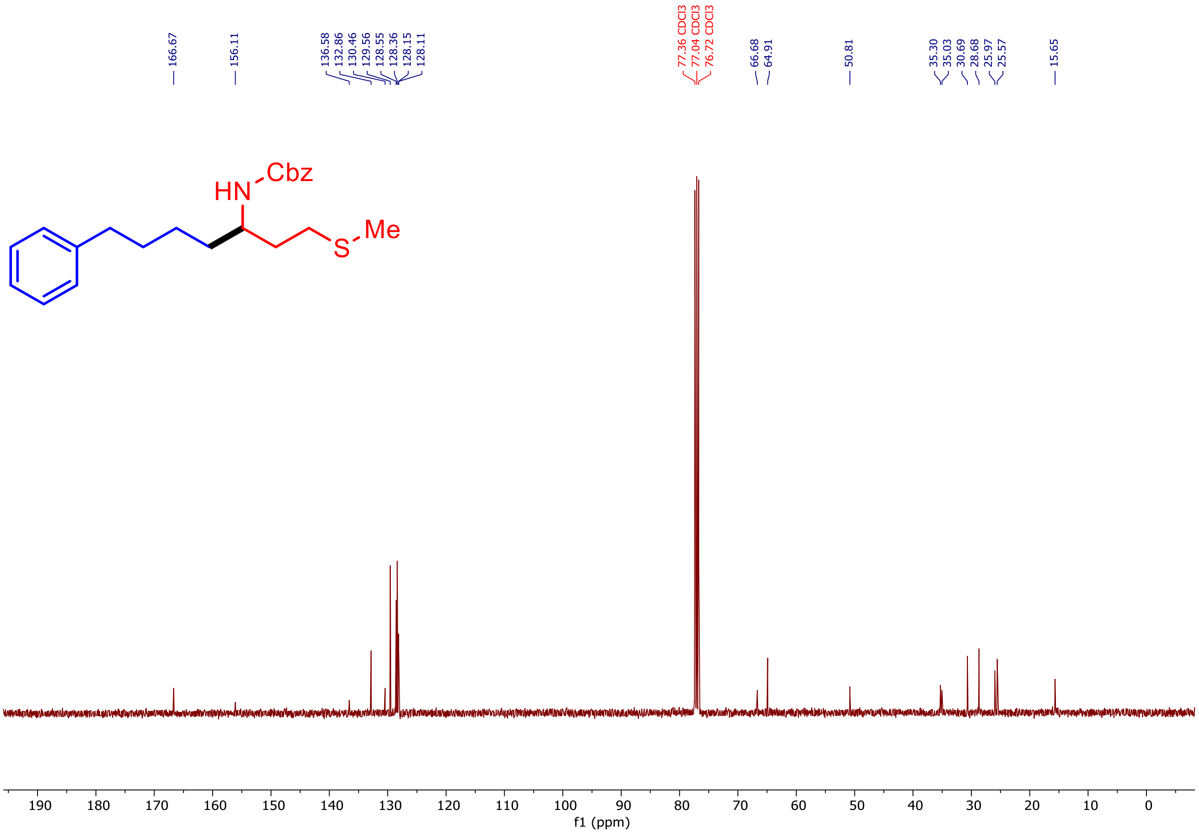

^{1}H NMR (400 MHz, $CDCl_3$) of **R20** (***see procedure***)

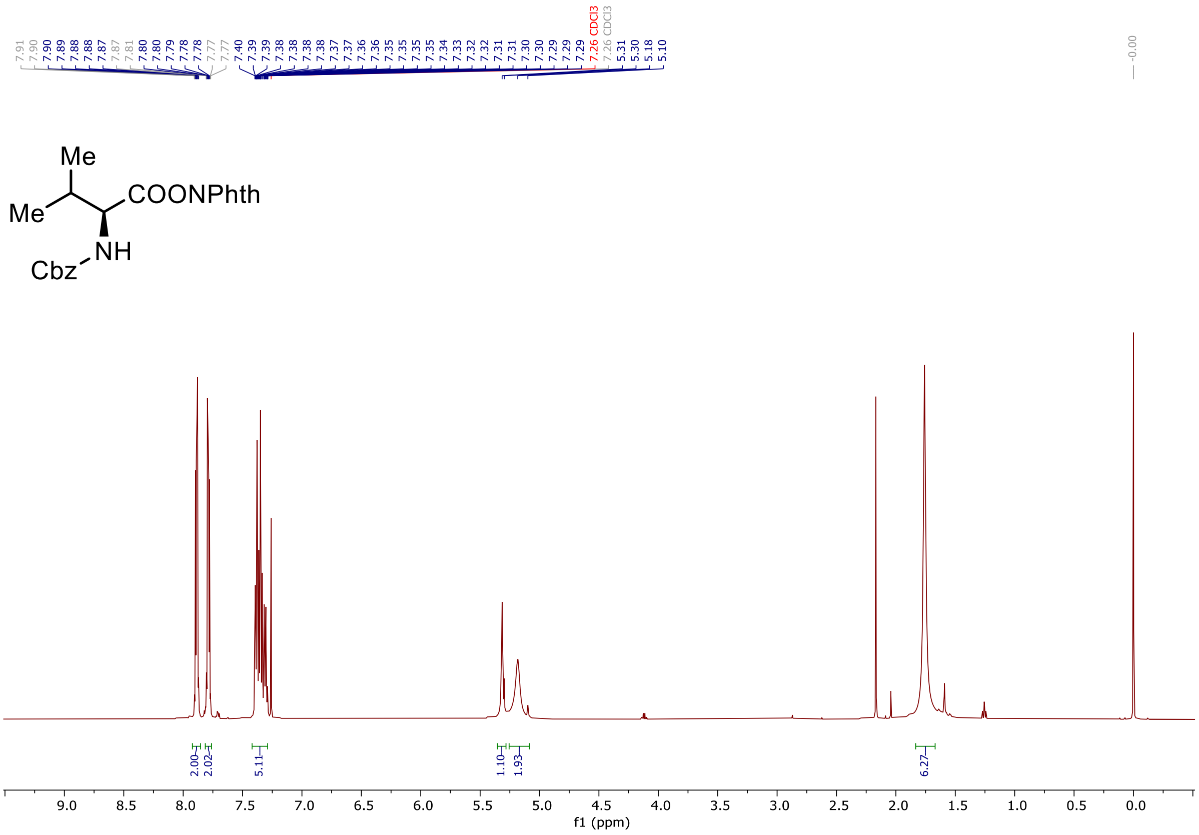

^{13}C NMR (101 MHz, $CDCl_3$) of **R20**

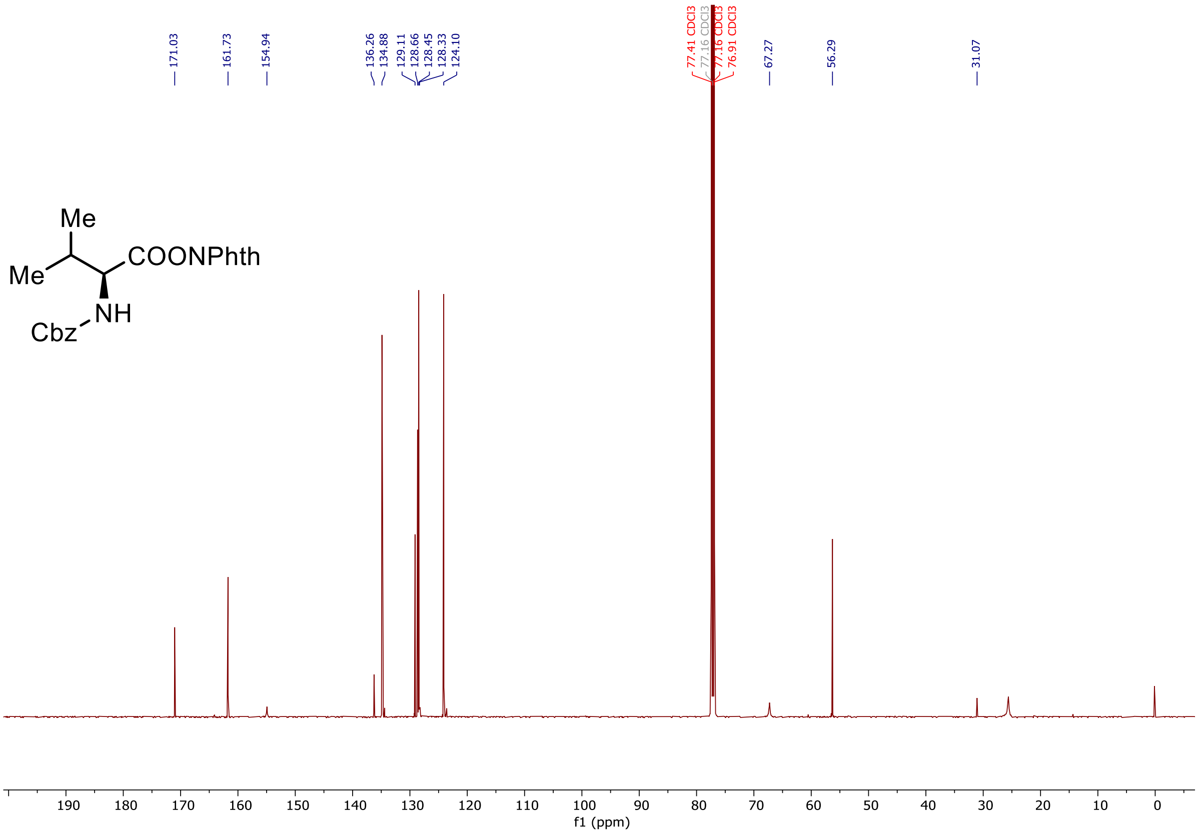

^{1}H NMR (400 MHz, $CDCl_3$) of **20** (*see procedure*)

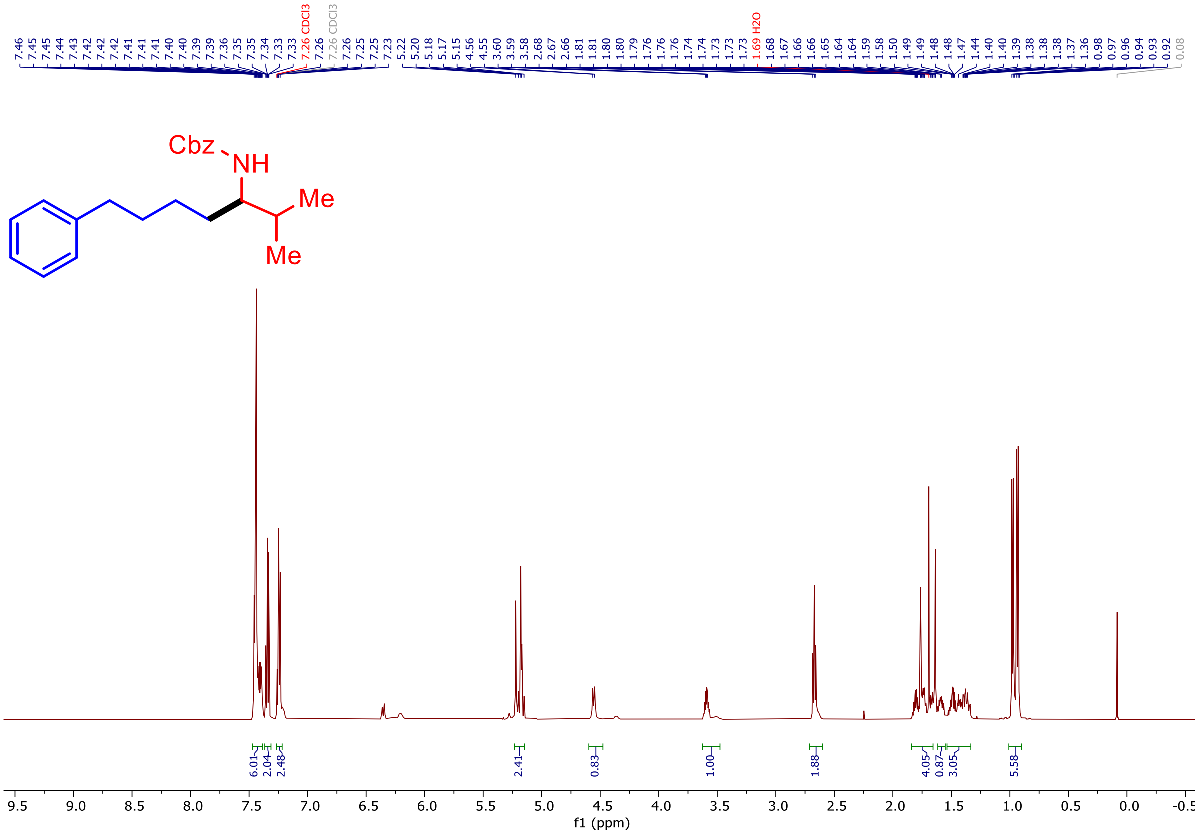

^{13}C NMR (101 MHz, $CDCl_3$) of **20**

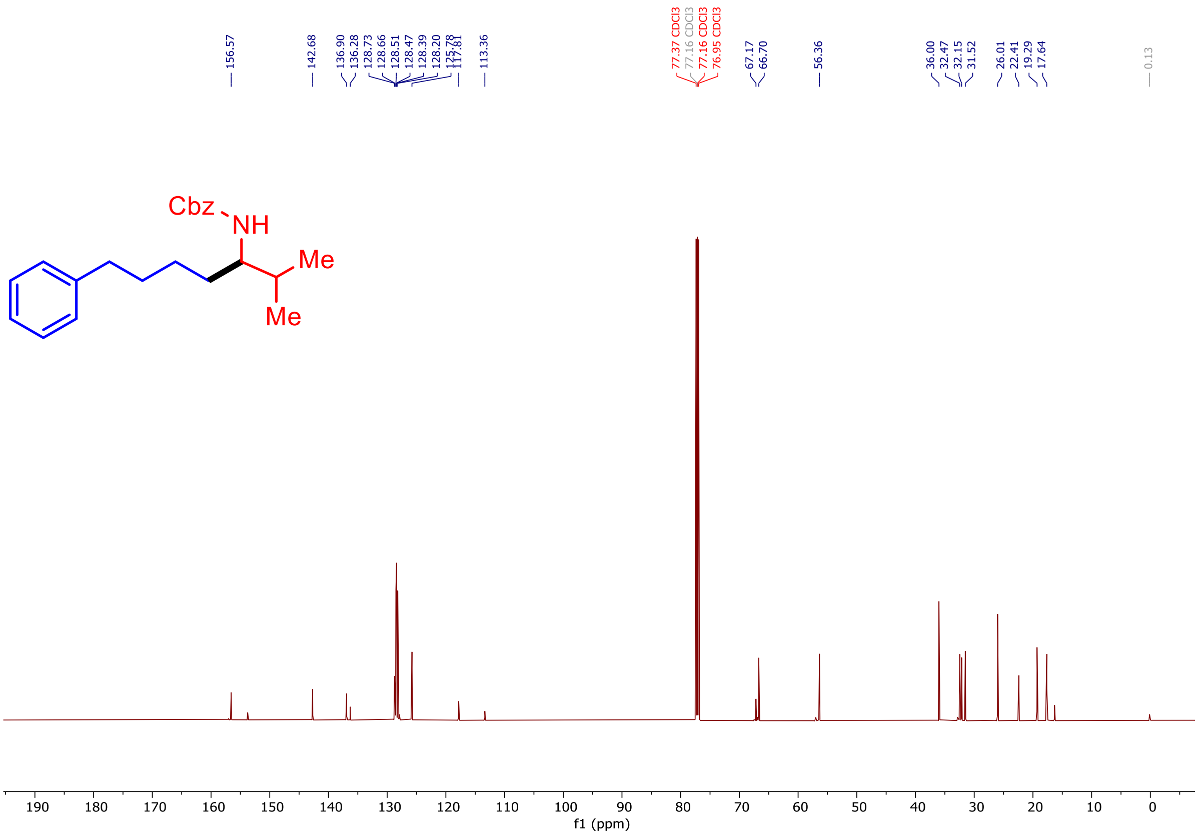

^{1}H NMR (400 MHz, $CDCl_3$) of **21** (***see procedure***)

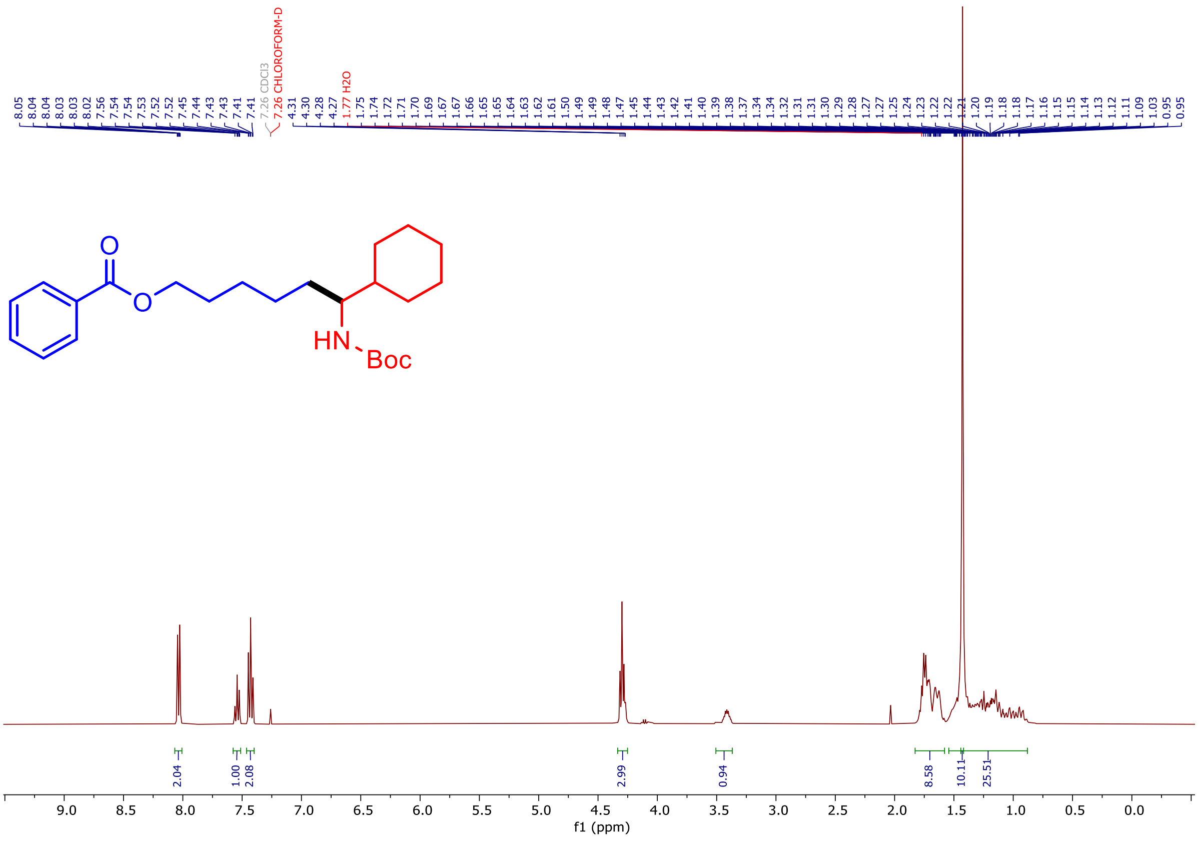

^{13}C NMR (101 MHz, $CDCl_3$) of **21**

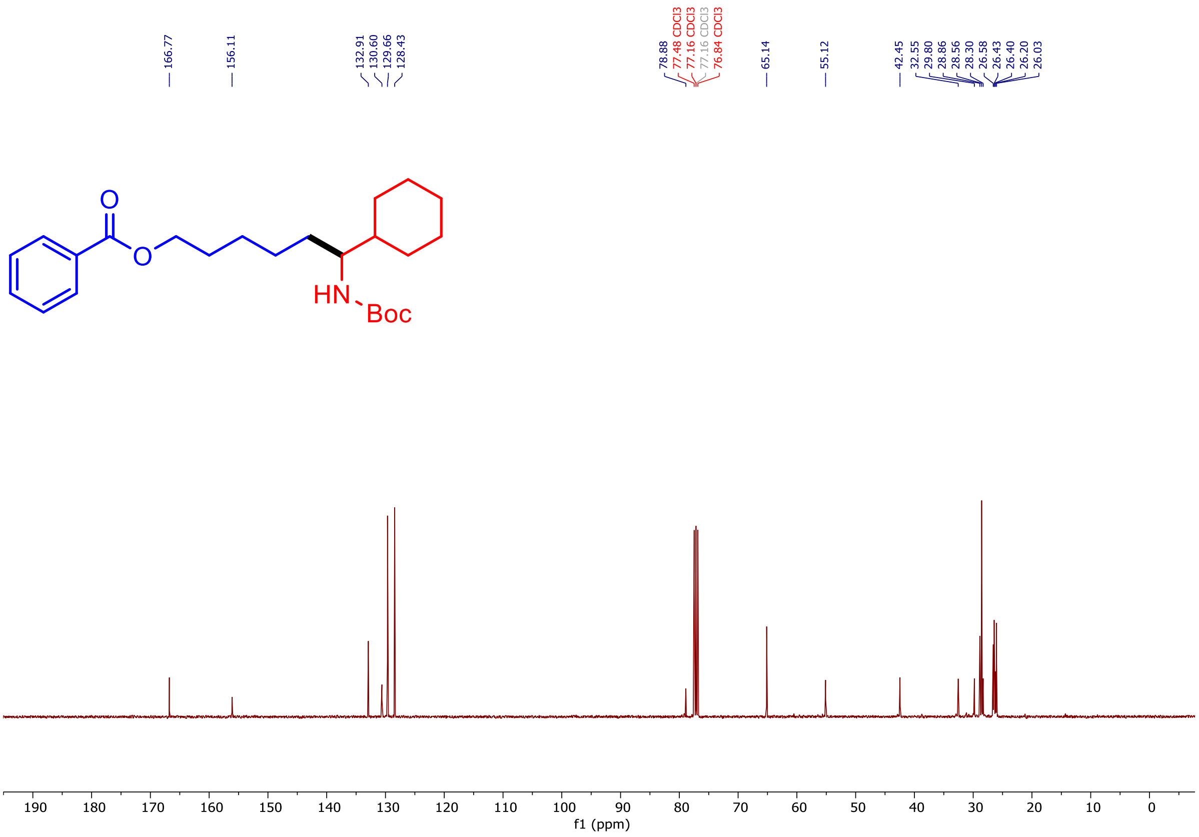

^{1}H NMR (400 MHz, $CDCl_3$) of **22** (*see procedure*)

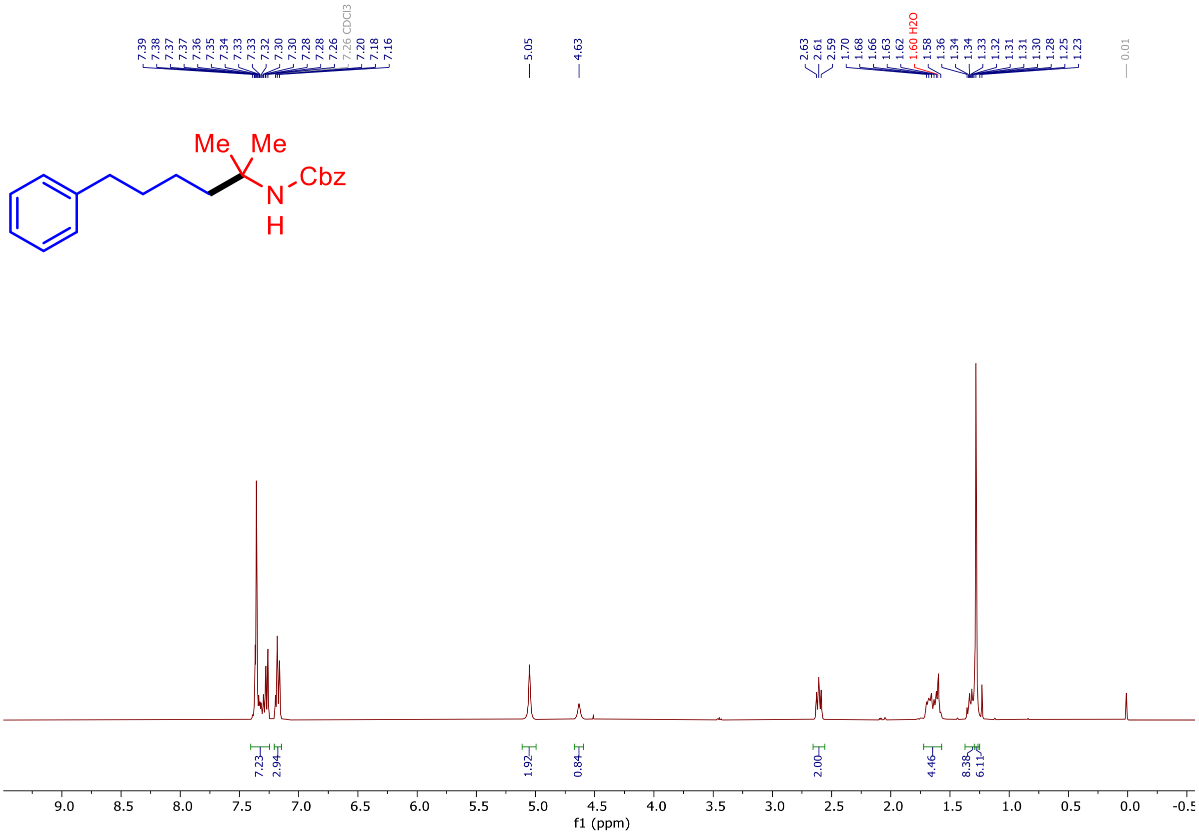

^{13}C NMR (101 MHz, $CDCl_3$) of **22**

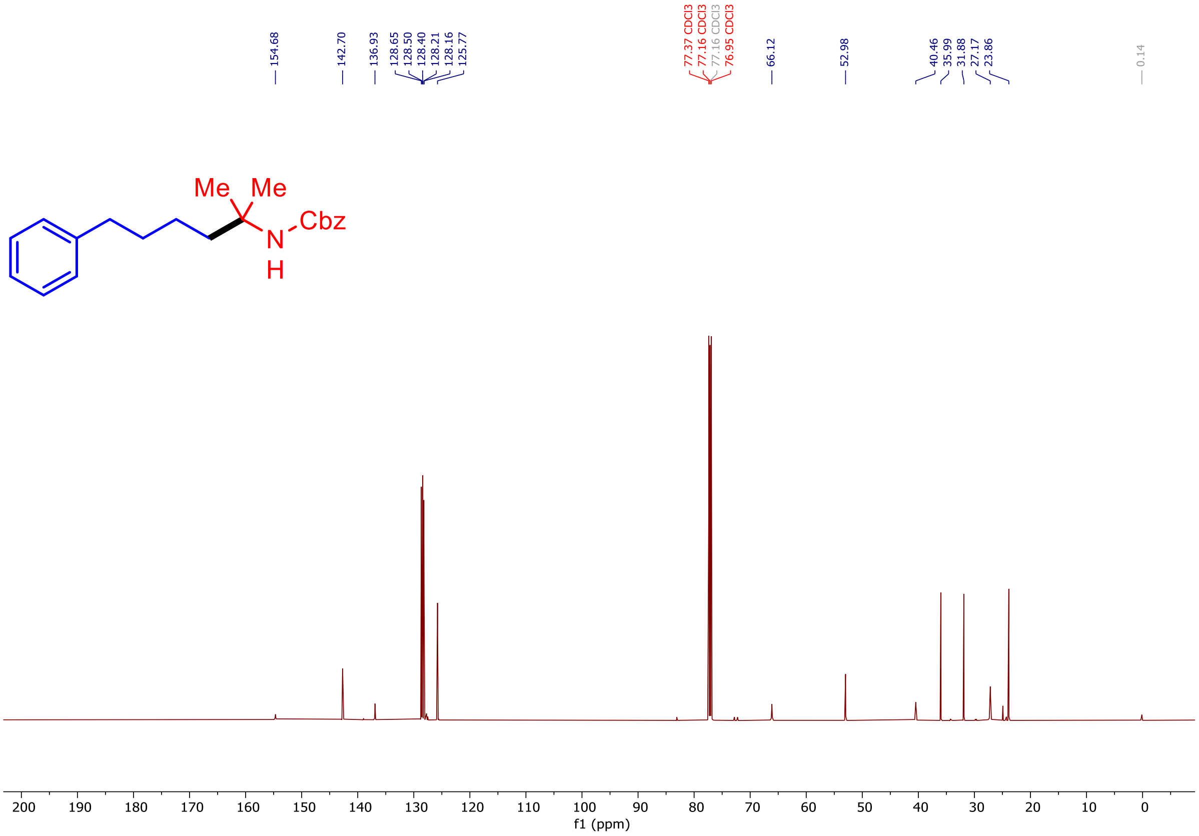

^{1}H NMR (400 MHz, $CDCl_3$) of **23** (*see procedure*)

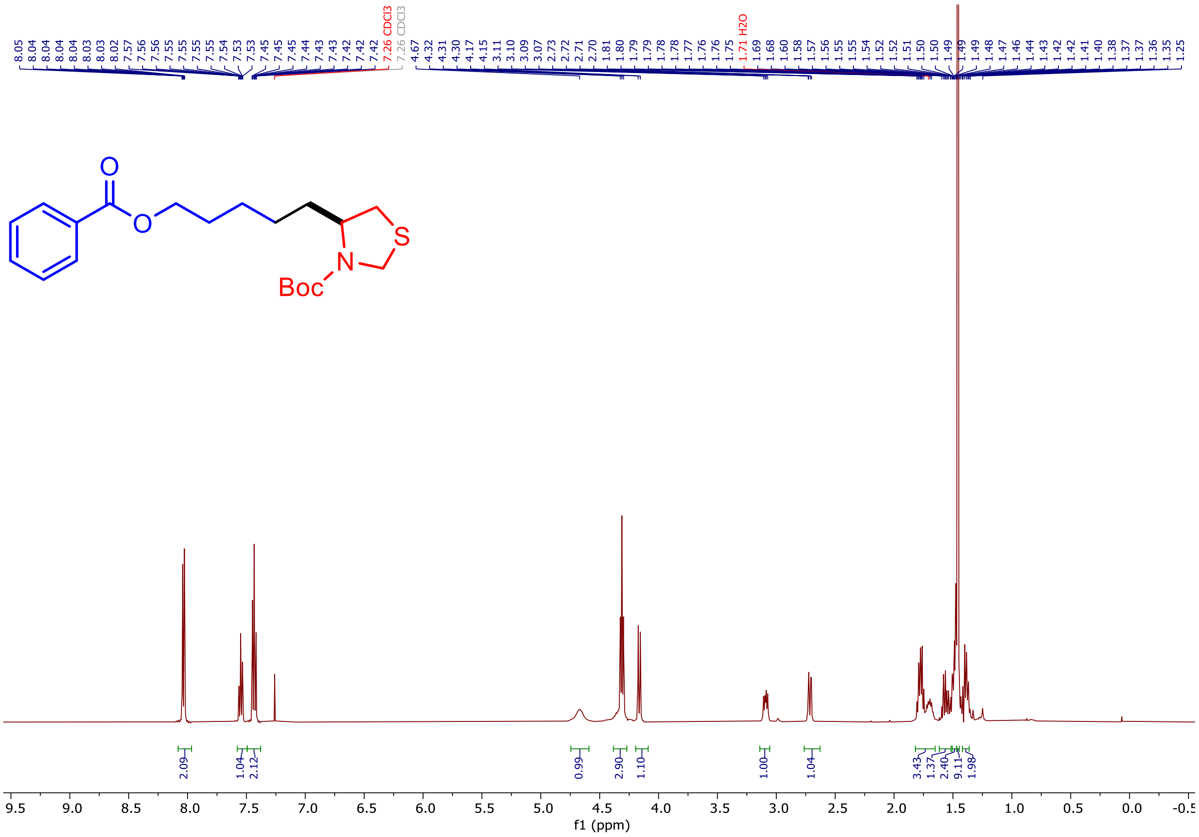

^{13}C NMR (101 MHz, $CDCl_3$) of **23**

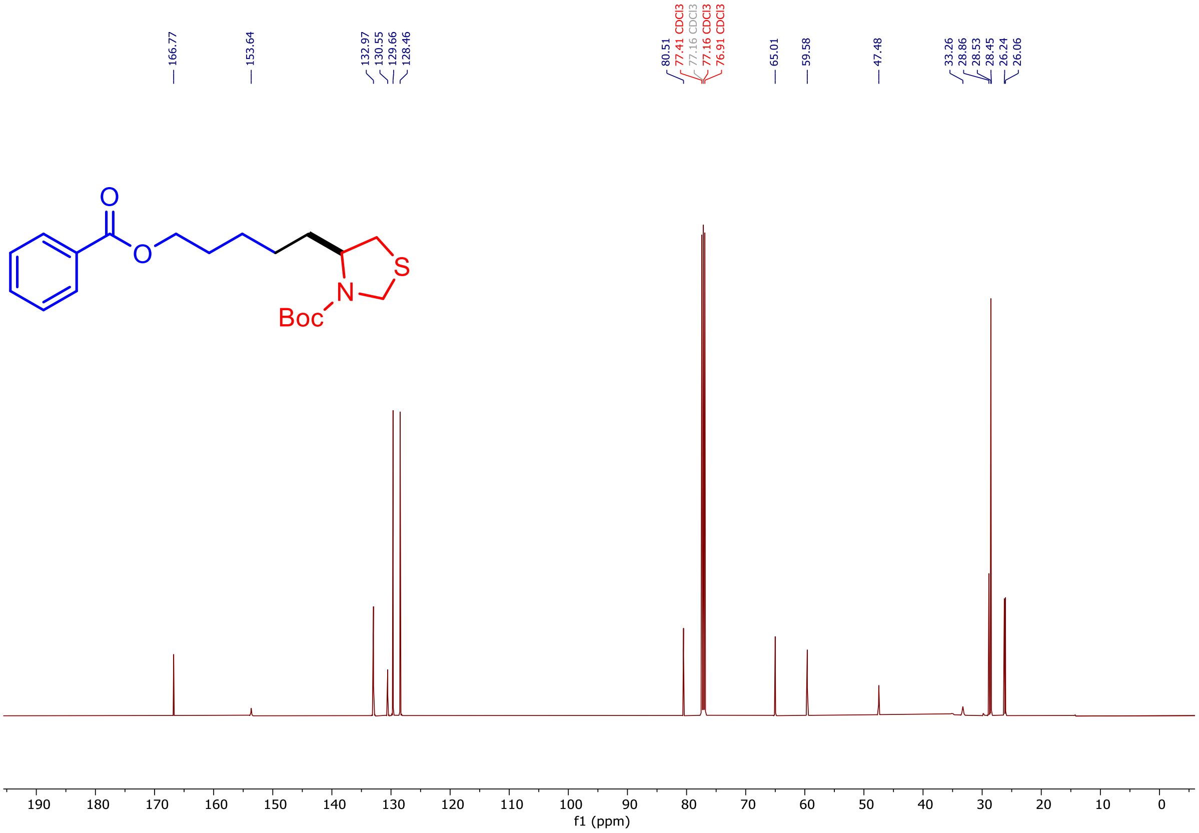

^{1}H NMR (400 MHz, $CDCl_3$) of **24** (*see procedure*)

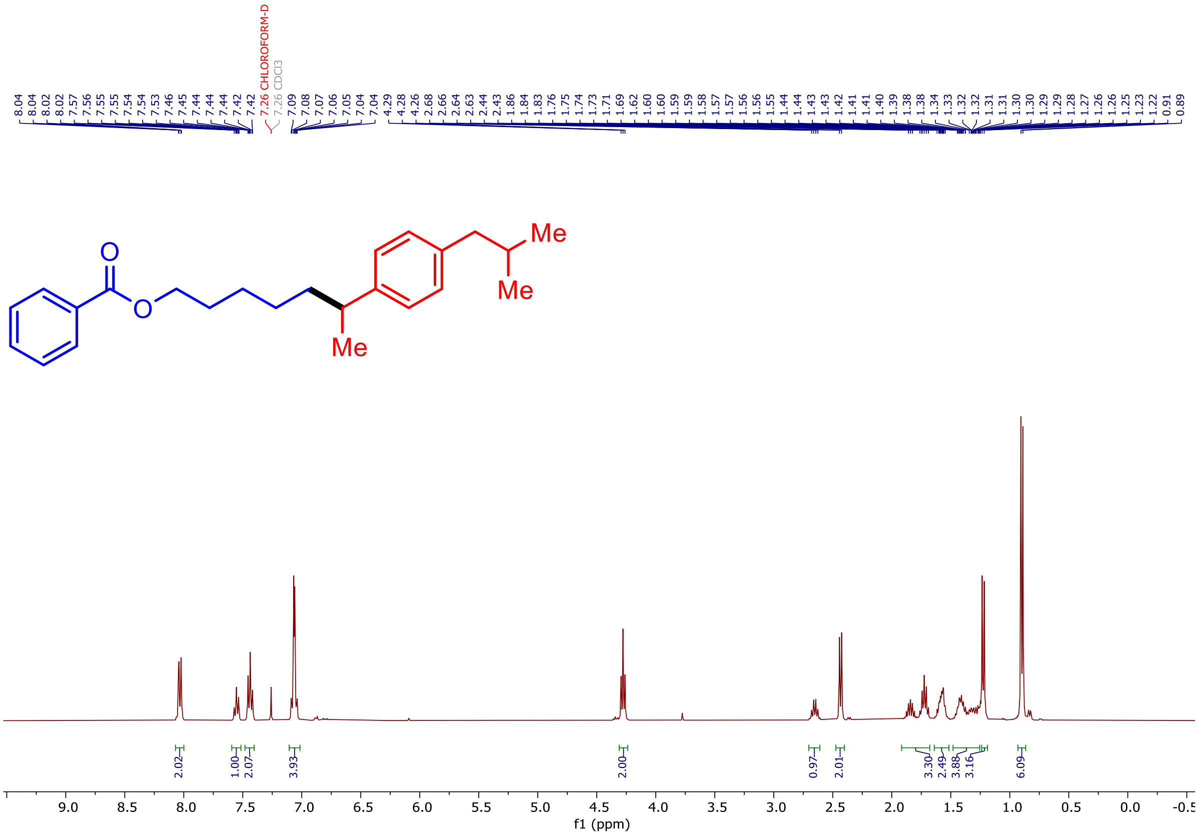

^{13}C NMR (101 MHz, $CDCl_3$) of **24**

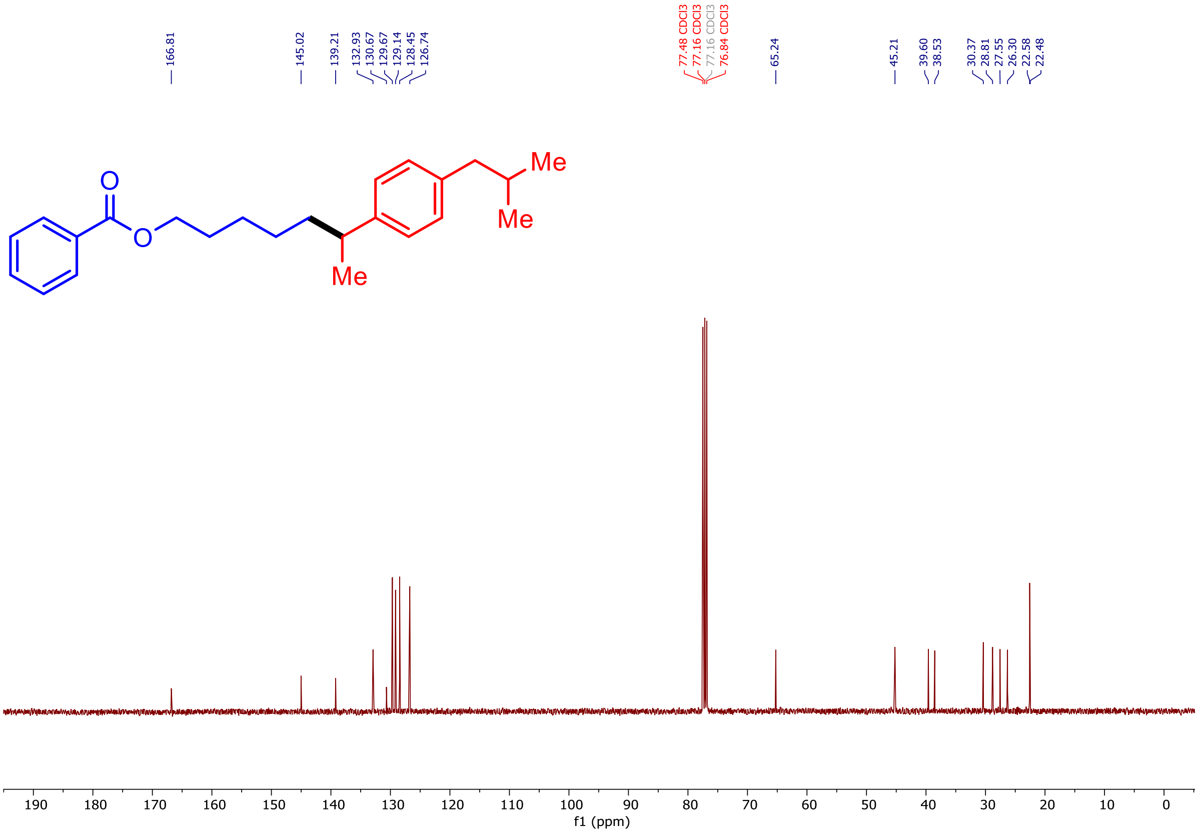

^{1}H NMR (400 MHz, $CDCl_3$) of **25** (*see procedure*)

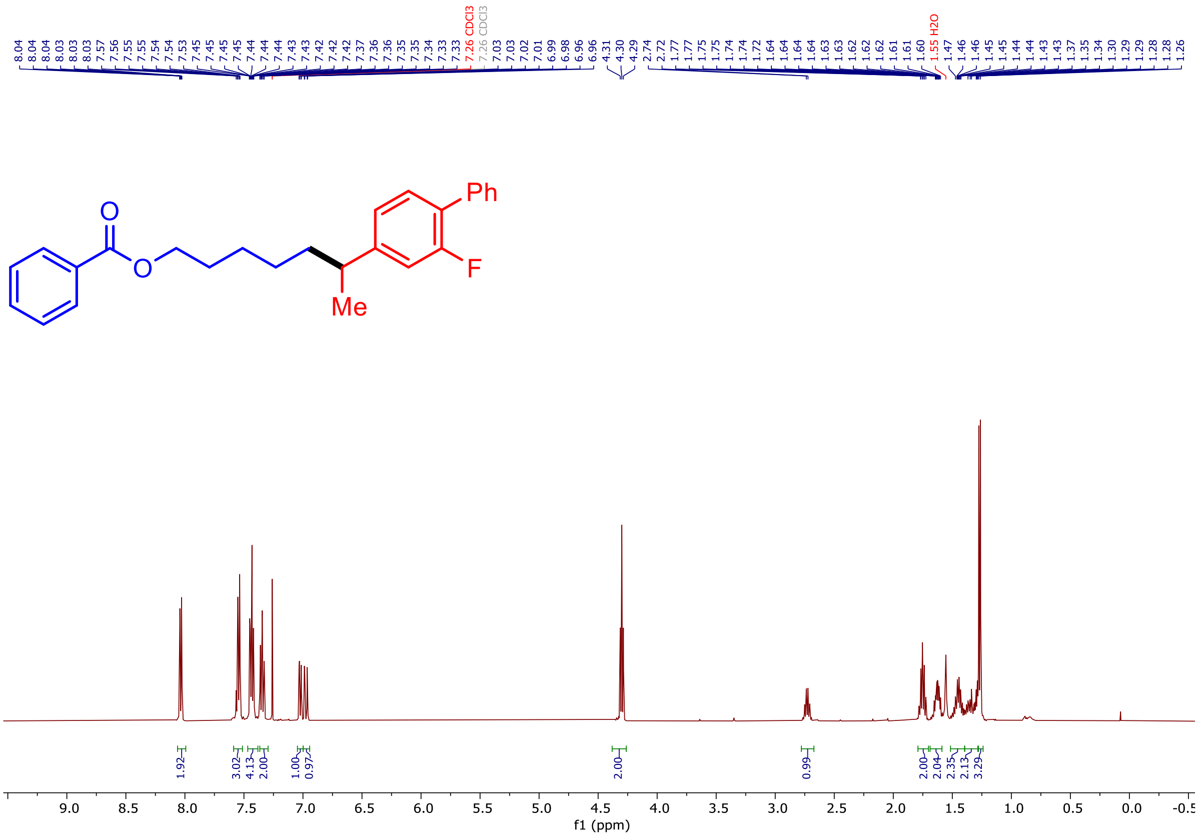

^{13}C NMR (101 MHz, $CDCl_3$) of **25**

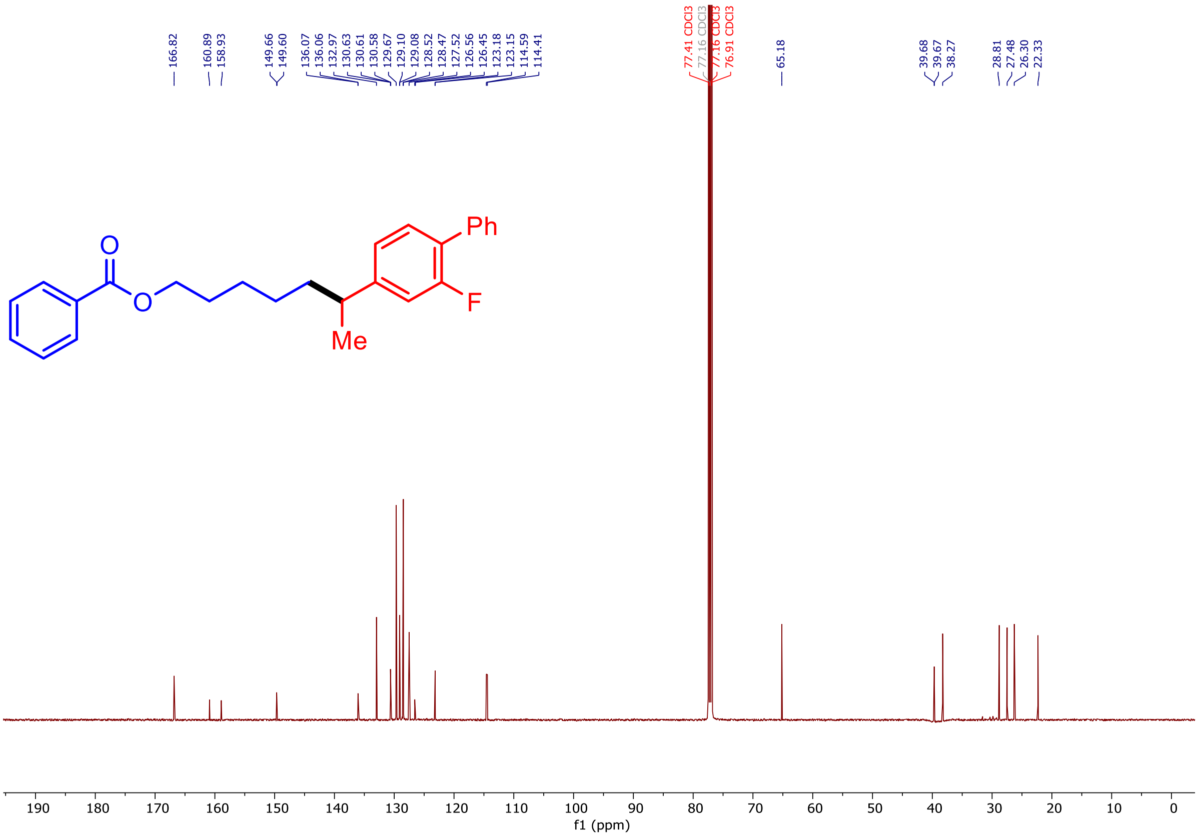

^{19}F NMR (377 MHz, $CDCl_3$) of **25**

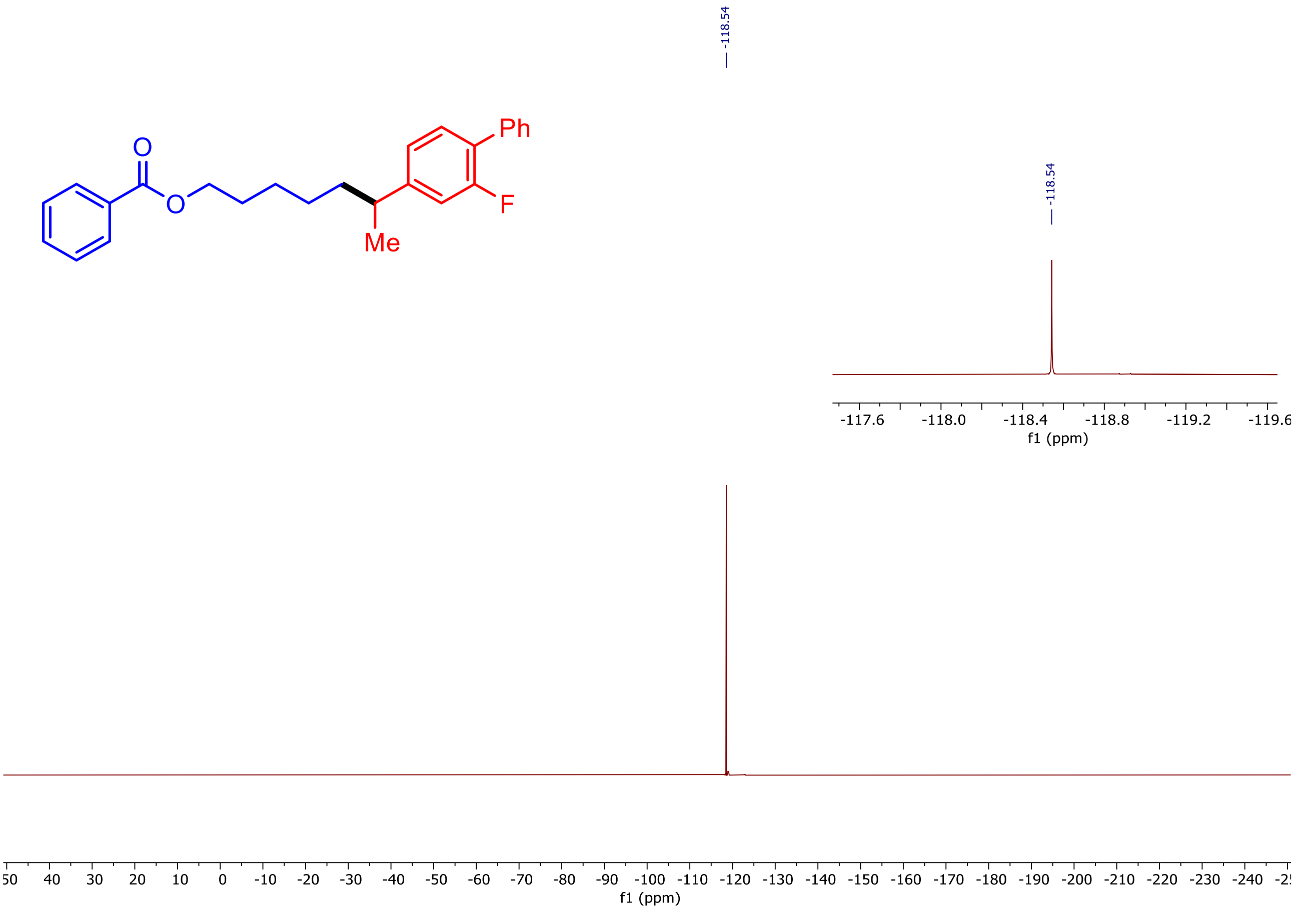

^{1}H NMR (400 MHz, $CDCl_3$) of **26** (***see procedure***)

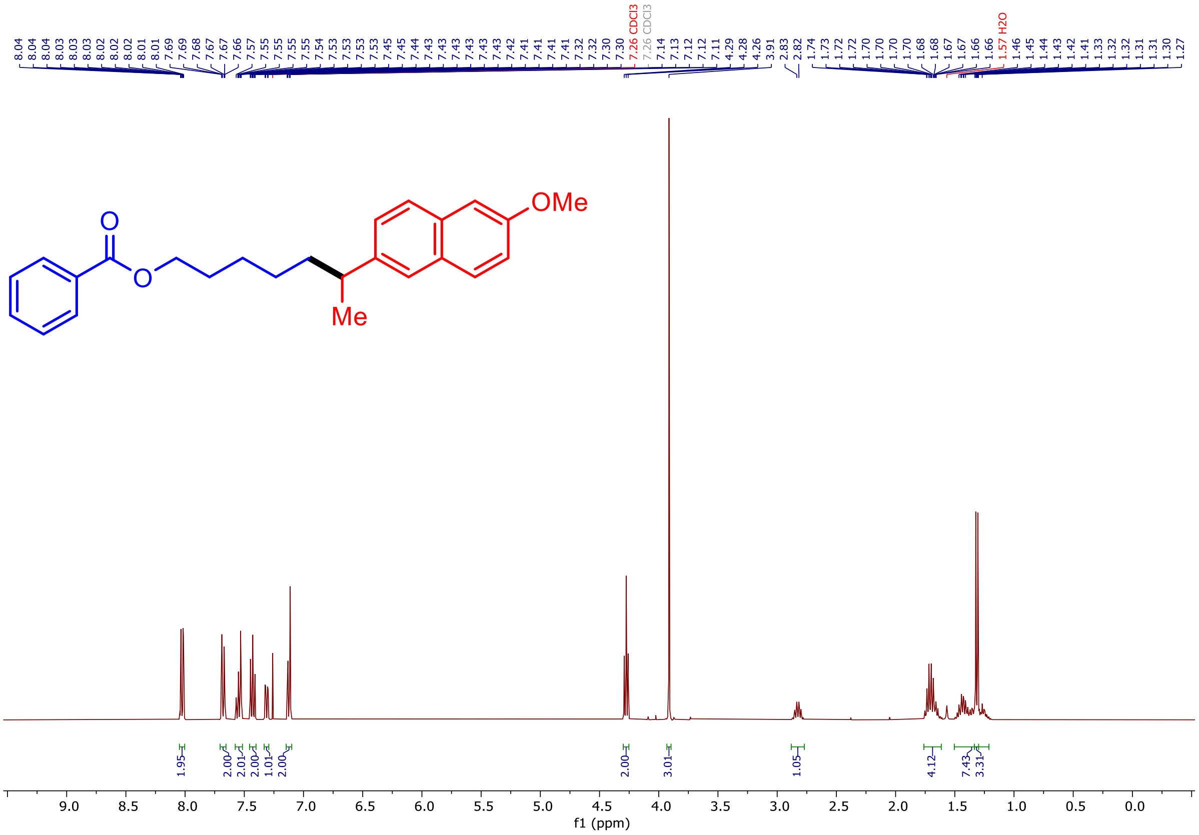

^{13}C NMR (101 MHz, $CDCl_3$) of **26**

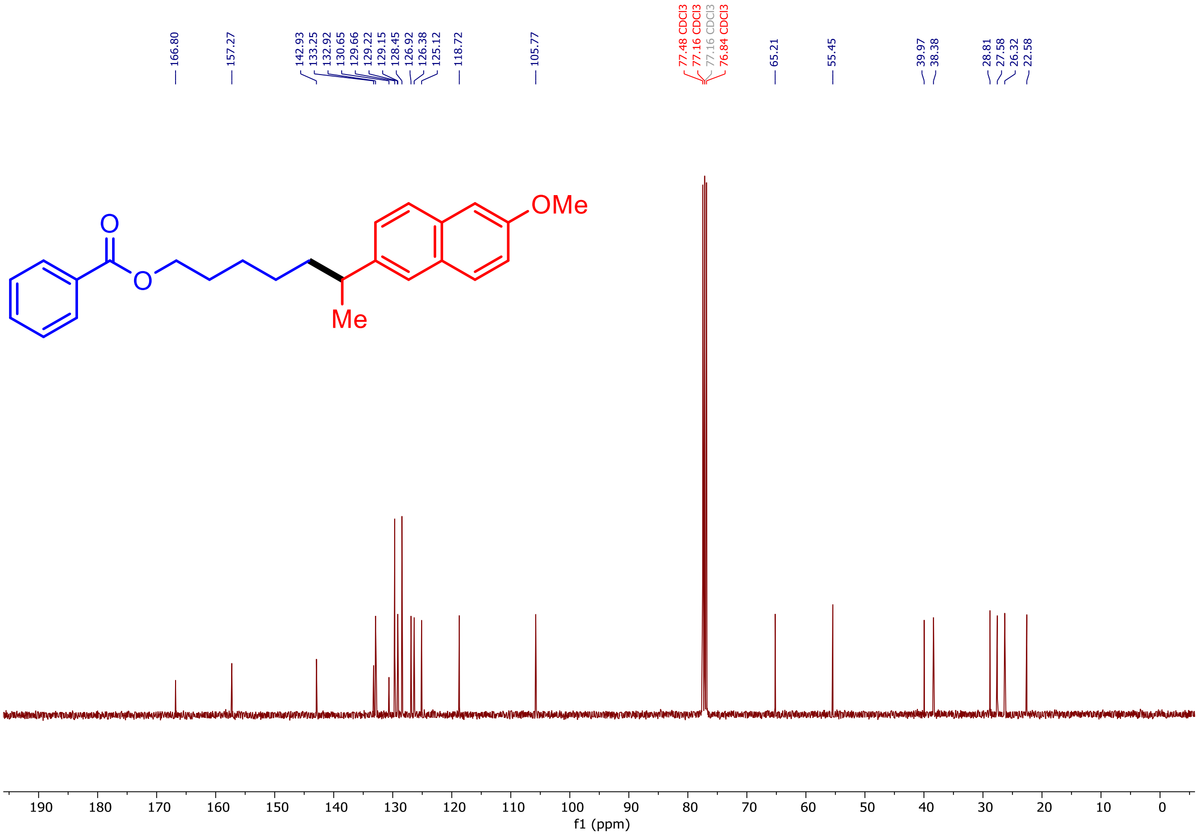

^{1}H NMR (400 MHz, $CDCl_3$) of **R27** (*see procedure*)

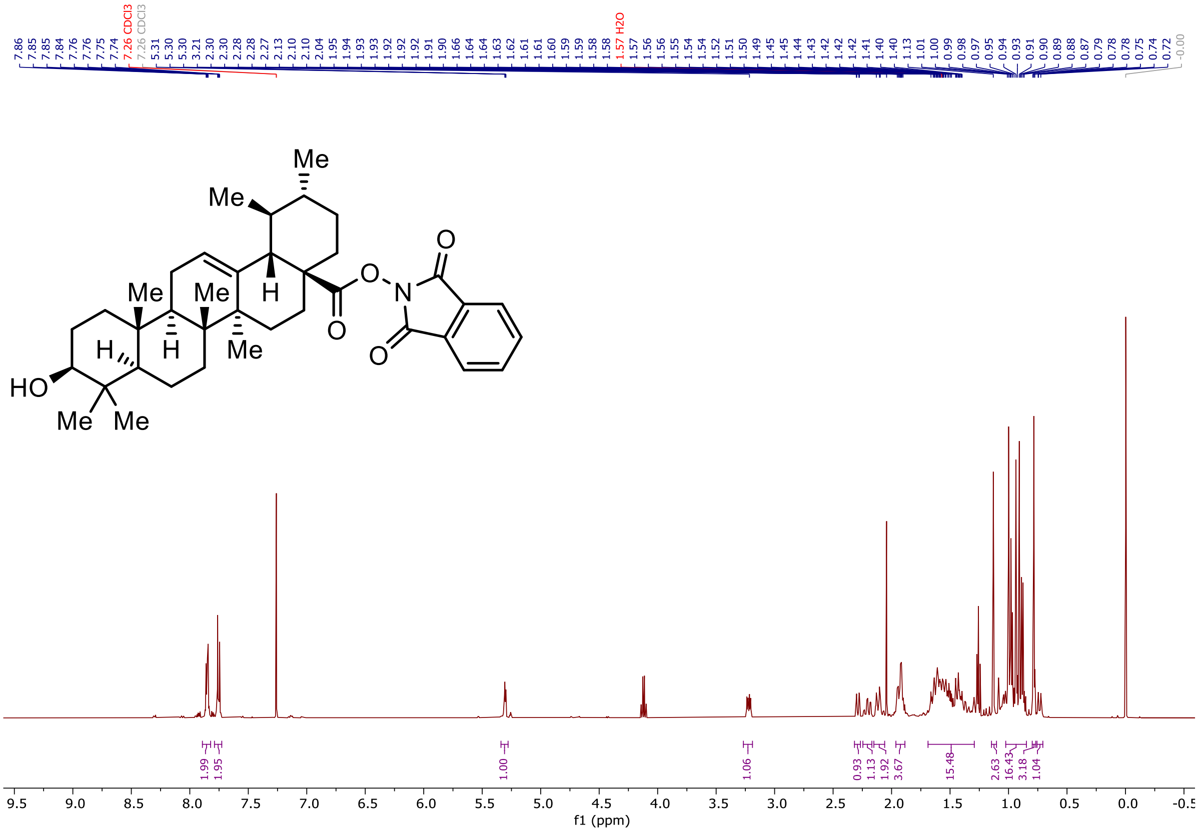

^{13}C NMR (101 MHz, $CDCl_3$) of **R27**

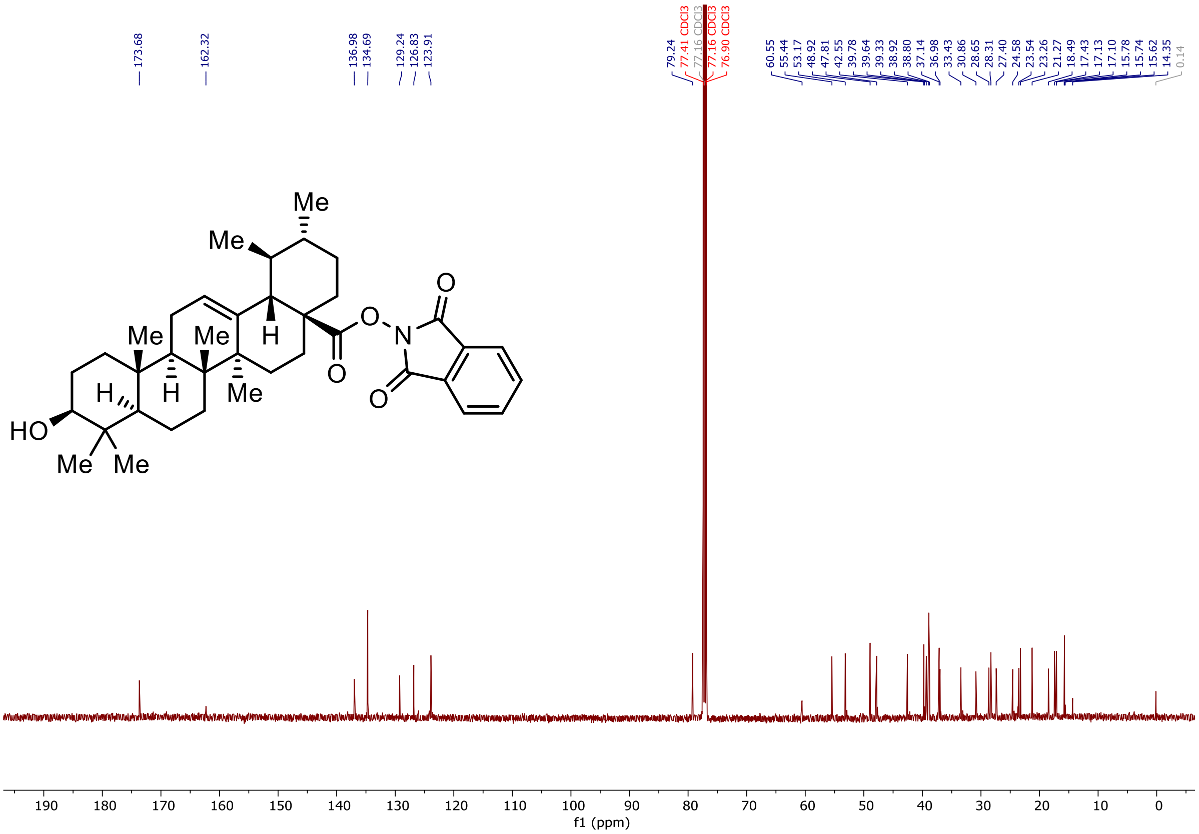

^{1}H NMR (400 MHz, $CDCl_3$) of **27** (***see procedure***)

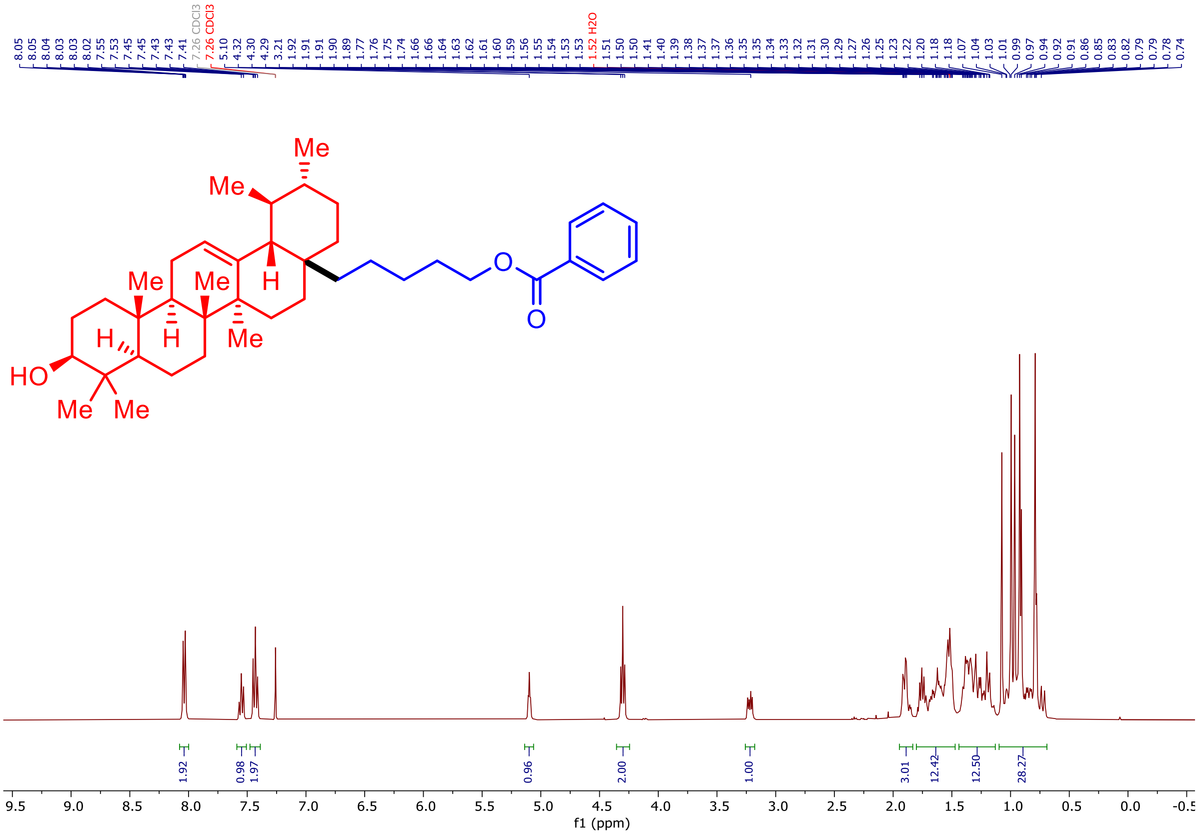

^{13}C NMR (101 MHz, $CDCl_3$) of **27**

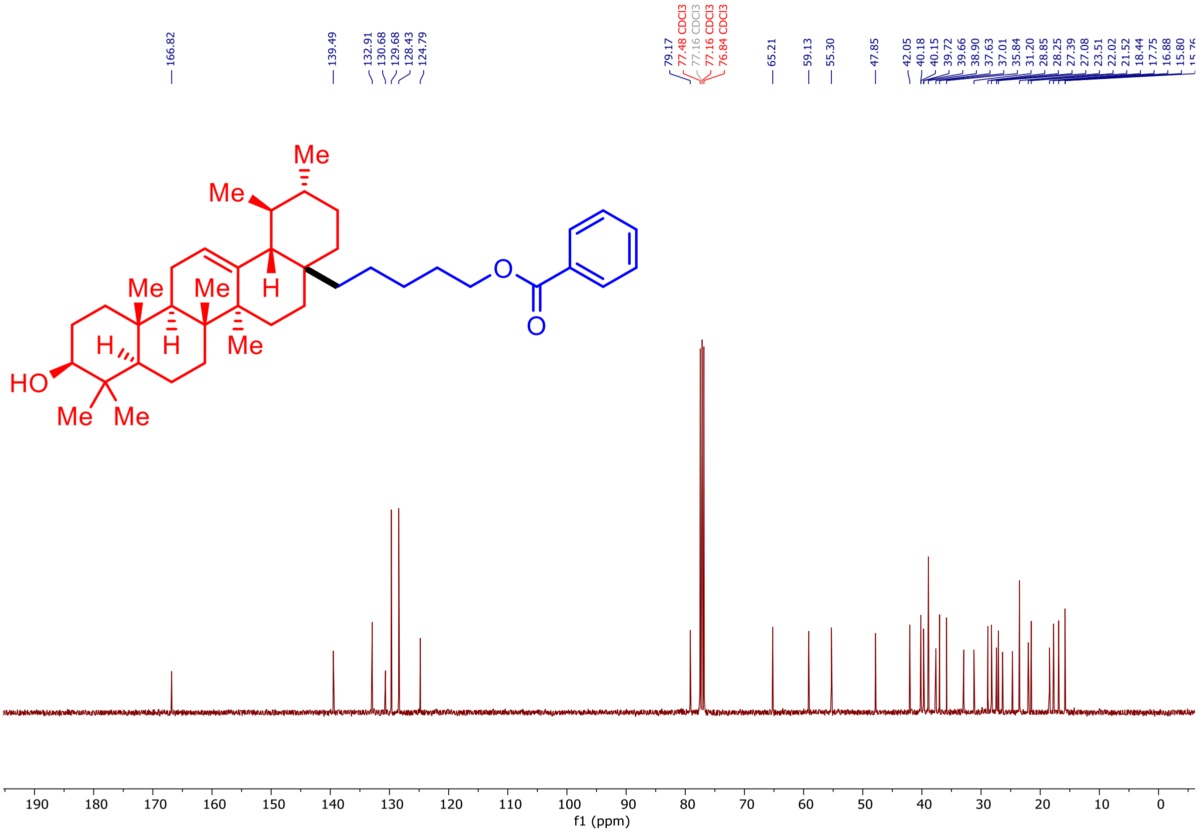

^{1}H NMR (400 MHz, $CDCl_3$) of **28** (***see procedure***)

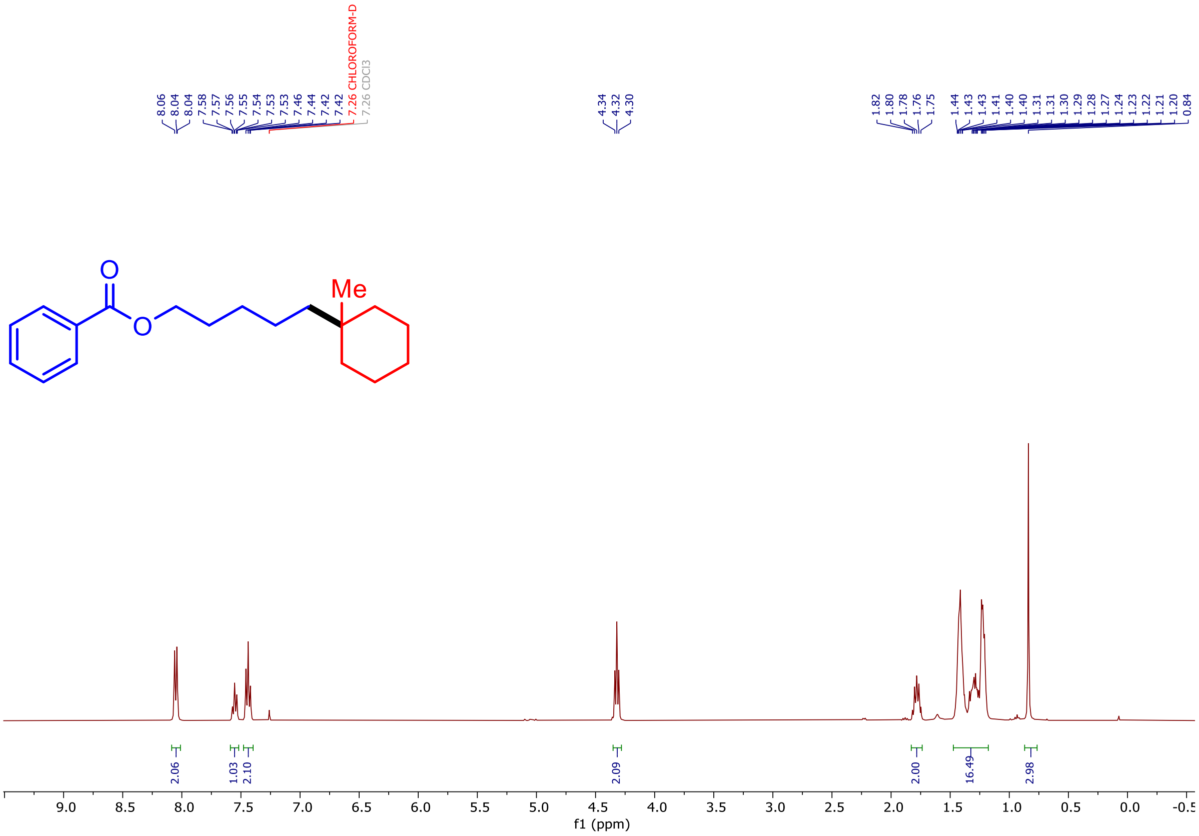

^{13}C NMR (101 MHz, $CDCl_3$) of **28**

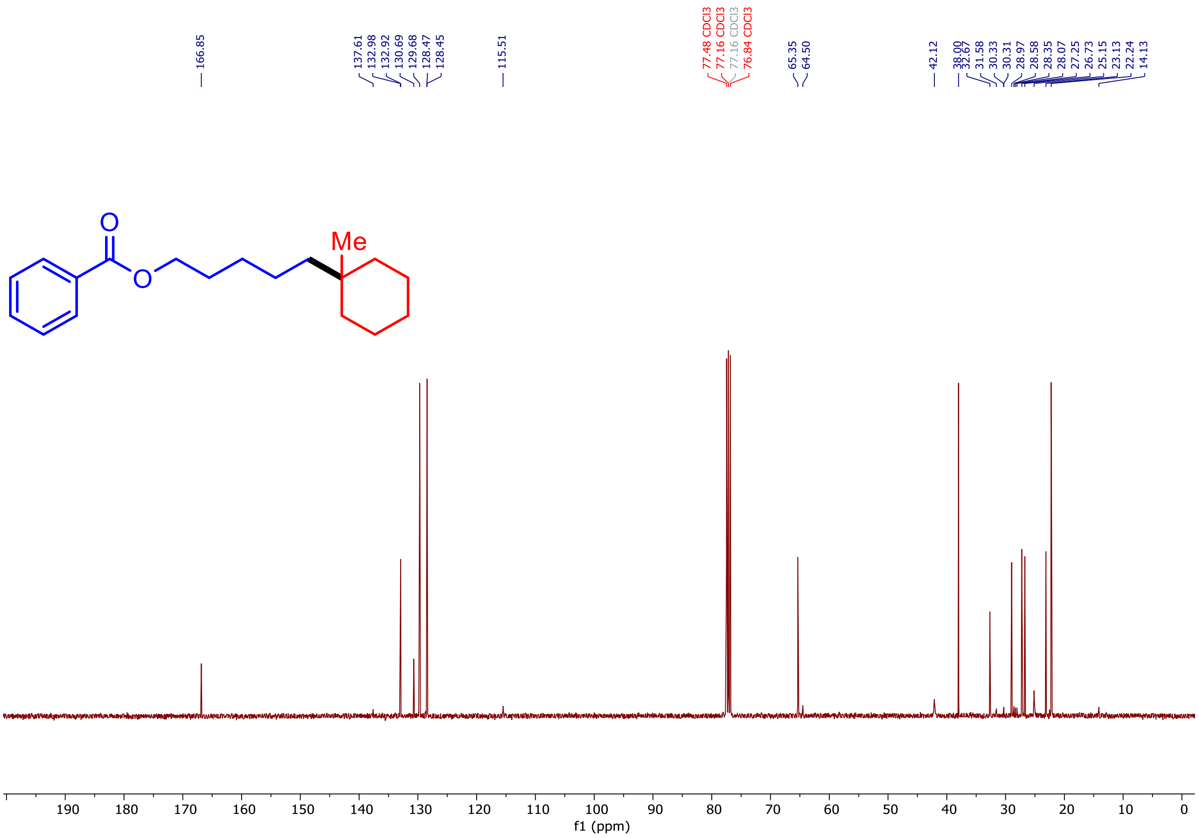

^{1}H NMR (400 MHz, $CDCl_3$) of **29** (***see procedure***)

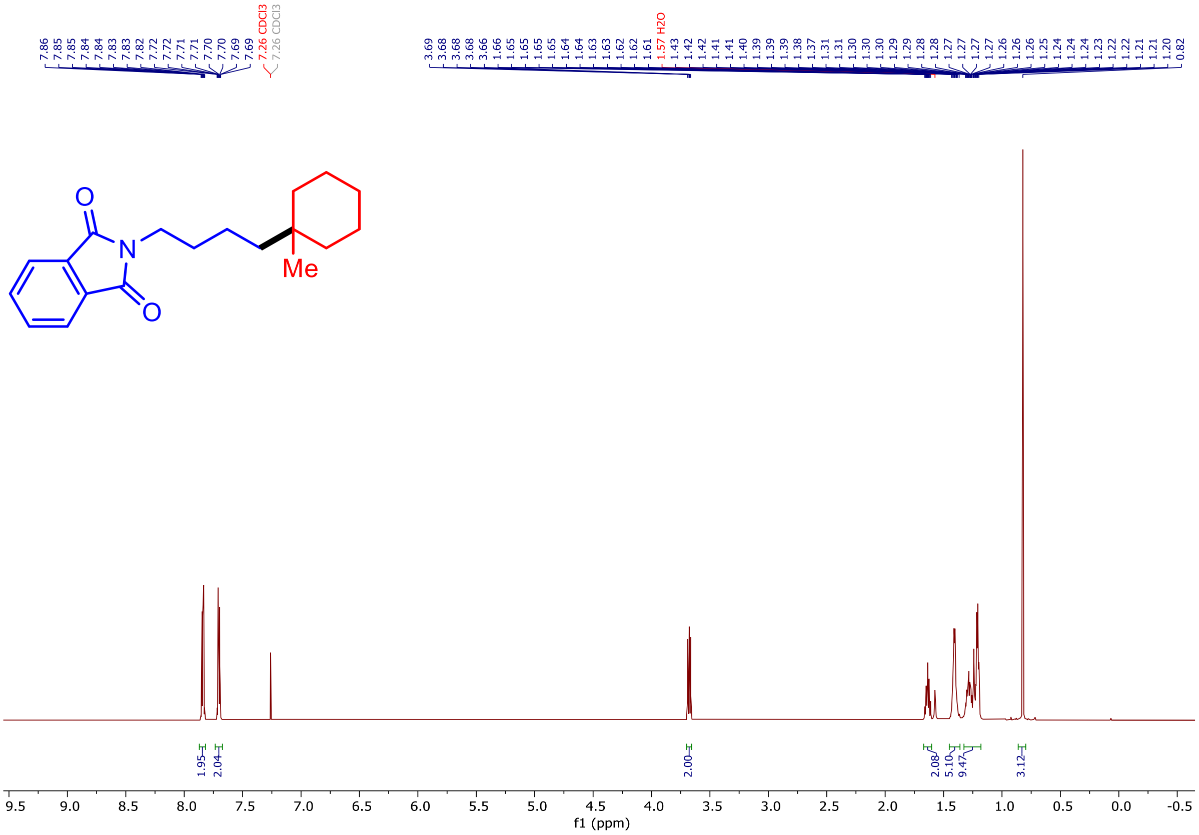

^{13}C NMR (101 MHz, $CDCl_3$) of **29**

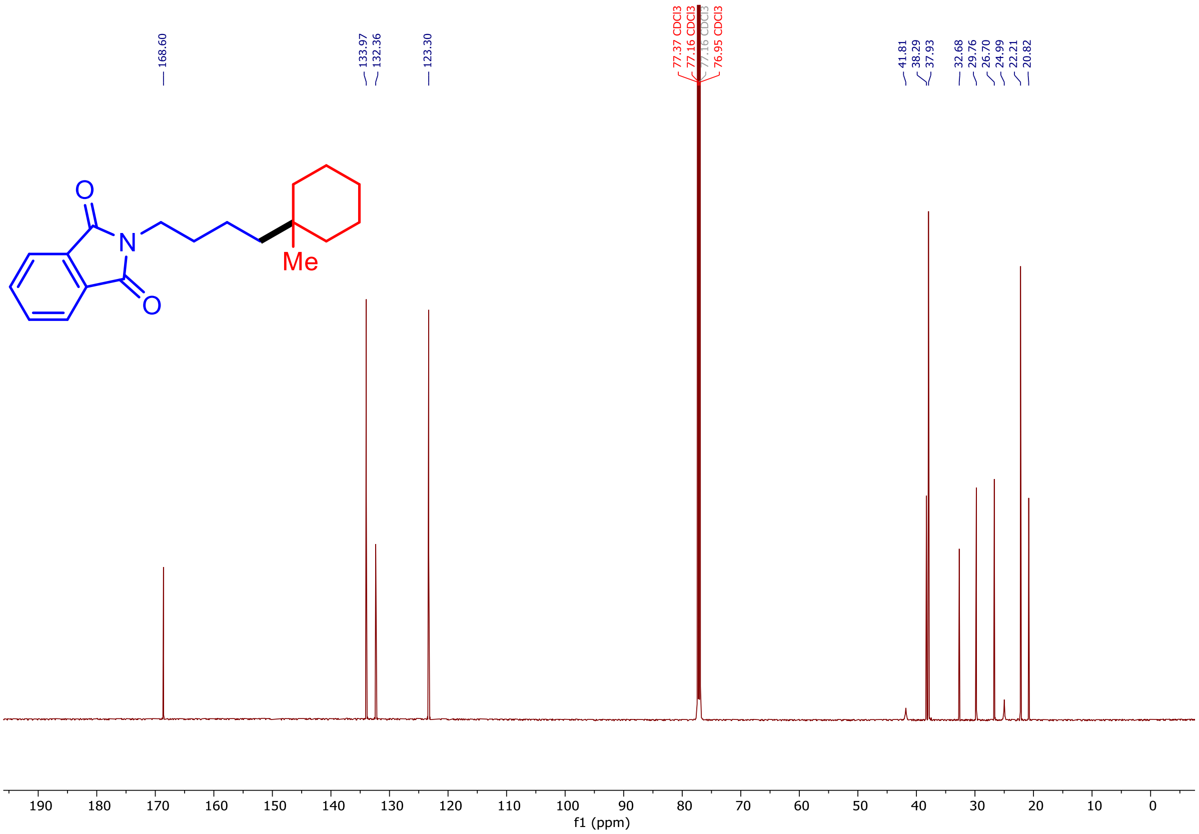

^{1}H NMR (400 MHz, $CDCl_3$) of **30** (*see procedure*)

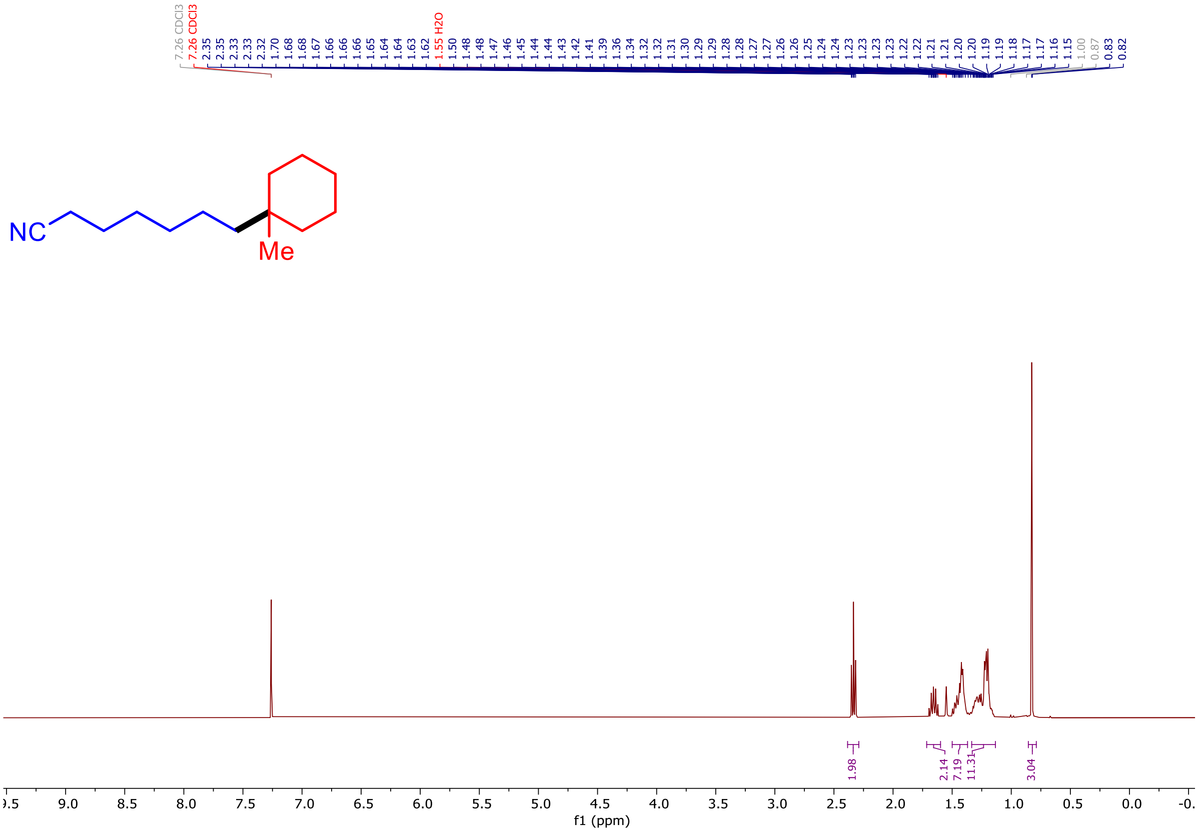

^{13}C NMR (101 MHz, $CDCl_3$) of **30**

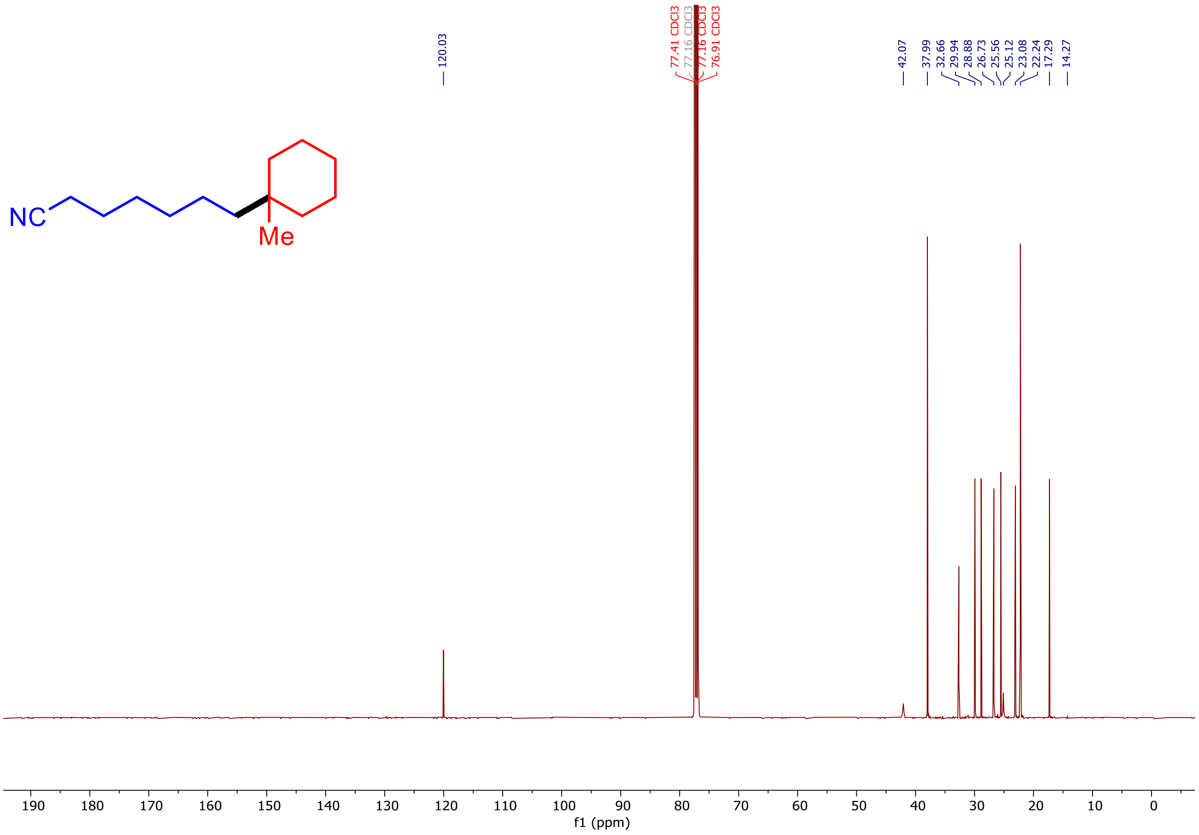

^{1}H NMR (400 MHz, $CDCl_3$) of **31** (***see procedure***)

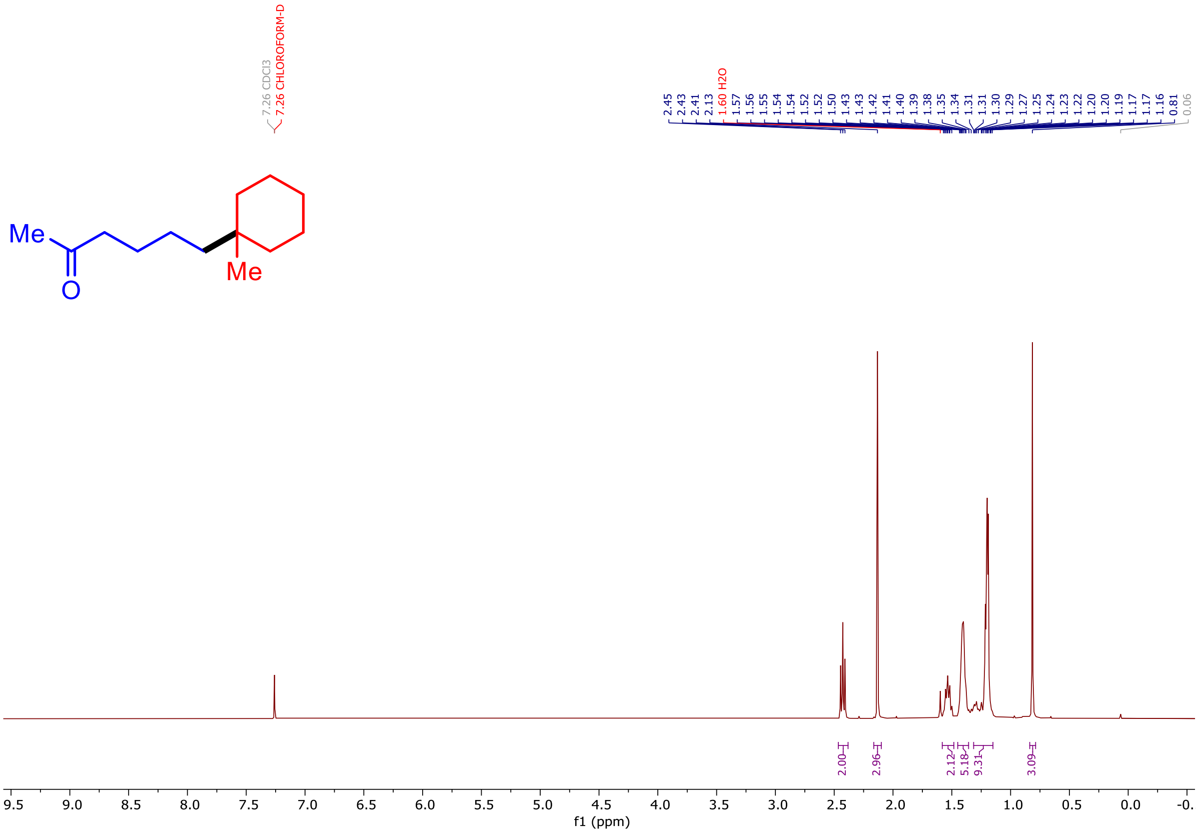

^{13}C NMR (101 MHz, $CDCl_3$) of **31**

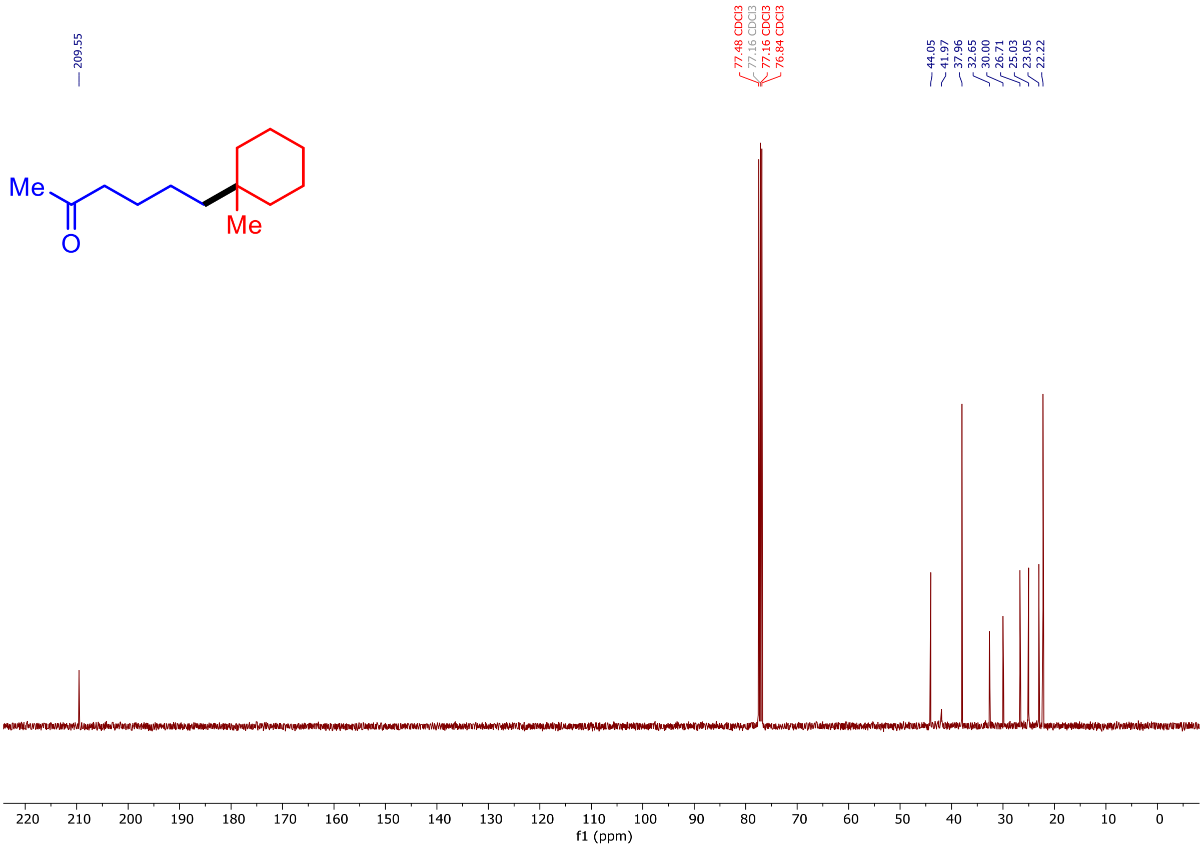

^{1}H NMR (400 MHz, $CDCl_3$) of **32** (*see procedure*)

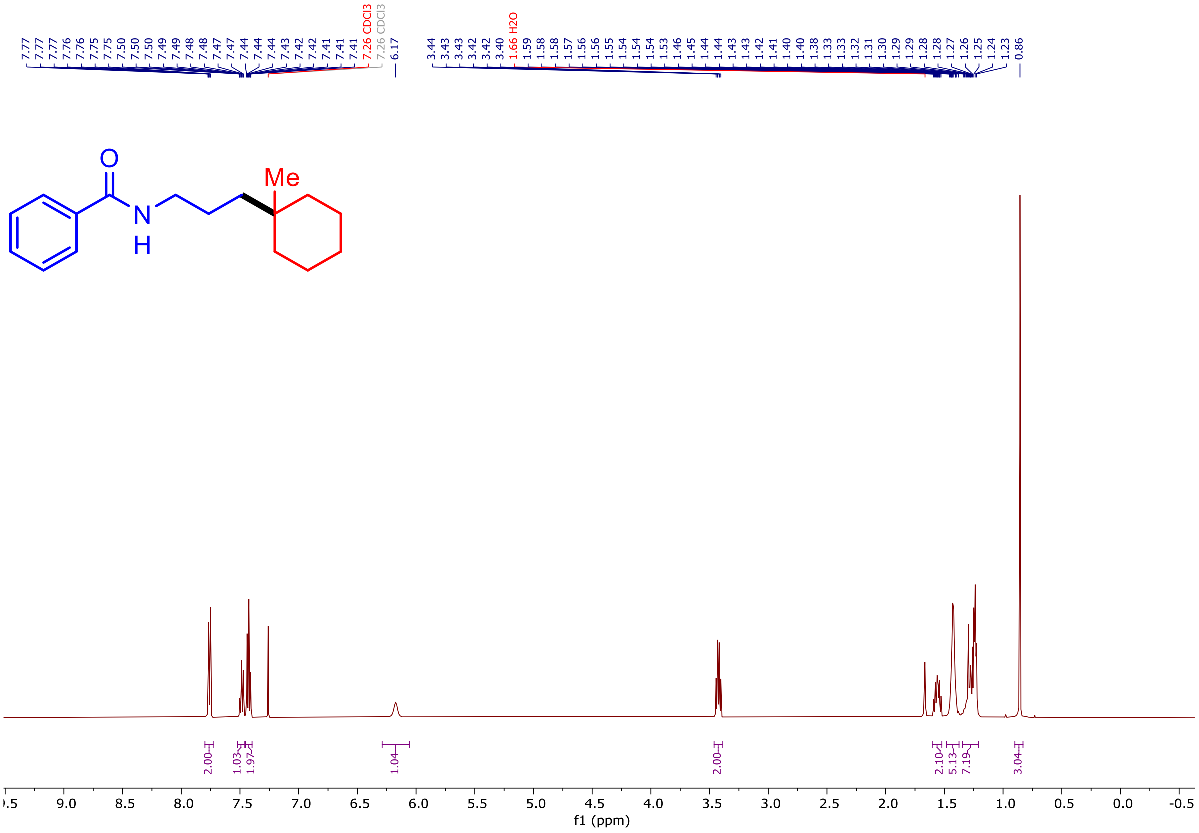

^{13}C NMR (101 MHz, $CDCl_3$) of **32**

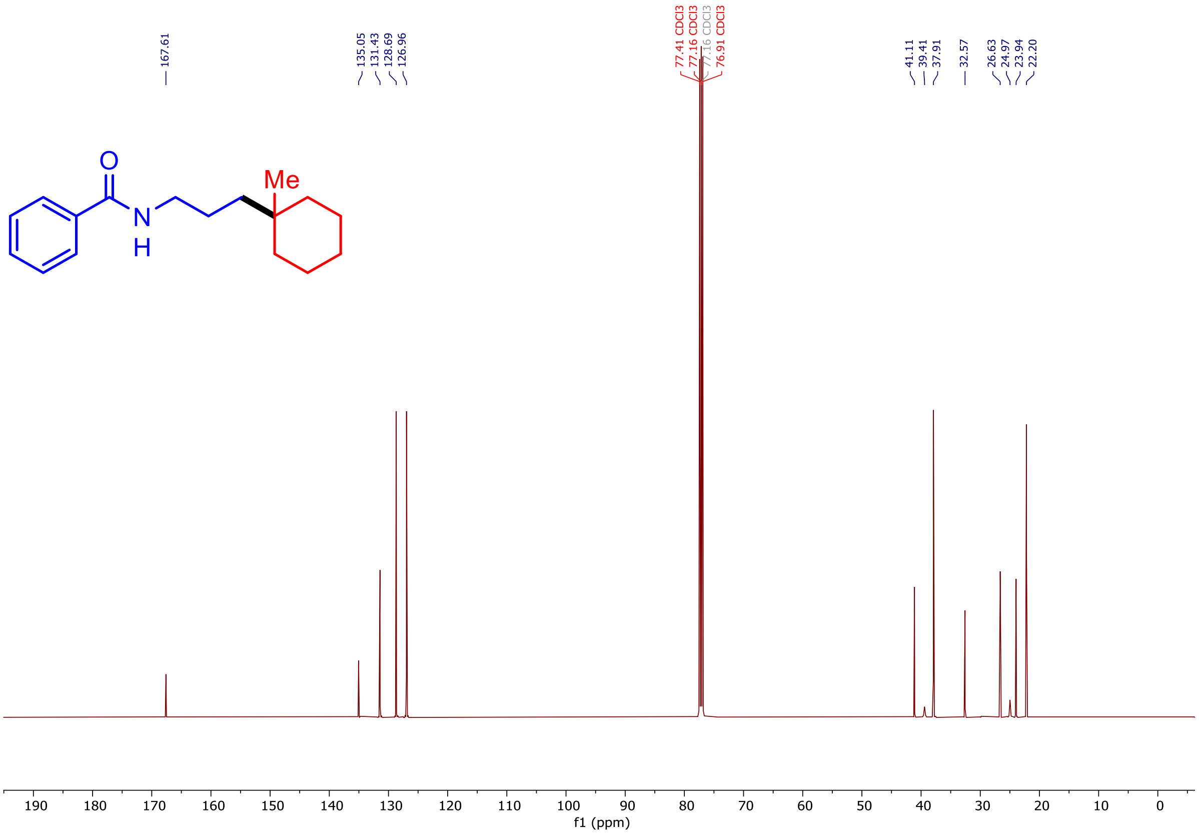

^{1}H NMR (400 MHz, $CDCl_3$) of **33** (***see procedure***)

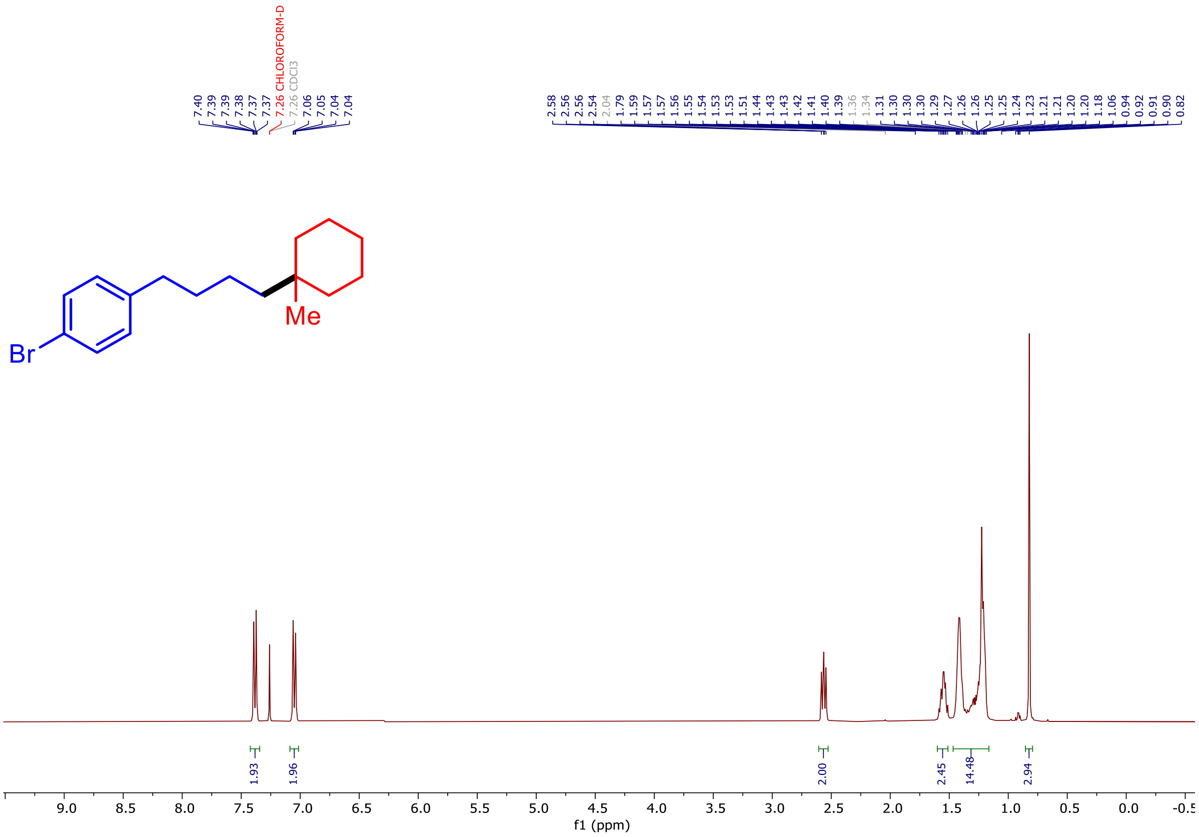

^{13}C NMR (101 MHz, $CDCl_3$) of **33**

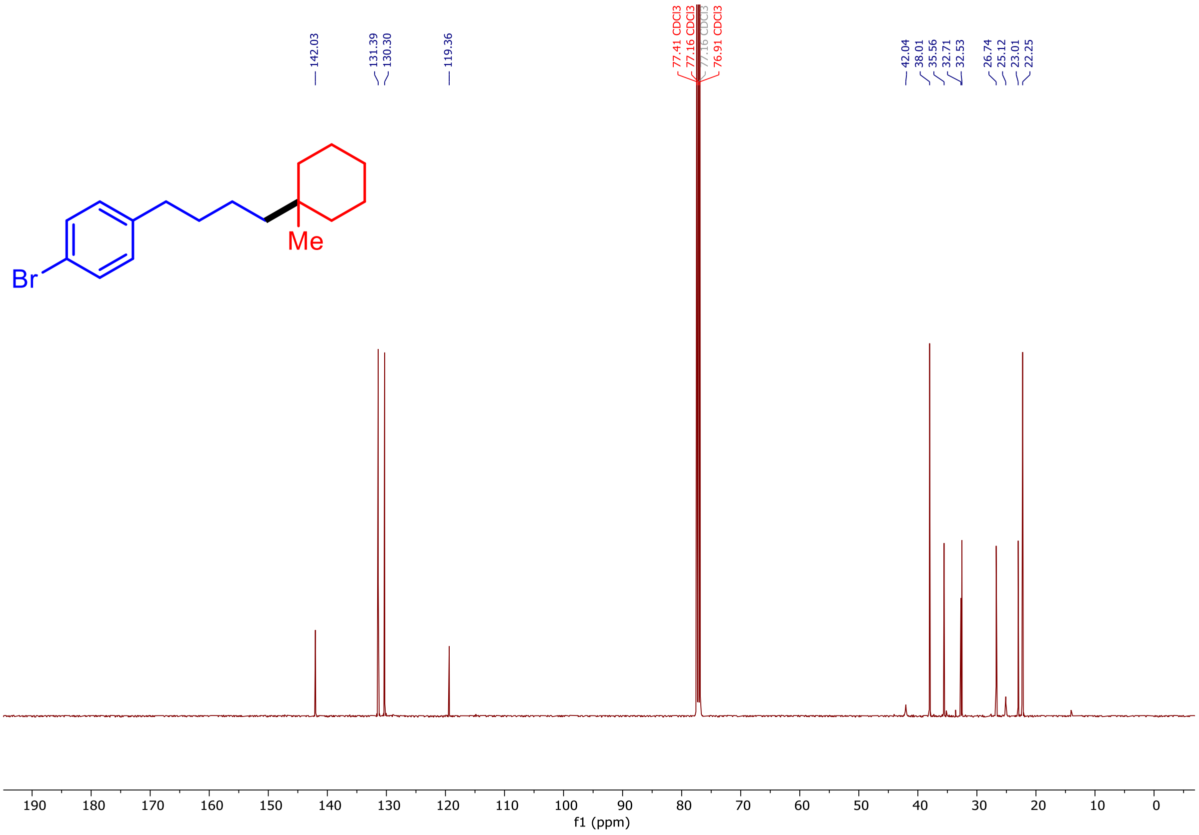

^{1}H NMR (400 MHz, $CDCl_3$) of **34** (*see procedure*)

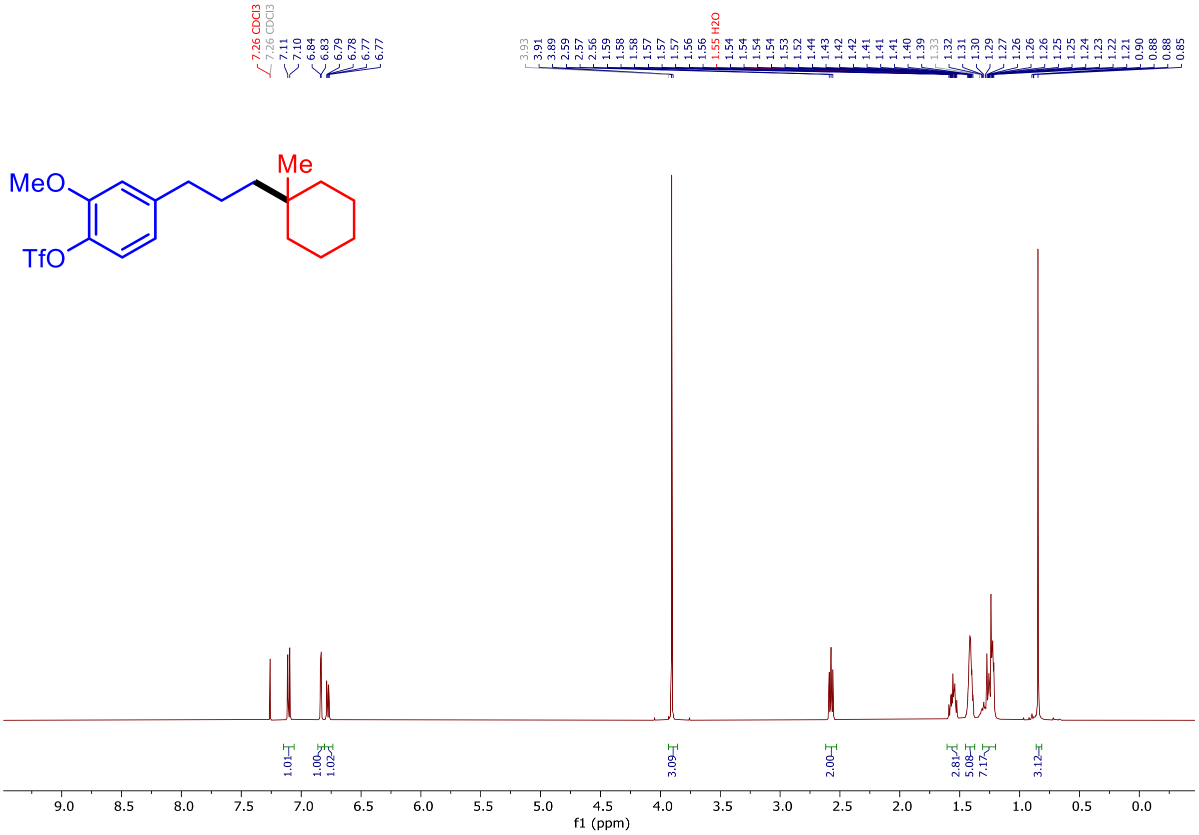

^{13}C NMR (101 MHz, $CDCl_3$) of **34**

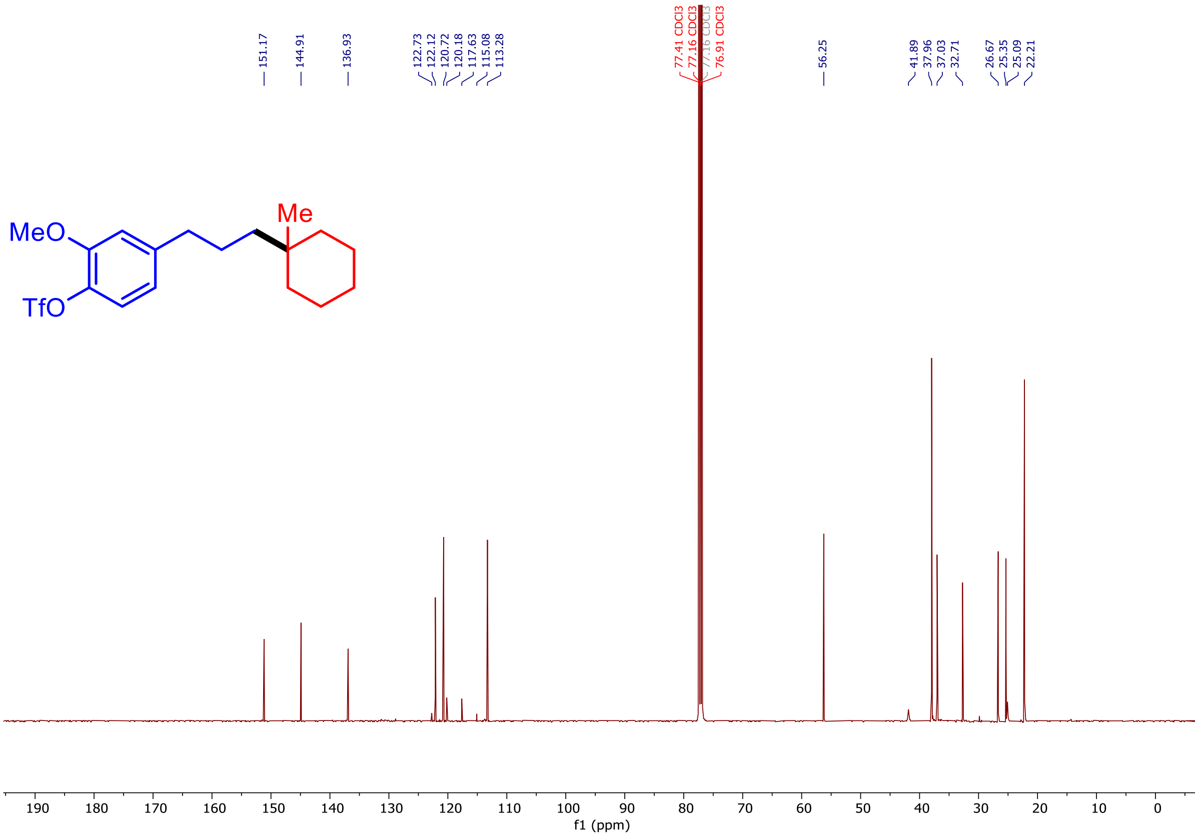

^{19}F NMR (377 MHz, $CDCl_3$) of **34**

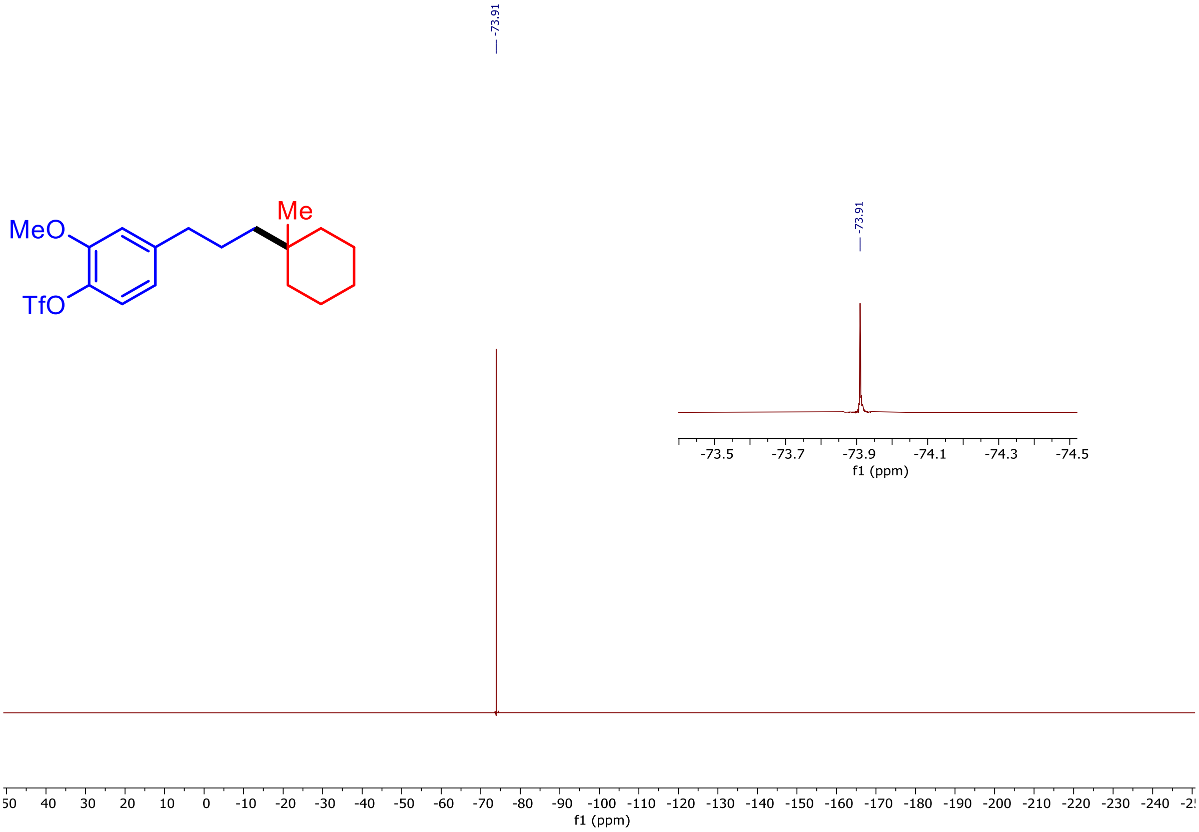

^{1}H NMR (400 MHz, $CDCl_3$) of **35a** (***see procedure***)

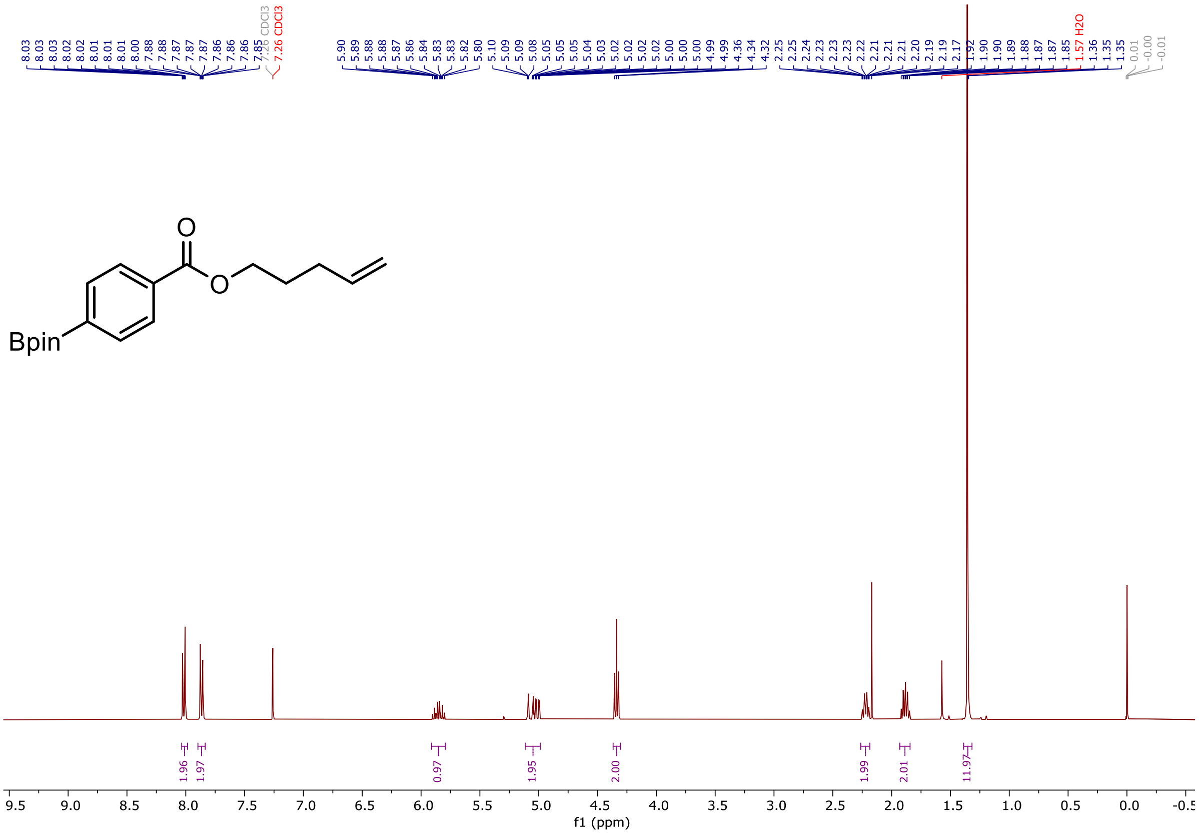

^{13}C NMR (101 MHz, $CDCl_3$) of **35a**

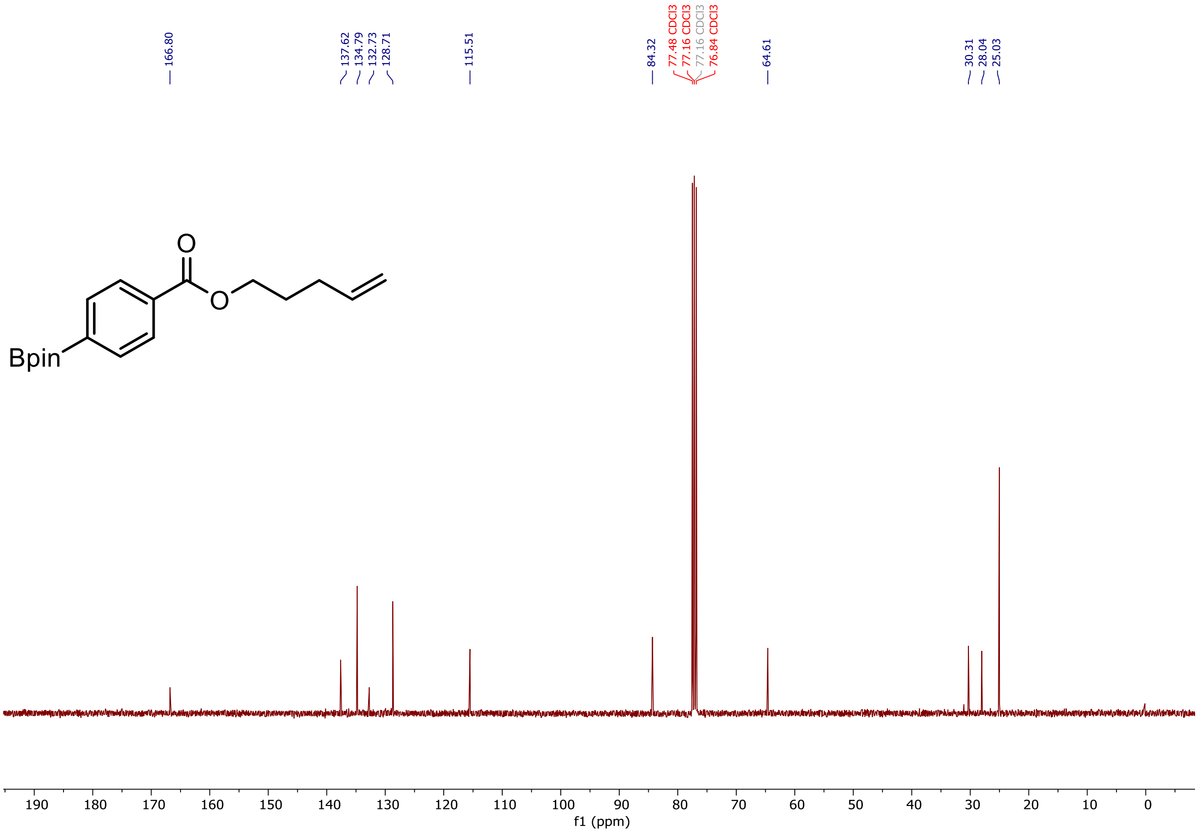

^{11}B NMR (128 MHz, $CDCl_3$) of **35a**

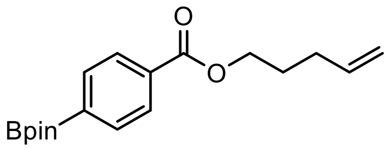

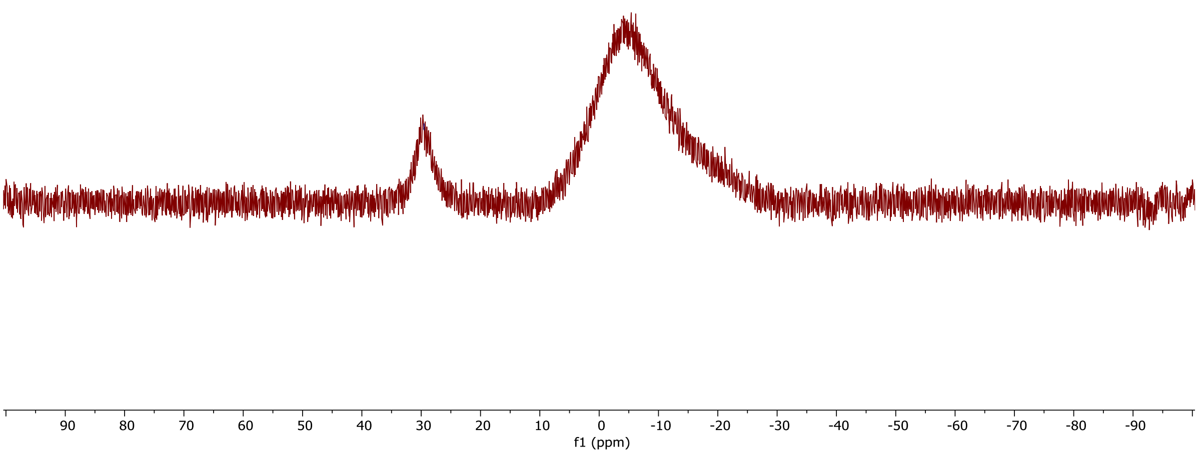

^{1}H NMR (400 MHz, $CDCl_3$) of **35** (***see procedure***)

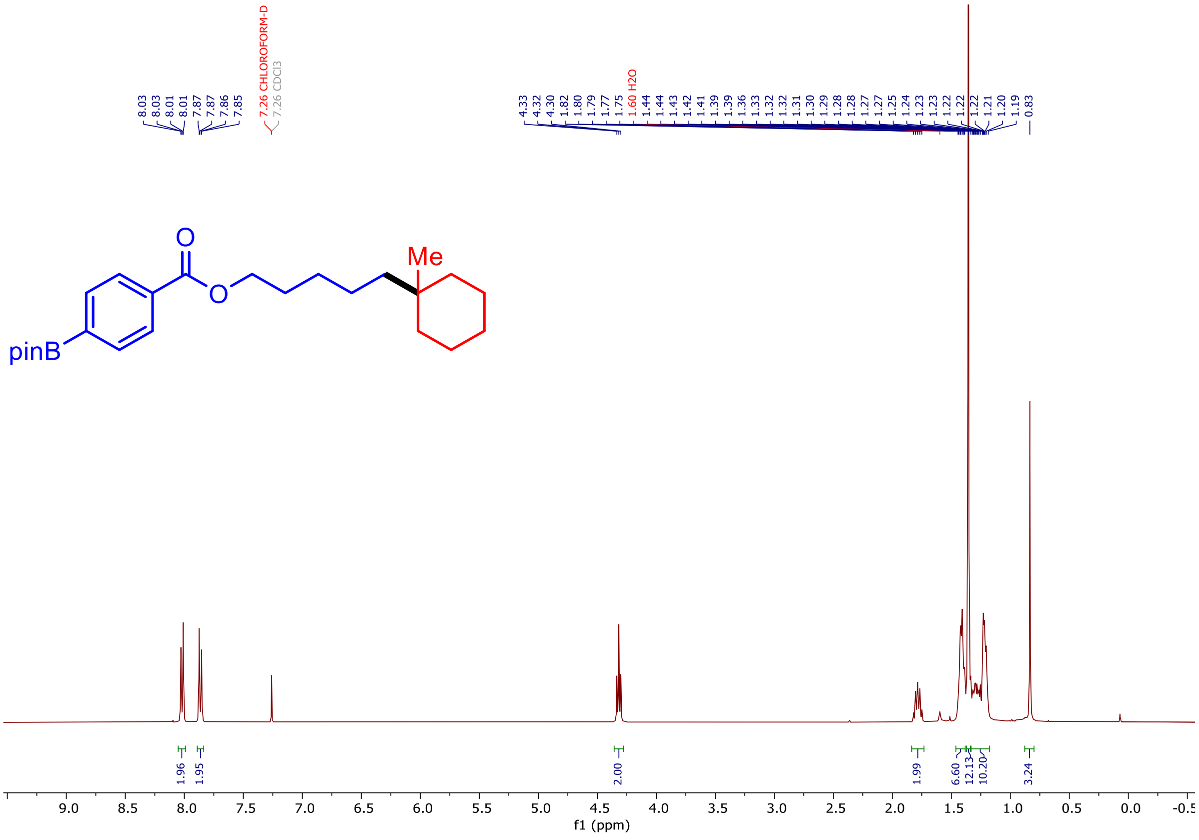

^{13}C NMR (101 MHz, $CDCl_3$) of **35**

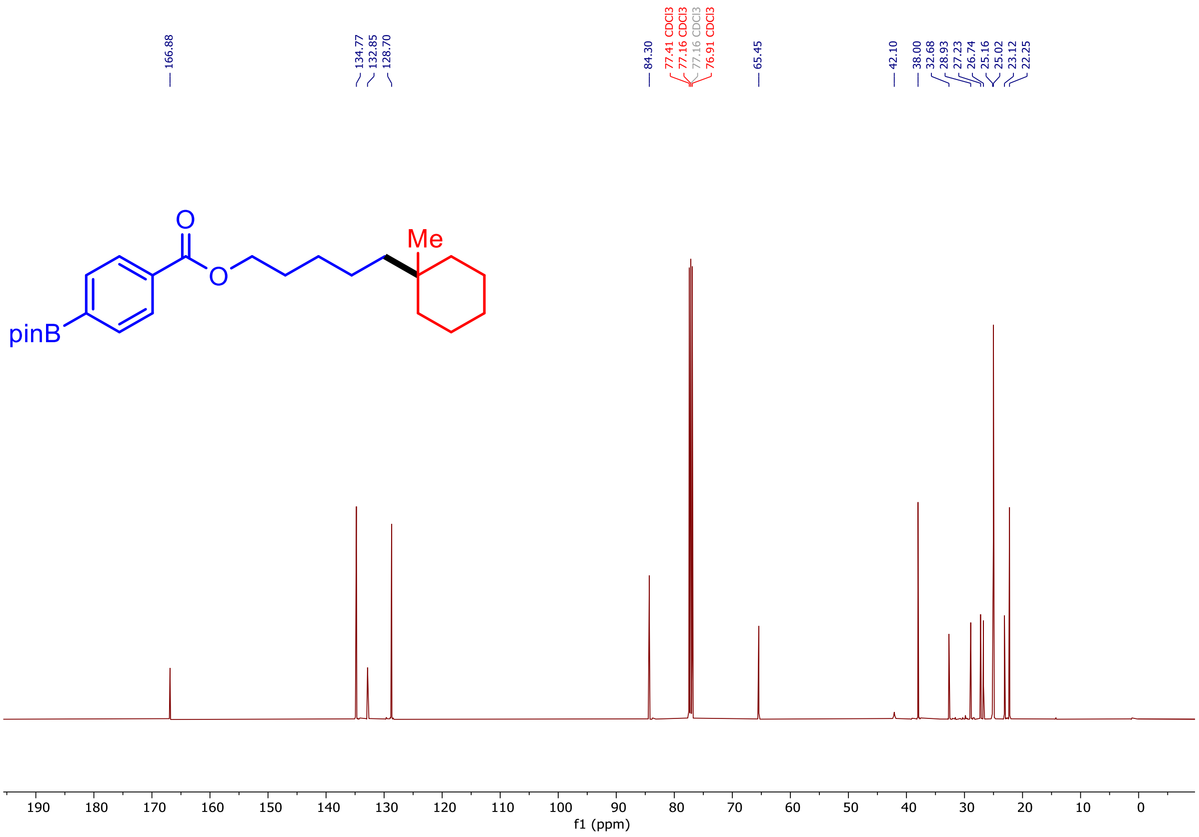

^{11}B NMR (128 MHz, $CDCl_3$) of **35**

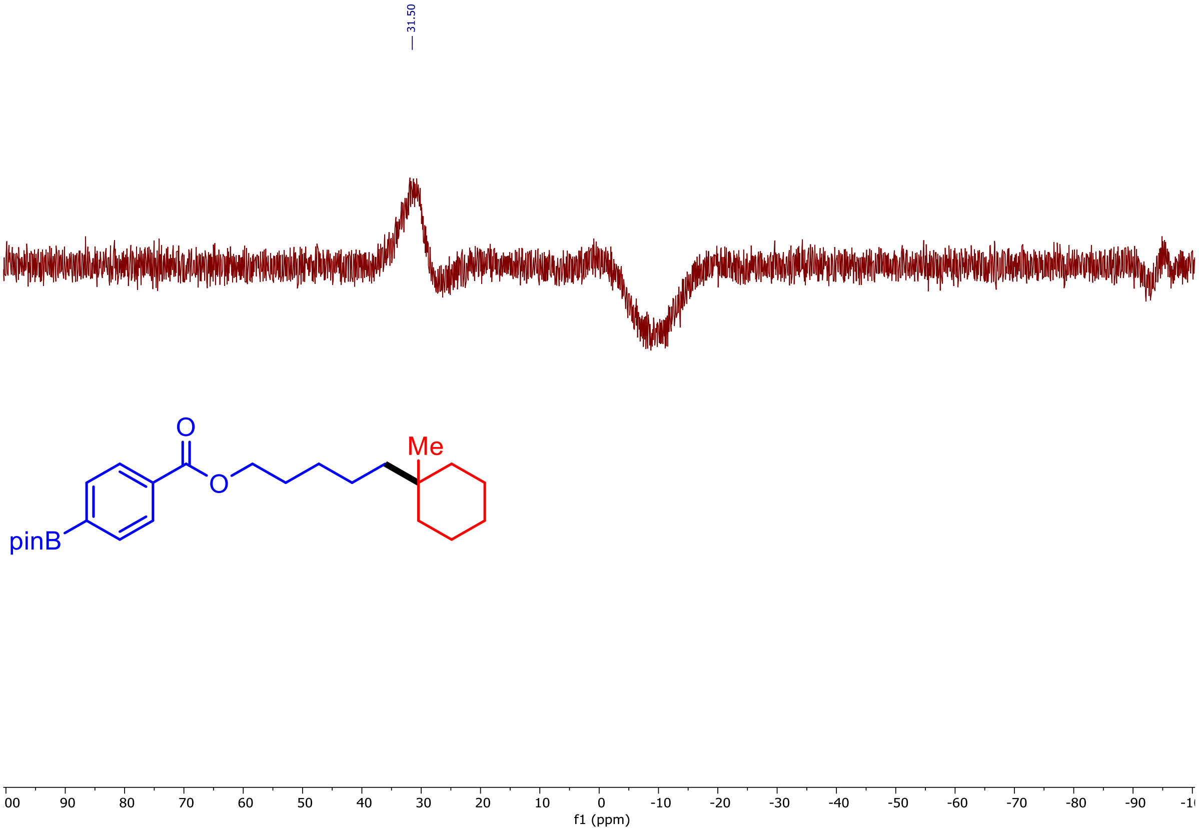

^{1}H NMR (400 MHz, $CDCl_3$) of **36** (*see procedure*)

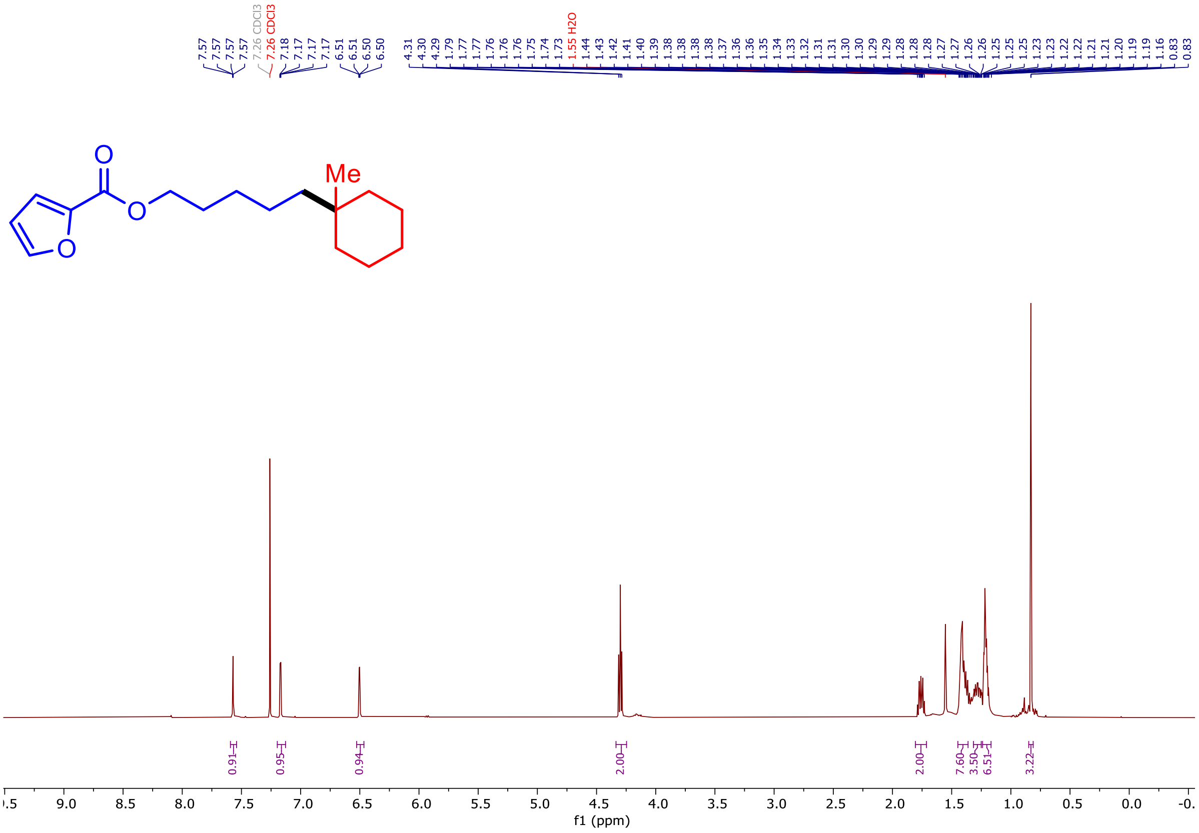

^{13}C NMR (101 MHz, $CDCl_3$) of **36**

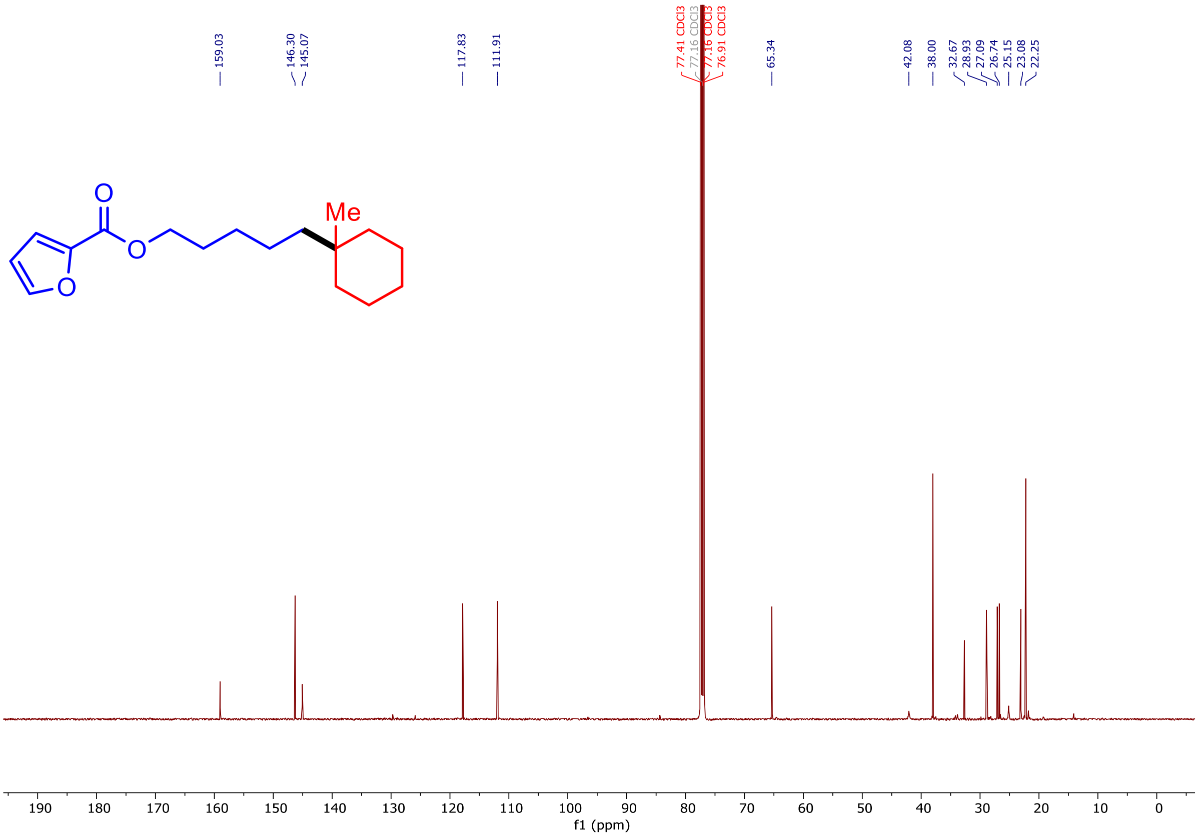

^{1}H NMR (400 MHz, $CDCl_3$) of **37a** (***see procedure***)

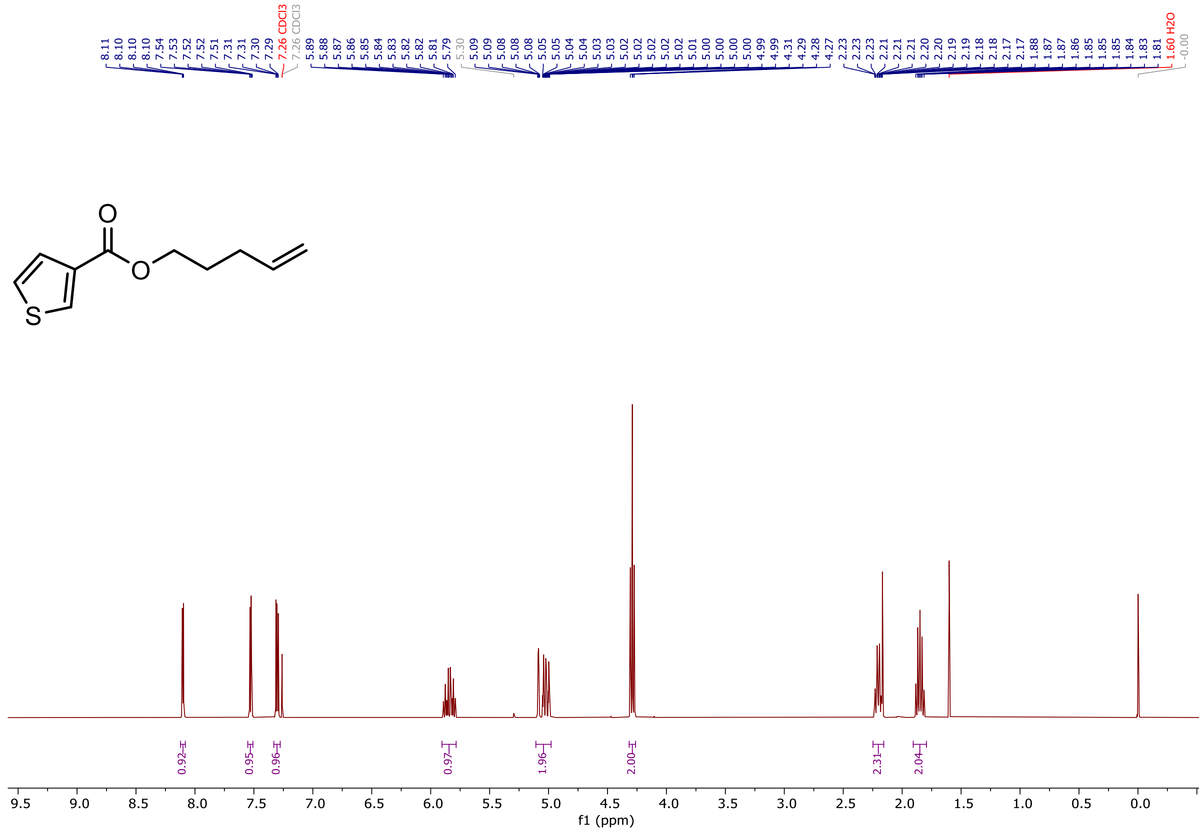

^{13}C NMR (101 MHz, $CDCl_3$) of **37a**

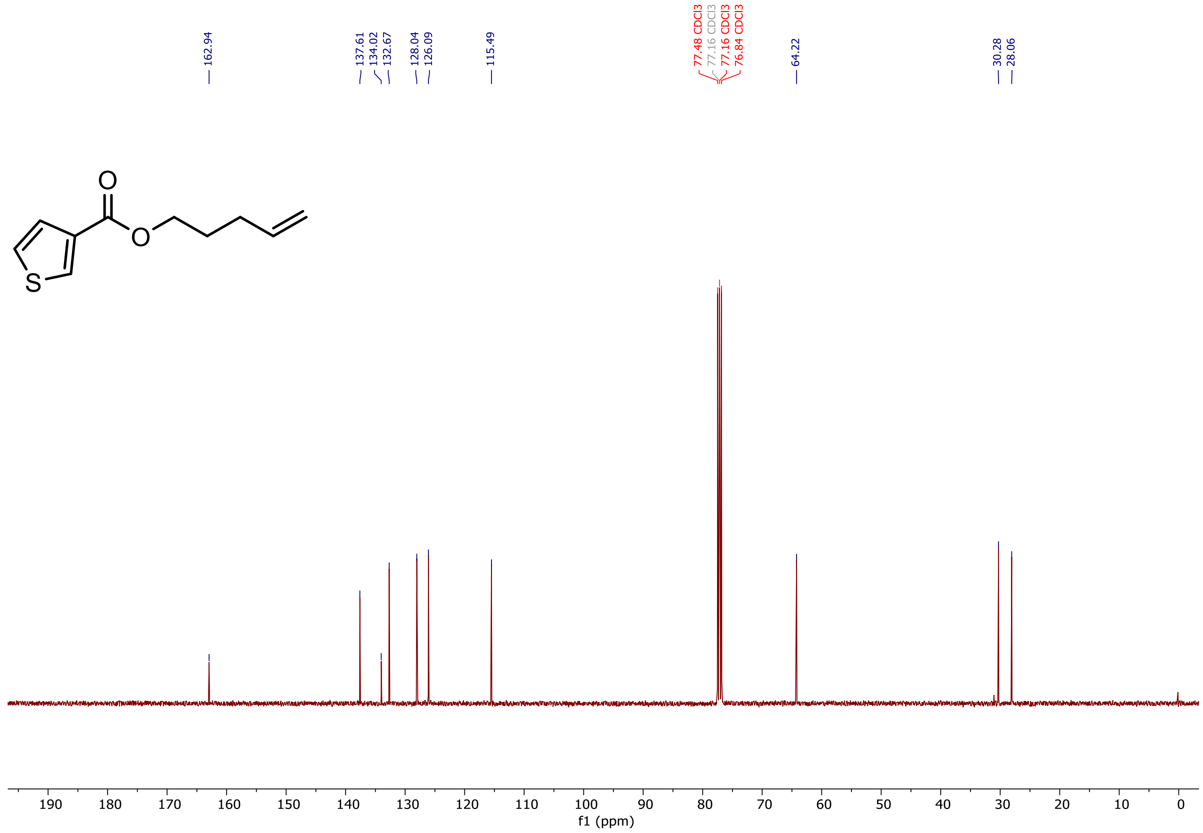

^{1}H NMR (400 MHz, $CDCl_3$) of **37** (*see procedure*)

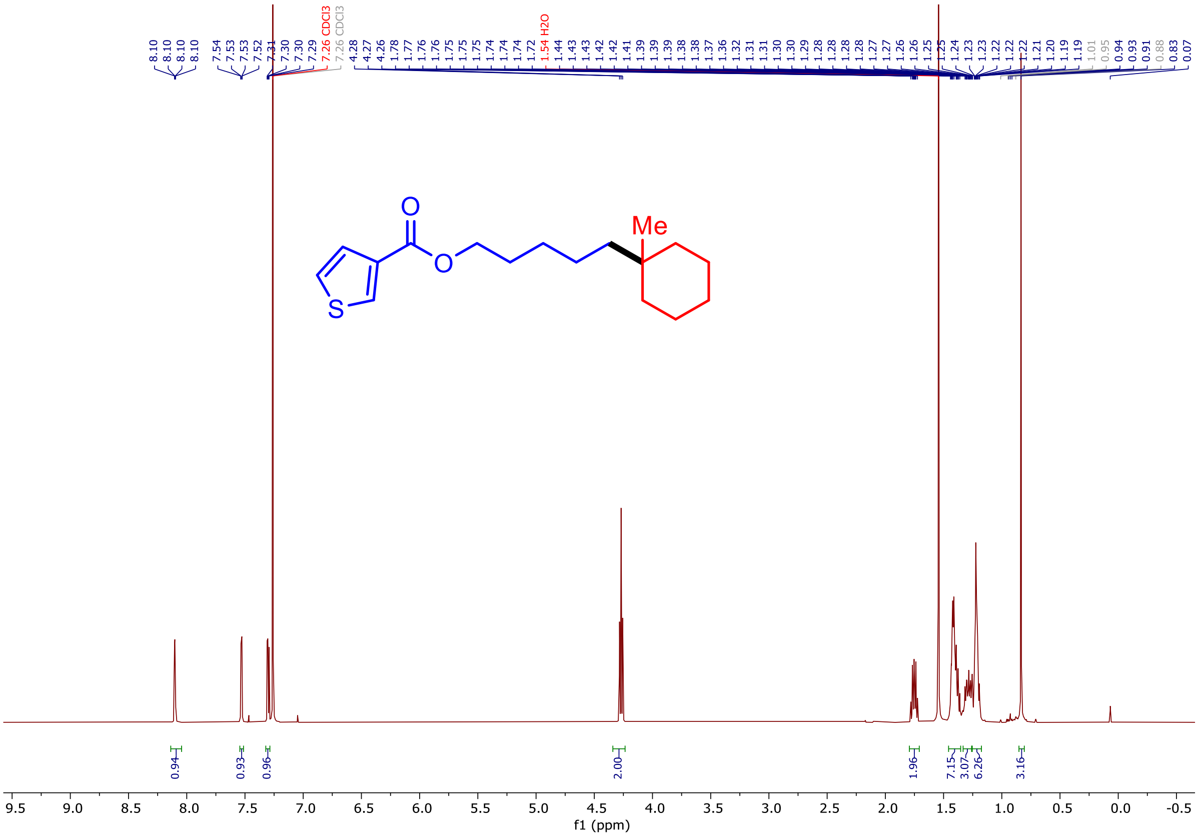

^{13}C NMR (101 MHz, $CDCl_3$) of **37**

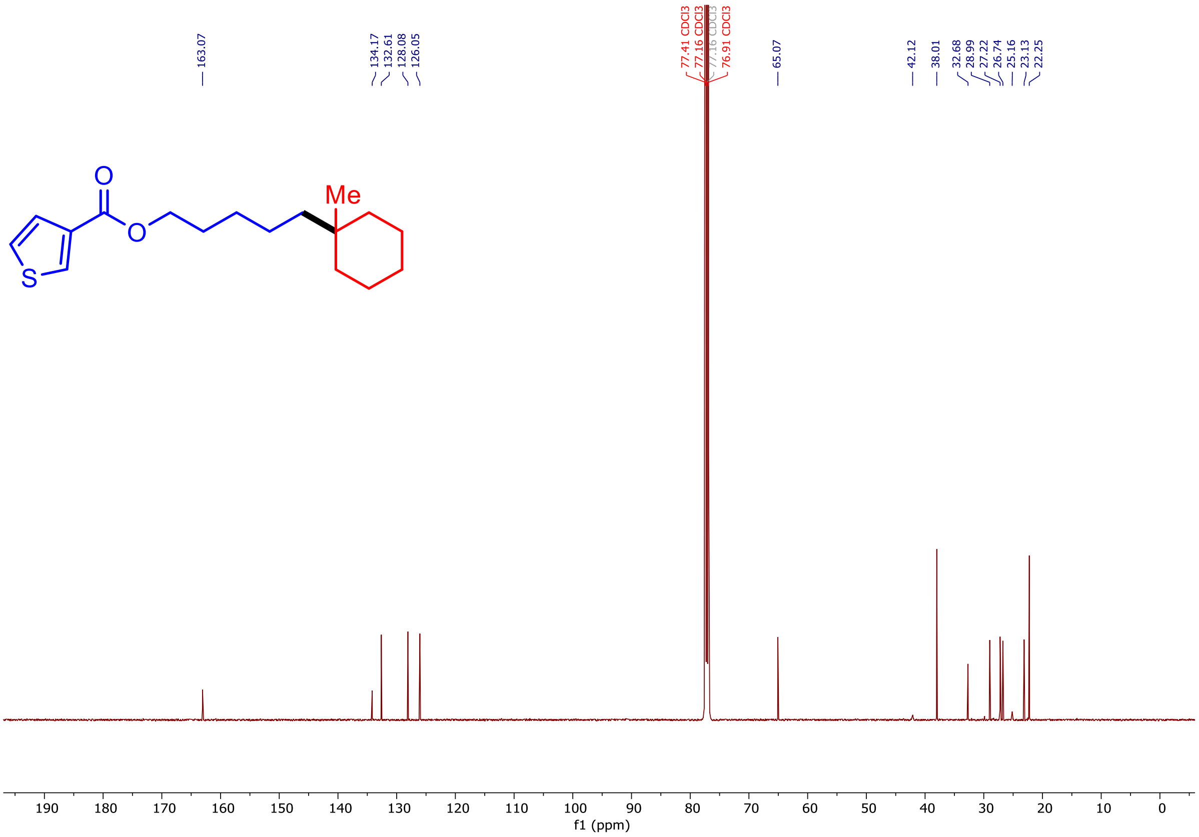

^{1}H NMR (400 MHz, $CDCl_3$) of **38a** (*see procedure*)

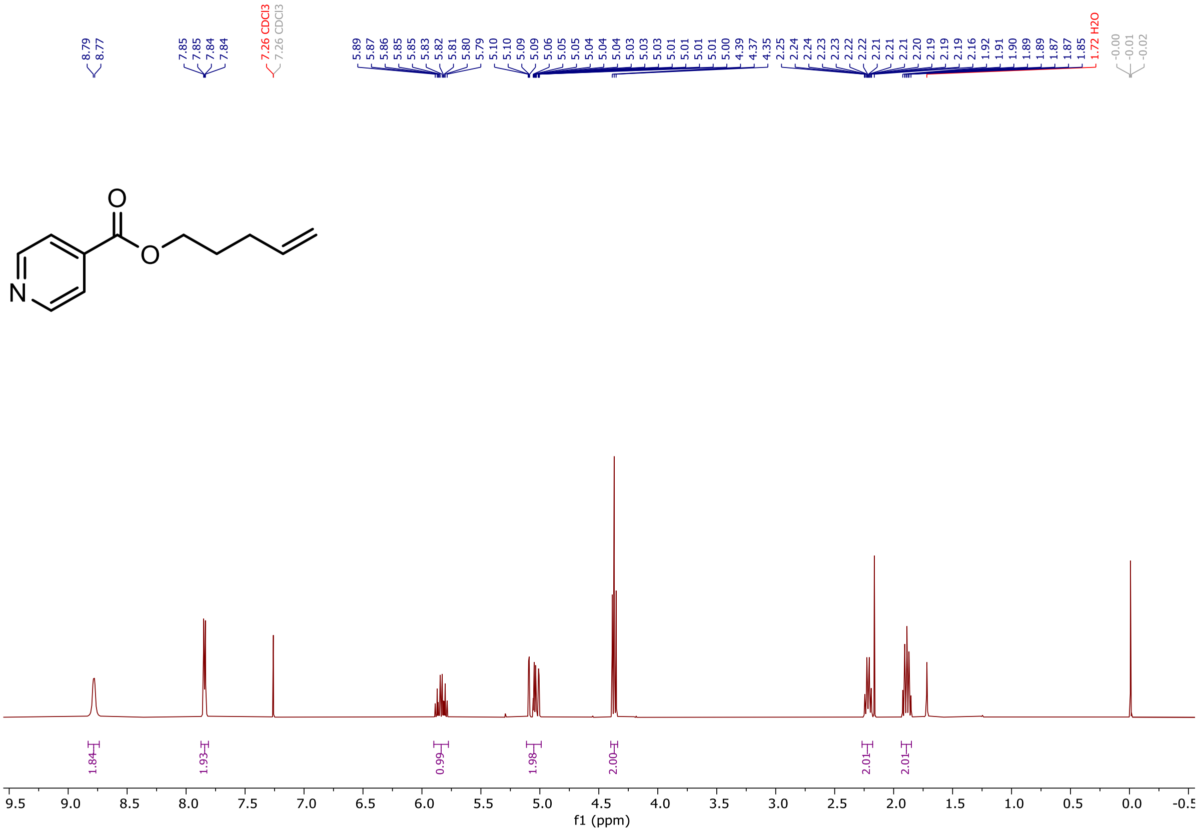

^{13}C NMR (101 MHz, $CDCl_3$) of **38a**

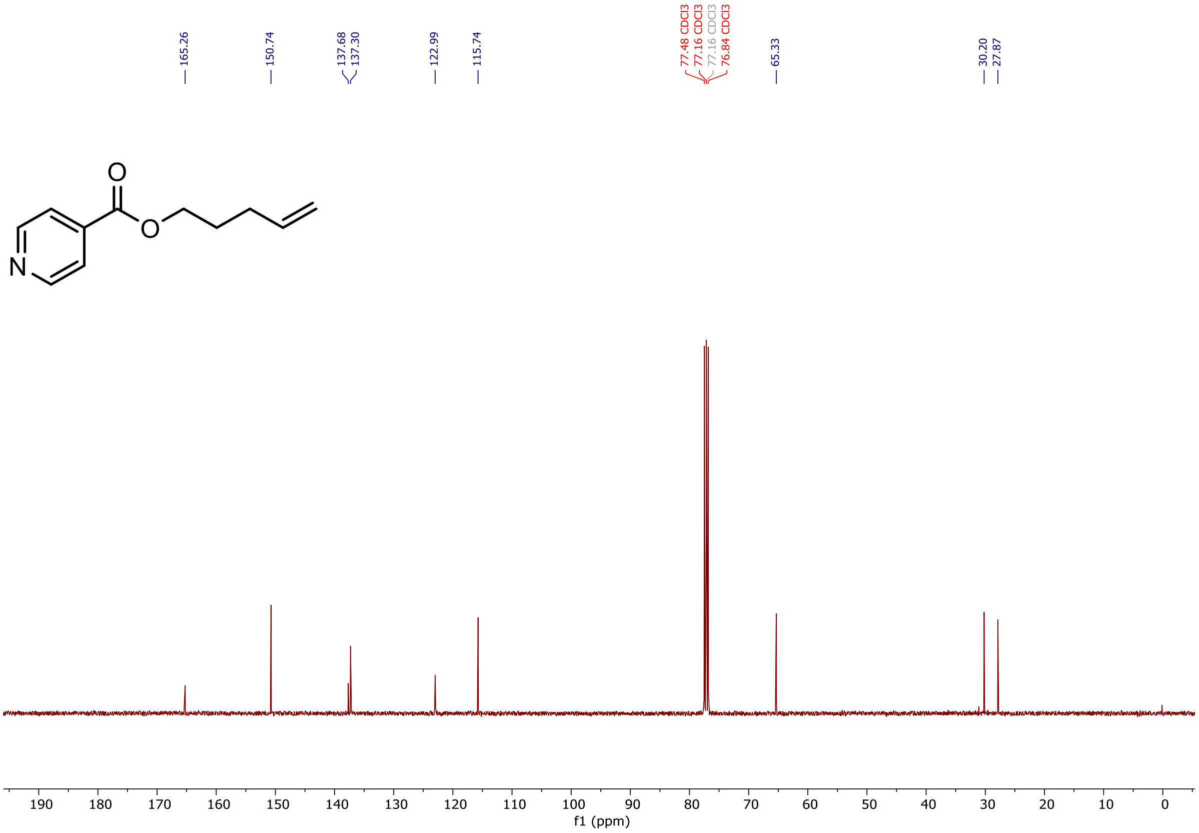

^{1}H NMR (400 MHz, $CDCl_3$) of **38** (*see procedure*)

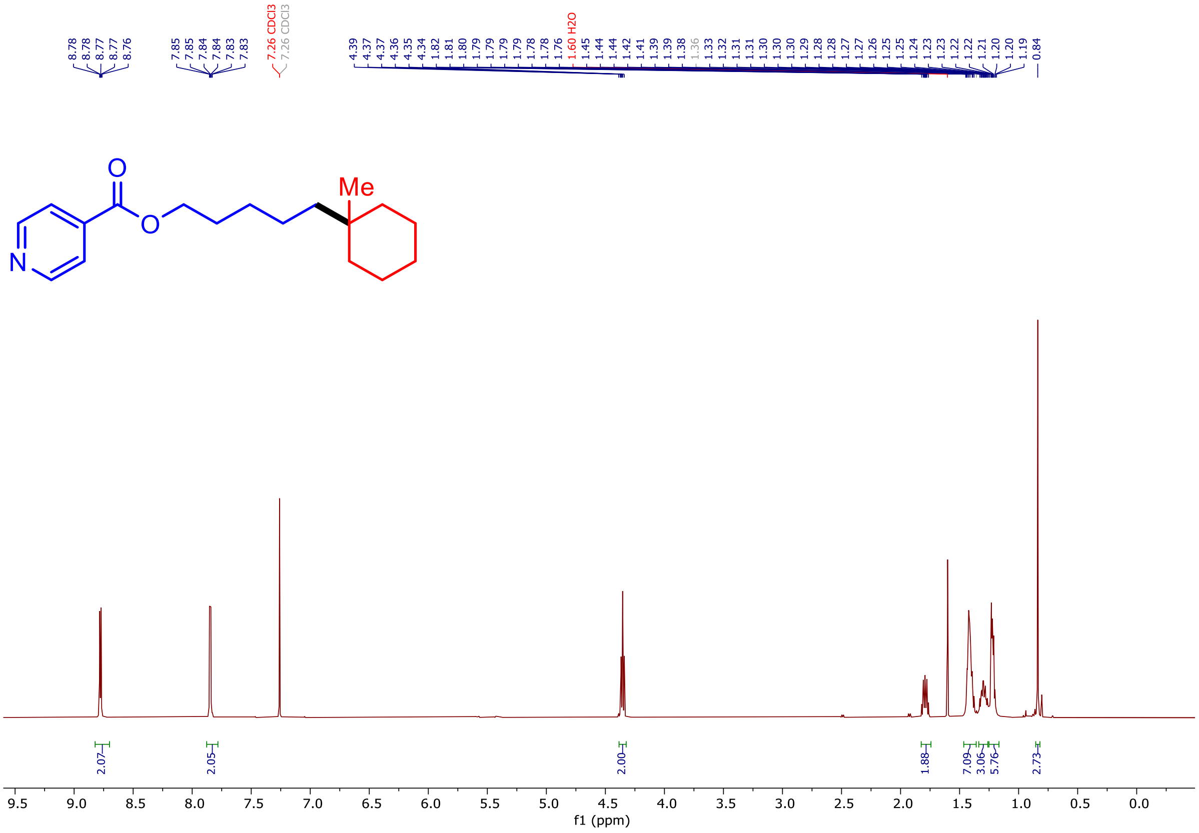

^{13}C NMR (101 MHz, $CDCl_3$) of **38**

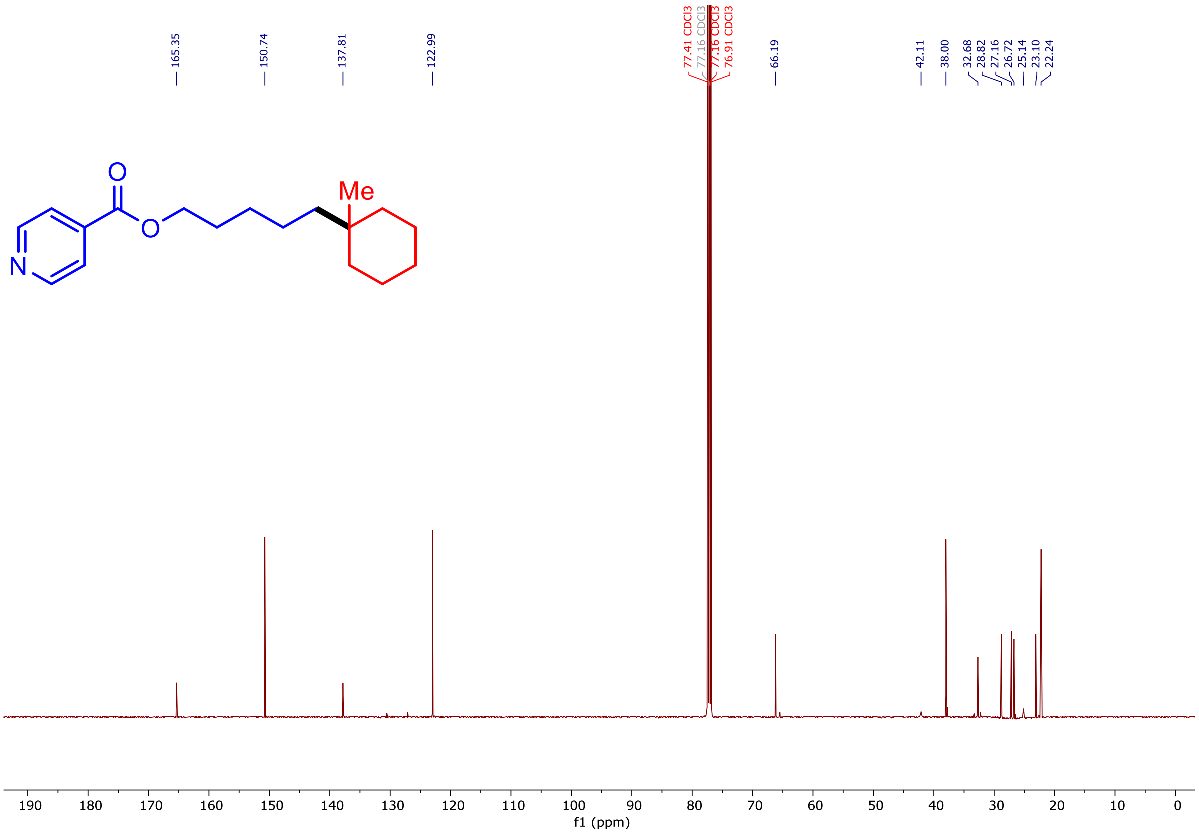

^{1}H NMR (400 MHz, $CDCl_3$) of **39** (***see procedure***)

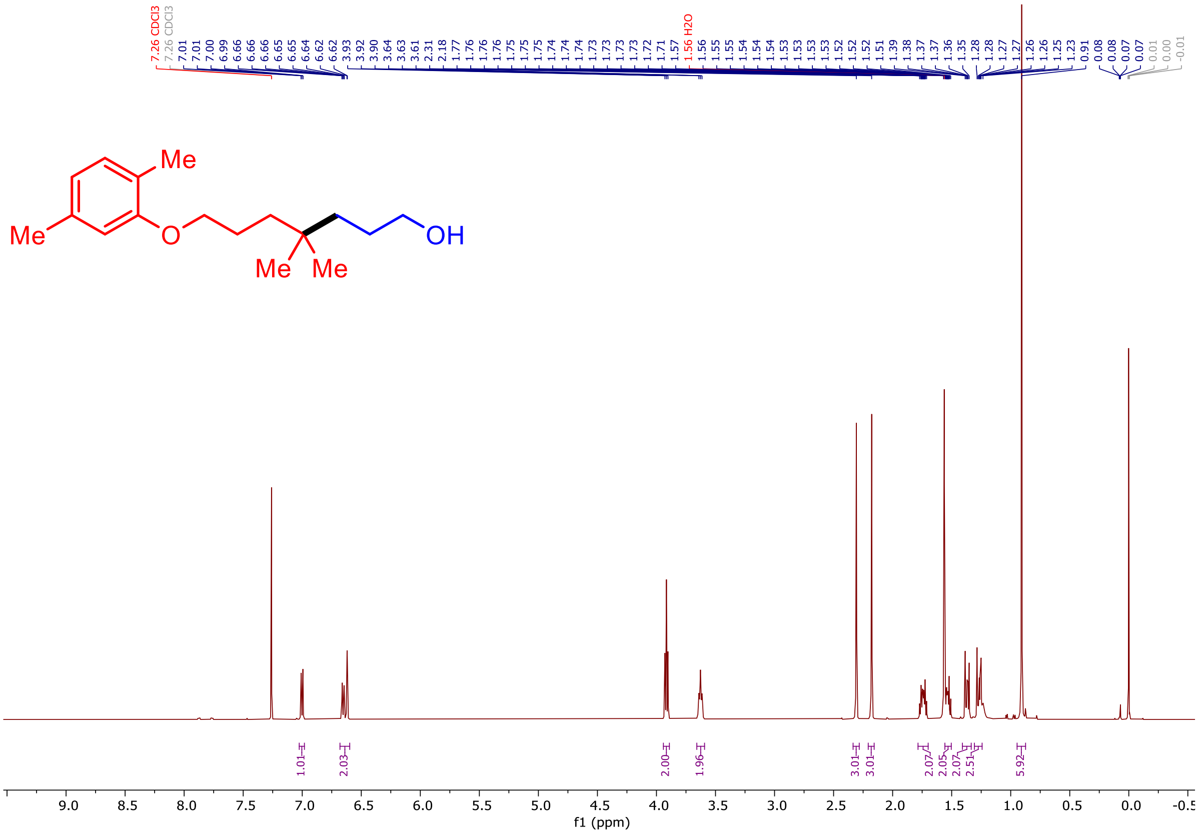

^{13}C NMR (101 MHz, $CDCl_3$) of **39**

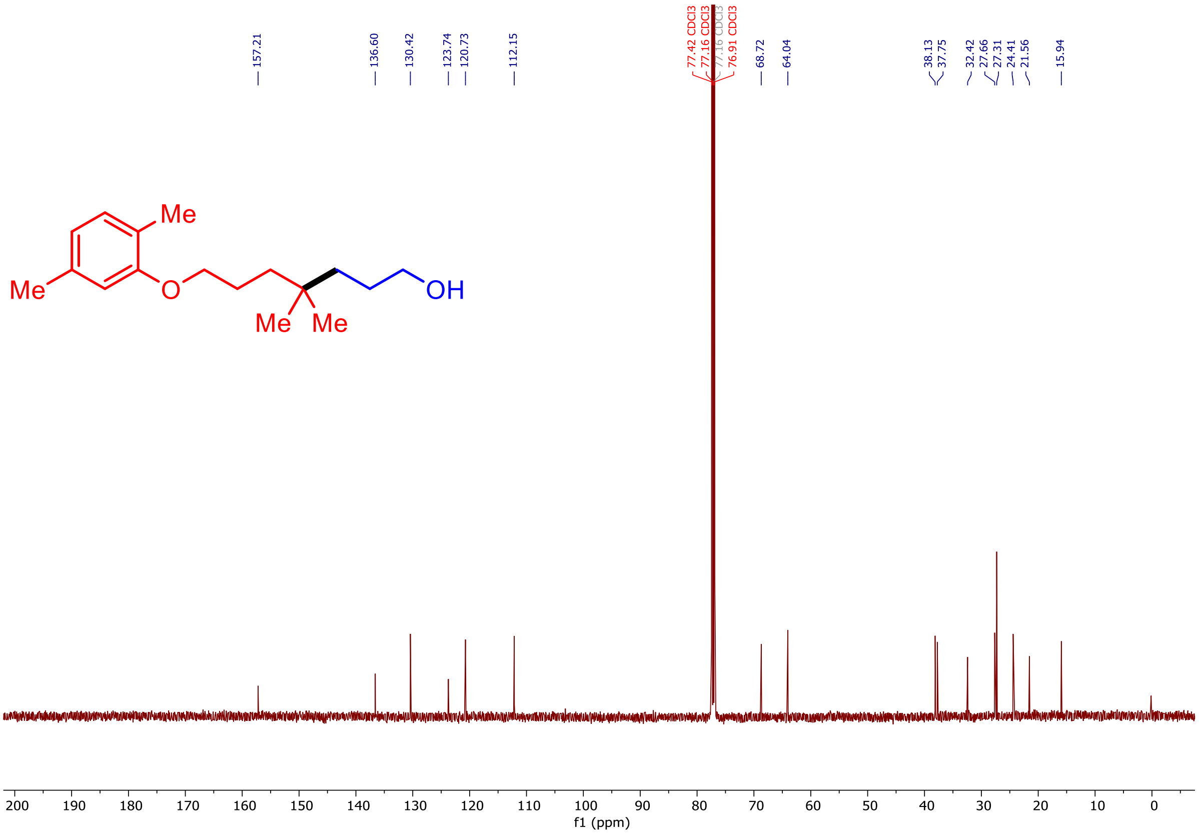

^{1}H NMR (400 MHz, $CDCl_3$) of **40a** (***see procedure***)

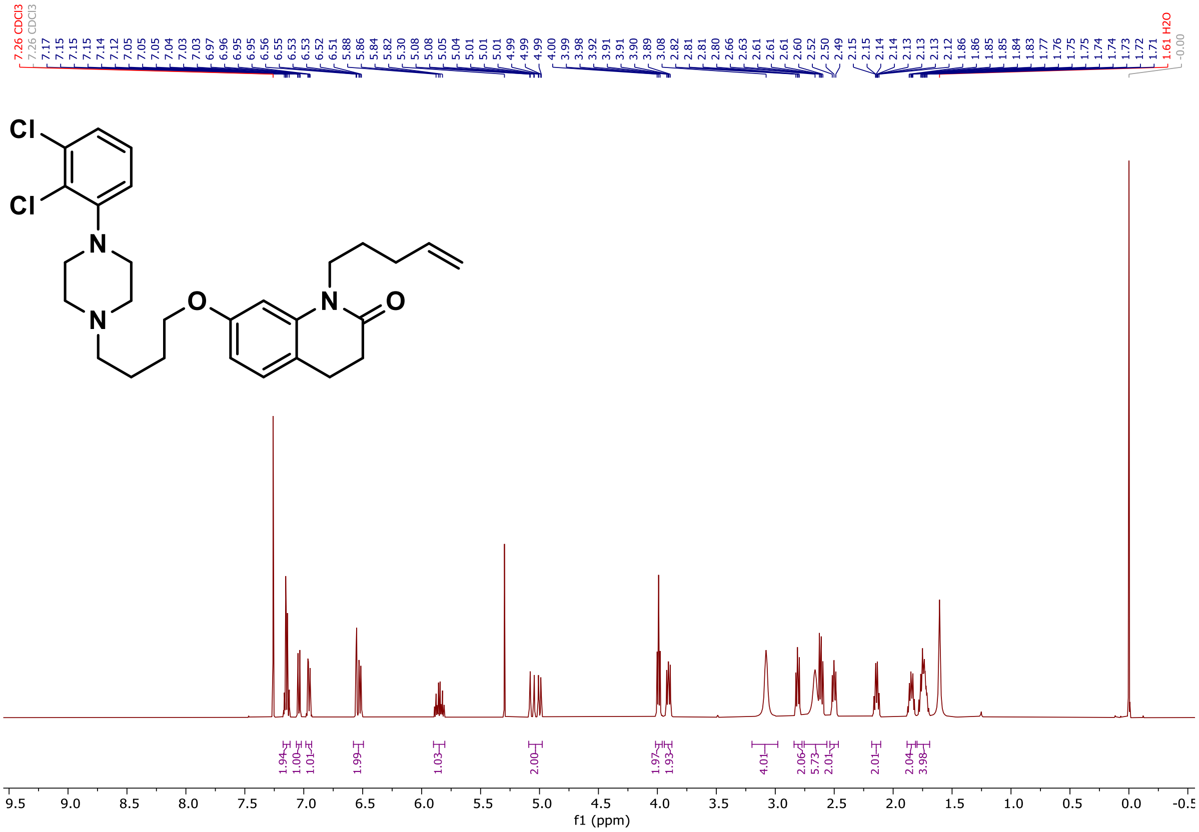

^{13}C NMR (101 MHz, $CDCl_3$) of **40a**

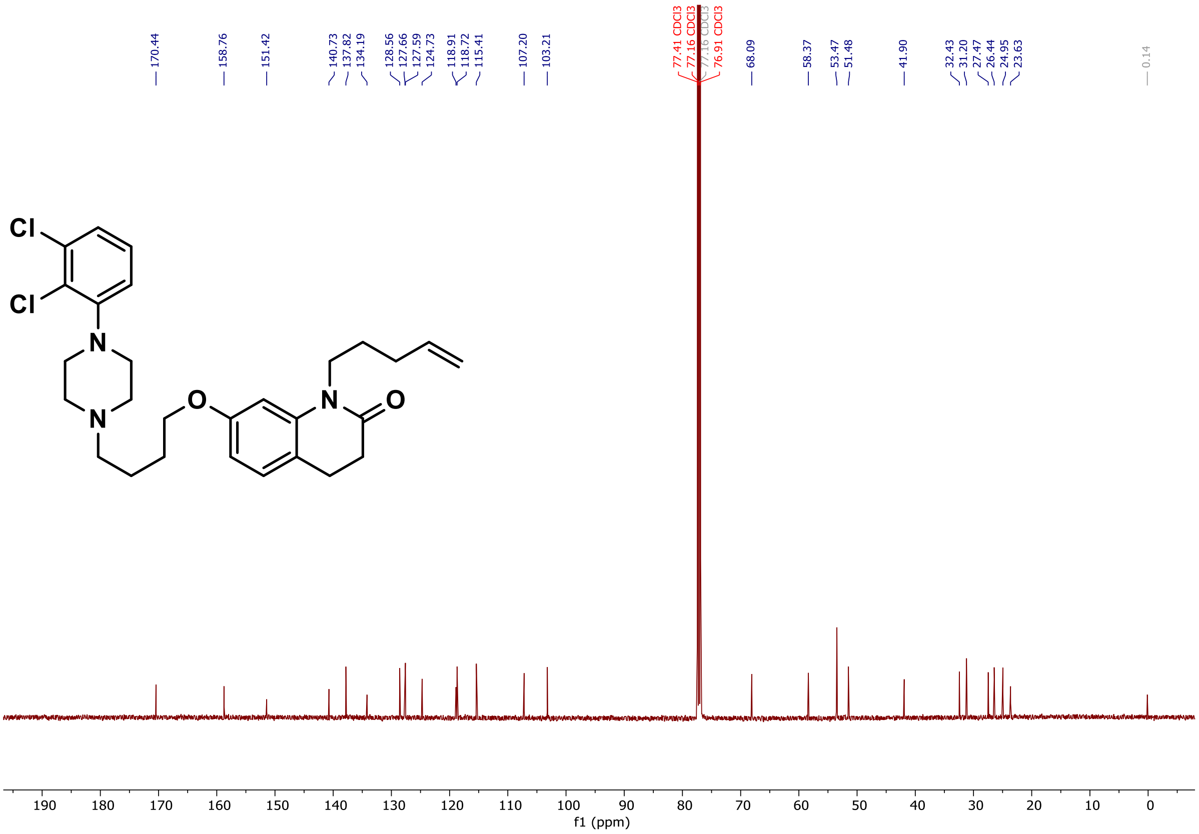

^{1}H NMR (500 MHz, $CDCl_3$) of **40** (***see procedure***)

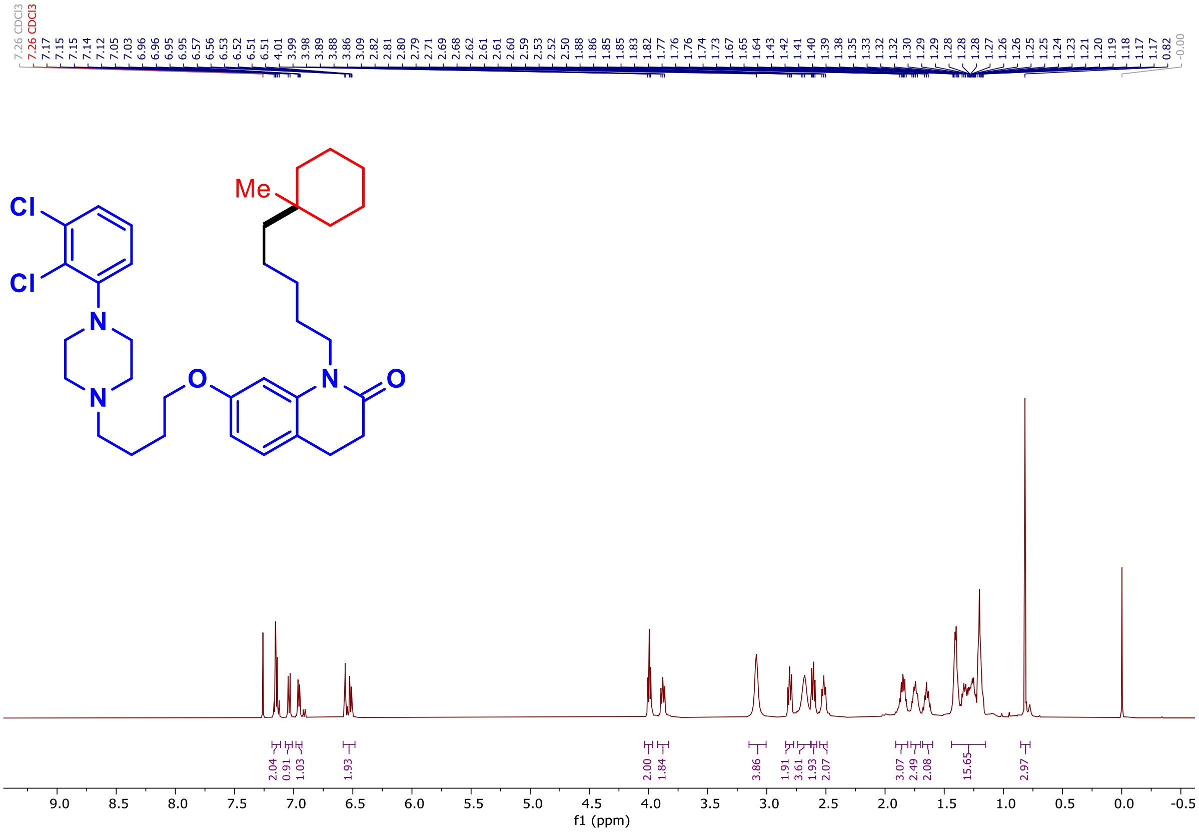

^{13}C NMR (126 MHz, $CDCl_3$) of **40**

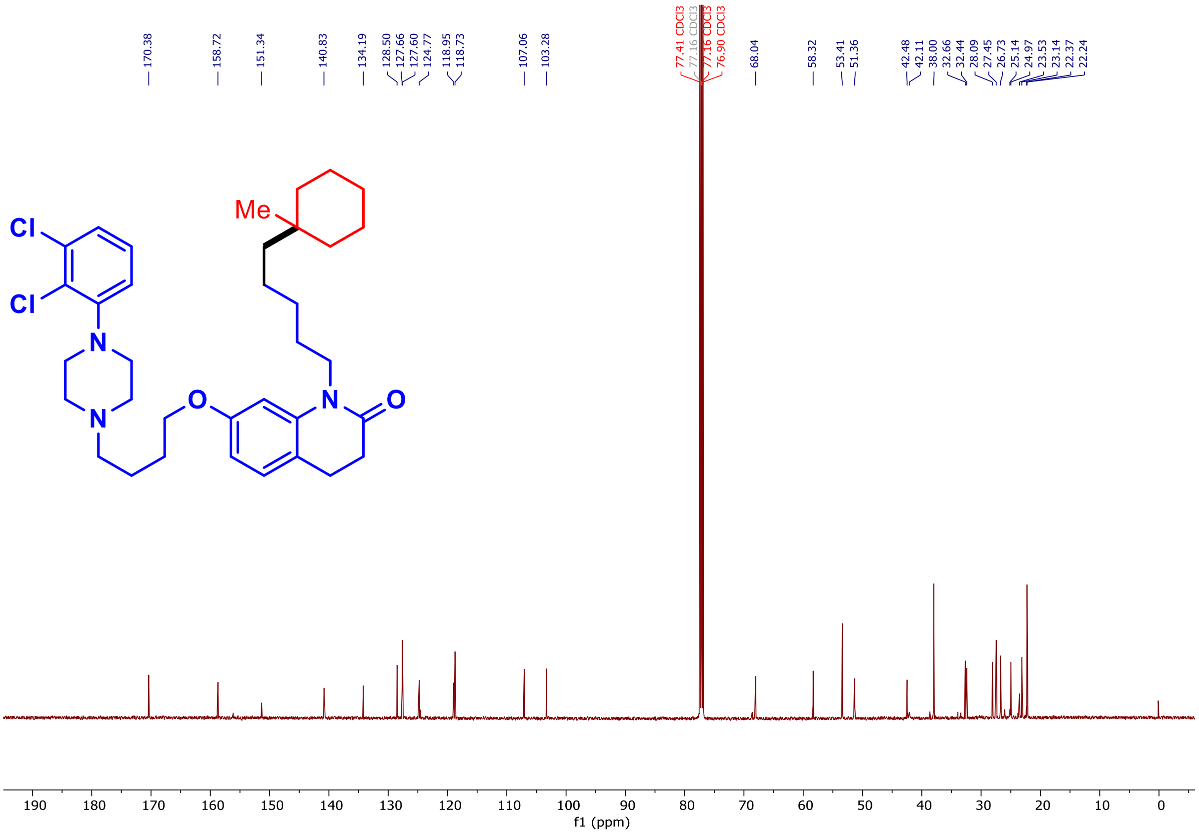

^{1}H NMR (400 MHz, $CDCl_3$) of **41a** (***see procedure***)

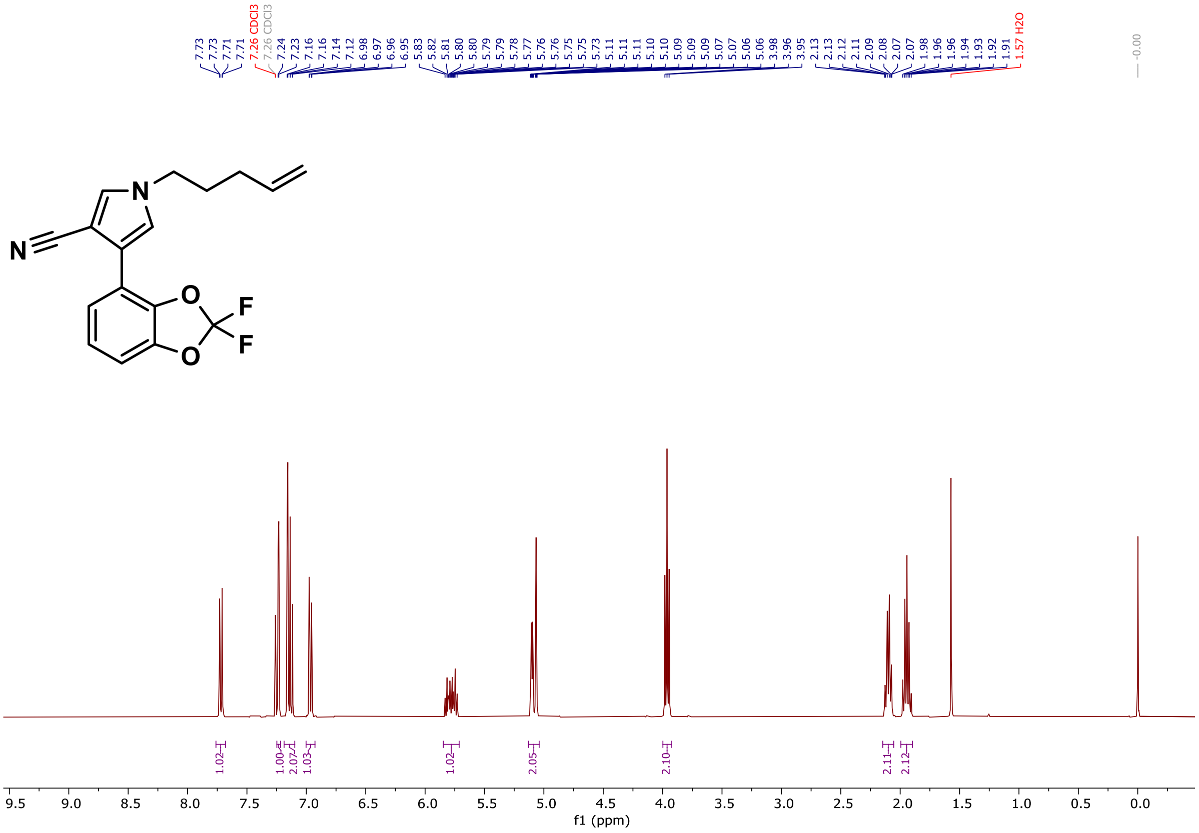

^{13}C NMR (101 MHz, $CDCl_3$) of **41a**

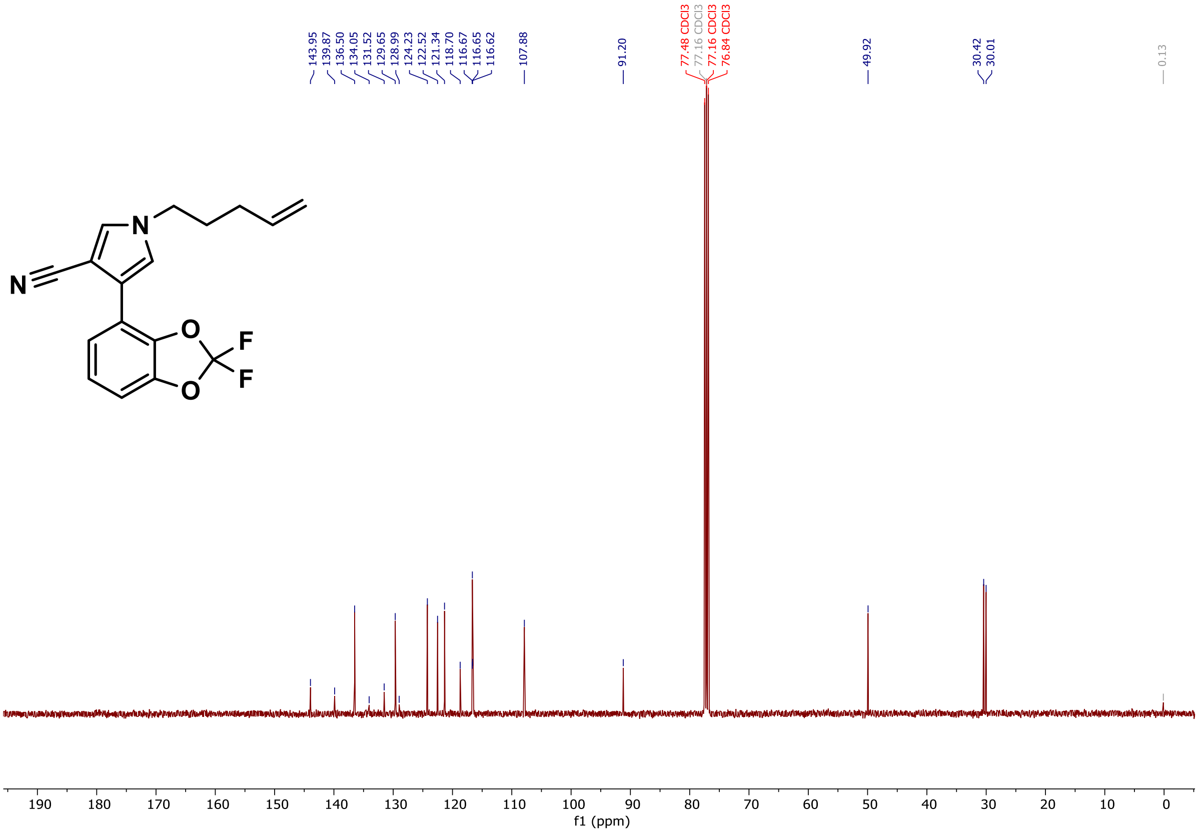

^{1}H NMR (500 MHz, CDCl$_3$) of **41** (*see procedure*)

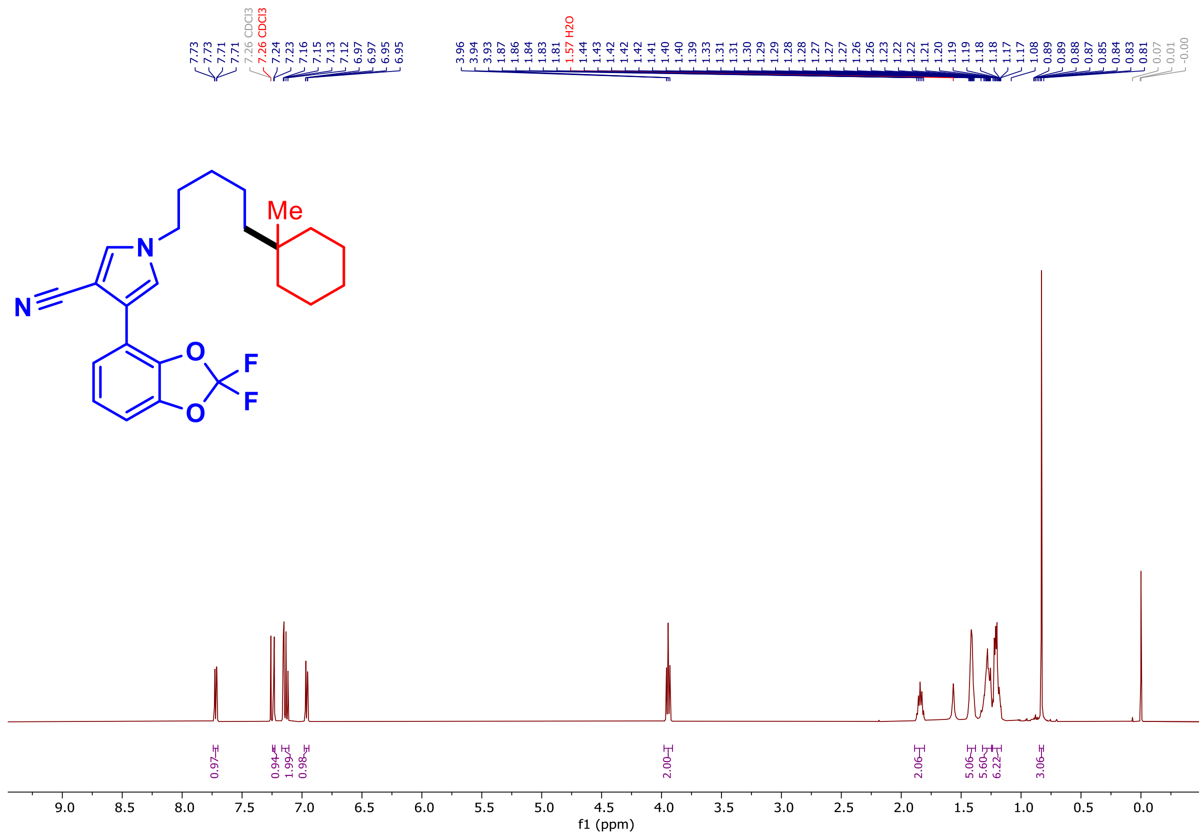

^{13}C NMR (126 MHz, CDCl$_3$) of **41**

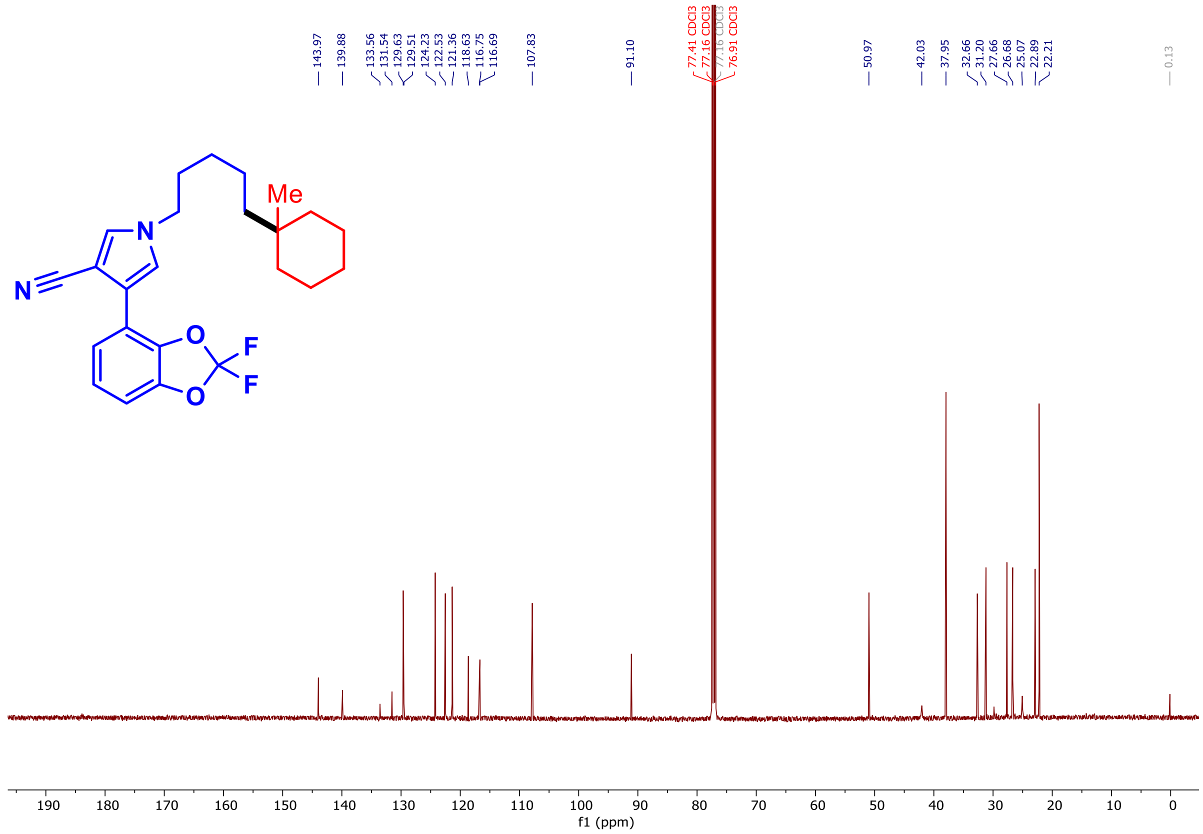

^{19}F NMR (377 MHz, $CDCl_3$) of **41**

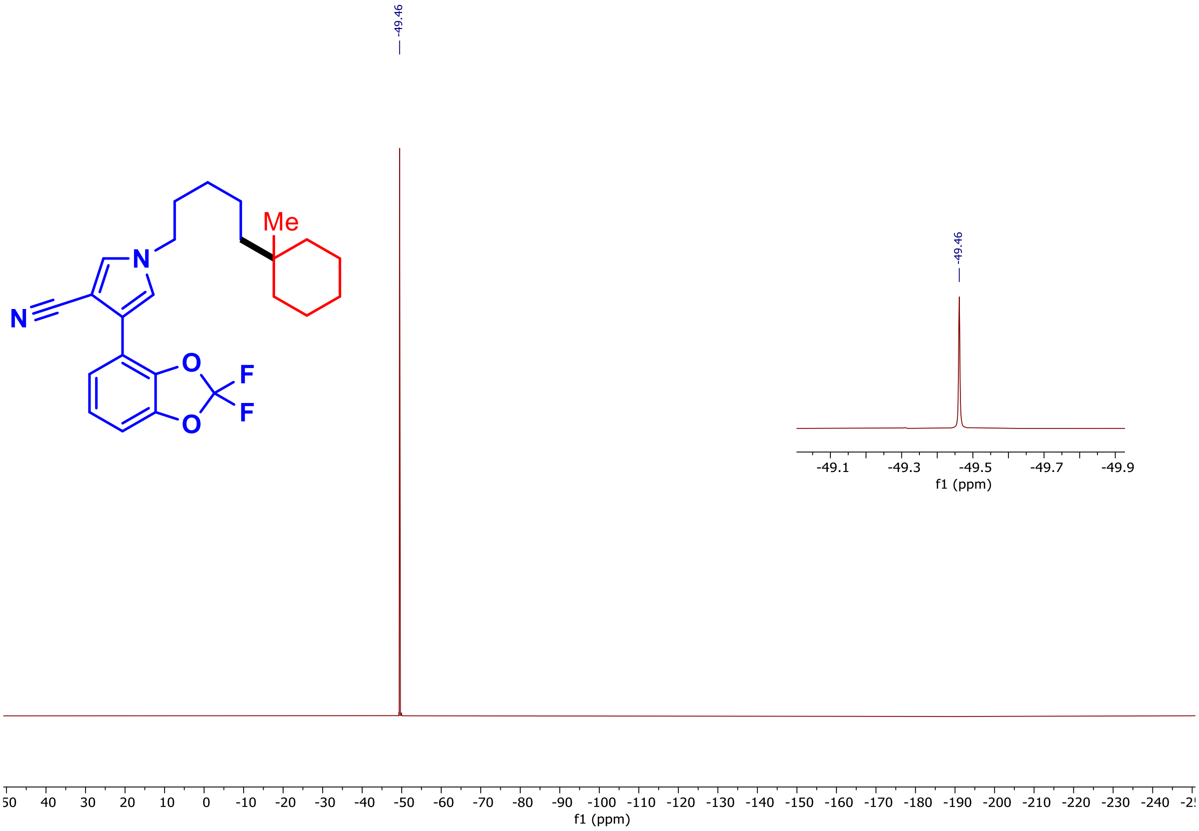

^{1}H NMR (400 MHz, $CDCl_3$) of **44** (***see procedure***)

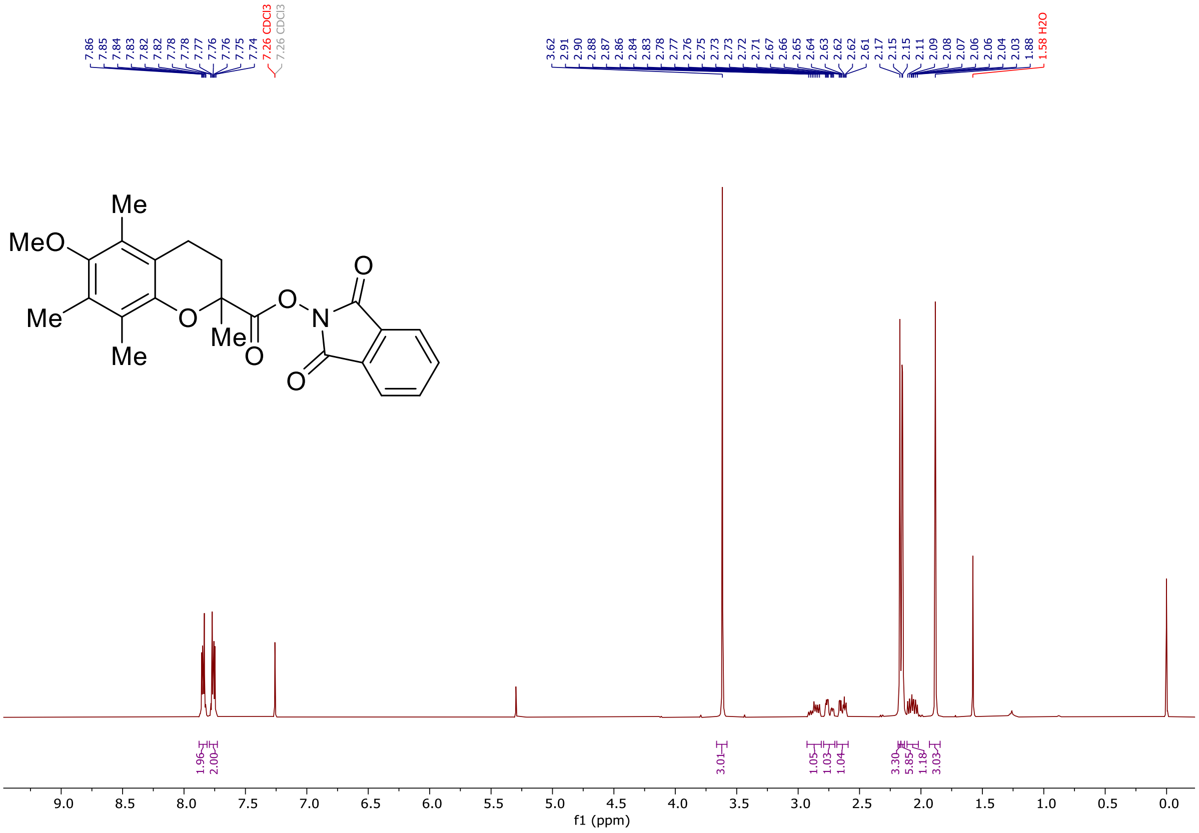

^{13}C NMR (101 MHz, $CDCl_3$) of **44**

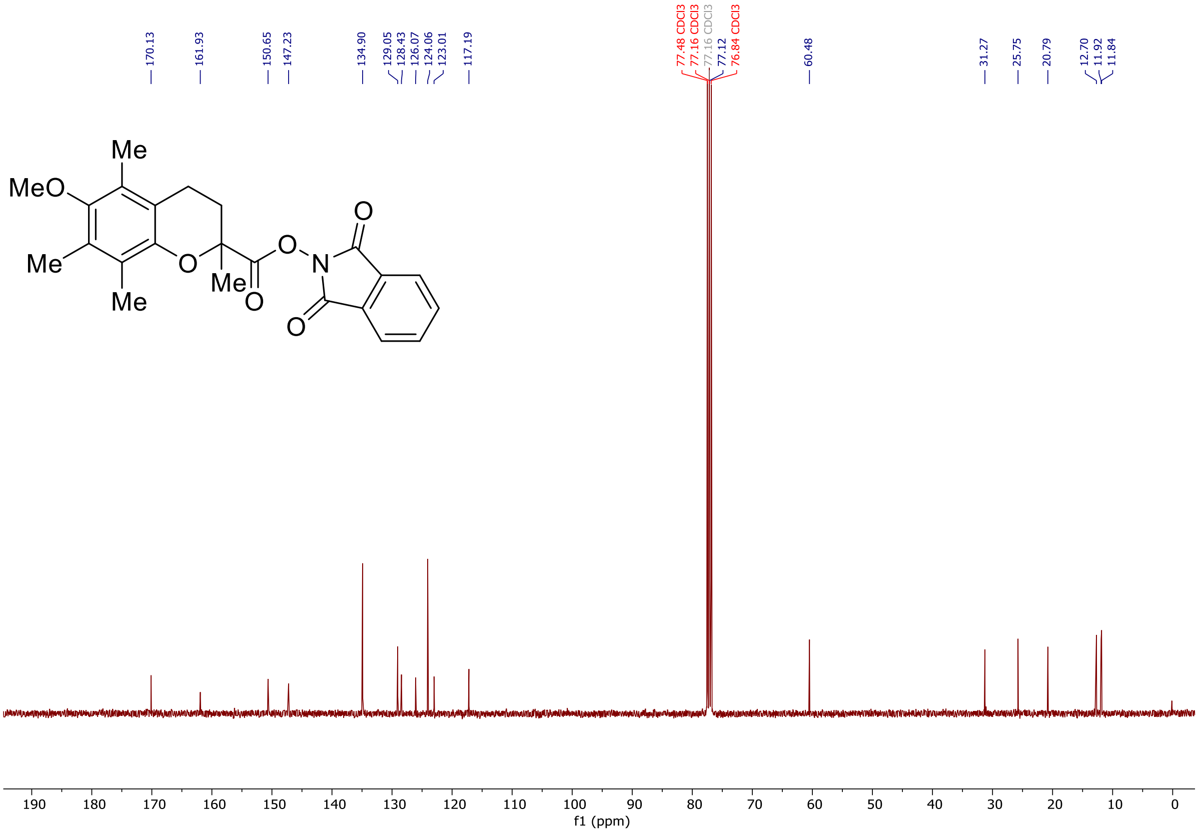

^{1}H NMR (400 MHz, $CDCl_3$) of **45** (*see procedure*)

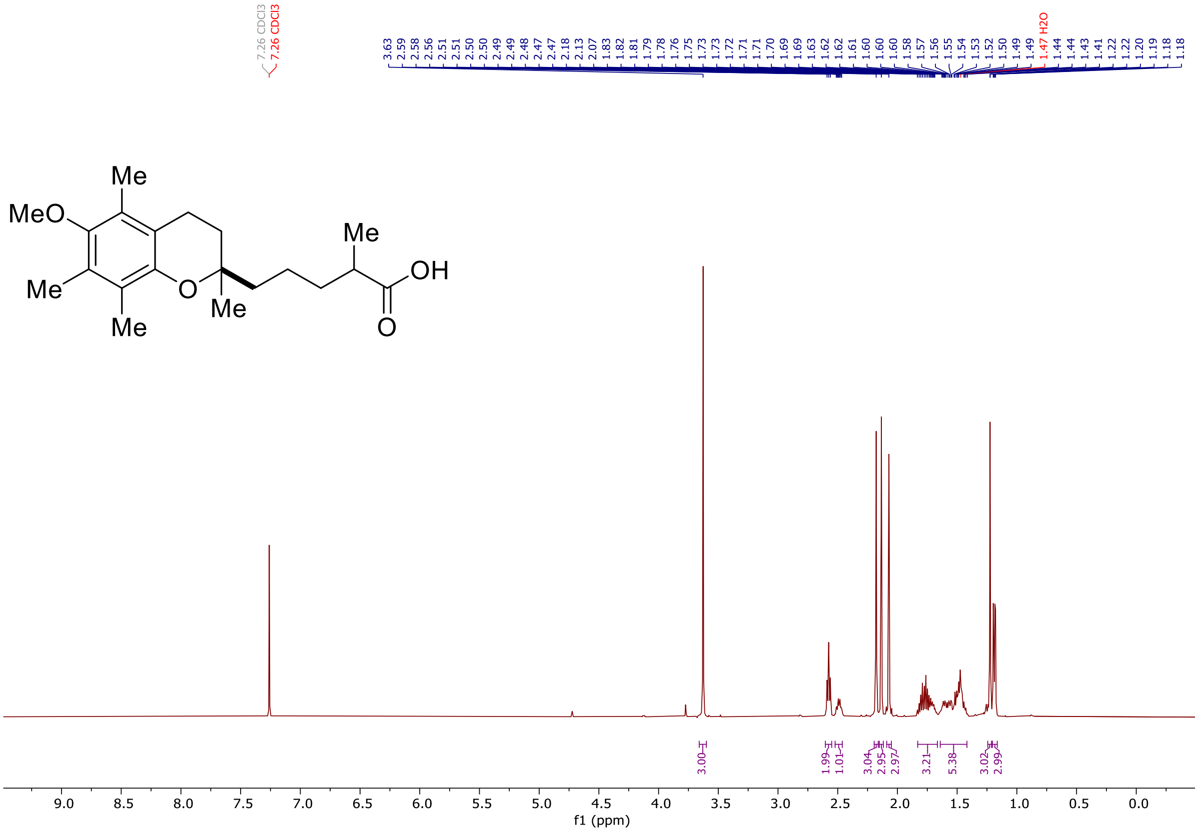

^{13}C NMR (101 MHz, $CDCl_3$) of **45**

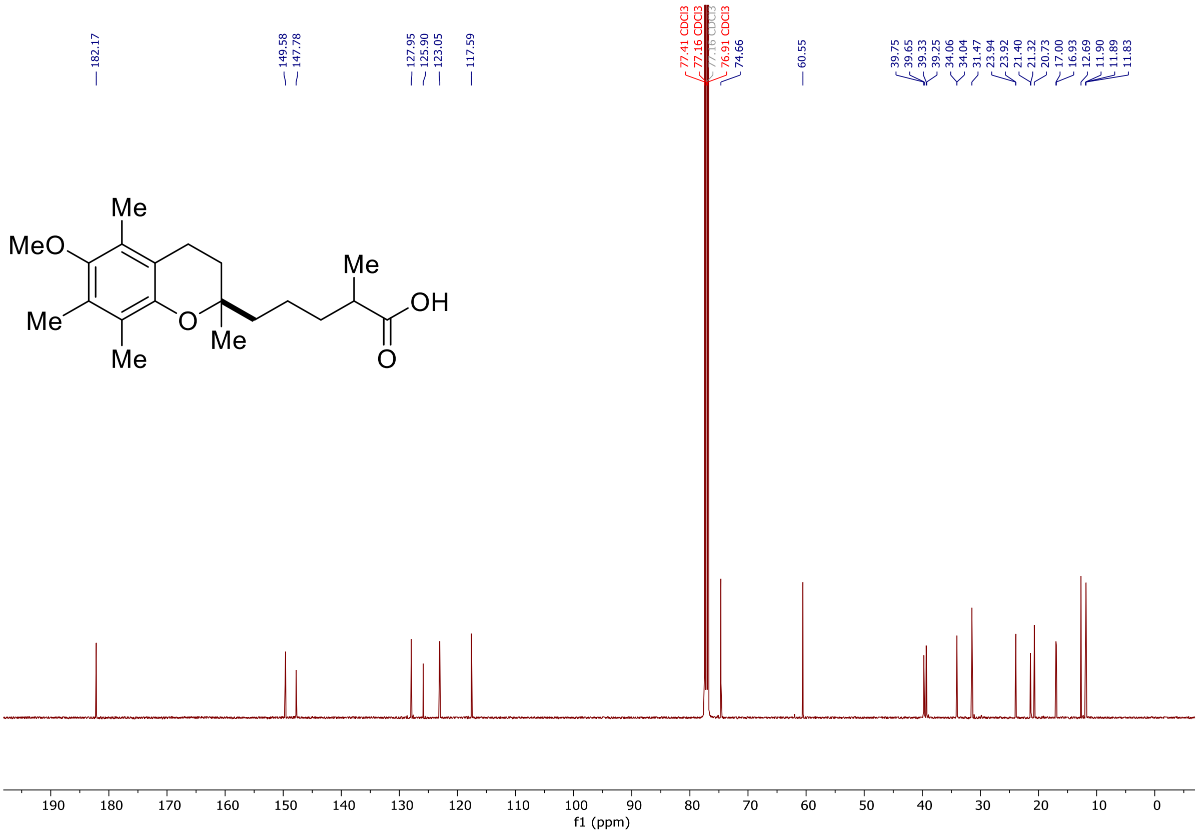

^{1}H NMR (400 MHz, $CDCl_3$) of **48** (*see procedure*)

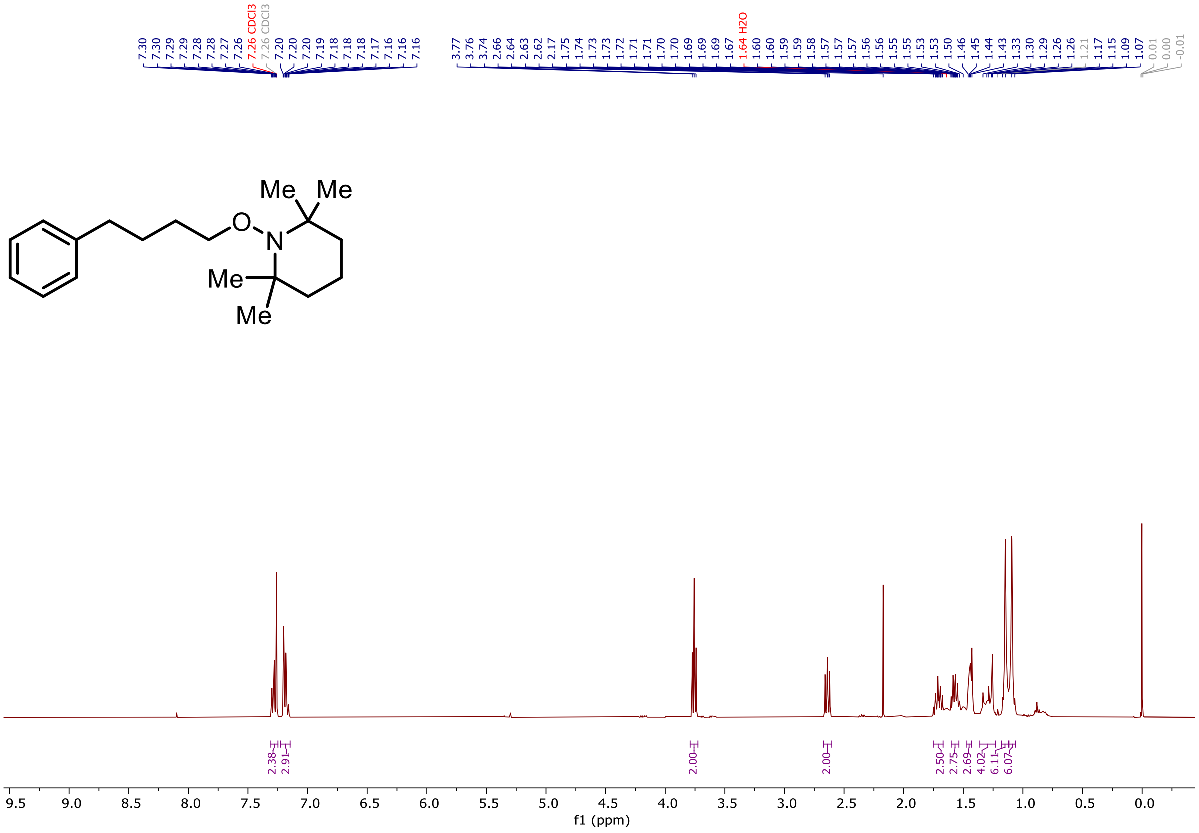

^{13}C NMR (101 MHz, $CDCl_3$) of **48**

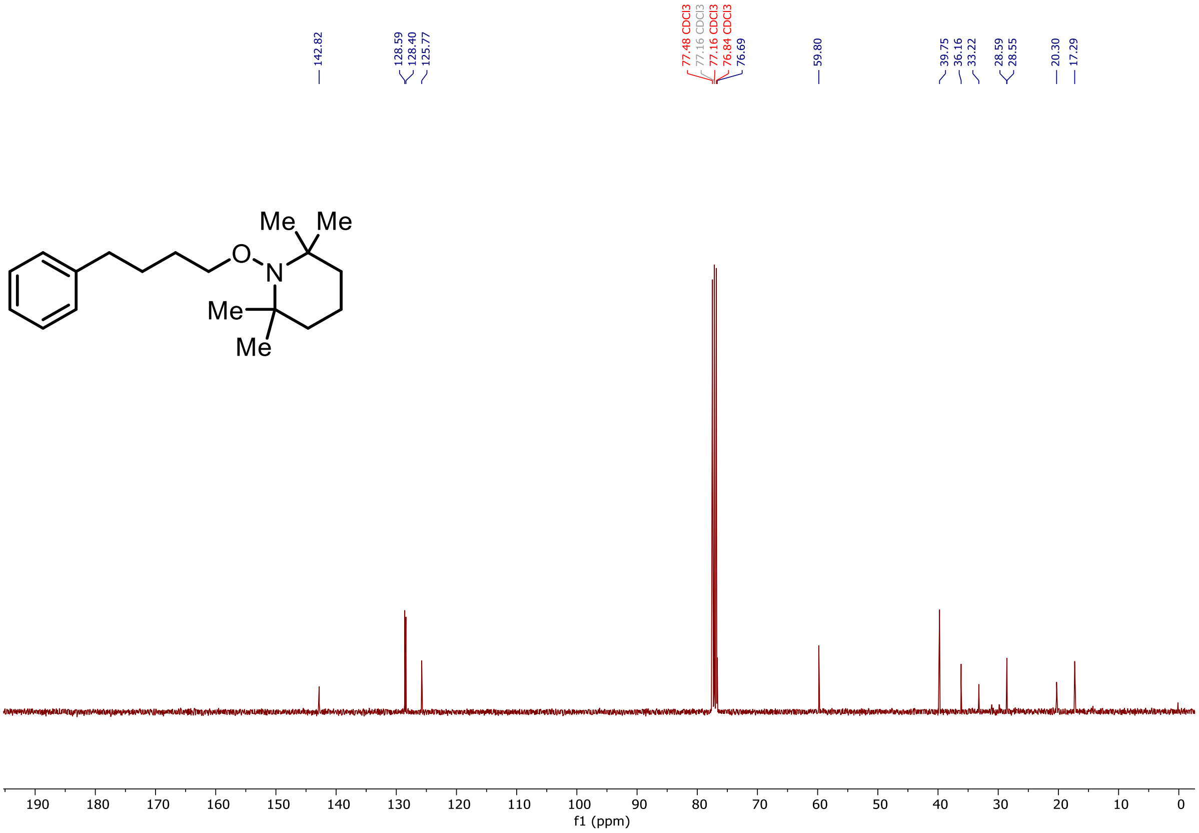

^{1}H NMR (400 MHz, $CDCl_3$) of **51** (*see procedure*)

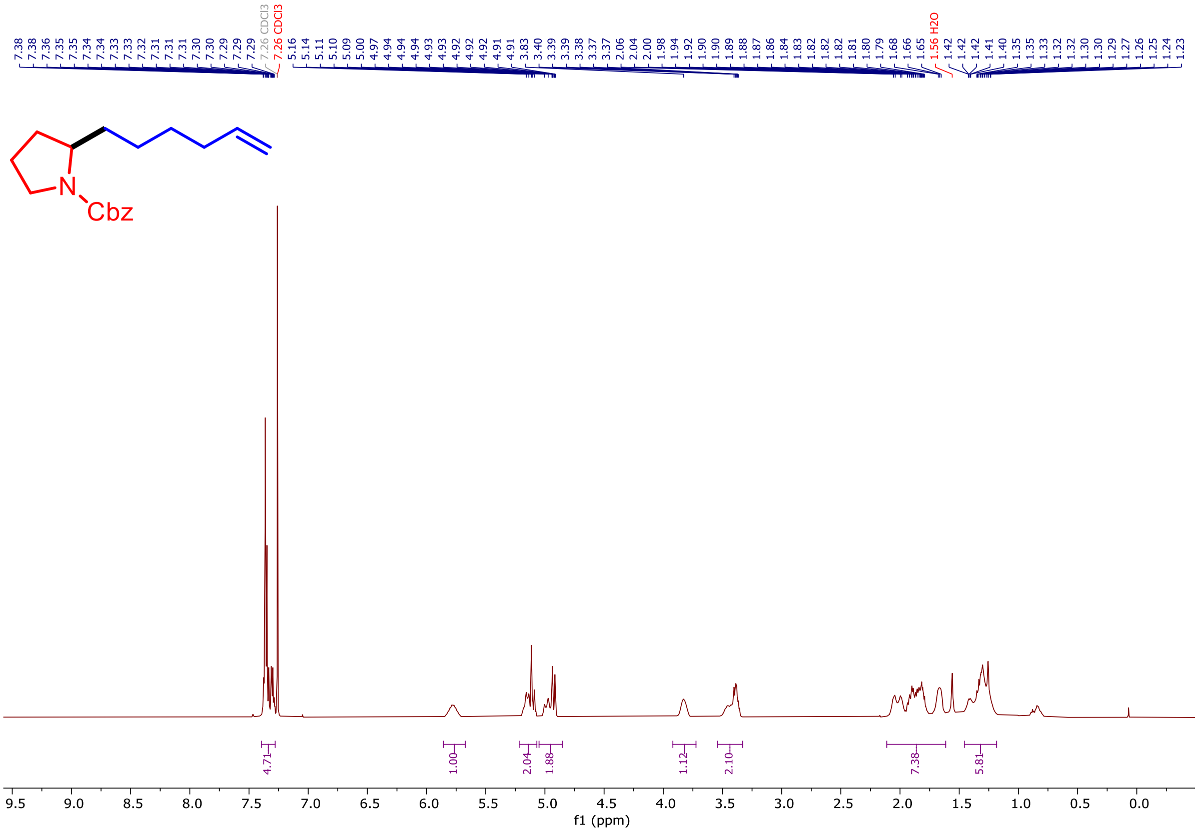

^{13}C NMR (101 MHz, $CDCl_3$) of **51**

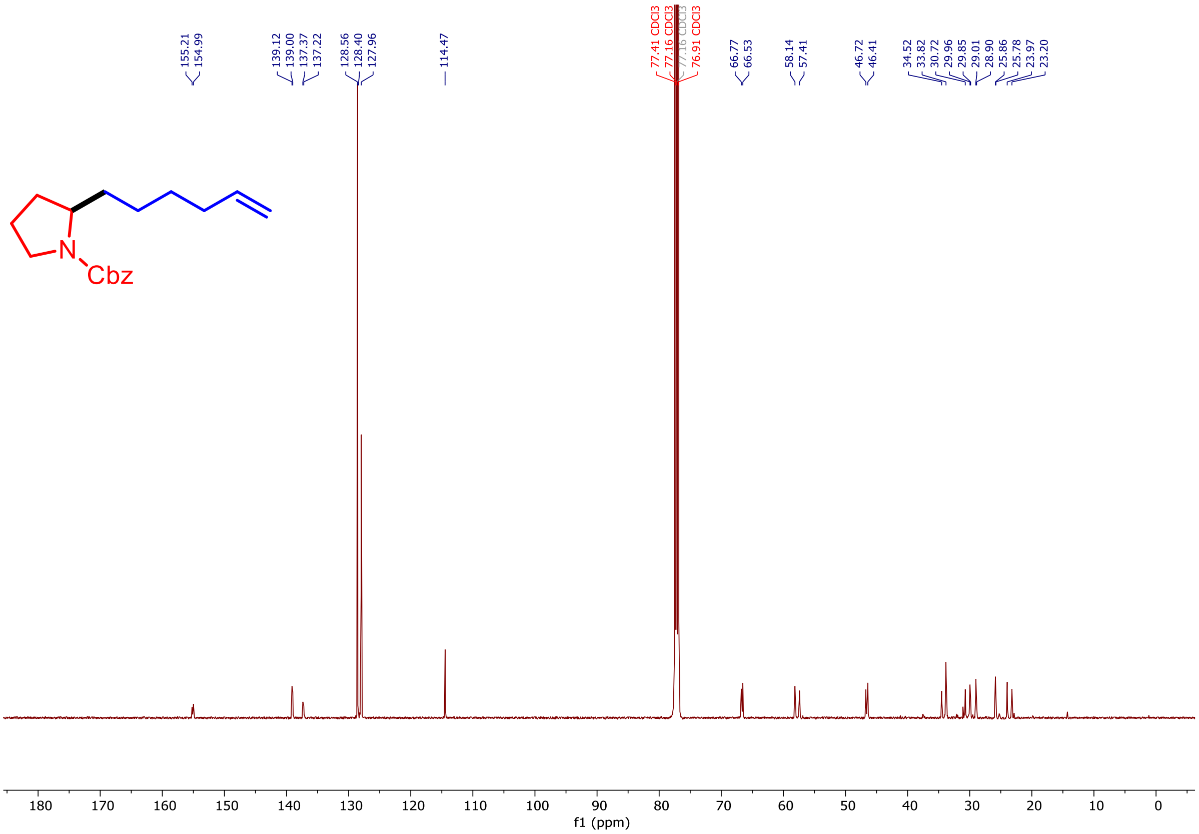

^1H NMR (400 MHz, $CDCl_3$) of **52** (*see procedure*)

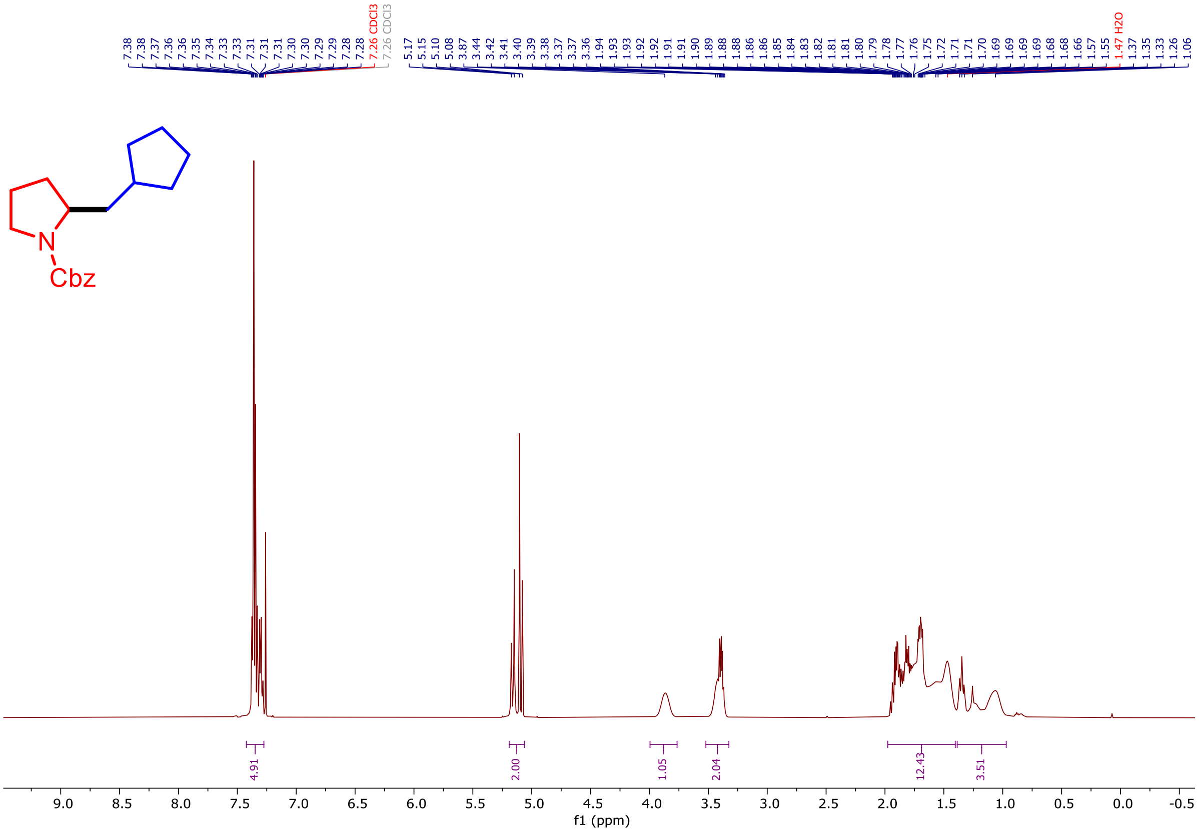

^{13}C NMR (101 MHz, $CDCl_3$) of **52**

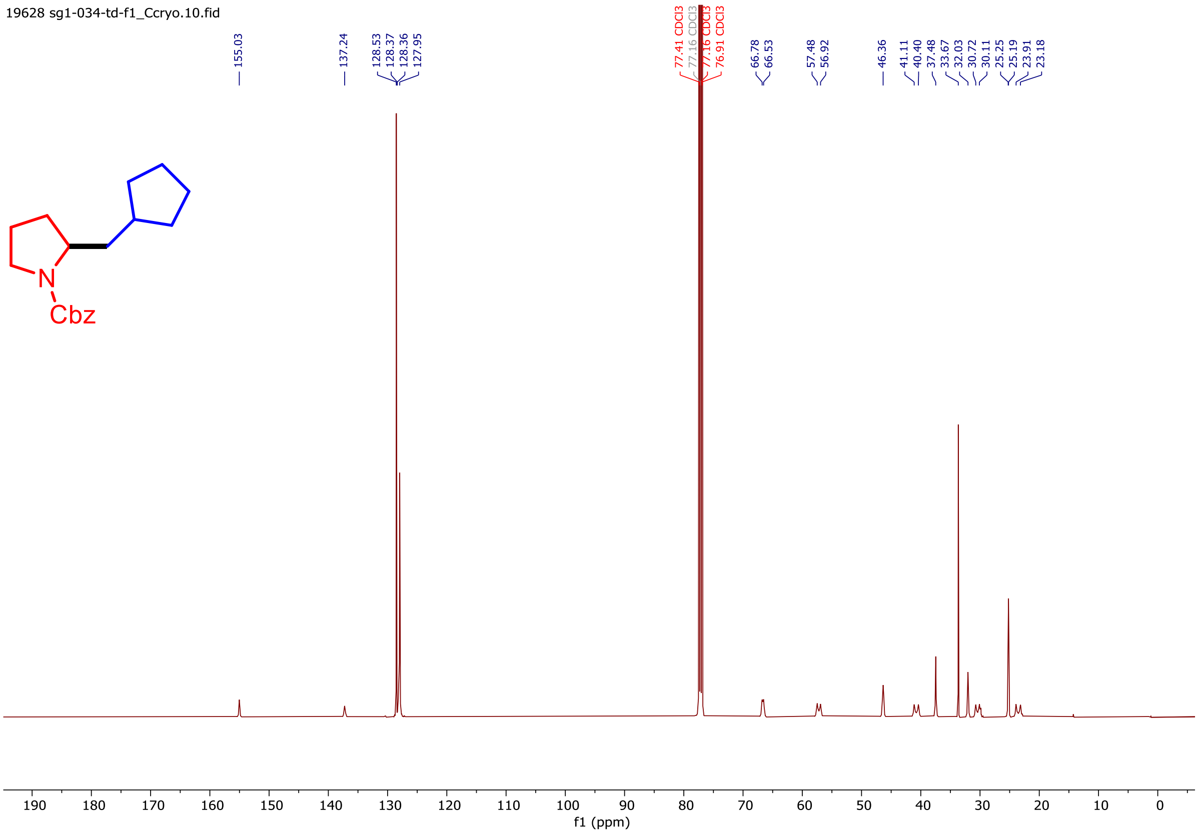

5. REFERENCES

1. Zheng, C.; Wang, Y.; Xu, Y.; Chen, Z.; Chen, G.; Liang, S. H. Ru-Photoredox-Catalyzed Decarboxylative Oxygenation of Aliphatic Carboxylic Acids through N-(acyloxy)phthalimide. *Org. Lett.* **2018**, *20*, 4824–4827.
2. Santos, M. S.; Corrêa, A. G.; Paixão, M. W.; König, B. C(sp^3)−C(sp^3) Cross-Coupling of Alkyl Bromides and Ethers Mediated by Metal and Visible Light Photoredox Catalysis. *Adv. Synth. Catal.* **2020**, *362*, 2367–2372.
3. Gan, X-C.; Zhang, B.; Dao, N.; Bi, C.; Pokle, M.; Kan, L.; Collins, M. R.; Tyrol, C. C.; Bolduc, P. N.; Nicastri, M.; Kawamata, Yu.; Baran, P. S.; Shenvi, R. A. Carbon Quaternization of Redox Active Esters and Olefins by Decarboxylative Coupling. *Science* **2024**, *384*, 113−118.
4. Heiny, M; Shastri, V. P. Cyclic Comonomers for the Synthesis of Carboxylic Acid and Amine Functionalized Poly(*l*-Lactic Acid). *Molecules* **2015**, *20*, 4764−4779.
